# CO_2_ Unlocks Reactivity: Boryl Silyl Ketene Acetals Enable Mild and Direct C=C Bond Cleavage

**DOI:** 10.1021/jacs.5c16770

**Published:** 2025-12-15

**Authors:** Noel Angel Espinosa-Jalapa, Manuel Kümper, Jonathan O. Bauer

**Affiliations:** Faculty of Chemistry and Pharmacy, Institute of Inorganic Chemistry, 199842 University of Regensburg, Universitätsstraße 31, D-93053 Regensburg, Germany

## Abstract

Boron–ligand cooperation (BLC) has emerged as a powerful principle of bond activation with main-group elements, yet pyridine-based systems have so far eluded experimental evidence of CO_2_ activation. We show here that four-membered pyridyl–boracycles activate CO_2_ through a dearomatizing boron–carbon bond cleavage, unambiguously proceeding by a BLC rather than a B/N-FLP-type mechanism, as confirmed by density functional theory (DFT) studies, in contrast to previously predicted computational pathways. This process furnishes boryl silyl ketene acetals, a hitherto unknown class of enolate equivalents in which the two oxygen atoms are differentiated by boryl and silyl substituents. These intermediates exhibit remarkable follow-up reactivity, eventually leading to a mild, one-step C=C double-bond cleavage that delivers fulvene derivatives under additive-free conditions, thereby constituting an unprecedented form of metathesis. Overall, our findings establish boryl silyl ketene acetals derived from CO_2_ as a novel class of main-group systems that unlock a reactivity platform with far-reaching synthetic implications.

## Graphical abstract

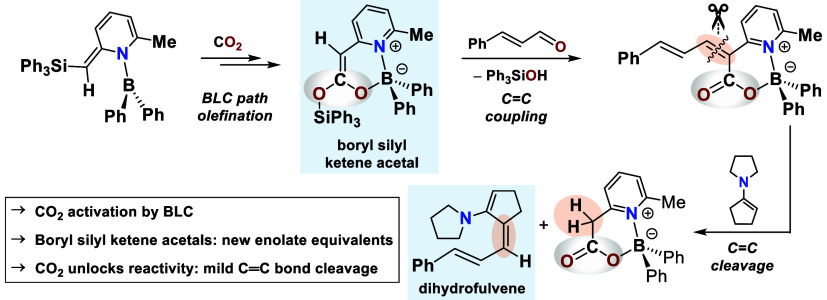

## Introduction

Element–ligand cooperation has recently emerged as a powerful paradigm for bond activation and catalysis with main-group element compounds.^1–11^ In such systems, the main-group element and the surrounding molecular framework (ligand) act in genuine synergy, with the ligand actively engaging in bond activation.^1–3^ In analogy to metal–ligand cooperation via aromatization/dearomatization in Milstein’s pyridine-based pincer complexes,^12–17^ boron–ligand cooperation (BLC) is more specifically defined as the intramolecular interplay of a Lewis-acidic boron center with an aromatic backbone.^2^ This unique manifestation of intramolecular frustrated Lewis pairs (FLPs)^18–21^ involves a dynamic reorganization of π-electron density within the cooperative motif and a switch from covalent to dative B–X interactions during bond activation.^2^

Following pioneering contributions on cooperative boron–ligand systems,^22–24^ pyridine-based boranes have attracted increasing attention as promising metal-free analogues of transition-metal pincer complexes for applications in bond activation processes (Figure 1,A–C).^25^ van der Vlugt demonstrated the stabilization of a dearomatized boron–pyridyl complex by intramolecular donor coordination (Figure 1,A),^26^ while Milstein and co-workers expanded this concept to explicitly involve the boron center in cooperative bond activation (Figure 1,B).^27^ The additional donor side arm essentially “locks” the dearomatized form in both examples A and B. Gellrich subsequently showcased the reversible activation of dihydrogen by pyridonate boranes (Figure 1,C),^28^ firmly establishing BLC as part of the catalytic toolbox.^29–33^ In parallel, substantial progress has been made in the capture, activation, and chemical utilization of CO_2_ using main-group element systems.^34–47^ Yet, despite its clear potential also in this area,^48–52^ ligand cooperation with main-group elements remains in its infancy. While pyridine-based transition-metal complexes readily mediate small-molecule activation, including CO_2_,^53–56^ analogous pyridine-based main-group systems have not been realized.

**Figure 1:**
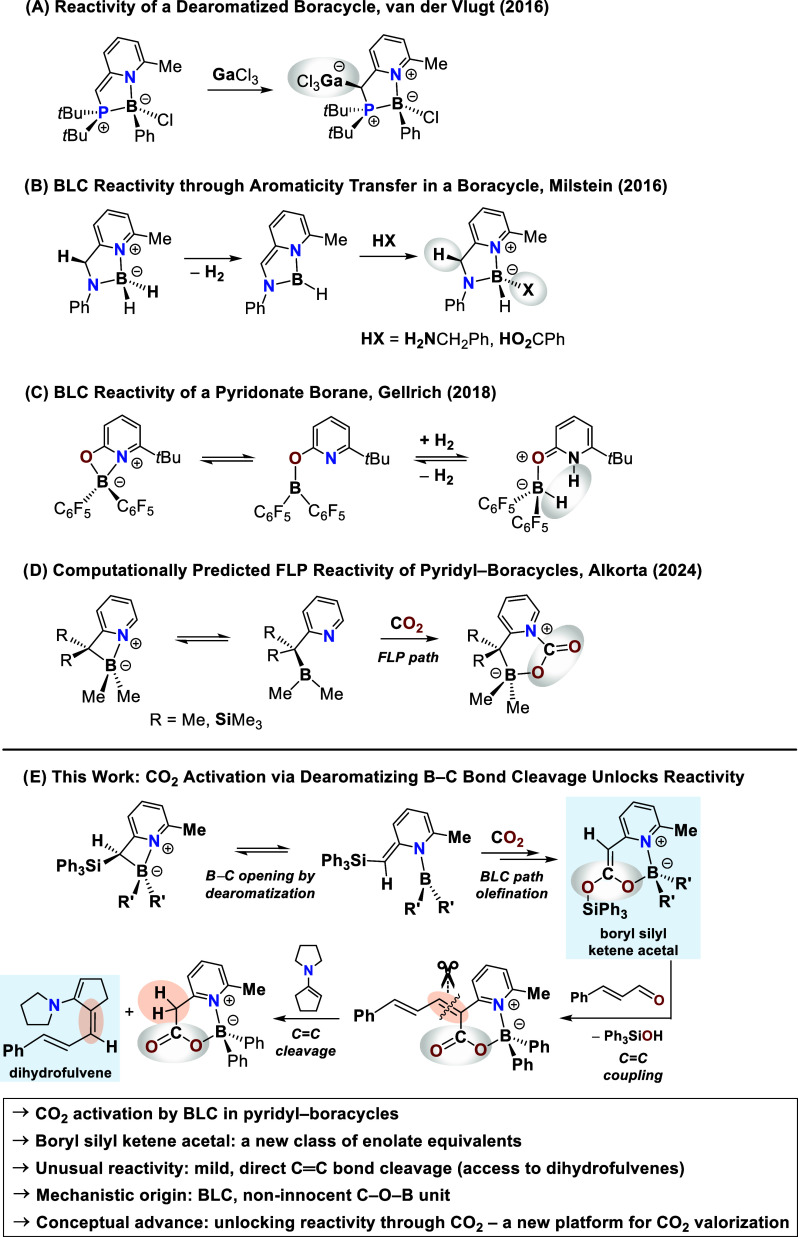
Conceptual development of boron–ligand cooperation (BLC): (A) van der Vlugt’s stabilized dearomatized boron–pyridyl complex;^26^ (B) Milstein’s cooperative boron–ligand system;^27^ (C) Gellrich’s reversible H_2_ activation with pyridonate boranes;^28^ (D) Alkorta’s theoretical prediction of a B/N-FLP pathway for CO_2_ activation;^57^ (E) this work: CO_2_ activation via dearomatizing B–C bond cleavage furnishes boryl silyl ketene acetals as a new class of enolate equivalents, unlocking exceptional reactivity, including mild and direct C=C double bond cleavage to dihydrofulvenes.

Here, we disclose the first experimental evidence that four-membered pyridyl–boracycles activate CO_2_ via a boron–ligand cooperation mechanism. In stark contrast to theoretical predictions of a B/N-FLP-type pathway (Figure 1,D),^57^ the activation proceeds unambiguously via a dearomatizing boron–carbon bond cleavage (Figure 1,E). Complementary density functional theory (DFT) studies reveal that the BLC pathway is strongly favored, both kinetically and thermodynamically, over any conceivable FLP-type mechanism. This unprecedented mode of CO_2_ fixation directly furnishes boryl silyl ketene acetals (which can also be viewed as boryl silyl ester enolates), a novel class of enolate equivalents that display a reactivity pattern far beyond that of classical enol or enolate chemistry. Remarkably, these intermediates not only undergo transformations such as Michael- and aldol-type reactions, but ultimately enable a mild, direct cleavage of a C=C double bond, providing straightforward access to fulvene derivatives.

Taken together, our findings illustrate how BLC drives the formation of unprecedented intermediates. In particular, they establish boryl silyl ketene acetals derived from CO_2_ as a novel class of main-group systems that unlock a previously inaccessible reactivity platform.

## Results and Discussion

### Evidence of Boron–Ligand Cooperation and Formation of Boryl Silyl Ketene Acetals from CO_2_

Building on the potential of small-ring systems^58^ and our previous contributions in this field,^59–62^ we have now extended our investigations to four-membered pyridine-based boracycles (Figure 1,E). The conjugated π-system in these scaffolds displays striking electronic parallels to the much-discussed bonding motifs in 2-picolyllithium^63–65^ and its 2-silylmethyl derivatives,^66–70^ to alkali-metal dihydropyridines,^71–75^ and to the pyridine backbone of pincer-type complexes.^12–18,25^ Reaction pathways in such boracycles, whether following a frustrated Lewis pair (FLP) or a boron–ligand cooperation (BLC) mechanism, have long been debated, and are known to be highly sensitive to the substitution pattern.^76–79^

The synthesis of the target pyridyl–boracycles commenced from 2-(triphenylsilyl)­lutidine (**1**) (for the X-ray structural analysis of **1**, see the Supporting Information, SI), which, after lithiation with *n*-butyllithium in tetrahydrofuran (THF), afforded the solvated enamide **2** as single crystals suitable for X-ray diffraction analysis (Scheme 1).

**Scheme 1:**
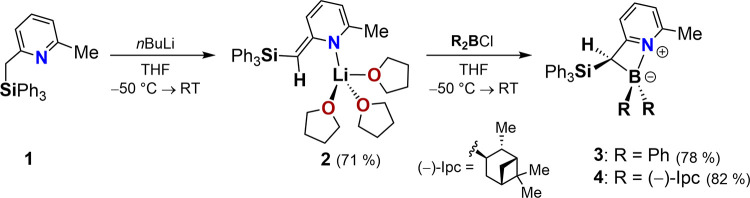
Synthesis of the Four-Membered Pyridyl–Boracycles 3 and 4 via the Fully Dearomatized Lithium Enamide 2. Ipc = (−)-***iso***-Pinocampheyl

The molecular structure of **2** clearly demonstrates the pronounced disruption of the aromatic π-system (Figure 2). In contrast to previously reported 2-picolyl alkali-metal complexes,^63–70^ the lithium cation exhibits no interaction with the aromatic π-system, neither in a carbanionic nor an azaallyl-type fashion. Instead, the coordination is exclusively through the enamide functionality, accompanied by shortened C19–C20 bond lengths [1.395 Å and 1.397 Å (**2**) versus 1.505 Å (**1**)], which are much closer to typical C­(sp^2^)=C­(sp^2^) double bonds.^80^ The alternating C–C bond lengths in the pyridine ring of **2** (values between 1.361 and 1.443 Å) further corroborate a loss of aromaticity (for comparison, the average C–C bond length in **1** is 1.383 Å). These features already point to a strong predisposition of the ligand framework toward the formation of dearomatized, highly reactive species.

**Figure 2:**
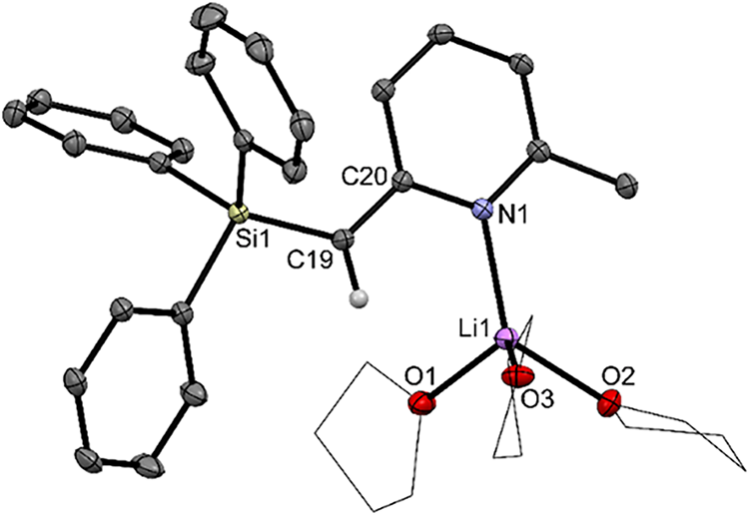
Molecular structure of compound **2** in the crystal (displacement ellipsoids at 50% probability). One molecule of the asymmetric unit is shown. Hydrogen atoms, except H at C19, are omitted for clarity.

Subsequent reaction of **2** with chlorodiphenyl- or chlorodi(−)-*iso*-pinocampheylborane directly afforded monomeric cyclic pyridyl boranes **3** and **4** (Scheme 1), both of which were structurally characterized by single-crystal X-ray diffraction analysis (Figure 3). Compounds **3** and **4** adopt almost perfectly planar four-membered ring geometries, and the boron atoms exhibit a pronounced tetrahedral character, with THCs of 80% (**3**) and 77% (**4**), respectively. The C19–C20 bond lengths of 1.499 Å (**3**) and 1.495 Å (**4**) are in the expected range of C­(sp^3^)–C­(aryl) single bonds^80^ [C19–C20 (**1**): 1.505 Å], while the C19–B1 distances are nearly identical across both species (1.705–1.709 Å). Notably, the dative B1–N1 bond in **4** (1.703 and 1.720 Å) is elongated compared to that in **3** (1.643 Å), a consequence of the steric demand imposed by the chiral (−)-*iso*-pinocampheyl substituents.

**Figure 3:**
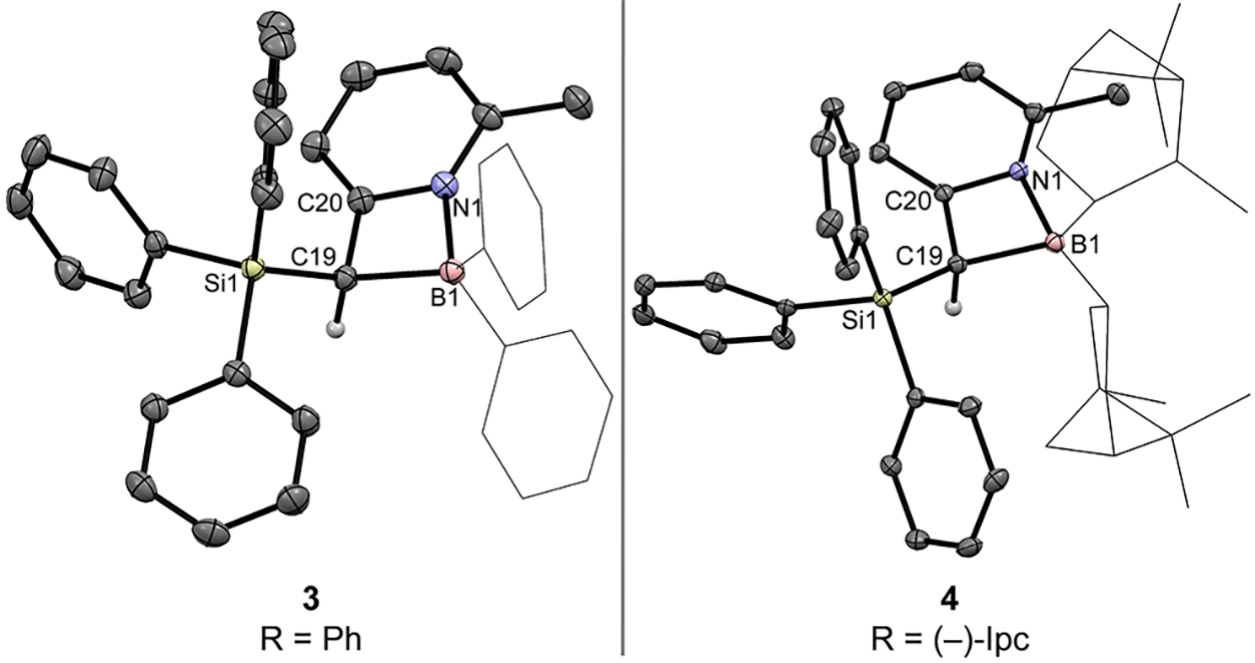
Molecular structures of the pyridyl–boracycles **3** and **4** in the crystal (displacement ellipsoids at 50% probability). One molecule of the asymmetric unit of **4** is shown. Solvent molecules (diethyl ether in **3**) and hydrogen atoms, except H at C19, are omitted for clarity.

At room temperature, neither **3** nor **4** showed any reactivity toward CO_2_ (3 bar). Upon heating to 90–120 °C in toluene, however, both underwent clean conversion to crystalline products **5** and **6** (Scheme 2), which were unequivocally identified by single-crystal X-ray diffraction analysis as unsaturated six-membered heterocycles (Figure 4). To the best of our knowledge, this transformation represents the first direct entry into boryl silyl ketene acetals, a previously unknown class of compounds. Mechanistically, their formation requires a three-step sequence: (i) dearomatizing cleavage of the B–C bond with formation of intermediates **3′** and **4′**, (ii) CO_2_ capture via boron–ligand cooperation, and (iii) an olefination of the α-silyl carboxyl intermediates **7** and **8** triggered by a [1,3]-silyl migration,^81,82^ which provides a substantial thermodynamic driving force for the process (Scheme 2).

**Figure 4:**
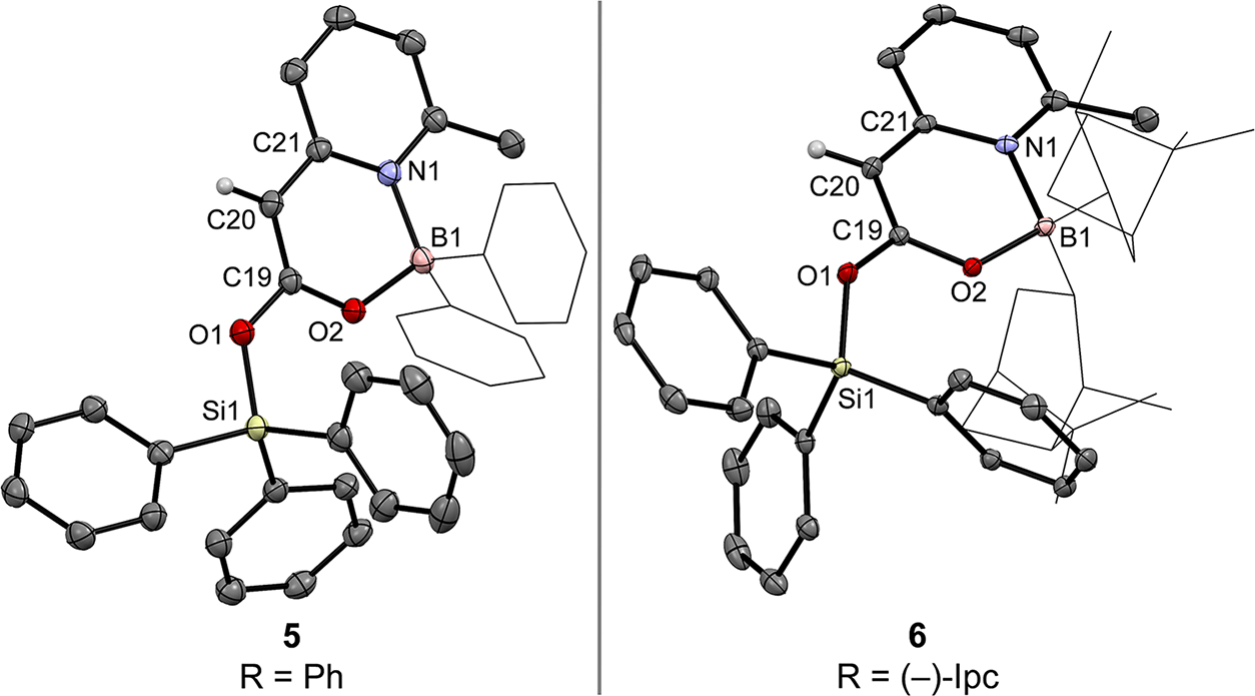
Molecular structures of the boryl silyl ketene acetals **5** and **6** in the crystal (displacement ellipsoids at 50% probability). Solvent molecules (benzene in **5**) and hydrogen atoms, except H at C20, are omitted for clarity.

**Scheme 2:**
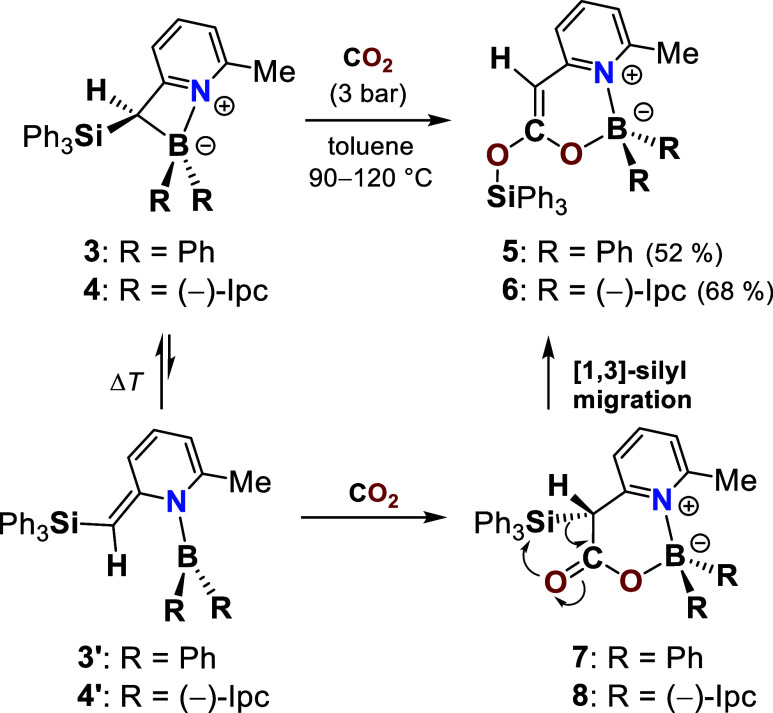
Reaction of Pyridyl–Boracycles 3 and 4 with CO_**2**_ to Give Boryl Silyl Ketene Acetals 5 and 6 via a BLC Mechanism Followed by an Immediate [1,3]-Silyl Migration

The structural parameters of **5** and **6** are fully consistent with their assignment as boryl silyl ketene acetals (Figure 4). Their C19–C20 bonds [1.353 Å (**5**), 1.356 Å (**6**)] clearly fall within the range of C­(sp^2^)=C­(sp^2^) double bonds of enol esters,^80^ corroborating the enolate-like character of these species. In both compounds, the C19–O2 bond [1.303 Å (**5**), 1.290 Å (**6**)] is shorter than the corresponding C19–O1 bond [1.333 Å (**5**), 1.337 Å (**6**)], providing clear structural evidence that O2 exerts the stronger +M effect.^83^ This differentiation can be rationalized by negative hyperconjugation of the O1 lone pairs with vicinal, antibonding σ*_(Si–C)_ orbitals,^84–86^ which stabilizes electron density at O1, whereas such an interaction is negligible for the tetracoordinate second-row element boron, rendering O2 the more effective electron-pair donor in enolate-type reactivity. Notably, the B1–N1 bond in **6** (1.679 Å) is again elongated relative to that in **3** (1.623 Å), highlighting the structural flexibility of the boracyclic framework and its capacity to adapt its bonding interactions in response to reaction demands.

### Mechanistic Investigations: BLC Versus B/N-FLP Pathway

To gain deeper insight into the reaction pathway, which our experimental data had already indicated to follow a boron–ligand cooperation (BLC) mechanism, we performed detailed DFT calculations at the M06-2X/6–311+G­(d,p) level of theory^87–91^ with solvation modeled by the polarizable continuum model (PCM) (solvent: toluene) (Figure 5).^92^ Previous theoretical work by Alkorta et al. had suggested an alternative FLP-type pathway, initiated by opening of the B–N bond in the boracycle and followed by exergonic adduct formation between the boron/nitrogen Lewis pair and CO_2_.^57^ We therefore examined both scenarios (BLC and FLP) in parallel.

**Figure 5:**
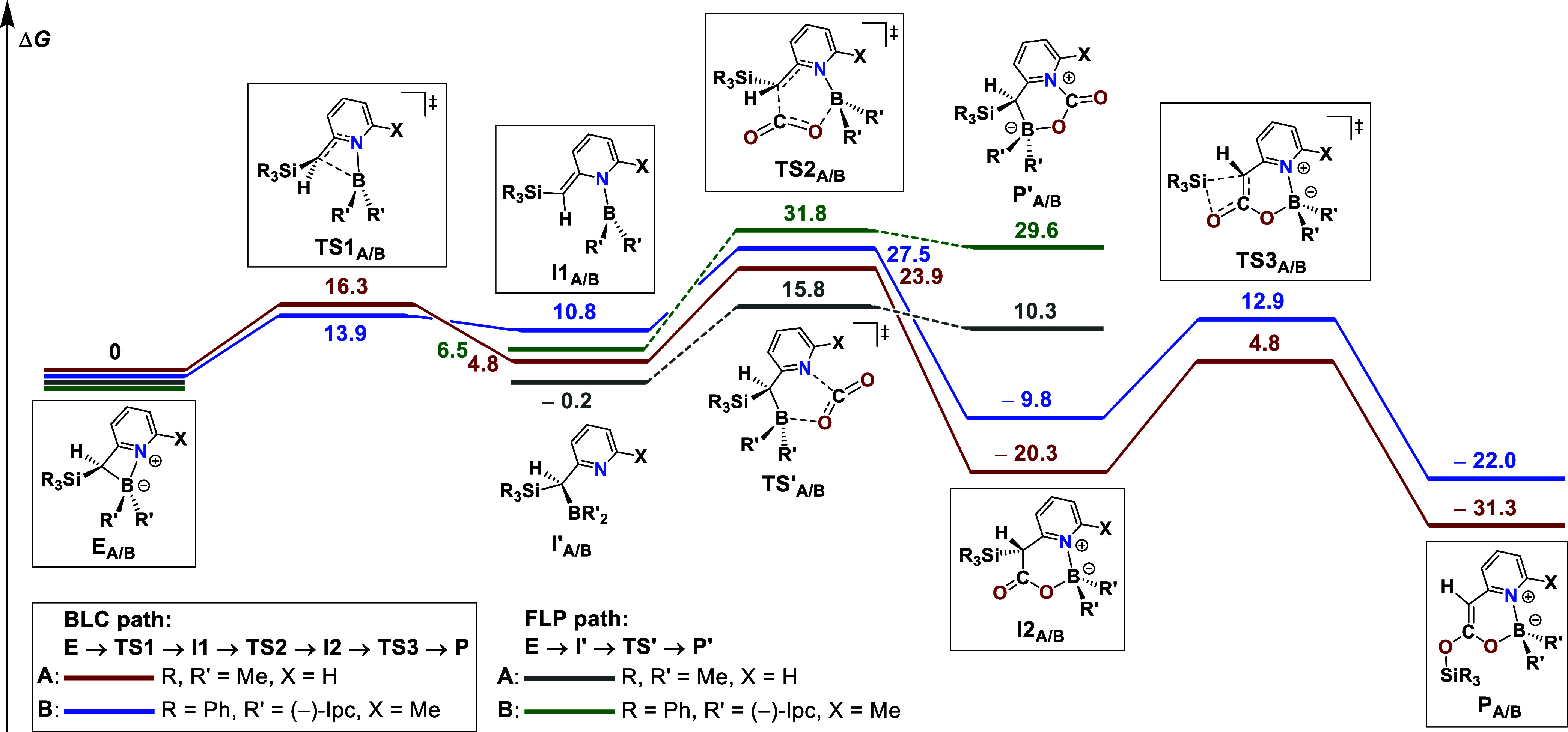
Computed mechanism of CO_2_ activation at the M06-2X/6–311+G­(d,p) level of theory (PCM, toluene).^87–92^ Comparison of the BLC pathway (red/blue) with the B/N-FLP pathway (gray/green). Gibbs energies (Δ*G*) in kcal mol^–1^.

Calculations were carried out for a simplified 2-picolylborane model system (**A**: R, R′ = Me, X = H) as well as for the experimentally studied 2-lutidylborane (**B**: R = Ph, R′ = (−)-Ipc, and X = Me). In the BLC pathway (red and blue traces, Figure 5), CO_2_ activation is initiated by dearomatizing cleavage of the B–C bond, giving the open enamide **I1** via transition state **TS1**. The activation barriers (ΔΔ*G* ^‡^) are modest and comparable for both systems (**TS1**_**A**_: 16.3 kcal mol^–1^; **TS1**_**B**_: 13.9 kcal mol^–1^). However, the relative stabilities of the dearomatized intermediates **I1** depend sensitively on the substitution pattern: effective n_N_ → p_B_ conjugation lowers the energy of **I1**_**A**_ (4.8 kcal mol^–1^), whereas steric congestion from the bulky (−)-*iso*-pinocampheyl substituents destabilizes **I1**_**B**_ (10.8 kcal mol^–1^).

Subsequent attack of CO_2_ at open boryl enamide **I1** proceeds through **TS2**_**A**_ (ΔΔ*G* ^‡^ = 19.1 kcal mol^–1^) or **TS2**_**B**_ (ΔΔ*G* ^‡^ = 16.7 kcal mol^–1^), forming a six-membered cyclic boryl ester intermediate (**I2**). Although **I2** is only transient, its formation is highly exergonic (ΔΔ*G* = −25.1 kcal mol^–1^ for **A**; ΔΔ*G* = −20.6 kcal mol^–1^ for **B**), driven by the restoration of aromaticity. The overall process gains its large thermodynamic driving force (**P**_**A**_: Δ*G* = −31.3 kcal mol^–1^; **P**_**B**_: Δ*G* = −22.0 kcal mol^–1^) from the final, rate-determining olefination (**I2**_**A/B**_ → **P**_**A/B**_). This step involves a concerted [1,3]-silyl migration proceeding through a four-membered cyclic transition state (**TS3**_**A**_: ΔΔ*G* ^‡^ = 25.1 kcal mol^–1^; **TS3**_**B**_: ΔΔ*G* ^‡^ = 22.7 kcal mol^–1^), thereby confirming Kira’s findings,^93^ which is driven by Si–O bond formation and accompanied by the generation of a synthetically valuable, electron-rich ketene-like C=C double bond (Figure 5). Notably, examples of C–C bond formation with CO_2_ in boron/carbon FLP systems are rare, emphasizing the distinctive reactivity of the present BLC system.^34,35,51^

In stark contrast, the alternative FLP pathway (gray and green traces; Figure 5) yields no feasible solution. According to our calculations, formation of a CO_2_ adduct from a putative frustrated B/N Lewis pair (**I′**_**A/B**_ → **P′**_**A/B**_) is strongly endergonic (ΔΔ*G* = 23.1 and 10.5 kcal mol^–1^ for **A** and **B**, respectively) and does not produce a thermodynamically stable species. The transition states (**TS′**_**A**_: ΔΔ*G* ^‡^ = 16.0 kcal mol^–1^; **TS′**_**B**_: ΔΔ*G* ^‡^ = 25.3 kcal mol^–1^) are even higher in energy or at least in a similar range than the corresponding BLC transition states, rendering the FLP scenario highly unlikely (computational results for the bis-silyl-substituted model system are provided in Figure S84 of the SI).

Overall, these computational results are in full agreement with the experiment and demonstrate that CO_2_ activation in pyridyl–boracycles proceeds unequivocally via a BLC mechanism. Importantly, this holds true irrespective of the specific substituents present at the 6-position of the pyridine ring or within the silyl and boryl groups, indicating the generality and robustness of this cooperative activation mode.

### Follow-Up Reactions (Michael and Aldol Reactions) of the Boryl Silyl Ketene Acetals

The products of CO_2_ activation constitute a previously unknown class of enolate equivalents, distinguished by a unique bifunctional motif in which one oxygen atom is bound to a silyl group and the other to a boryl substituent. Owing to the embedding of the electron-rich olefinic unit within a rigid cyclic framework, we next probed their dienophilicity. As electron-deficient reaction partners for a potential inverse-electron-demand hetero-Diels–Alder (iEDHDA) reaction,^94–99^ acrolein and cinnamaldehyde were selected (Schemes 3 and 4). Reaction with acrolein (Scheme 3) did not proceed via a concerted [4 + 2] cycloaddition but instead followed a stepwise pathway. The initial Michael-type 1,4-addition is facilitated by the C–O–B fragment, which acts as a noninnocent cooperative motif, with its pronounced +M effect playing a decisive role.^83^ The resulting zwitterionic intermediate **10-i** is stabilized through proton abstraction and subsequent keto–enol tautomerization (Scheme 3, bottom). Steric constraints render a ring closure via intramolecular attack of the alkoxide at the activated carboxyl carbon atom of **10-i** implausible. Remarkably, the transformation does not follow a Mukaiyama–Michael pathway either;^100–103^ instead, the ketene acetal functionality in **9** remains entirely intact.

**Scheme 3:**
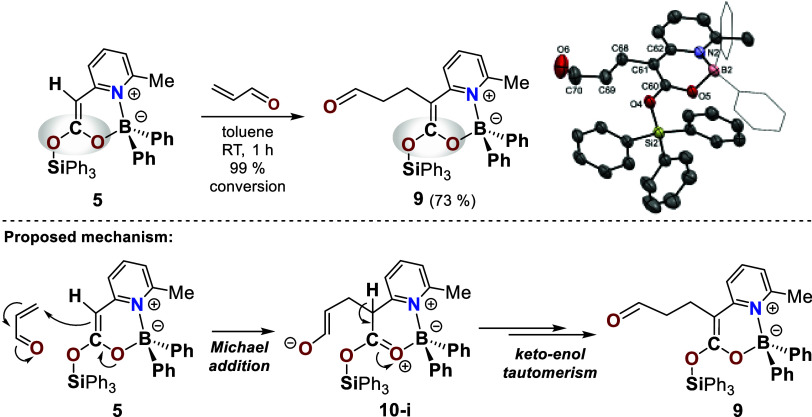
Reaction of Boryl Silyl Ketene Acetal 5 with Acrolein to Give Product 9: Michael-Type Addition Facilitated by the C–O–B Fragment^a^

a Molecular structure of compound **9** in the crystal (displacement ellipsoids at 50% probability). One molecule of the asymmetric unit is shown. Hydrogen atoms are omitted for clarity.

**Scheme 4:**
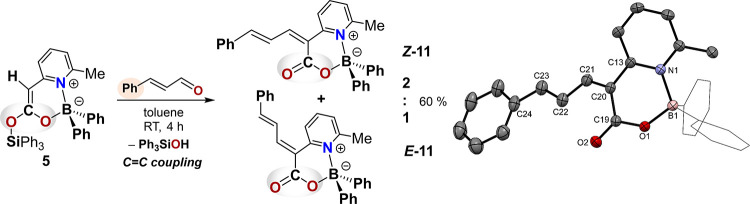
Reaction of Boryl Silyl Ketene Acetal 5 with Cinnamaldehyde: Mukaiyama-Type Aldol Addition Followed by Condensation to the Diastereomers ***Z***-11 and ***E***-11^a^

a Molecular structure of boryl ester ***Z*-11** in the crystal (displacement ellipsoids at 50% probability). Hydrogen atoms are omitted for clarity.

In sharp contrast, the reaction of **5** with cinnamaldehyde follows a different trajectory (Scheme 4). Here, a C=C coupling reaction occurs that can be interpreted as a Lewis-acid-free, Mukaiyama-type aldol addition^104–106^ followed by condensation under mild conditions, affording the two diastereomers ***Z*** **-11** and ***E*** **-11** in a 2:1 ratio. We attribute this unusually mild, additive-free reactivity to the unique dual character of our boryl silyl ketene acetals, which merge key features of both boron and silicon enolates.^107–109^ In contrast to classical Lewis-acid-promoted Mukaiyama aldol reactions, which are generally assumed to proceed via an open transition state,^110–112^ the present addition most plausibly follows a closed, Zimmerman–Traxler-type chairlike transition state,^113^ in which a pentacoordinate silicon atom constitutes an integral part of the cyclic transition-state framework (for mechanistic details supported by DFT calculations, see Figure S85 in the SI). Subsequent elimination of Ph_3_SiOH likely occurs from the enol form via a hydrogen-bonded transition state. The observed *Z*:*E* ratio can be rationalized by differences in the activation barriers of the two diastereomeric transition states, arising after rotation around the C­(α)–C­(β) bond in the enolic intermediate (see Section S5 in the SI).

### Mild, Direct C=C Bond Cleavage Enabling Access to Fulvene Derivatives

The selective scission of C=C double bonds is of fundamental importance in synthetic organic chemistry.^114,115^ In addition to olefin metathesis,^116,117^ the oxidative cleavage of alkenes into valuable carbonyl compounds represents one of the most widely applied and most actively explored transformations.^118–125^ Against this backdrop, the discovery of a mild approach to C=C bond cleavage, providing direct access to previously inaccessible classes of compounds, is highly significant.

Given that the α,β,γ,δ-unsaturated boryl esters ***Z*** **-11** and ***E*** **-11** possess an electron-deficient diene framework, we next explored their reactivity in the context of a hetero-Diels–Alder reaction with inverse electron demand, employing an enamine as an electron-rich dienophile. Strikingly, rather than proceeding through an iEDHDA pathway, the reaction follows an entirely unanticipated course, resulting in novel, mild, and direct C=C bond cleavage (Scheme 5).

**Scheme 5:**
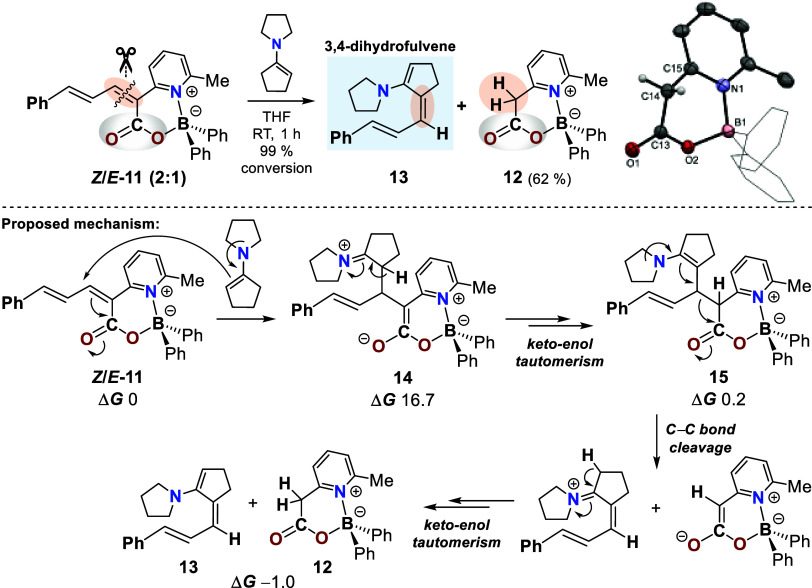
C=C Bond Cleavage of ***Z***/***E***-11 (2:1) (Only the ***Z*** Isomer is Shown) with 1-(1-Cyclopent-1-enyl)­pyrrolidine: Formation of Reduced Boryl Ester 12 and 3,4-Dihydrofulvene 13^a^

a Molecular structure of compound **12** in the crystal (displacement ellipsoids at 50% probability). Hydrogen atoms, except H at C14, are omitted for clarity. Gibbs energies (Δ*G*, in kcal mol^**–1**^) for key species along the reaction pathway were computed at the M06-2X/6–311+G­(d,p) level of theory.

Treatment of the ***Z***/***E*** **-11** isomer mixture (2:1) with 1-(1-cyclopent-1-enyl)­pyrrolidine at room temperature resulted in complete conversion (99%) and afforded reduced cyclic boryl ester **12** in 62% isolated yield, which was fully characterized by single-crystal X-ray diffraction analysis. Retrosynthetic analysis a priori suggested the concomitant formation of a 3,4-dihydrofulvene (**13**) (Scheme 5, bottom), which was indeed confirmed by NMR spectroscopy and high-resolution mass spectrometry (HRMS), even though isolation proved unfeasible owing to its pronounced tendency toward polymerization.

The mechanistic sequence begins with the formation of the zwitterionic iminium intermediate **14**, which undergoes proton elimination followed by keto–enol tautomerization rather than ring closure. The resulting enamine intermediate **15** promotes C–C bond cleavage, and subsequent proton transfer reactions furnish products **12** and **13**. In this process, a new C=C bond is formed as the exocyclic double bond of 3,4-dihydrofulvene, bearing an electron-donating substituent at its 1-position. Accordingly, this transformation can be regarded as a new type of metathesis reaction.

Moreover, the facile recovery of compound **5**, directly accessible from compound **12**, underlines its potential as a sustainable auxiliary in preparative chemistry (for details, see Section S3 in the SI).

In sum, these findings establish cyclopentenylamines as hitherto unknown cleavage reagents that enable direct access to fulvene derivatives, a synthetically valuable class of compounds with a uniquely rich reactivity profile, whose preparation has always been challenging.^126–130^ This discovery not only expands the scope of boron–ligand cooperation chemistry but also highlights a conceptually new strategy for selective C=C bond scission under remarkably mild conditions. Ongoing studies are directed toward probing the synthetic potential and extending this methodology to diverse substitution patterns.

## Conclusions

This work establishes boryl silyl ketene acetals derived from CO_2_ as a previously unexplored class of main-group intermediates with unique reactivity. We provide the first experimental evidence that pyridyl–boracycles activate CO_2_ through a boron–ligand cooperation (BLC) mechanism rather than via a B/N-FLP-type pathway. Crucially, CO_2_ is not merely sequestered but initiates a reaction cascade, leading to boryl silyl ketene acetals. These intermediates display a strikingly broad spectrum of unforeseen follow-up reactivity, including Michael- and aldol-type reactions, and ultimately leading to a mild, one-step cleavage of C=C double bonds that affords fulvene derivatives under additive-free conditions. Collectively, CO_2_ capture by pyridyl–boracycles enables entirely new reactivity patterns and synthetic strategies far beyond classical enolate or enol chemistry, thereby opening new conceptual and synthetic perspectives for main-group element chemistry. All transformations of the boryl silyl ketene acetals proceed under mild conditions, in the absence of additives, and with high chemoselectivity, which are features that underscore the robustness and versatility of this platform. We attribute this behavior to the unusual combination of a rigid yet electronically adaptive cyclic framework with the cooperative interplay between the keto and enolate forms of the C–O–B functionality.

## Supplementary Material



## Notes

Synthetic procedures; NMR spectra of all isolated compounds; and detailed information on X-ray crystallography and quantum chemical calculations (PDF)
