# Supplementary material for: CO2 Unlocks Reactivity: Boryl Silyl Ketene Acetals Enable Mild and Direct C=C Bond Cleavage
Source: J Am Chem Soc. 2025 Dec 15;147(51):47573–83. doi: 10.1021/jacs.5c16770 (PMC12752474; doi:10.1021/jacs.5c16770)
Supplement: Supplementary file 1 [file ja5c16770_si_001.pdf]

Supporting Information

CO_2 Unlocks Reactivity: Boryl Silyl Ketene Acetals Enable Mild and Direct C═C Bond Cleavage

Noel Angel Espinosa-Jalapa, Manuel Kümper, and Jonathan O. Bauer*

Faculty of Chemistry and Pharmacy, Institute of Inorganic Chemistry, University of Regensburg, Universitätsstraße 31, D-93053 Regensburg, Germany

Corresponding author: jonathan.bauer@ur.de

Table of contents

1. General Remarks 2

2. Synthetic Procedures 3

2.1. Synthesis of Compound **1** 3

2.2. Synthesis of Compound **2** 5

2.3. Synthesis of Compound **3** 8

2.4. Synthesis of Compound **4** 11

2.5. Synthesis of Compound **5** 17

2.6. Synthesis of Compound **6** 23

2.7. Synthesis of Compound **9** 30

2.8. Synthesis of Compounds ***Z*/*E*-11** 35

2.9. Synthesis of Compounds **12** and **13** 40

3. Recovery of Compound **5** 46

4. Single-Crystal X-Ray Diffraction Analysis 52

5. Density Functional Theory (DFT) Calculations 66

6. References 191

1. General Remarks

All experiments were performed in an inert atmosphere of purified nitrogen by using standard Schlenk techniques or an MBraun Unilab 1200/780 glovebox. Glassware was heated at 200 °C prior to use. Diethyl ether (Et_2O), dichloromethane (DCM), *n*-hexane, *n*-pentane, tetrahydrofuran (THF), and toluene were dried and degassed with an MBraun SP800 solvent purification system. Chlorotriphenylsilane (96%, Aldrich), *n*-butyllithium (2.5 M, in hexane, Aldrich), methyllithium (1.6 M, in diethyl ether, Aldrich), and chlorodi-(–)-*iso*-pinocampheylborane [(–)-Ipc_2BCl, Aldrich) were used as received. Prior to use, cinnamaldehyde (≥95%, Aldrich) and acrolein (90%, Aldrich) were stored within a glovebox under dry molecular sieves (3Å) at room temperature and ~0 °C, respectively. Chlorodiphenylborane,[131] 2,6-lutidyllithium,[132] and 1-(1-cyclopent-1-enyl)pyrrolidine[133,134] were prepared following reported procedures. NMR spectra were recorded in benzene-d_6 (C_6D_6, ≥ 99.6%, Merck, dried over Na/K amalgam), toluene-d_8 (Tol-d_8, ≥ 99.6%, Merck, dried over Na/K amalgam), dichloromethane-d_2 [CD_2Cl_2, > 99.8%, Fluorochem, dried over molecular sieves (3 Å)], or tetrahydrofuran-d_8 (THF-d_8, ≥ 99.5%, Merck, dried over Na/K amalgam). NMR spectra were either recorded on a Bruker Avance 400 (400.13 MHz), a Bruker Avance III HD 400 (400.13 MHz), or a Bruker Avance Neo (500.18 MHz) at the reported temperature. Chemical shifts (δ) are reported in parts per million (ppm). ^{1}H and $^{13}C\{^{1}H\}$ NMR spectra are referenced to tetramethylsilane ($SiMe_4$, δ = 0.0 ppm) as external standard, with the deuterium signal of the solvent serving as internal lock and the residual solvent signal as an additional reference. $^{29}Si\{^{1}H\}$ NMR spectra are referenced to $SiMe_4$ (δ = 0.0 ppm), $^{7}Li\{^{1}H\}$ NMR spectra to 1 M solution of LiCl in D_2O (δ = 0.0 ppm) and $^{11}B\{^{1}H\}$ NMR spectra to $Et_2O{\bullet}BF_3$ (δ = 0.0 ppm) as external standard. For the assignment of the multiplicities, the following abbreviations were used: b, broad; s, singlet; d, doublet; t, triplet; q, quartet; quint, quintet; m, multiplet. Elemental analyses were performed on a Vario MICRO cube apparatus. High-resolution mass spectrometry was carried out on a Jeol AccuTOF GCX and an Agilent Q-TOF 6540 UHD spectrometer.

2. Synthetic Procedures

2.1. Synthesis of Compound 1

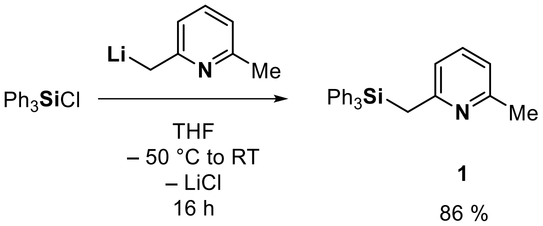

To a Schlenk flask charged with Chlorotriphenylsilane (4.42 g, 15.0 mmol, 1.0 equiv.) in THF (80 mL) at –50 °C, a solution of 2,6-lutidyllithium (1.70 g, 15.0 mmol, 1.0 equiv.) in THF (40 mL) was added dropwise via PTFE cannula under stirring. The glassware used for the preparation of the 2,6-lutidyllithium solution was rinsed with THF (1 × 5 mL), and the washing was transferred to the reaction mixture. The resulting pale orange suspension was allowed to warm to room temperature and stirred overnight. The reaction mixture was subsequently dried under vacuum, and the residue was suspended in dichloromethane (20 mL). The suspension was transferred via PTFE cannula to a fritted column packed with Celite®, filtered, and the solids were washed with dichloromethane (3 × 20 mL). Removal of all volatiles from the combined beige filtrates under reduced pressure afforded a thick oily residue. Addition of hexane (~60 mL) and stirring at room temperature for 30 min resulted in a beige suspension. Filtration by cannula suction yielded a pale beige powder of spectroscopically pure compound **1**. The combined clear filtrates were stored at –30 °C to give, over the course of three days, a second crop of **1** as beige crystalline material suitable for single-crystal X-ray diffraction analysis. The combined batches afforded compound **1** in 86 % yield (4.73 g, 12.96 mmol).

^{1}H NMR (400.13 MHz, DCM-d_2, 25° C): δ 2.38 (s, 3H, CH_3), 3.21 (s, 2H, SiCH_2), 6.61 (d, $^{3}J_{HH}$ = 7.6 Hz, 1H, $H_{m\text{-Py}}$), 6.82 (d, $^{3}J_{HH}$ = 7.6, 1H, $H_{m\text{-Py}}$), 7.24 (t, $^{3}J_{HH}$ = 7.6 Hz, 1H, $H_{p\text{-Py}}$), 7.32 – 7.38 (m, 6H, H_{Ph}), 7.39 – 7.45 (m, 3H, H_{Ph}), 7.50 – 7.54 (m, 6H, H_{Ph}). **^{13}C{^{1}H} NMR** (100.62 MHz, DCM-d_2, 25°C): δ 24.0 (s, CH$_3$), 26.6 (s, SiCH$_2$), 118.9 (s, $C_{m\text{-Py}}$), 120.0 (s, $C_{m\text{-Py}}$), 127.7 (s, C_{Ph}), 129.5 (s, C_{Ph}), 134.5 (s, C_{Ph}), 135.8 (s, $C_{p\text{-Py}}$), 136.0 (s, C_{Ph}), 157.4 (s, $C_{o\text{-Py}}$), 158.6 (s, $C_{o\text{-Py}}$). **^{29}Si{^{1}H} NMR** (79.49 MHz, DCM-d_2, 25 °C): δ – 12.5 (s). **HRMS (EI^{+})**, calcd. m/z for [M^{+}]: 364.15160; found 364.15175. **Elemental analysis**: calcd. for $C_{25}H_{23}NSi$; C, 82.14; H, 6.34; N, 3.83; found: C, 82.12; H, 6.31; N, 3.68.

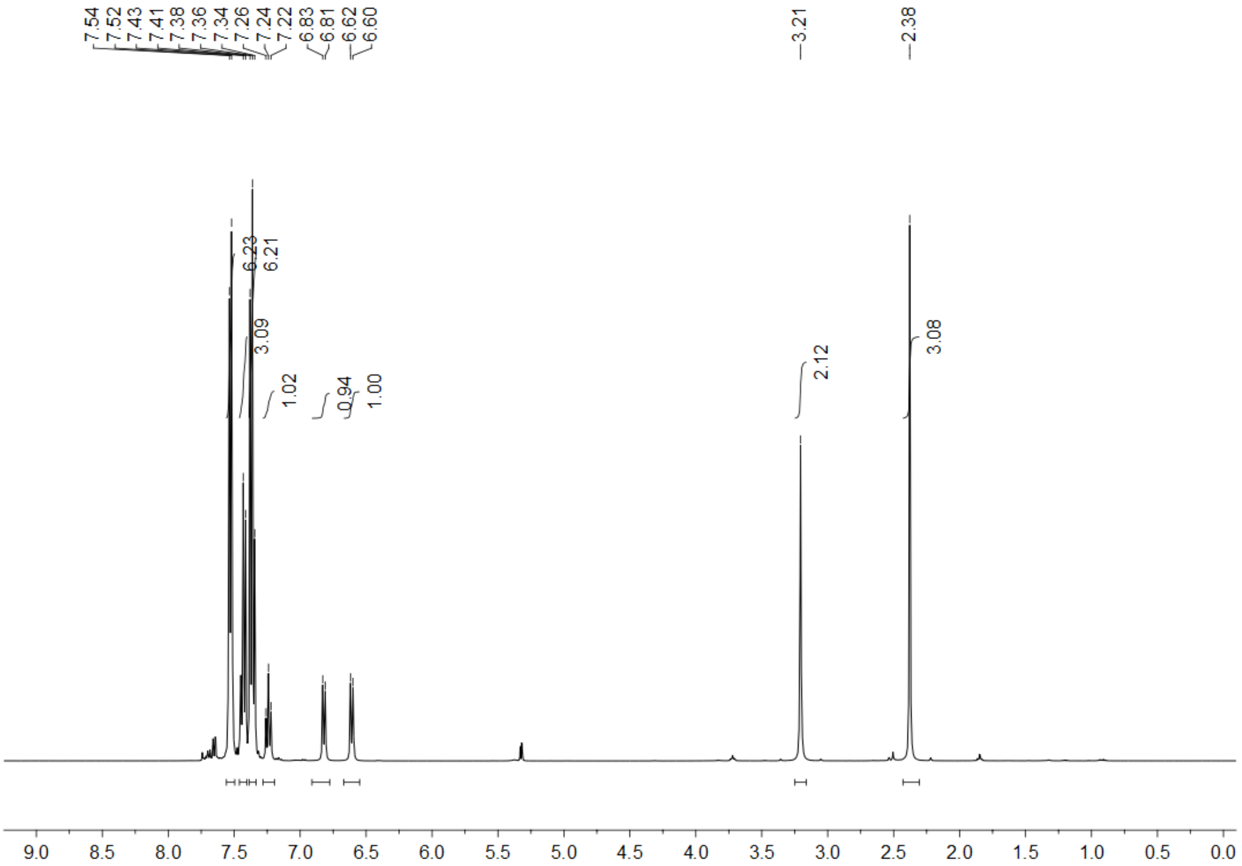

Figure S1. ^{1}H NMR spectrum (CD_2Cl_2, 298 K) of compound **1**.

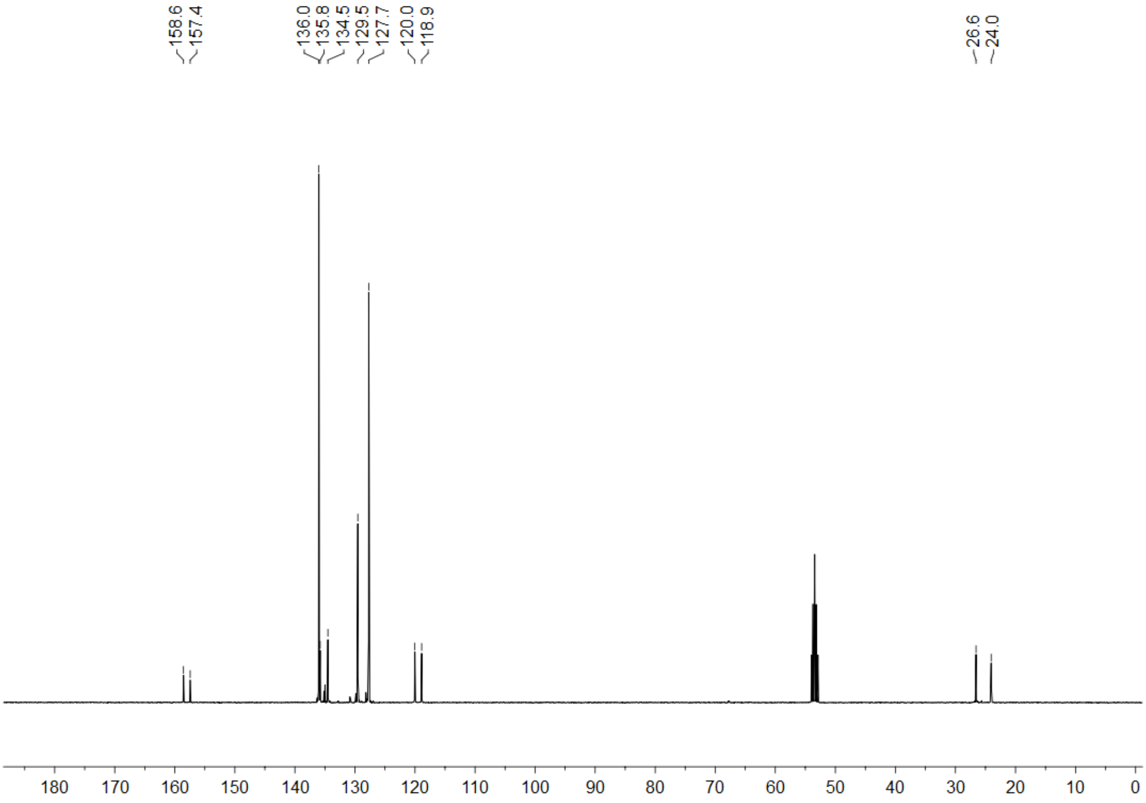

Figure S2. ^{13}C NMR spectrum (CD_2Cl_2, 298 K) of compound **1**.

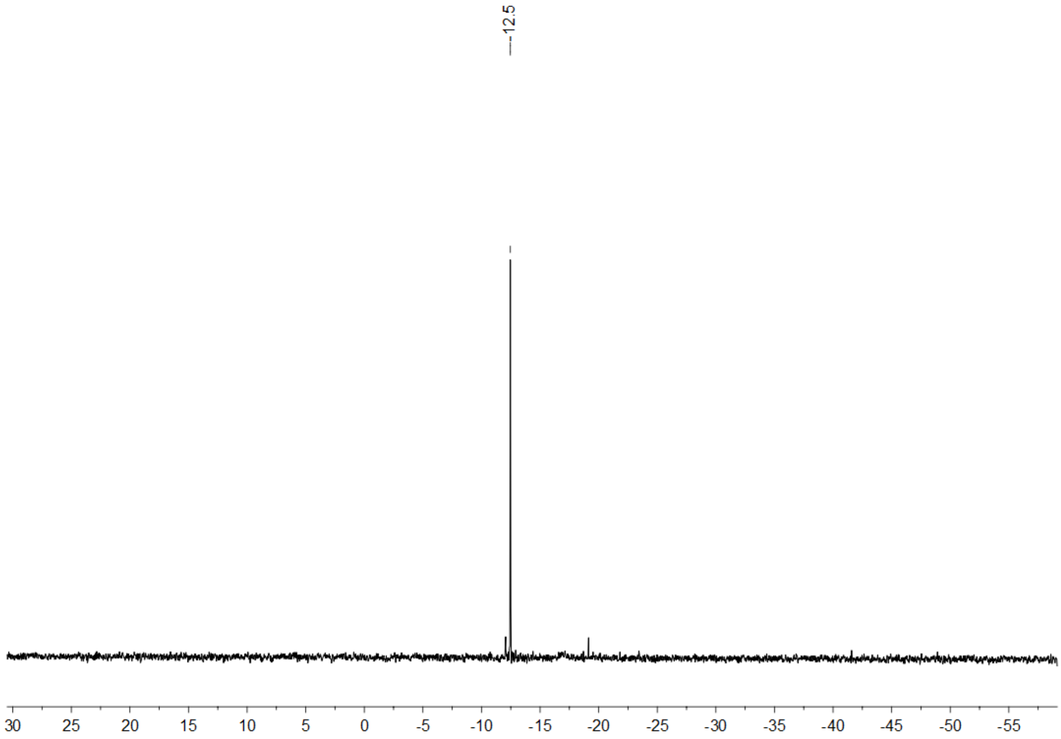

Figure S3. ^{29}Si{^{1}H} NMR spectrum (CD_2Cl_2, 298 K) of compound **1**.

2.2. Synthesis of Compound 2

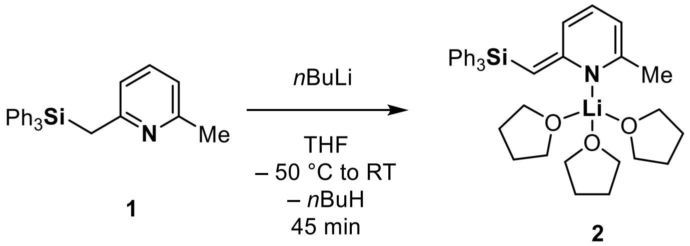

n-Butyllithium (4.0 mL of a 2.5 M solution in hexanes, 10.00 mmol, 1.02 equiv.) was added dropwise to a Schlenk flask containing compound **1** (3.58 g, 9.79 mmol, 1.0 equiv.) in THF (30 mL) at –50 °C under vigorous stirring. Immediate precipitation and an orange coloration were observed. Upon warming the mixture slowly to room temperature, the precipitate dissolved, yielding a clear orange solution. The reaction was stirred for a total of 45 min. The mixture was then concentrated to ca. 6 mL under reduced pressure, and the residue layered with hexane (~60 mL). The biphasic mixture was sealed and stored at –30 °C for three days, affording pure compound **2** as orange needle-shaped crystals suitable for single-crystal X-ray diffraction analysis. The mother liquor was removed, and the solid was dried in vacuo to give spectroscopically pure compound **2** as a yellow microcrystalline material (4.03 g, 71 %).

^{1}H NMR (400.13 MHz, THF-d_8, 25°C): δ 1.72 (m, H_{THF}), 1.87 (s, 3H, CH_3), 2.62 (s, 1H, SiC*H*), 3.58 (m, H_{THF}), 5.15 (d, $^{3}J_{HH}$ = 6.6 Hz, 1H, $H_{m\text{-}Py}$), 5.81 (d, $^{3}J_{HH}$ = 8.6 Hz, 1H, $H_{m\text{-}Py}$), 6.12 (dd, $^{3}J_{HH}$ = 8.6 Hz, $^{3}J_{HH}$ = 6.6 Hz, 1H, $H_{p\text{-}Py}$), 7.12 (m, 9H, H_{Ph}), 7.57 (m, 6H, H_{Ph}).**^{13}C{^{1}H} NMR** (100.62 MHz, THF-d_8, 25°C): δ 23.5 (s, CH_3), 25.4 (s, C_{THF}), 53.6 (s, Si*C*H), 67.3 (s, C_{THF}), 99.4 (s, $C_{m\text{-}Py}$), 113.6 (s, $C_{m\text{-}Py}$), 126.6 (s, C_{Ph}), 127.2 (s, C_{Ph}), 131.7 (s, $C_{p\text{-}Py}$), 135.8

(s, C_{Ph}), 141.6 (s, Si$C_{i\text{-}Ph}$), 154.2 (s, $C_{o\text{-}Py}$), 168.3 (s, $C_{o\text{-}Py}$). **^{29}Si{^{1}H} NMR** (79.49 MHz, THF-d_8, 25 °C): δ – 19.7 (s). **^{7}Li{^{1}H} NMR** (155.50 MHz, THF-d_8, 25 °C): δ 0.3 (s).

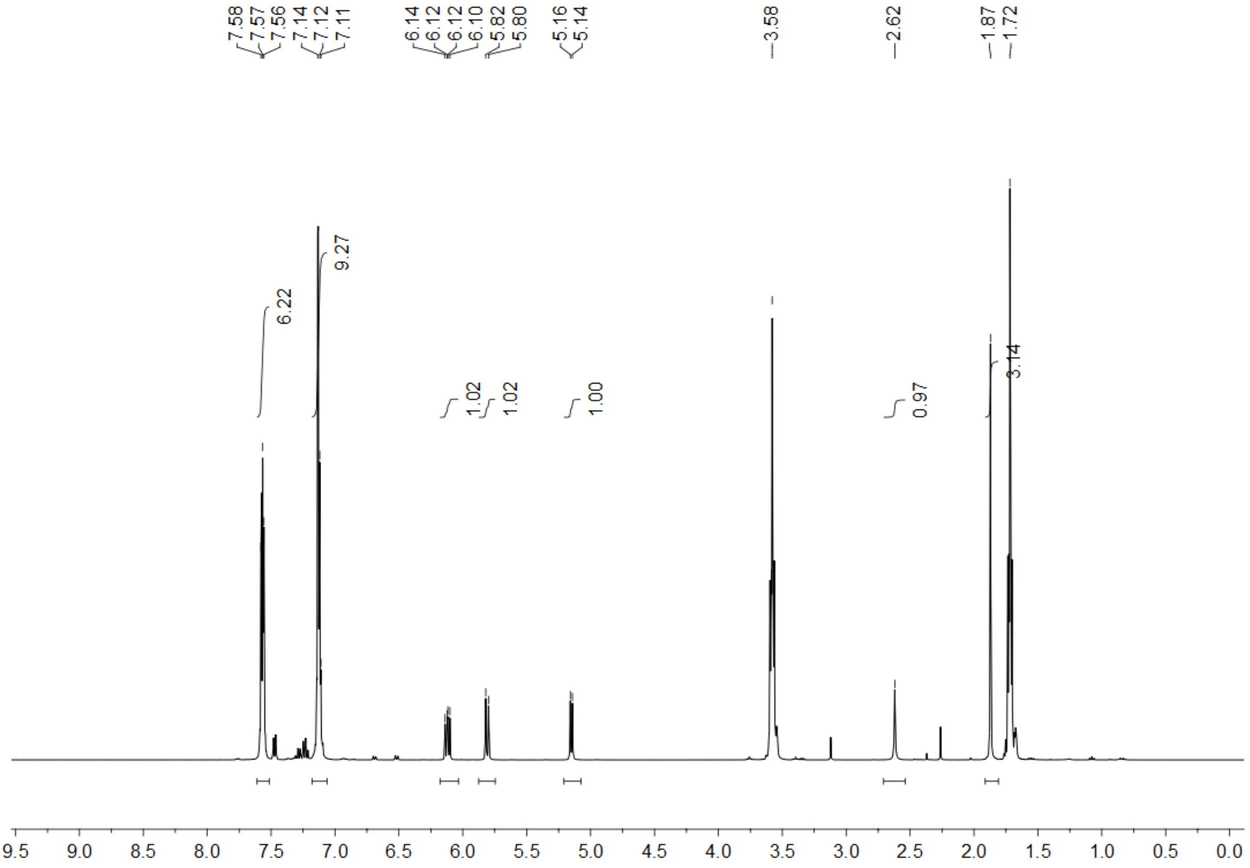

Figure S4. ^{1}H NMR spectrum (THF-d_8, 298 K) of compound **2**.

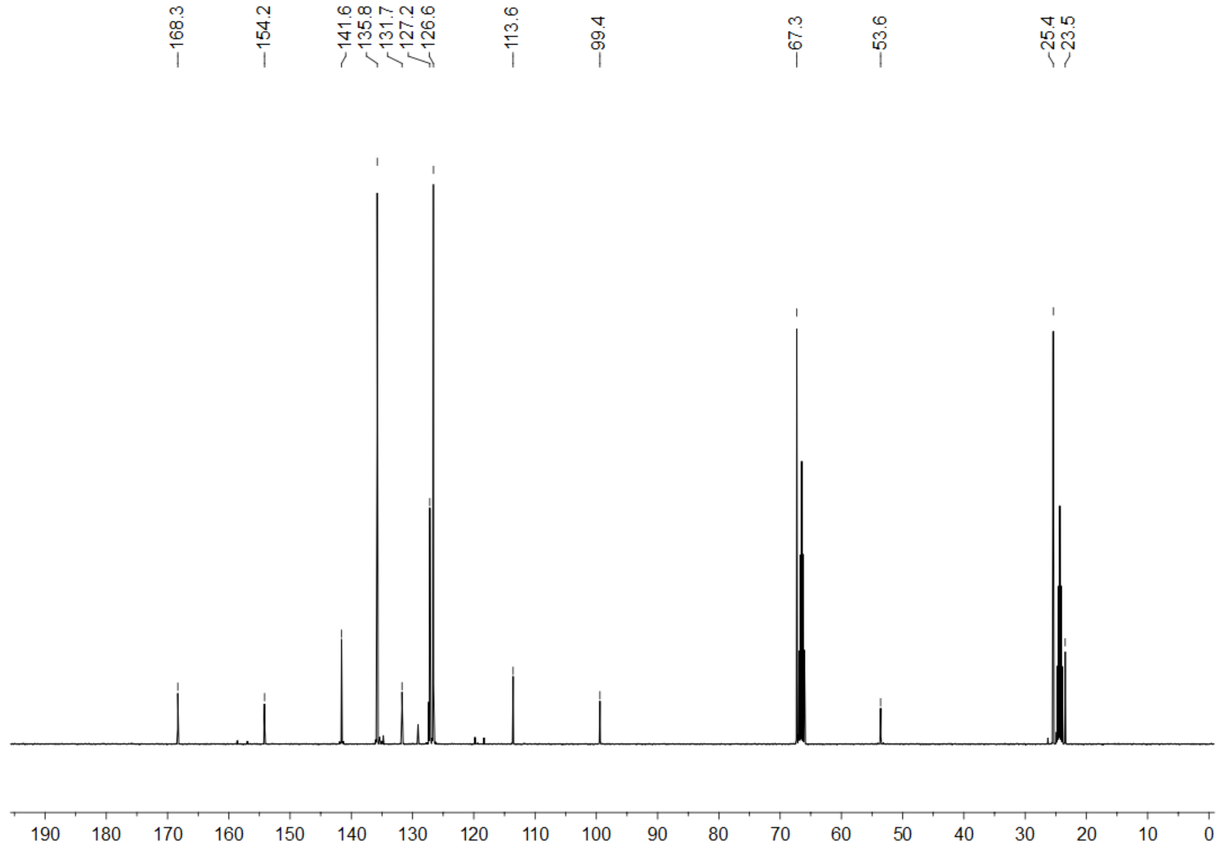

Figure S5. ^{13}C NMR spectrum (THF-d_8, 298 K) of compound **2**.

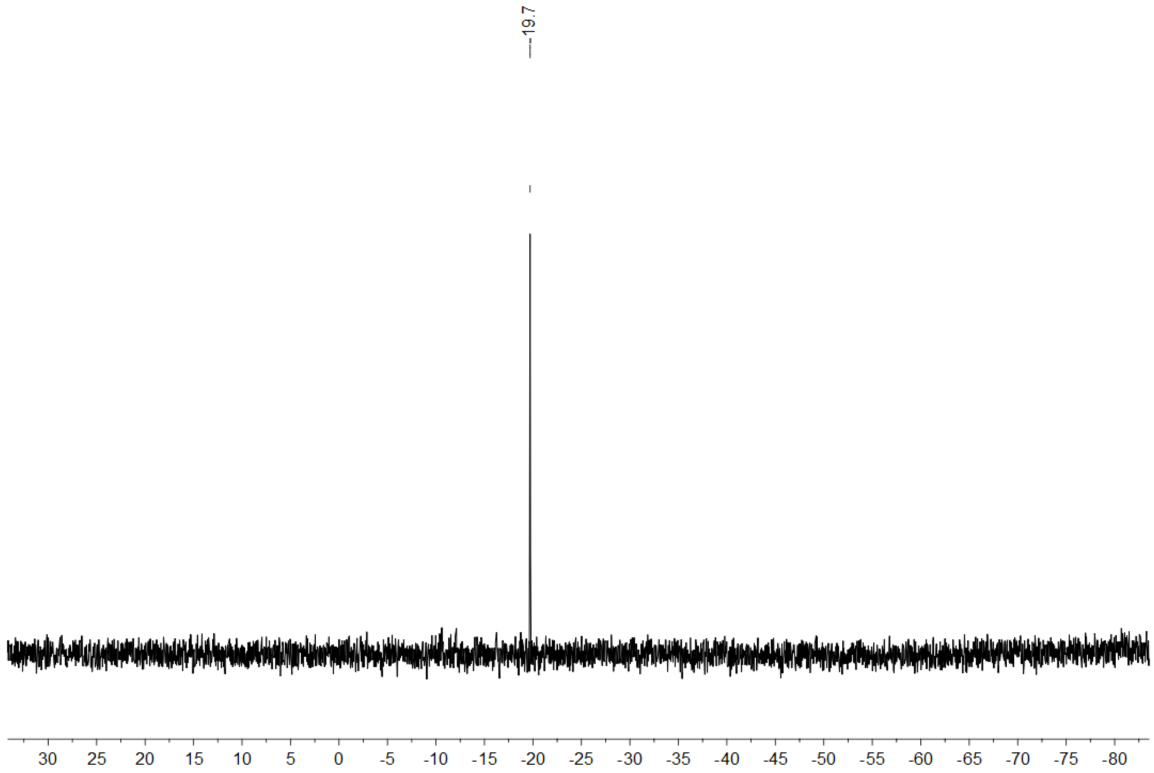

Figure S6. $^{29}Si\{^1H\}$ NMR spectrum (THF-d_8, 298 K) of compound **2**.

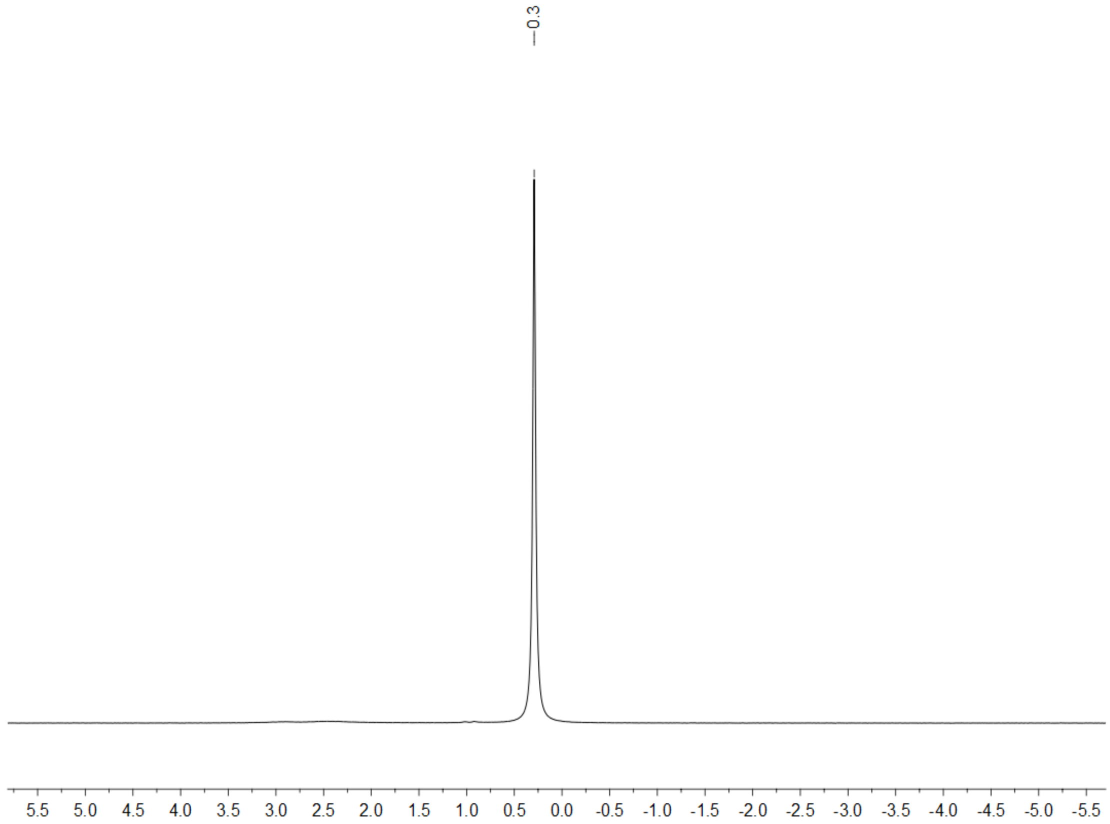

Figure S7. $^7Li\{^1H\}$ NMR spectrum (THF-d_8, 298 K) of compound **2**.

2.3. Synthesis of Compound 3

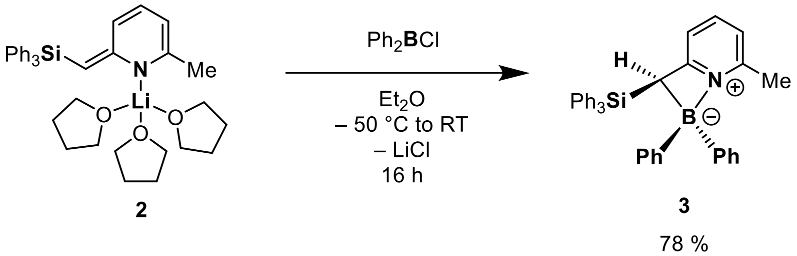

Chlorodiphenylborane (786 mg, 2.45 mmol, 1.1 equiv.) was dissolved in Et_2O (10 mL) and added slowly via PTFE cannula to a cold solution of compound **2** (1.31 g, 2.22 mmol, 1.0 equiv.) in Et_2O (30 mL) at –50 °C under constant stirring. The reaction mixture was allowed to warm gradually to room temperature and stirred overnight. Volatiles were removed in vacuo, and the residue was suspended in dichloromethane (30 mL) and transferred through a fritted column layered with Celite® by means of PTFE cannula. The solids were washed with dichloromethane (3 × 10 mL), and the combined pale-yellow filtrates were concentrated under reduced pressure. The resulting pale-yellow oily residue was suspended in pentane (~40 mL) and stirred for 4 h at room temperature. The suspension was filtered by cannula suction, the solids washed with pentane (5 mL), and the residue dried in vacuo to yield compound **3** as a white powder (920 mg, 1.74 mmol, 78 %). Crystals suitable for single-crystal X-ray diffraction analysis were obtained by slow evaporation of a saturated Et_2O solution of **3**.

^{1}H NMR (400.13 MHz, CD_2Cl_2, 25 °C): δ 2.31 (s, 3H, PyCH_3), 3.46 (s, 1H, SiCH), 6.73 (bd, $^3J_{HB}$ = 17.3 Hz, 3H, H_{BPh}), 6.91 (bs, 1H, H_{BPh}), 6.99 (d, $^3J_{HH}$ = 7.8 Hz, 1H, $H_{m\text{-Py}}$), 7.09 (d, $^3J_{HH}$ = 7.8 Hz, 1H, $H_{m\text{-Py}}$), 7.24 (m, 6H, H_{Ph}), 7.32 (m, 9H, H_{Ph}), 7.65 – 7.55 (m, 6H, H_{Ph}), 7.60 (bs, 1H, H_{BPh}), 7.70 (t, $^3J_{HH}$ = 7.8 Hz, 1H, $H_{p\text{-Py}}$). **^{13}C** NMR (100.62 MHz, CD_2Cl_2, 25 °C): δ 19.3 (s, Py-CH$_3$), 24.3 (bs, SiCH), 121.1 (s, $C_{m\text{-Py}}$), 121.9 (bs, $C_{m\text{-Py}}$), 125.4 (bd, $^2J_{CB}$ = 126.8 Hz, $C_{o\text{-BPh}}$), 126.8 (bd, $^3J_{CB}$ = 80.4 Hz, $C_{m\text{-BPh}}$), 127.5 (s, C_{Ph}), 133.4 (bs, C_{BPh}), 135.8 (s, C_{Ph}), 136.3 (s, C_{Ph}), 140.0 (s, $C_{p\text{-Py}}$), 147.7 (bd, $^1J_{CB}$ = 289.3 Hz, $C_{i\text{-BPh}}$), 154.5 (bs, $C_{o\text{-Py}}$), 167.1 (bs, $C_{o\text{-Py}}$). **^{11}B{^{1}H}** NMR (128.38 MHz, CD_2Cl_2, 25 °C): δ 7.2 (bs). **^{29}Si{^{1}H}** NMR (79.49 MHz, Tol-d_8, 25 °C): δ – 11.8 (s). **HRMS (EI$^+$)**, calcd. m/z for {[(M + H) – BPh$_2$]$^+$}: 366.16725; found 366.16754. **Elemental analysis**: calcd. for $C_{37}H_{32}BNSi$: C 83.92, H 6.09, N 2.65; found: C 82.73, H 6.21, N 2.58.

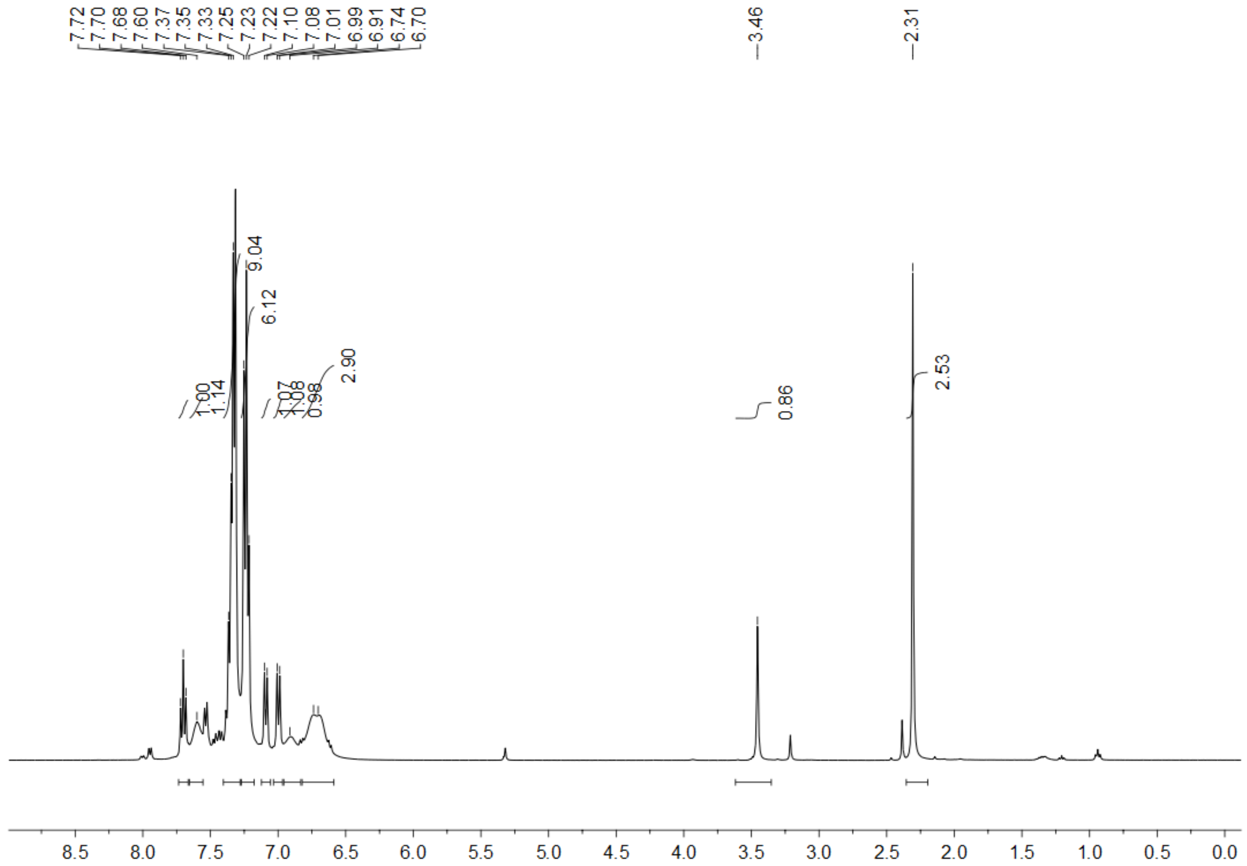

Figure S8. ^{1}H NMR spectrum (CD_2Cl_2, 298 K) of compound **3**. The unassigned ^{1}H NMR signals can be attributed to small amounts of hydrolyzed product, corresponding to compound **1**.

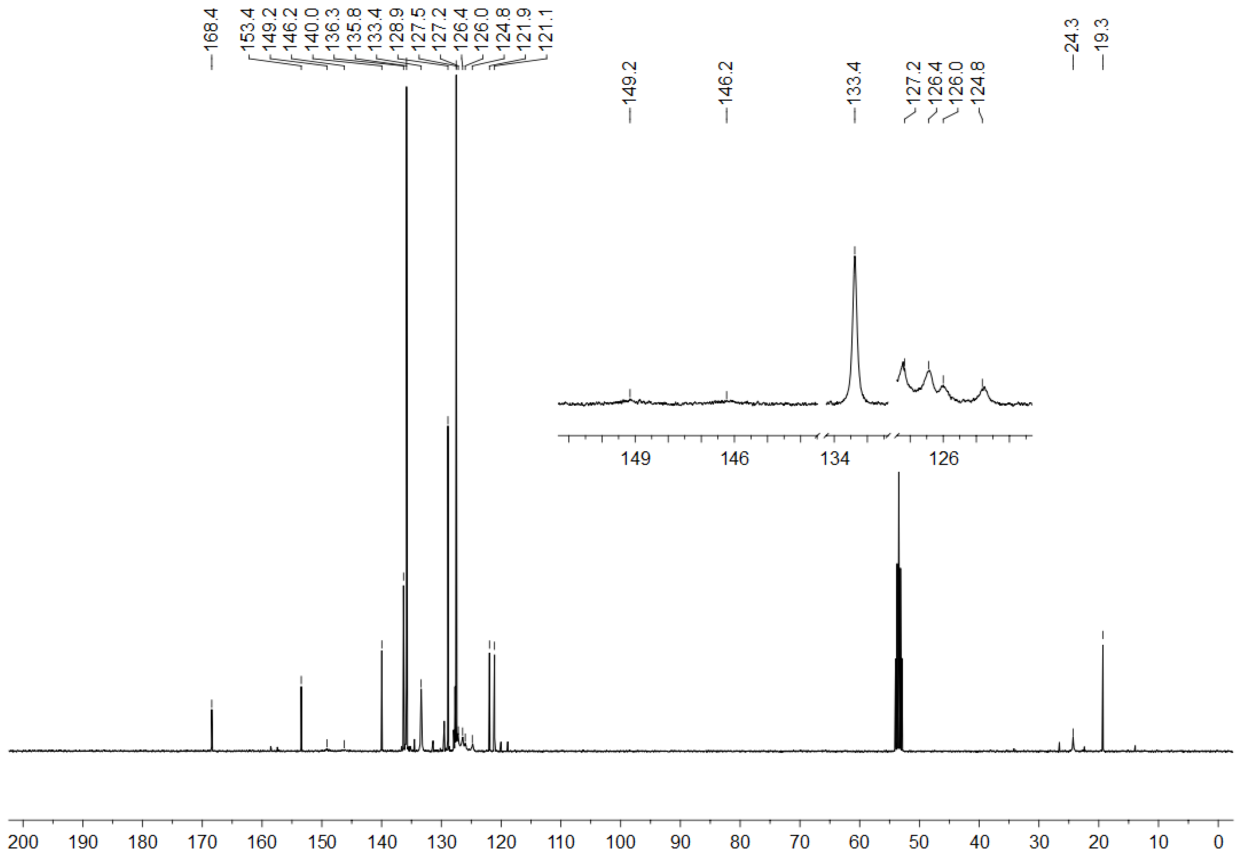

Figure S9. ^{13}C NMR spectrum (CD_2Cl_2, 298 K) of compound **3**.

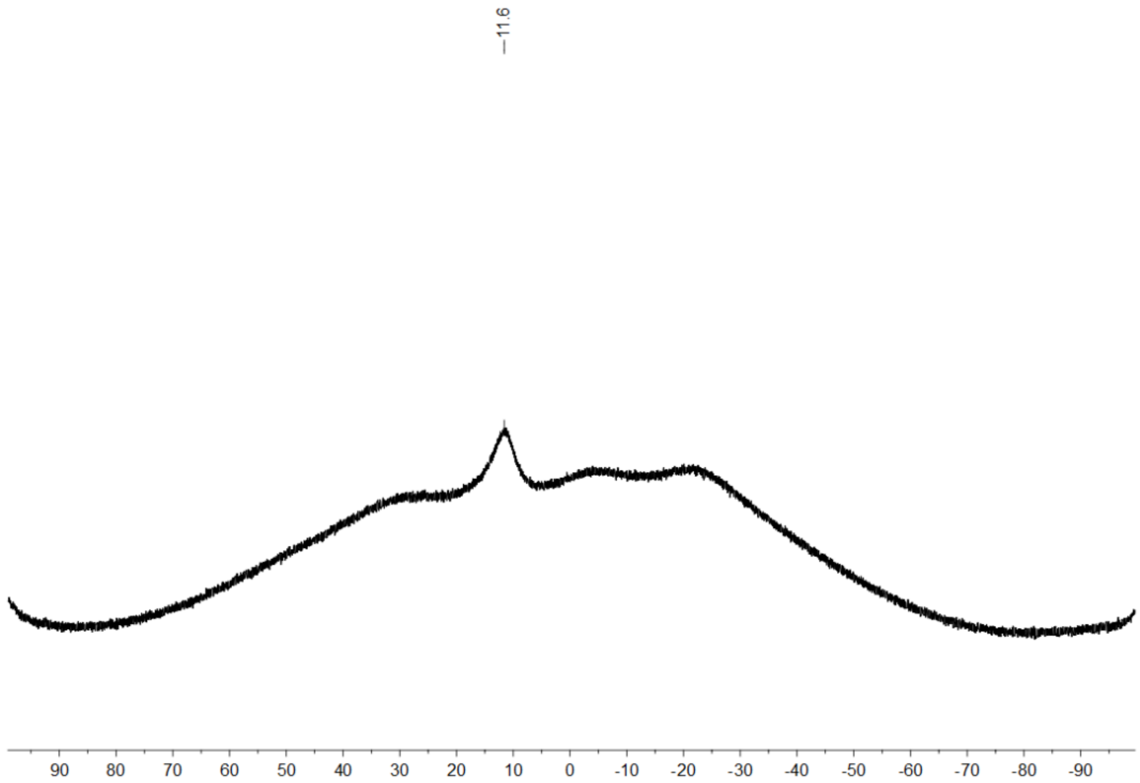

Figure S10. ^{11}Bi{^{1}H} NMR spectrum (CD_2Cl_2, 298 K) of compound **3**.

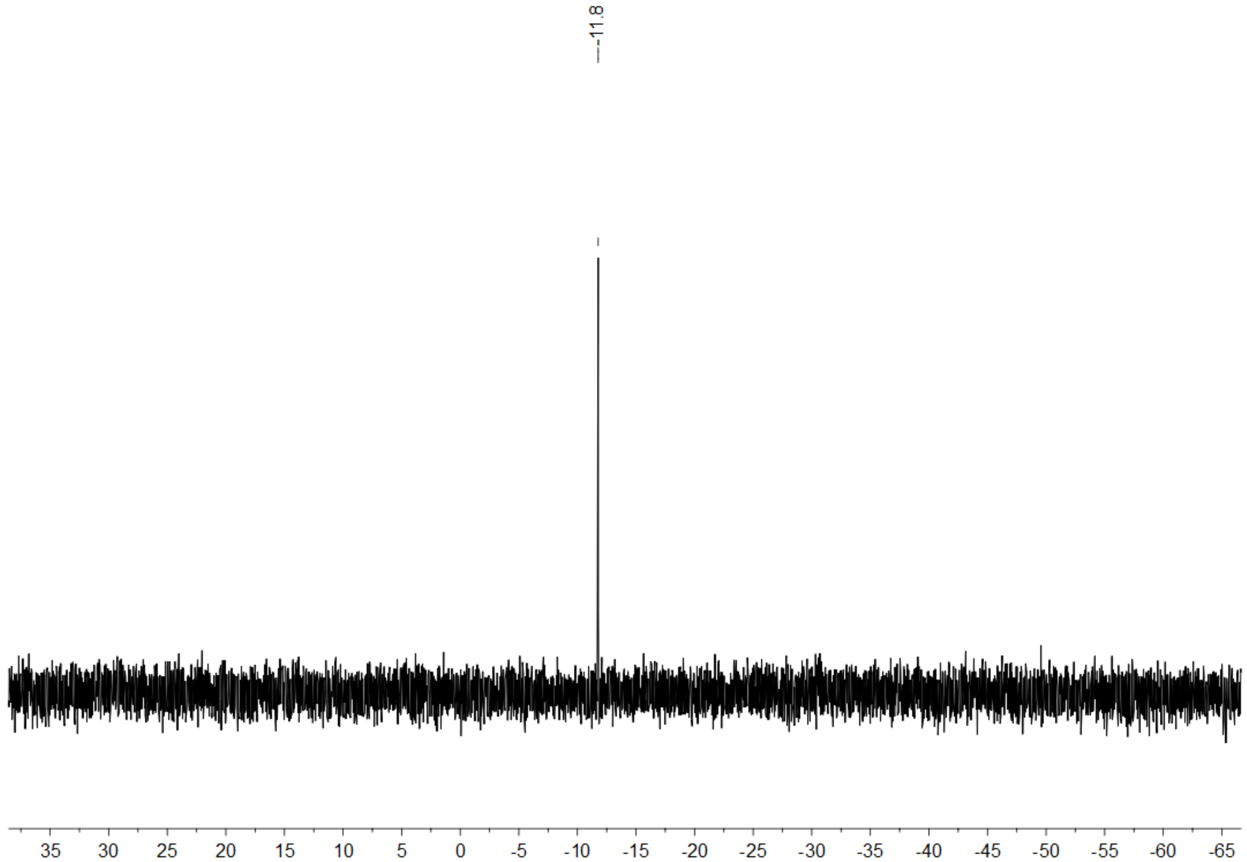

Figure S11. ^{29}Si{^{1}H} NMR spectrum (Tol-d_8, 298 K) of compound **3**.

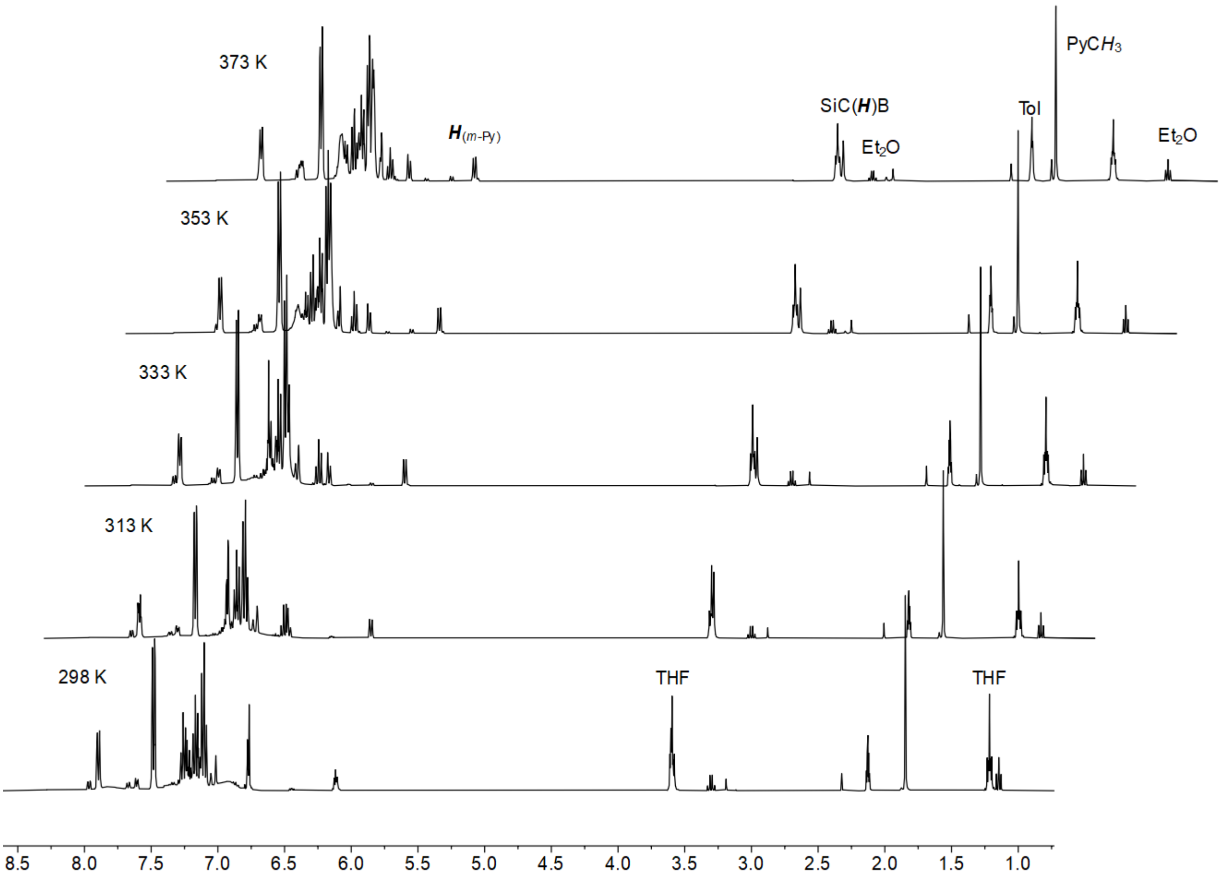

Figure S12. Variable-high-temperature (VHT) ^{1}H NMR spectrum (Tol-d_8) of compound **3**. No significant changes were observed over the course of the experiment (298–373 K).

2.4. Synthesis of Compound 4

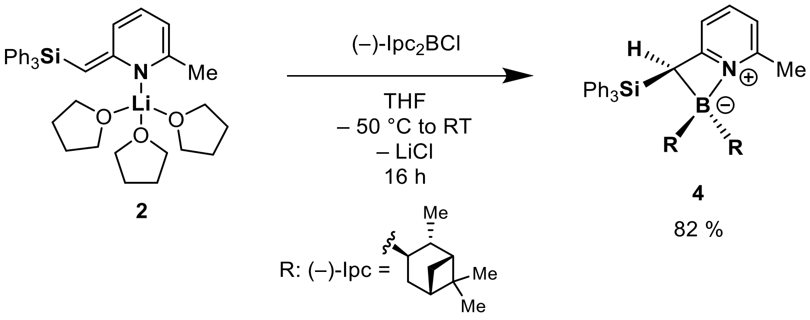

Chlorodi-(–)-*iso*-pinocampheylborane (900 mg, 2.81 mmol, 1.1 equiv.) was dissolved in THF (10 mL) and added slowly via PTFE cannula to a cold solution of compound **2** (1.50 g, 2.55 mmol, 1.0 equiv.) in THF (30 mL) at –50 °C under constant stirring. The reaction was allowed to warm to room temperature and stirred overnight. Volatiles were removed under reduced pressure, and the residue was suspended in dichloromethane (30 mL) and filtered through a Celite® pad. The solids were washed with dichloromethane (3 × 10 mL), and the combined yellow filtrates were dried in vacuo. The pale-yellow oily residue was suspended in hexane (~40 mL) and stirred for 1 h at room temperature. After filtration by cannula suction, the solids were washed with hexane (5 mL) and dried in vacuo to give compound **4** as a beige powder. The beige filtrates were stored at –30 °C for three days, affording a second crop of beige crystalline material suitable for single-crystal X-ray diffraction analysis. No significant spectroscopic

differences were observed between the powder and the crystalline material. The combined yield of **4** was 1.35 g (2.08 mmol, 82 %).

^{1}H NMR (400.13 MHz, THF-d_8, 25 °C): δ 0.58 (bs, 2H, C$H_{2\ Ipc}$), 0.84 (bd, $^{3}J_{HH}$ = 6.4 Hz, 6H, 2 × CHC$H_{3\ Ipc}$), 1.01 (bs, 6H, C(CH_3)$_{2\ Ipc}$), 1.08 (bs, 6H, C(CH_3)$_{2\ Ipc}$), 1.49 (bm, 2H, 2 × CH_{Ipc}), 1.58 (bm, 2H, C$H_{2\ Ipc}$), 1.65 (bm, 2H, 2 × CH_{Ipc}), 1.74 – 1.89 (bm, 6H, 4 × C*H*, C$H_{2\ Ipc}$), 1.98 (bm, 2H, C$H_{2\ Ipc}$), 2.54 (s, 3H, PyCH_3), 3.11 (bs, 1H, SiC*H*), 6.73 (d, $^{3}J_{HH}$ = 7.7 Hz, 1H, $H_{m\text{-Py}}$), 6.99 (d, $^{3}J_{HH}$ = 7.7 Hz, 1H, $H_{m\text{-Py}}$), 7.34 – 7.20 (m, 9H, H_{Ph}), 7.50 (t, $^{3}J_{HH}$ = 7.7 Hz, 1H, $H_{p\text{-Py}}$), 7.65 – 7.55 (m, 6H, H_{Ph}). **^{13}C NMR** (100.62 MHz, THF-d_8, 25 °C): δ 22.1 (s, Py-CH_3), 22.5 (s, C$H_{3\ Ipc}$), 24.1 (bs, CH(CH_3)$_{2\ Ipc}$), 27.8 (s, C$H_{3\ Ipc}$), 28.9 (bs, Si*C*H), 31.3 (bs, C$H_{2\ Ipc}$), 32.7 (s, C$H_{2\ Ipc}$), 39.0 (s, CH_{Ipc}), 42.0 (s, CH_{Ipc}), 49.6 (s, CH_{Ipc}), 120.3 (s, $C_{m\text{-Py}}$), 121.1 (bs, $C_{m\text{-Py}}$), 127.3 (s, C_{Ph}), 128.7 (s, C_{Ph}), 136.1 (s, C_{Ph}), 137.3 (s, C_{Ph}), 138.2 (bs, $C_{p\text{-Py}}$), 154.5 (bs, $C_{o\text{-Py}}$), 167.1 (bs, $C_{o\text{-Py}}$). **^{11}B{^{1}H} NMR** (128.38 MHz, THF-d_8, 25 °C): δ 25.2 (bs). **^{29}Si{^{1}H} NMR** (79.49 MHz, THF-d_8, 25 °C): δ – 11.8 (s). **HRMS (EI^{+}),** calcd. m/z for {[(M + H) - BIpc$_2$]$^{+}$}: 366.1633; found 366.1677. **Elemental analysis**: calcd. for $C_{45}H_{56}BNSi$: C 83.17, H 8.69, N 2.16; found: C 83.18, H 8.59, N 1.97.

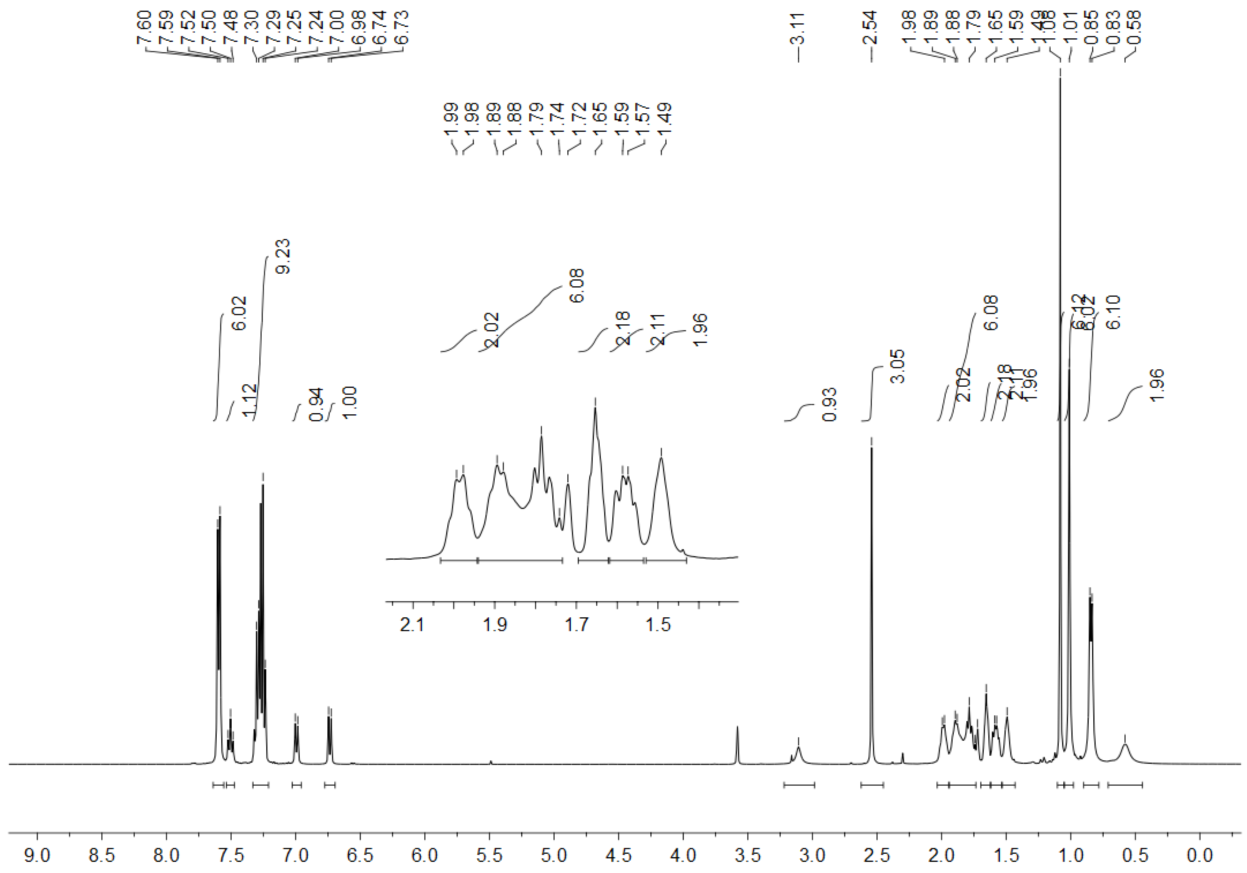

Figure S13. ^{1}H NMR spectrum (THF-d_8, 298 K) of compound **4**.

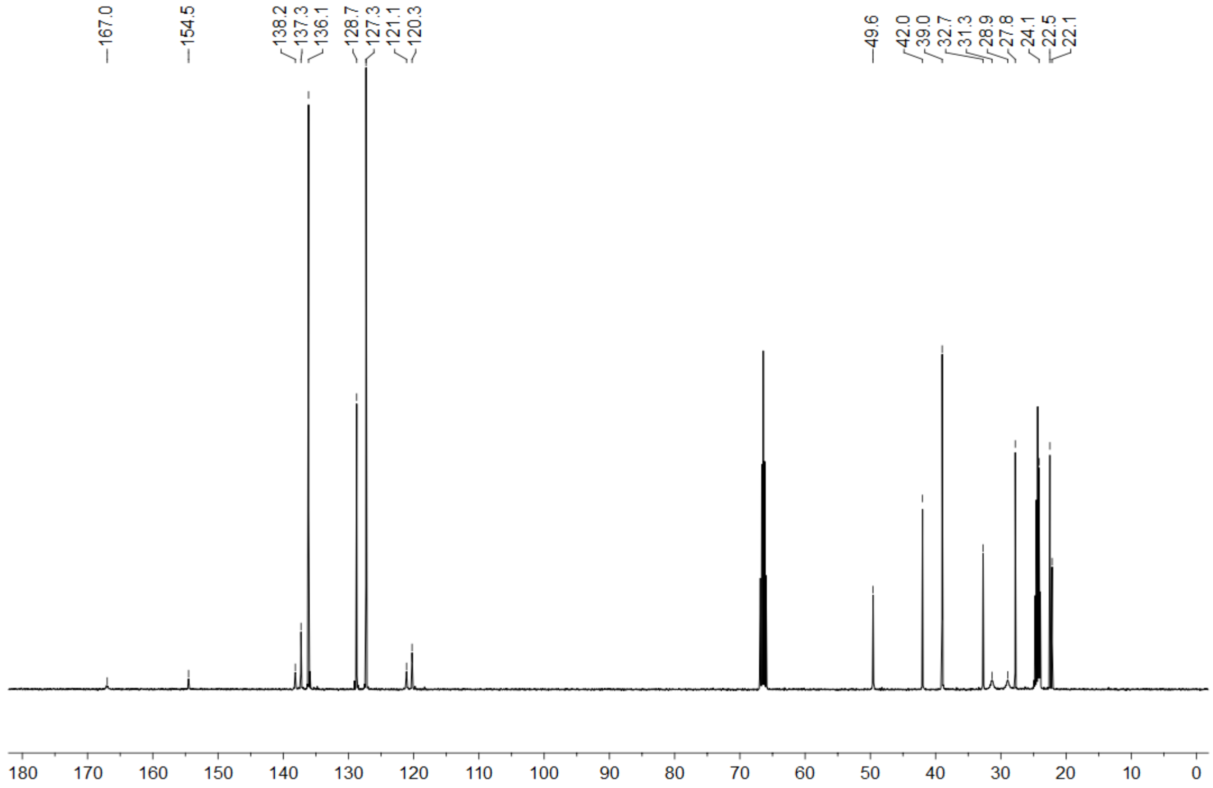

Figure S14. ^{13}C NMR spectrum (THF-d_8, 298 K) of compound **4**.

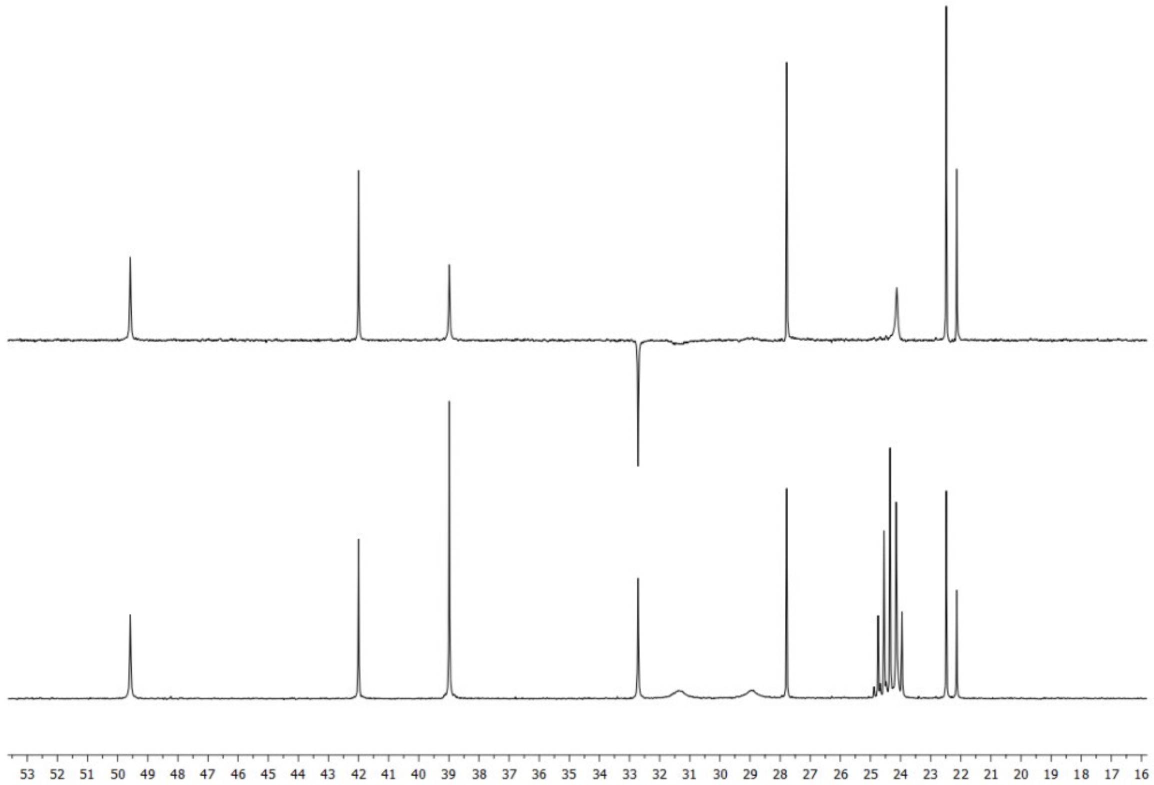

Figure S15. ^{13}C DEPT-135 (top) and ^{13}C (bottom) NMR spectra (THF-d_8, 298 K), relevant for the unambiguous determination of the signals of compound **4**.

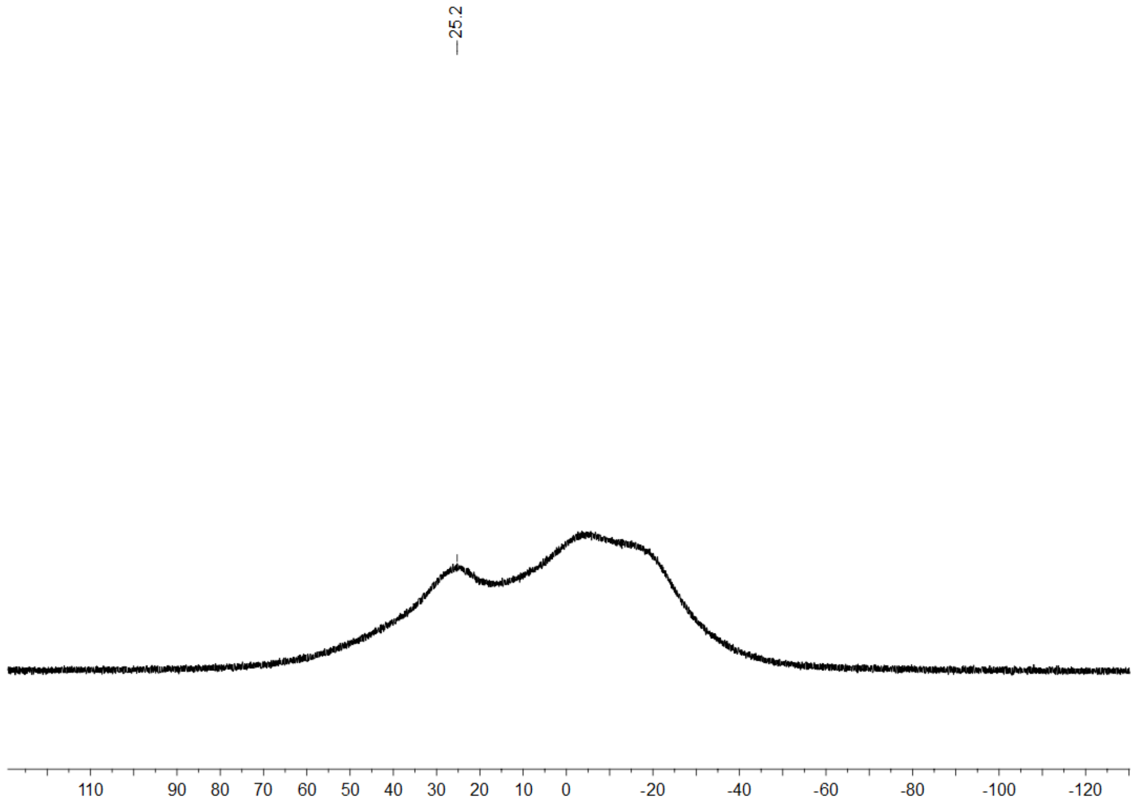

Figure S16. $^{11}B\{^1H\}$ NMR spectrum (THF-d_8, 298 K) of compound **4**.

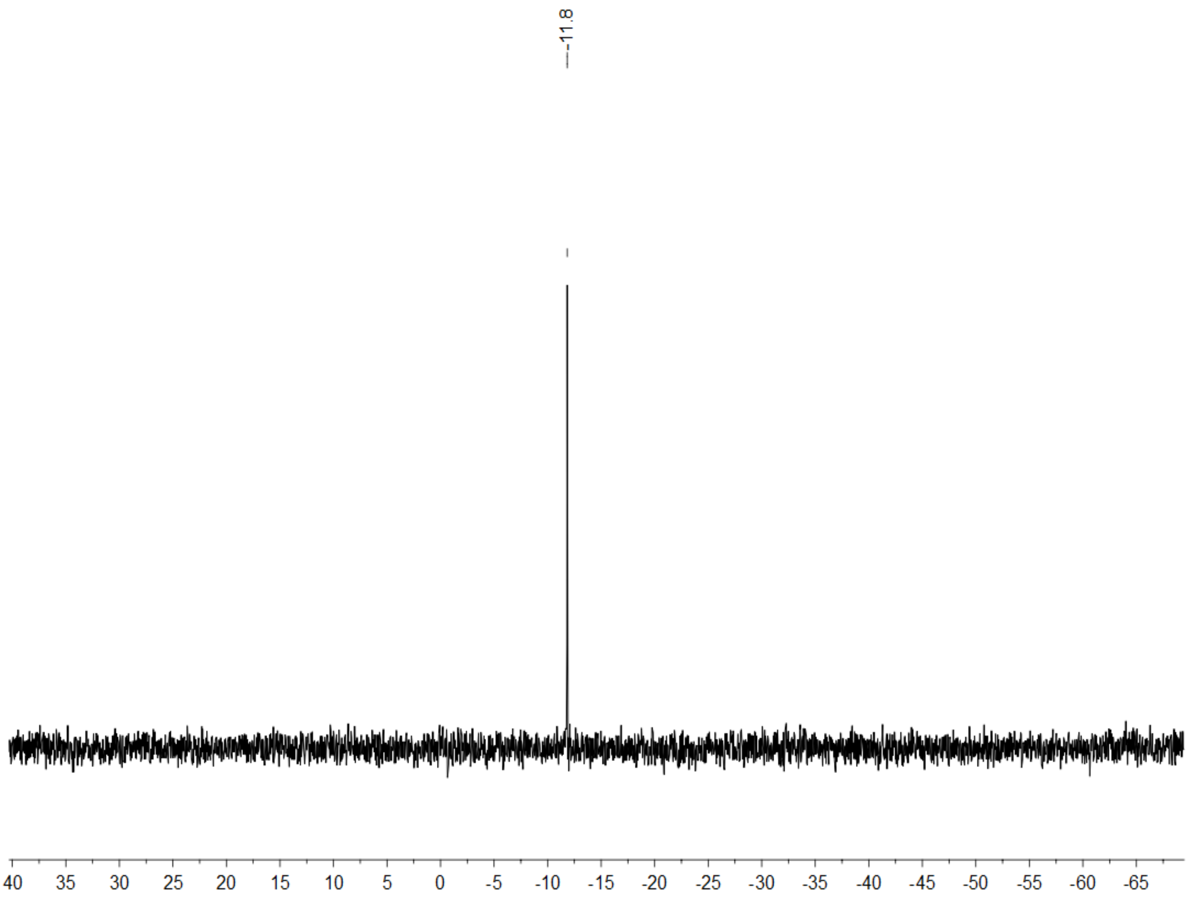

Figure S17. $^{29}Si\{^1H\}$ NMR spectrum (THF-d_8, 298 K) of compound **4**.

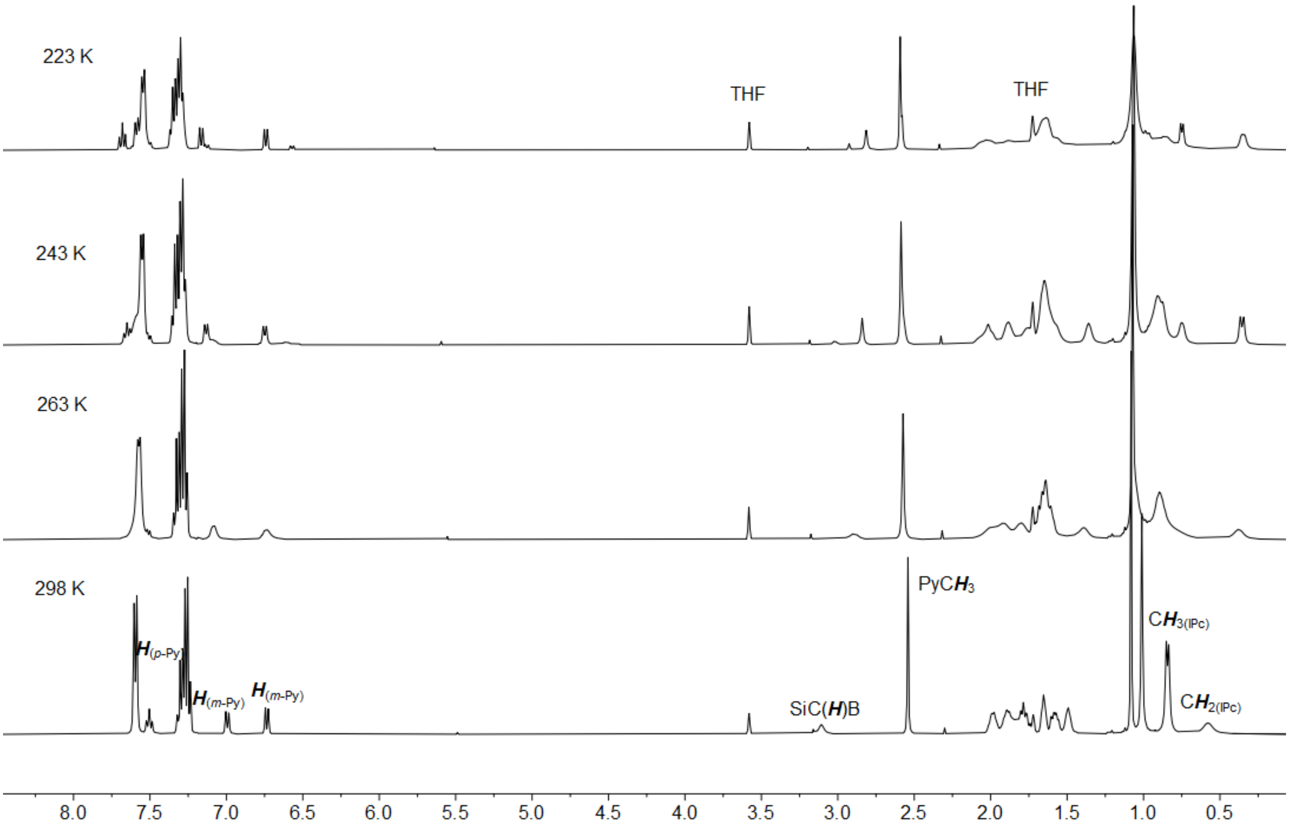

Figure S18. Variable-low-temperature (VLT) ^{1}H NMR spectrum (THF-d_8) of compound **4**. The signal for the methine group bound to boron and silicon (δ = 3.11 ppm at 298 K) shows the largest changes upon cooling: it shifts 0.22 ppm high-field at 263 K and splits into two signals at 2.93 and 2.81 ppm (1:4) at 243 K and 223 K. Aromatic protons of the pyridine fragment exhibit low-field shifts and splitting in the same 1:4 ratio. The methyl group on the pyridine also shows splitting, but with smaller separation, leading to overlapping signals at δ = 2.58 ppm at 223 K. These changes are consistent with restricted rotation at lower temperatures.

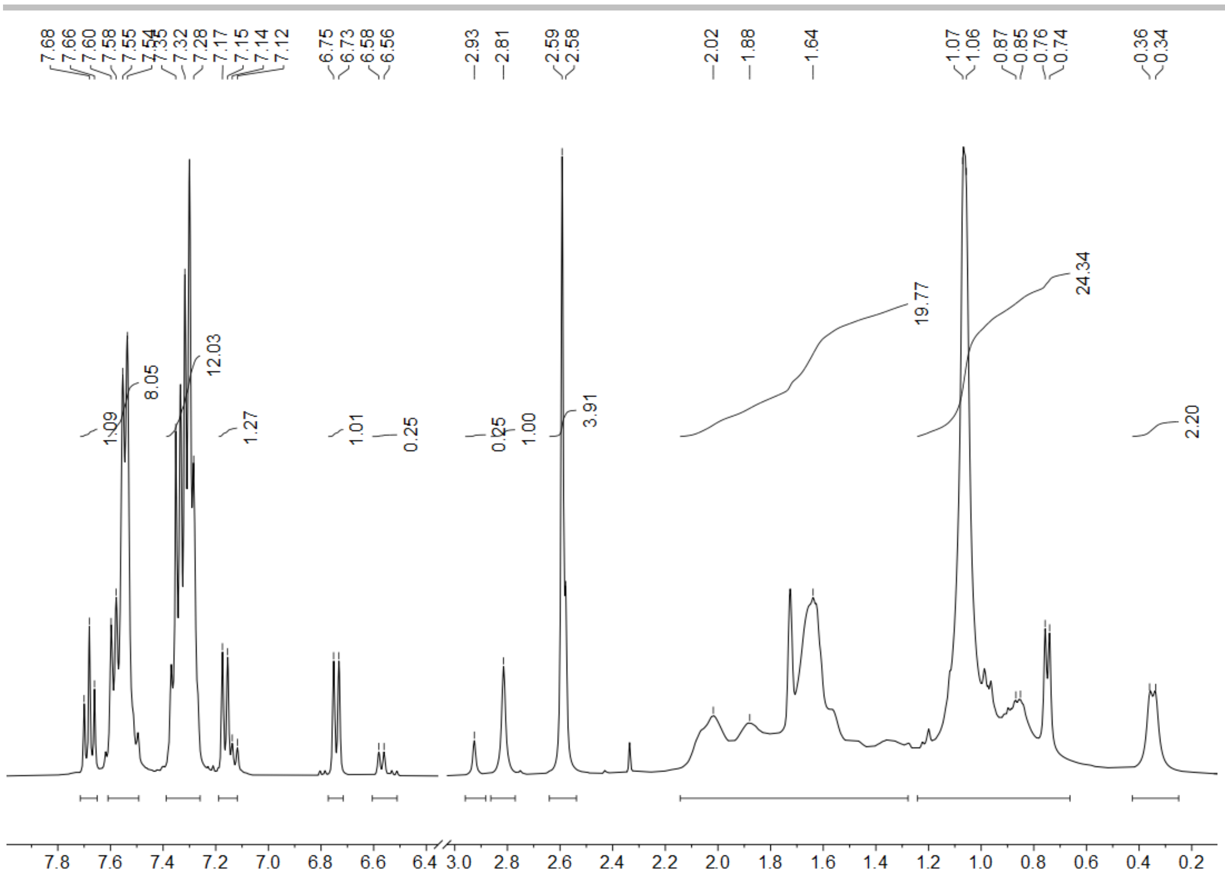

Figure S19. Enlarged section of the variable-low-temperature (VLT) ^{1}H NMR spectrum (THF-d_8) of compound **4**.

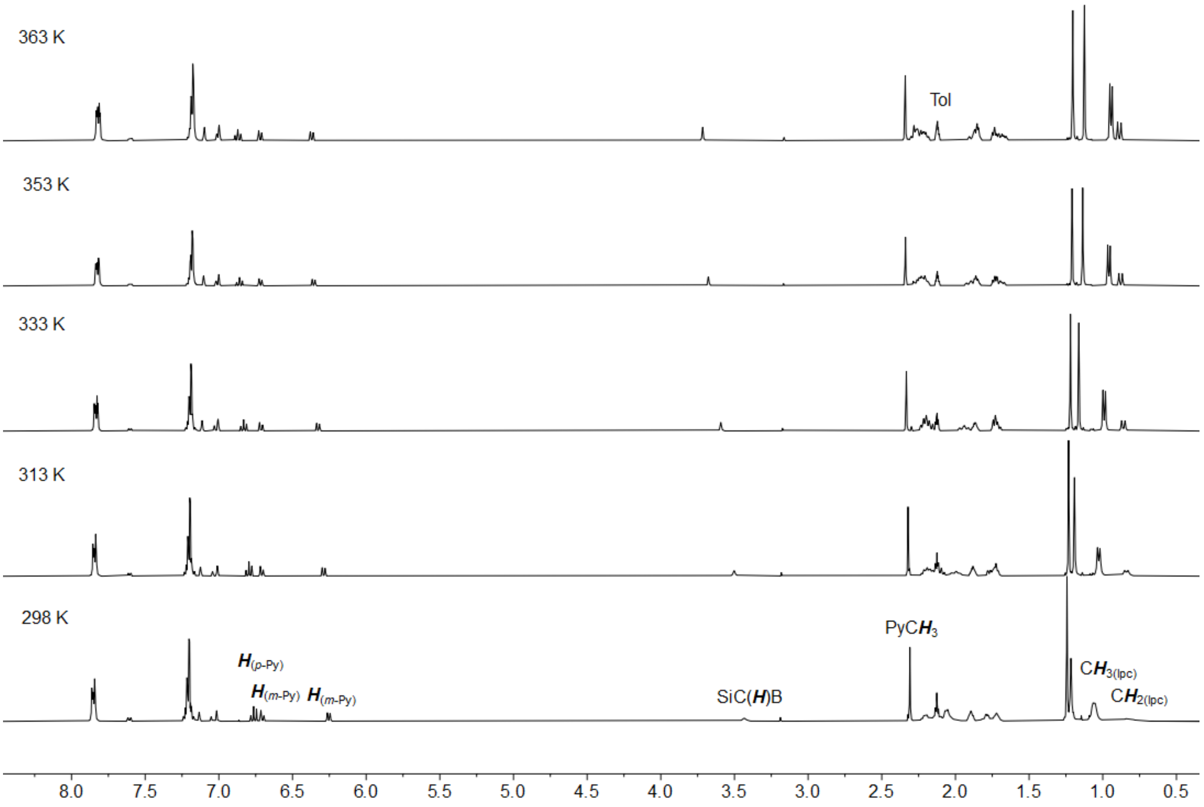

Figure S20. Variable-high-temperature (VHT) ^{1}H NMR spectrum (Tol-d_8) of compound **4**. The signal for the methine group bound to boron and silicon (δ = 3.43 ppm at 298 K) shows the largest changes upon heating: it shifts progressively downfield, reaching a 0.30 ppm shift difference at 363 K. Two aromatic pyridine protons (m- and p-positions) exhibit analogous but smaller downfield shifts (0.12 ppm difference at 363 K). No signal splitting is observed. The continuous downfield shift of the SiC(*H*)B proton may indicate a partial population of the open enamide form under thermal conditions.

2.5. Synthesis of Compound 5

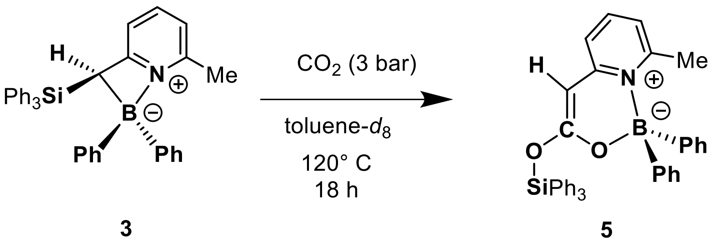

99% (52%)[a]

[a]Isolated yield in brackets

An oven-dried intermediate-pressure Young-type NMR tube was charged under argon in a glovebox with compound **3** (53 mg, 0.10 mmol) and toluene-d_8 (0.5 mL). The NMR tube was connected to a Schlenk line equipped with a pressure gauge manometer. After three freeze–pump–thaw cycles, dry CO_2 was condensed into the tube cooled with liquid nitrogen, ca. 2 cm above the frozen solution (~1 min). The valve was closed, and the tube was allowed to warm to room temperature. Initial experiments under 1 bar CO_2 pressure were unsuccessful (see Figures S21 and S22). The internal pressure was then adjusted to 3 bar CO_2 by releasing excess gas. Formation of compound **5** was monitored by NMR. After standing at room temperature for 16 h, no conversion was observed. Heating at 90 °C for 16 h resulted in only minimal conversion to compound **5** (< 1%). Heating at 120 °C overnight led to full conversion. Replacement of the CO_2 atmosphere by inert gas rendered compound **5** stable. The crude mixture was dried in vacuo, and the dark-orange oily residue washed with pentane (2 × 3 mL), affording spectroscopically pure **5** as an orange solid (30 mg, 0.05 mmol, 52 %). Crystals suitable for single-crystal X-ray diffraction analysis were obtained by slow evaporation of a C_6D_6 solution of **5**.

Spectroscopic data from the crude mixture under CO_2 atmosphere (3 bar):
^{1}H NMR (400.13 MHz, Tol-d_8, 25 °C): δ 1.86 (s, 3H, CH_3), 5.10 (s, 1H, C=C*H*), 6.02 (dd, $^3J_{HH}$ = 7.6 Hz, $^4J_{HH}$ = 0.6 Hz, 1H, $H_{m\text{-Py}}$), 6.35 (dd, $^3J_{HH}$ = 8.5 Hz, $^4J_{HH}$ = 0.6 Hz, 1H, $H_{m\text{-Py}}$), 6.81 (ddd, $^3J_{HH}$ = 8.5 Hz, $^3J_{HH}$ = 7.6 Hz, $^4J_{HH}$ = 0.6 Hz, 1H, $H_{p\text{-Py}}$), 7.34 – 7.41 (m, 19H, H_{Ph}), 7.62 (m, 6H, H_{Ph}). **^{13}C NMR** (100.62 MHz, Tol-d_8, 25 °C): δ 25.7 (s, CH$_3$), 78.4 (s, C=*C*H), 119.1 (s, $C_{m\text{-Py}}$), 119.1 (s, $C_{m\text{-Py}}$), 124.9 (s, CO_2), 126.4 (s, C_{Ph}), 127.4 (s, C_{Ph}), 128.0 (s, C_{Ph}), 130.5 (s, C_{Ph}), 133.2 (s, $C_{p\text{-Py}}$), 133.7 (s, C_{Ph}), 136.3 (s, C_{Ph}), 136.5 (s, C_{Ph}), 137.5 (s, C_{Ph}), 148.7 (bs, C_{Ph}), 154.0 (s, $C_{o\text{-Py}}$), 158.0 (s, *C*=CH), 164.5 (bs, $C_{o\text{-Py}}$). **^{11}B{^{1}H} NMR** (128.38 MHz, Tol-d_8, 25 °C): δ 7.2 (bs).

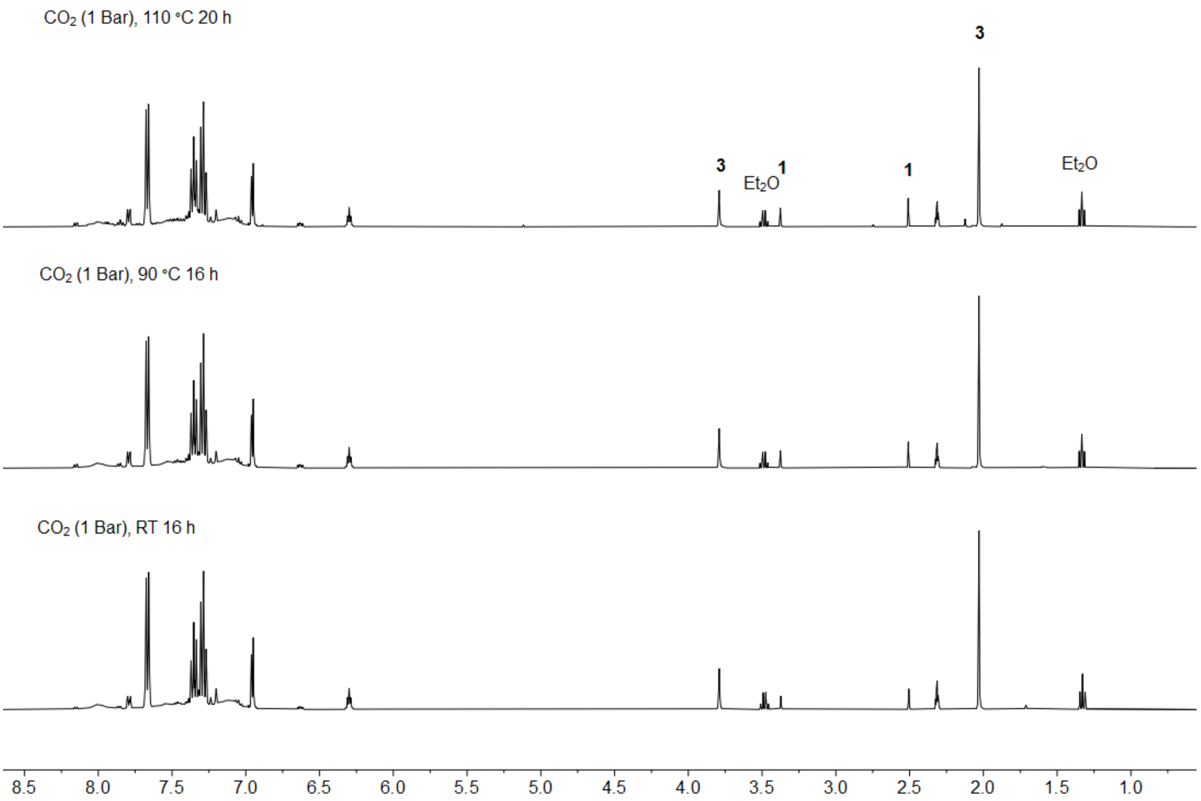

Figure S21. ^{1}H NMR spectrum (Tol-d_8, 298 K) of the crude mixture under a CO_2 atmosphere (1 bar). No CO_2 activation was observed at room temperature after 16 h (bottom), at 90 °C after 16 h (middle), or at 110 °C after 20 h (top). Small amounts of hydrolyzed starting material can also be detected.

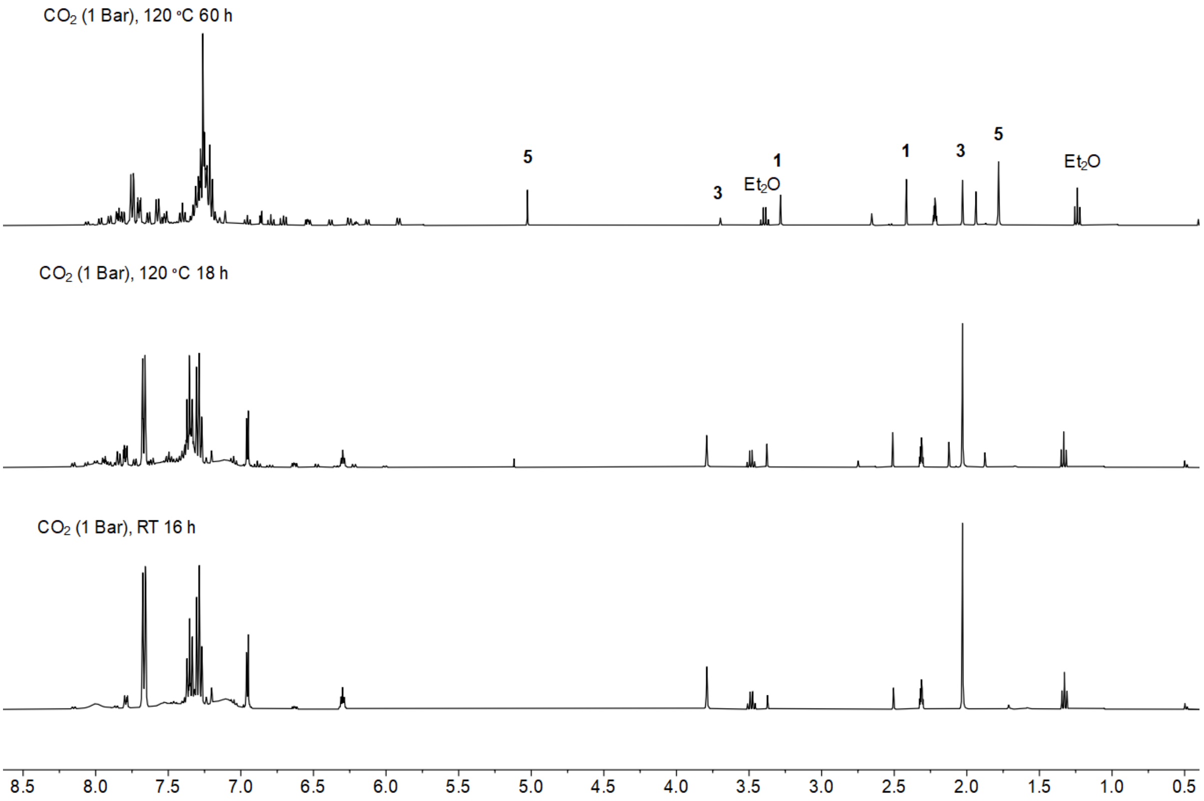

Figure S22. ^{1}H NMR spectrum (Tol-d_8, 298 K) of the crude mixture under a CO_2 atmosphere (1 bar). No CO_2 activation was observed at room temperature after 16 h (bottom). At 120 °C, 8% conversion to compound **5** was observed after 18 h (middle), and 35% conversion after 60 h (top), with respect to an internal standard (hexamethyldisiloxane). A significantly increased amount of hydrolyzed starting material (compound **1**) is detected.

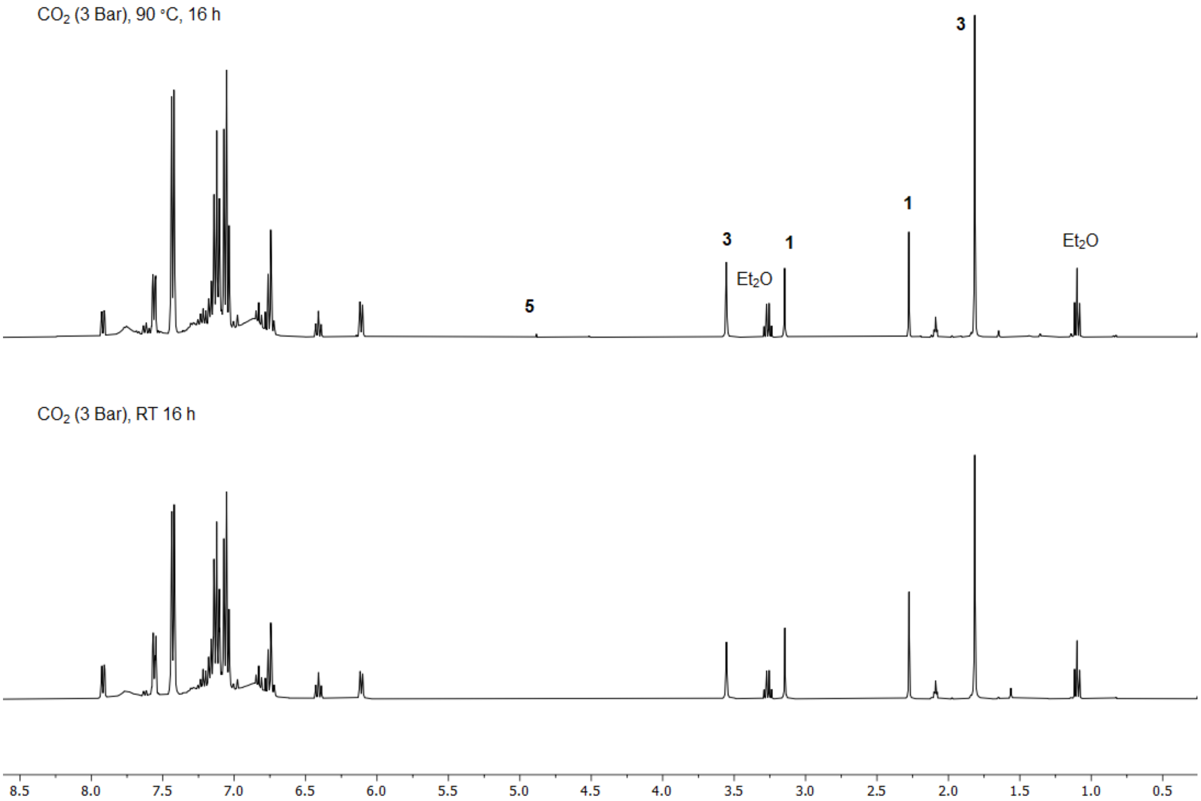

Figure S23. ^{1}H NMR spectrum (Tol-d_8, 298 K) of the crude mixture after standing overnight at room temperature for 16 h (bottom) followed by heating at 90 °C for 16 h (top) under a CO_2 atmosphere (3 bar). Small amounts of hydrolyzed starting material can also be detected.

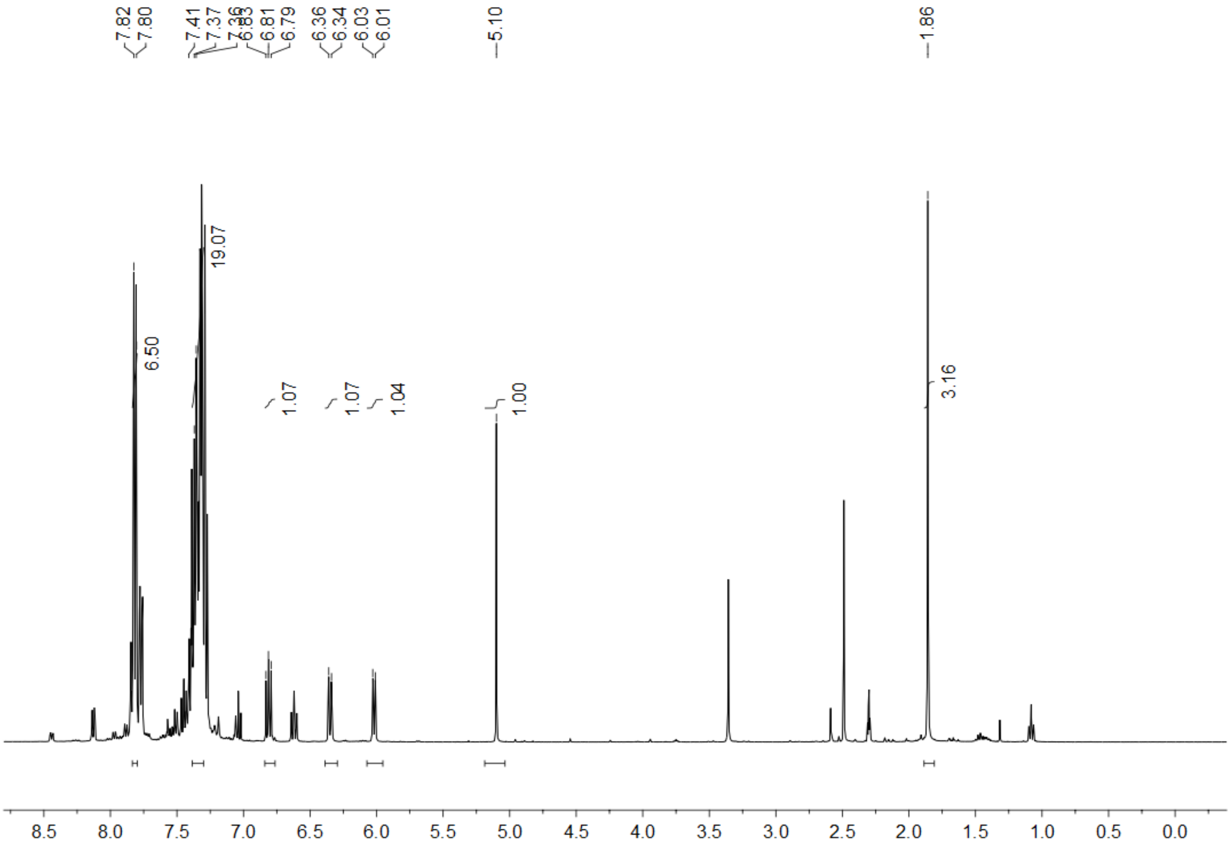

Figure S24. ^{1}H NMR spectrum (Tol-d_8, 298 K) of the crude mixture containing compound **5** under a CO_2 atmosphere (3 bar) after heating at 120°C for 16 h. The unassigned ^{1}H NMR signals can be attributed to small amounts of hydrolyzed starting material, corresponding to compound **1**.

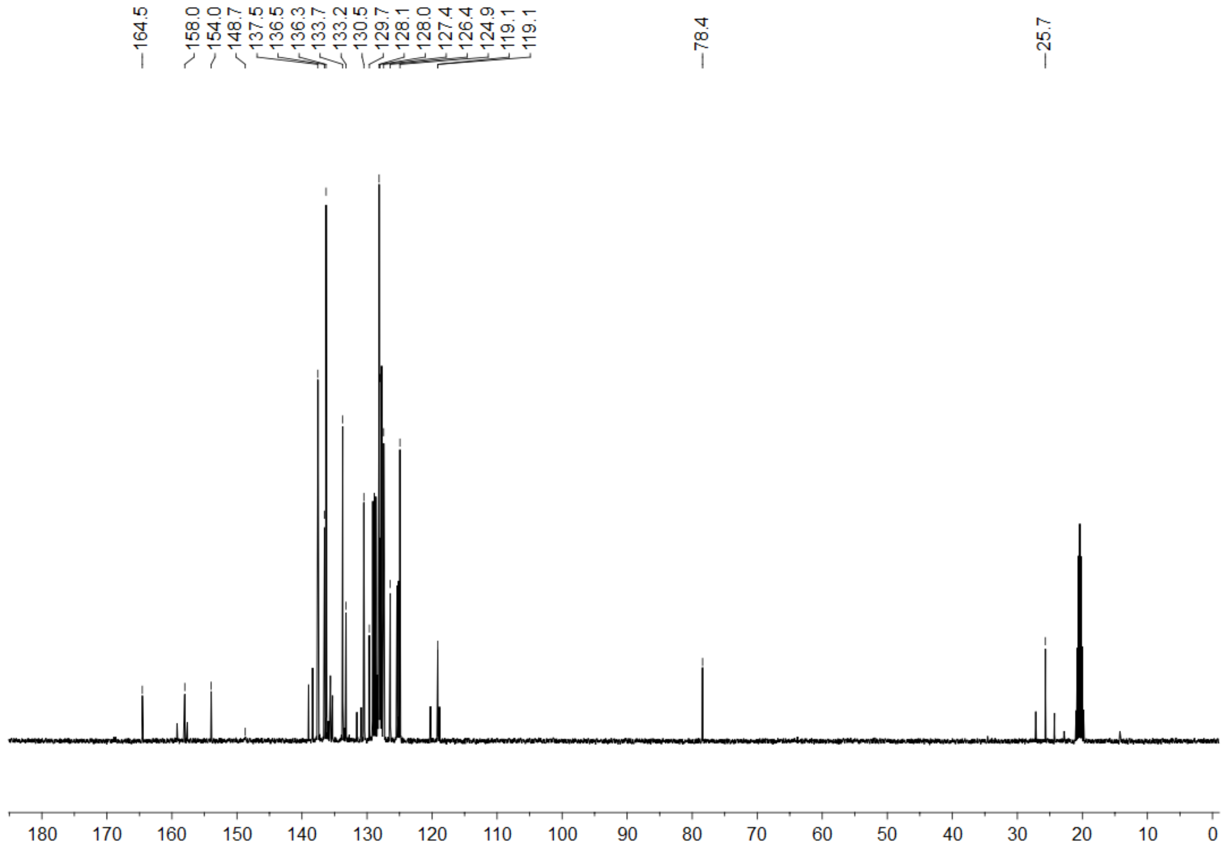

Figure S25. ^{13}C NMR spectrum (Tol-d_8, 298 K) of the crude mixture containing compound **5** under a CO_2 atmosphere (3 bar).

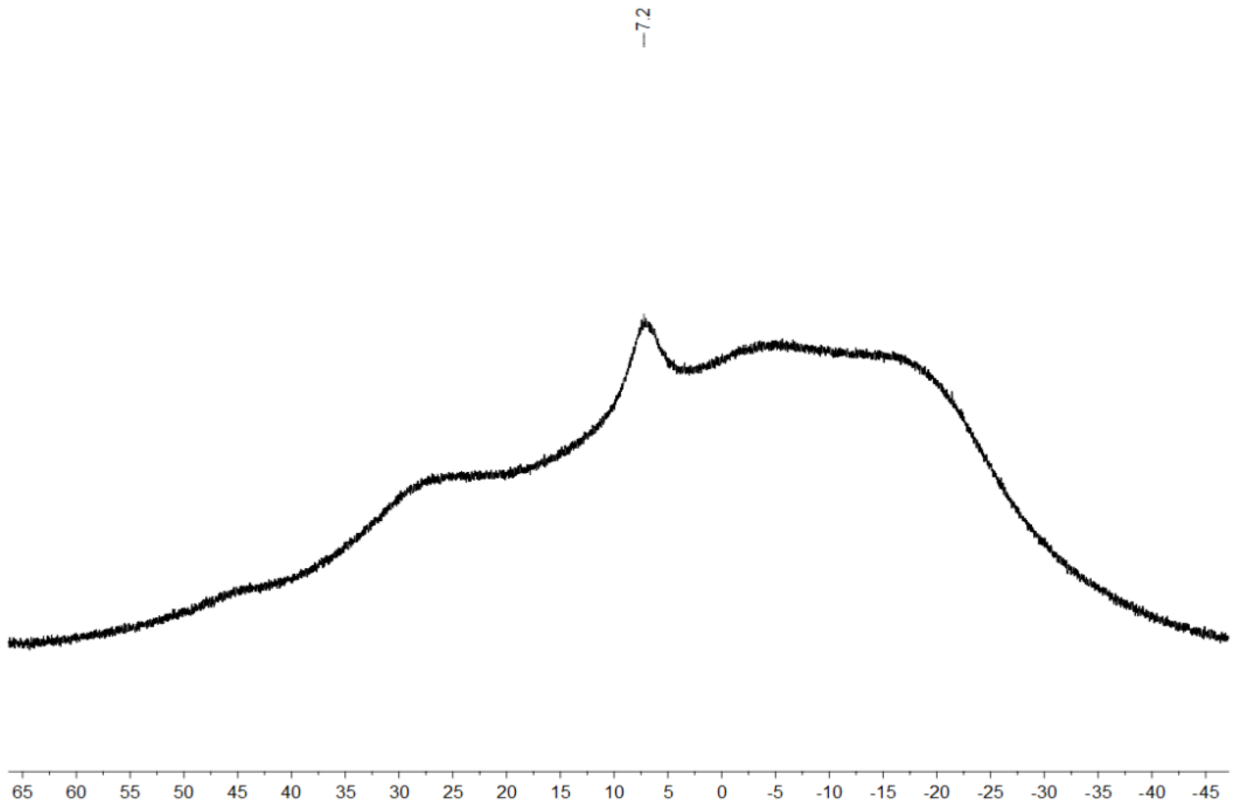

Figure S26. $^{11}B\{^{1}H\}$ NMR spectrum (Tol-d_8, 298 K) of the crude mixture containing compound **5** under a CO_2 atmosphere (3 bar).

Spectroscopic data of the isolated compound **5**:

^{1}H NMR (400.13 MHz, C_6D_6, 25 °C): δ 1.62 (s, 3H, C*H*$_3$), 4.91 (s, 1H, C=C*H*), 5.72 (d, $^{3}J_{HH}$ = 7.9 Hz, 1H, $H_{m\text{-Py}}$), 6.07 (d, $^{3}J_{HH}$ = 7.9 Hz, 1H, $H_{m\text{-Py}}$), 6.48 (t, $^{3}J_{HH}$ = 7.9 Hz, 1H, $H_{p\text{-Py}}$), 7.05 (m, 6H, H_{Ph}), 7.14 (m, 13H, H_{Ph}), 7.62 (m, 6H, H_{Ph}). **^{13}C NMR** (100.62 MHz, C_6D_6, 25 °C): δ 25.4 (s, *C*H$_3$), 78.1 (s, C=*C*H), 118.7 (s, $C_{m\text{-Py}}$), 118.8 (s, $C_{m\text{-Py}}$), 126.1 (s, C_{Ph}), 127.1 (s, C_{Ph}), 127.8 (s, C_{Ph}), 130.2 (s, C_{Ph}), 132.8 (s, $C_{p\text{-Py}}$), 133.4 (s, C_{Ph}), 135.9 (s, C_{Ph}), 138.0 (s, C_{Ph}), 148.3 (bs, C_{Ph}), 153.5 (bs, $C_{o\text{-Py}}$), 157.5 (s, *C*=CH), 164.1 (bs, $C_{o\text{-Py}}$). **^{11}B{^{1}H} NMR** (128.38 MHz, C_6D_6, 25 °C): δ 7.4 (bs). **^{29}Si{^{1}H} NMR** (79.49 MHz, C_6D_6, 25 °C): δ – 10.3 (s). **HRMS (ESI$^+$)**, calcd. m/z for [M$^+$]: 574.2368; found 574.2376. **Elemental analysis**: calcd. for $C_{38}H_{32}BNO_2Si$: C 79.57, H 5.62, N 2.44; found: C 78.60, H 5.82, N 2.39.

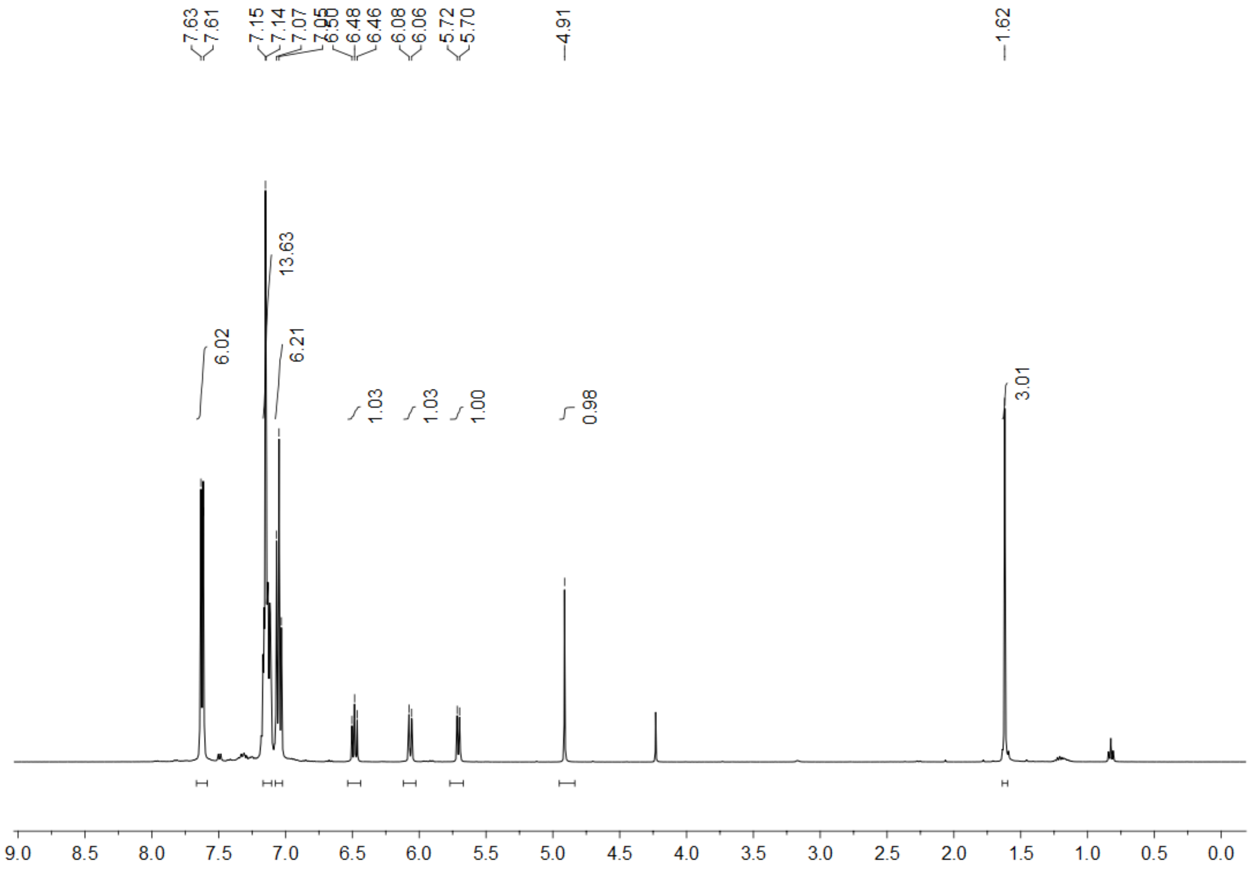

Figure S27. ^{1}H NMR spectrum (C_6D_6, 298 K) of compound **5**.

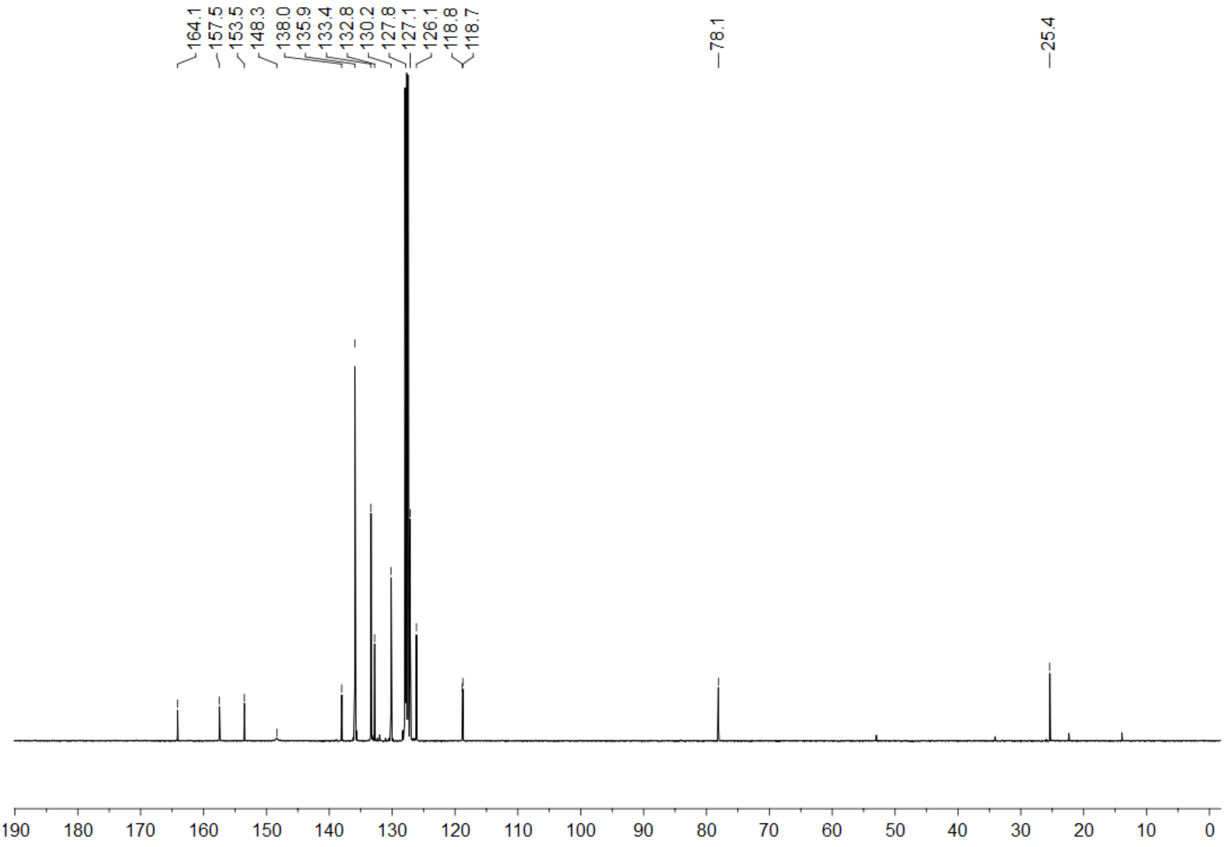

Figure S28. ^{13}C NMR spectrum (C_6D_6, 298 K) of compound **5**.

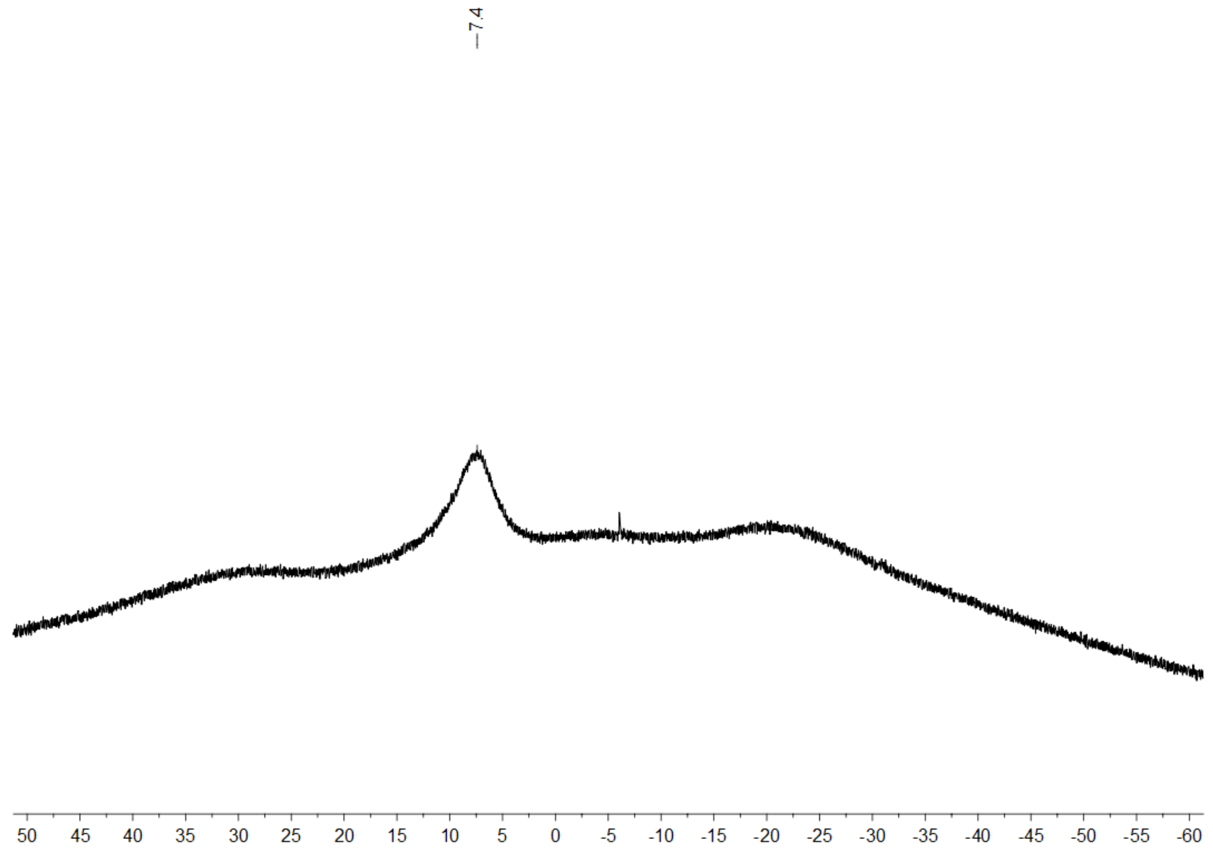

Figure S29. $^{11}B\{^1H\}$ NMR spectrum (C_6D_6, 298 K) of compound **5**.

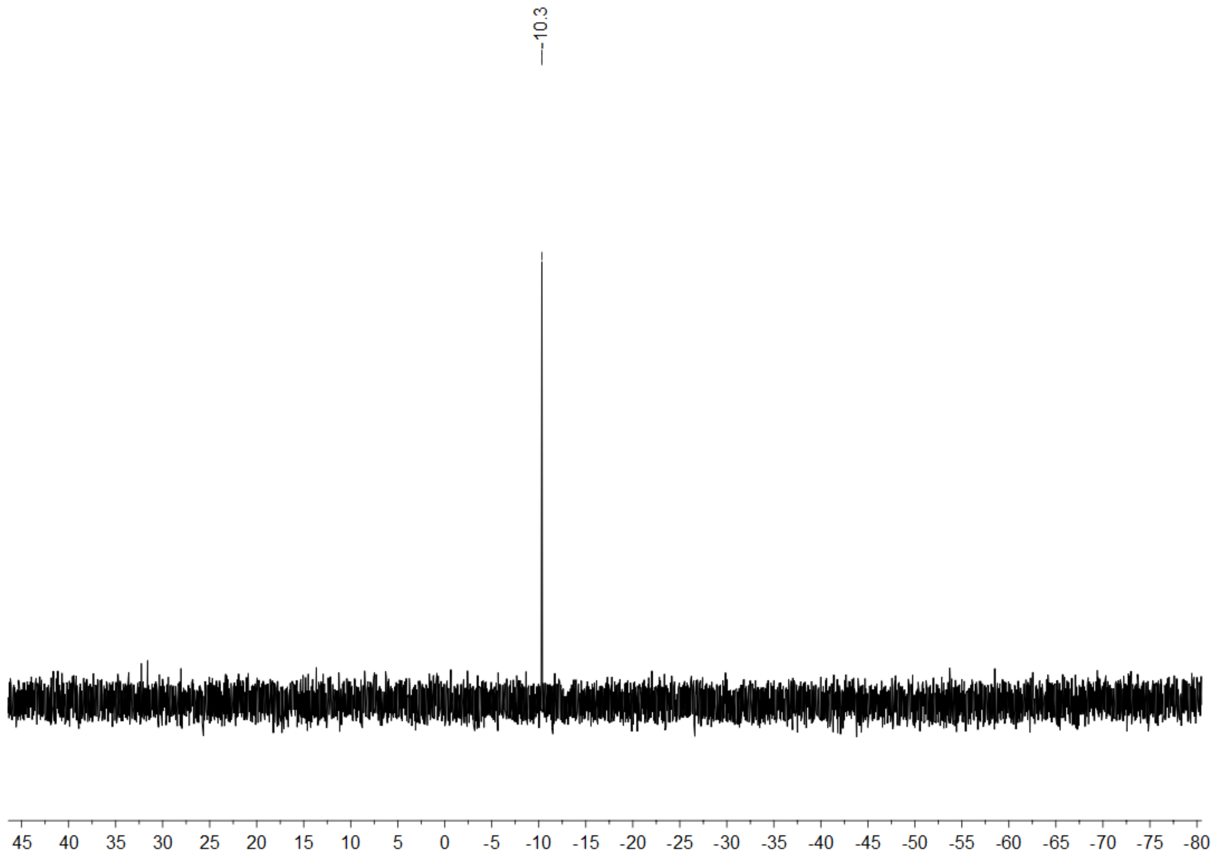

Figure S30. $^{29}Si\{^1H\}$ NMR spectrum (C_6D_6, 298 K) of compound **5**.

2.6. Synthesis of Compound 6

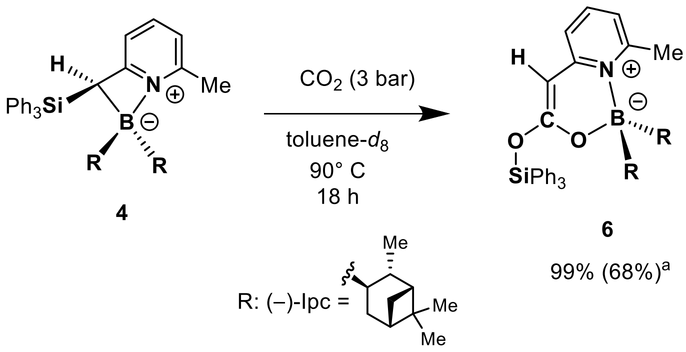

[a]Isolated yield in brackets.

Compound **4** (65 mg, 0.10 mmol) in toluene-d_8 (0.5 mL) was handled analogously in a Young-type NMR tube (see Chapter 2.5). Initial experiments under 1 bar CO_2 pressure gave unsatisfactory results (see Figure S31). After degassing and CO_2 condensation (~0.5 bar stream, ~30 s, ca. 1 cm above the frozen mixture), the pressure was adjusted to 3 bar CO_2. The tube was kept at room temperature for 16 h, showing no conversion, and then heated at 90 °C for 18 h, giving complete conversion to **6**. Slow evaporation of toluene yielded yellow crystals suitable for single-crystal X-ray diffraction analysis. Careful washing with cold pentane (2 mL, –30 °C) gave pure **6** (47 mg, 0.07 mmol, 68 %).

Spectroscopic data of the crude mixture under CO_2 atmosphere (3 bar):

^{1}H NMR (400.13 MHz, Tol-d_8, 25 °C): δ 0.64 (d, $^3J_{HH}$ = 7.1 Hz, 3H, CHC$H_{3\ Ipc}$), 0.94 (d, $^3J_{HH}$ = 7.1 Hz, 1H, CHH_{Ipc}), 1.15 (bd, 3H, $^3J_{HH}$ = 2.1 Hz, 3H, CHC$H_{3\ Ipc}$), 1.18 (bm, 4H, CHH and C$H_{3\ Ipc}$), 1.19 (bs, 3H, C$H_{3\ Ipc}$), 1.21 (bs, 1H, CHH_{Ipc}), 1.23 (2 × bs, 6H, 2 × C$H_{3\ Ipc}$), 1.42 (bm, 2H, 2 × CH_{Ipc}), 1.57 (bm, 1H, CHH_{Ipc}), 1.68 (bm, 1H, CH_{Ipc}), 1.72 – 1.81 (bm, 3H, 2 × CH and CHH_{Ipc}), 1.88 (bm, 1H, CH_{Ipc}), 1.96 (bm, 1H, CHH_{Ipc}), 2.03 (bm, 1H, CHH_{Ipc}), 2.14 (bm, 2H, CH and CHH $_{Ipc}$), 2.36 (bm, 1H, CH $_{Ipc}$), 2.47 (s, 3H, PyCH_3), 4.66 (s, 1H, C=CH), 5.92 (m, 2H, 2 × $H_{m\text{-Py}}$), 6.55 (t, $^3J_{HH}$ = 7.7 Hz, 1H, $H_{p\text{-Py}}$), 7.18 (m, 9H, H_{Ph}), 7.83 (m, 6H, H_{Ph}). **^{13}C NMR** (100.62 MHz, Tol-d_8, 25 °C): δ 23.2 (s, C$H_{3\ Ipc}$), 23.4 (s, C$H_{3\ Ipc}$), 24.1 (s, CHC$H_{3\ Ipc}$), 24.4 (s, CHC$H_{3\ Ipc}$), 24.9 (s, Py-CH_3), 28.4 (s, C$H_{2\ Ipc}$), 28.4 (s, C$H_{3\ Ipc}$), 28.8 (s, C$H_{3\ Ipc}$), 31.9 (s, C$H_{2\ Ipc}$), 32.1 (bs, CH_{Ipc}), 32.6 (s, C$H_{2\ Ipc}$), 33.2 (s, C$H_{2\ Ipc}$), 36.9 (s, CH_{Ipc}), 39.0 (s, CH_{Ipc}), 39.4 (s, CH_{Ipc}), 40.6 (s, CH_{Ipc}), 42.7 (s, CH_{Ipc}), 43.1 (s, CH_{Ipc}), 49.5 (s, CH_{Ipc}), 50.7 (s, CH_{Ipc}), 77.7 (s, C=CH), 119.0 (s, $C_{m\text{-Py}}$), 119.8 (s, $C_{m\text{-Py}}$), 125.0 (s, CO2), 128.3 (s, C_{Ph}), 130.7 (s, C_{Ph}), 133.6 (s, C_{Ph}), 136.0 (s, C_{Ph}), 136.6 (bs, C_{Ph} and $C_{p\text{-Py}}$), 153.0 (s, $C_{o\text{-Py}}$), 158.9 (s, C=CH), 164.6 (bs, $C_{o\text{-Py}}$). **^{11}B{^{1}H} NMR** (128.38 MHz, Tol-d_8, 25 °C): δ 11.6 (bs).

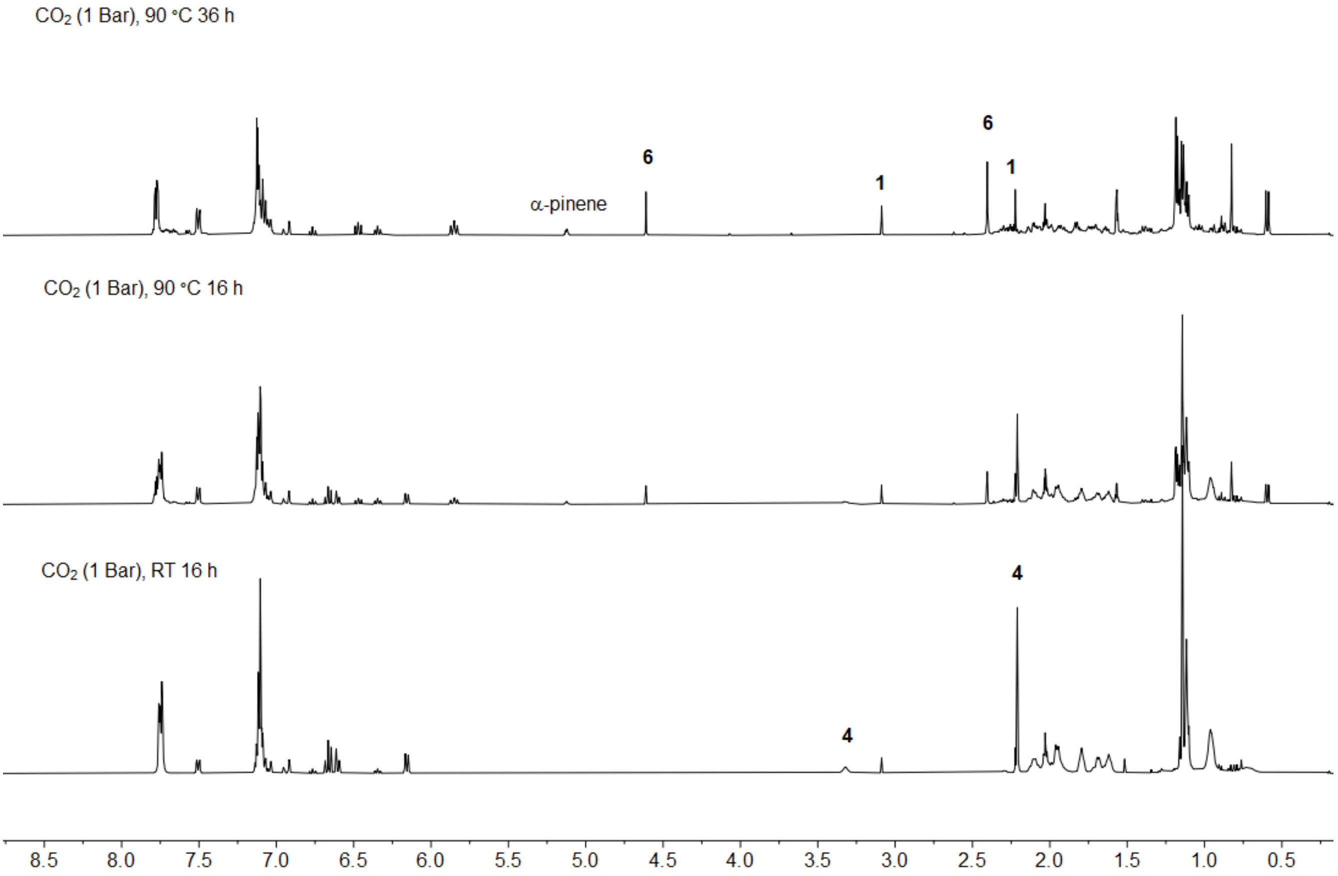

Figure S31. ^{1}H NMR spectrum (Tol-d_8, 298 K) of the crude mixture under a CO_2 atmosphere (1 bar). No CO_2 activation was observed at room temperature after 16 h (bottom). At 90 °C, 25 % conversion to compound **6** was observed after 16 h (middle), and 53% conversion after 36 h (top), with respect to an internal standard (hexamethyldisiloxane). A significantly increased amount of side products is detected, including hydrolysis to compound **1** and deborylation to α-pinene.

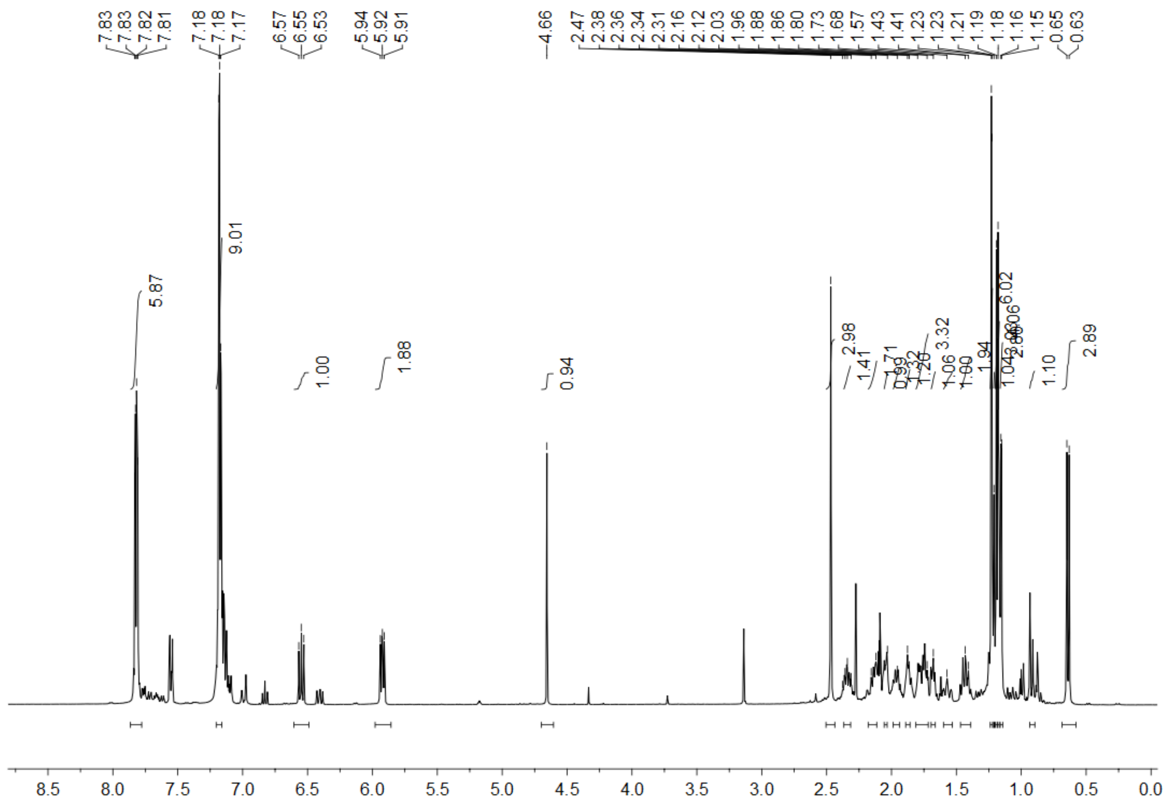

Figure S32. ^1H NMR spectrum (Tol-d_8, 298 K) of the crude mixture containing compound **6** under a CO_2 atmosphere (3 bar) after heating at 90°C for 18 h. The spectrum already indicates a very clean conversion to compound **6** (compare with Figure S36, recorded in CD_2Cl_2).

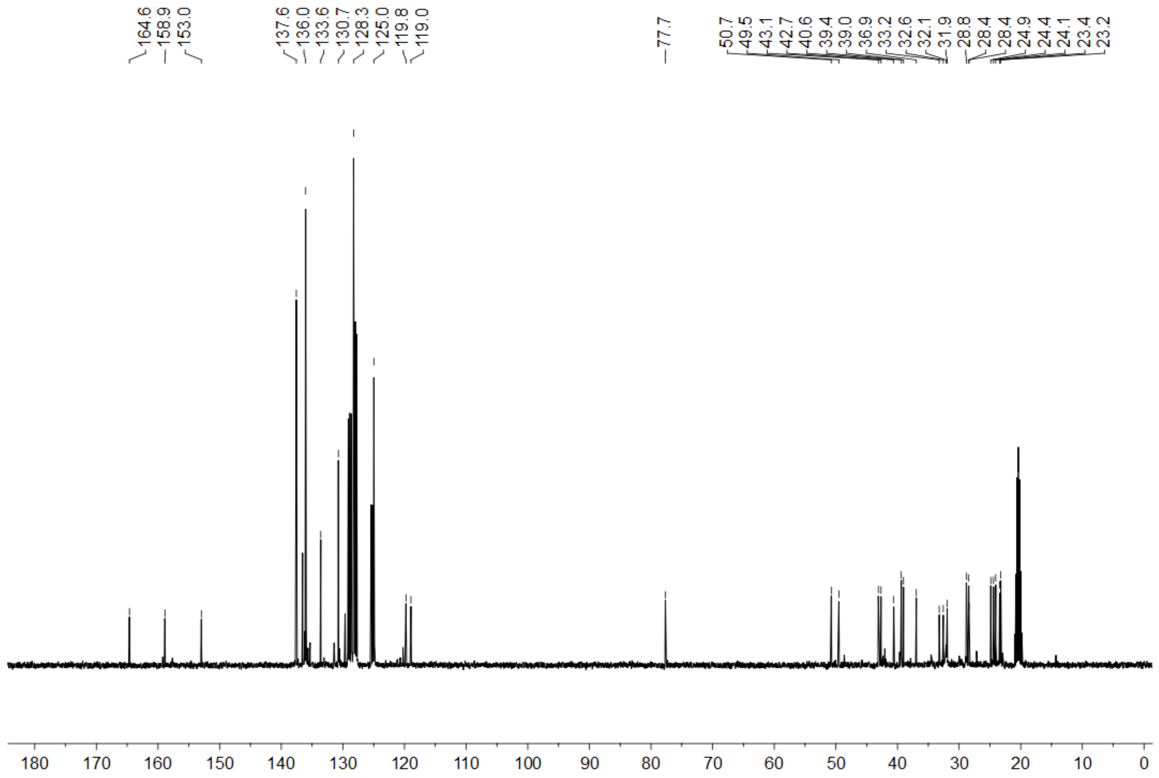

Figure S33. ^{13}C NMR spectrum (Tol-d_8, 298 K) of the crude mixture containing compound **6** under a CO_2 atmosphere (3 bar).

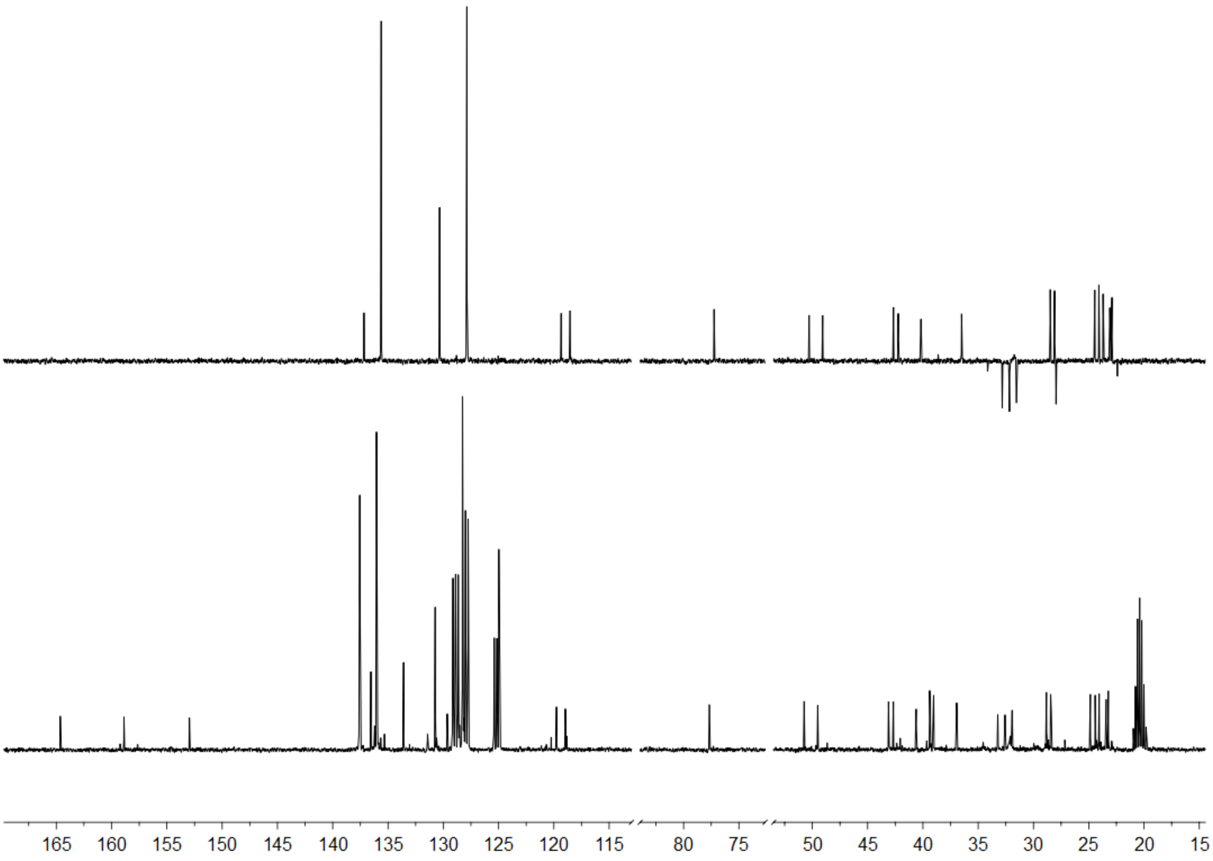

Figure S34. ^{13}C DEPT-135 (top) and ^{13}C (bottom) NMR spectra (Tol-d_8, 298 K) of the crude mixture containing compound **6** under a CO_2 atmosphere (3 bar).

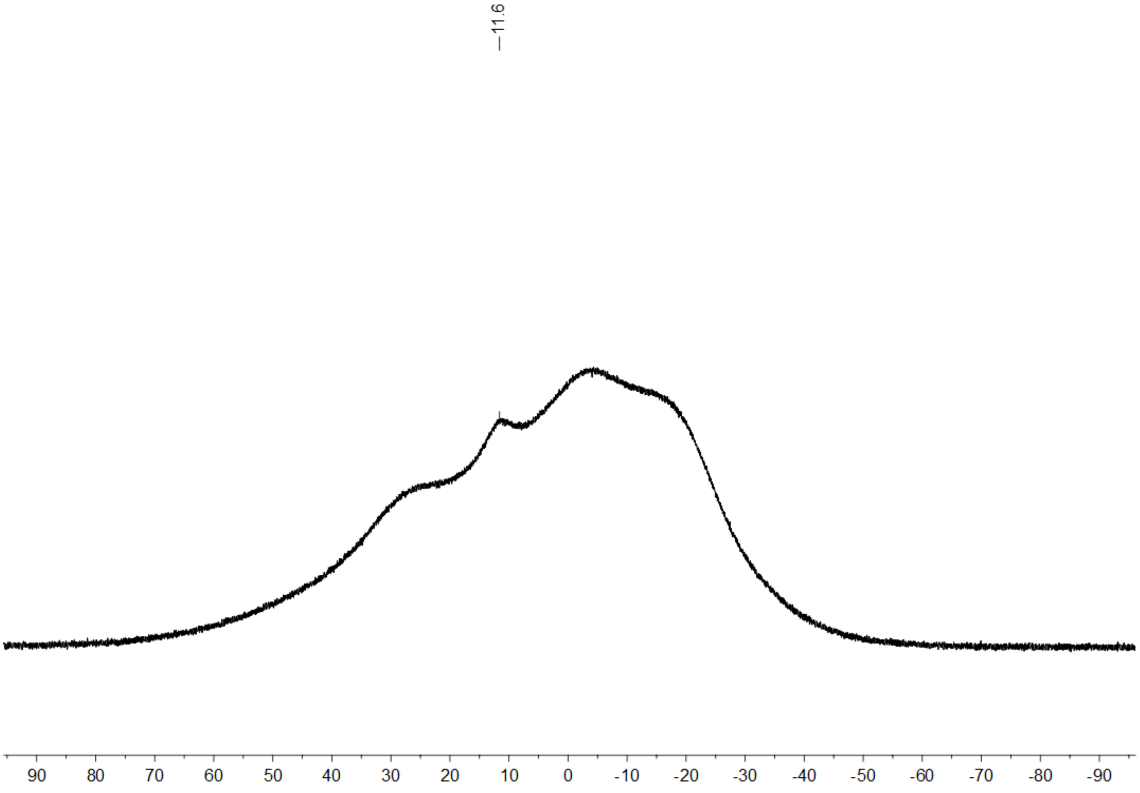

Figure S35. $^{11}B\{^1H\}$ NMR spectrum (Tol-d_8, 298 K) of the crude mixture containing compound **6** under a CO_2 atmosphere (3 bar).

Spectroscopic data of the isolated compound **6**:

^{1}H NMR (400.13 MHz, CD_2Cl_2, 25 °C): δ 0.49 (d, $^3J_{HH}$ = 7.1 Hz, 3H, $CHCH_{3\ Ipc}$), 0.68 (d, $^3J_{HH}$ = 7.1 Hz, 1H, CHH_{Ipc}), 0.92 (d, $^3J_{HH}$ = 7.1 Hz, 1H, CHH_{Ipc}), 0.94 (d, $^3J_{HH}$ = 7.1 Hz, 3H, $CHCH_{3\ Ipc}$), 1.01 (s, 3H, $CH_{3\ Ipc}$), 1.02 (s, 3H, $CH_{3\ Ipc}$), 1.08 (s, 3H, $CH_{3\ Ipc}$), 1.10 (s, 3H, $CH_{3\ Ipc}$), 1.28 – 1.36 (bm, 3H, CH_2 and CH_{Ipc}), 1.48 – 1.57 (bm, 3H, CH*H* and 2 × CH_{Ipc}), 1.60 (bm, 1H, CH_{Ipc}), 1.67 (bm, 1H, CH_{Ipc}), 1.74 – 1.82 (bm, 3H, 2 × C*H* and CHH_{Ipc}), 1.93 (bm, 2H, C*H* and CHH_{Ipc}), 2.08 (bm, 1H, CH_{Ipc}), 2.72 (s, 3H, $PyCH_3$), 4.64 (s, 1H, C=C*H*), 6.44 (bd, $^3J_{HH}$ = 7.7 Hz, 1H, $H_{m\text{-}Py}$), 6.58 (dd, $^3J_{HH}$ = 7.5 Hz, $^4J_{HH}$ = 1.0 Hz, 1H, $H_{m\text{-}Py}$), 7.28 (t, $^3J_{HH}$ = 7.7 Hz, 1H, $H_{p\text{-}Py}$), 7.40 (m, 6H, H_{Ph}), 7.47 (m, 3H, H_{Ph}), 7.72 (m, 6H, H_{Ph}). **^{13}C NMR** (100.62 MHz, CD_2Cl_2, 25 °C): δ 22.6 (s, $CH_{3\ Ipc}$), 22.8 (s, $CH_{3\ Ipc}$), 23.3 (s, $CHCH_{3\ Ipc}$), 23.6 (s, $CHCH_{3\ Ipc}$), 24.7 (s, Py-CH_3), 27.7 (s, $CH_{2\ Ipc}$), 27.9 (s, $CH_{3\ Ipc}$), 28.2 (s, $CH_{3\ Ipc}$), 31.2 (s, $CH_{2\ Ipc}$), 31.4 (bs, CH_{Ipc}), 31.9 (s, $CH_{2\ Ipc}$), 32.5 (s, $CH_{2\ Ipc}$), 36.4 (s, CH_{Ipc}), 38.5 (s, CH_{Ipc}), 38.8 (s, CH_{Ipc}), 40.1 (s, CH_{Ipc}), 42.0 (s, CH_{Ipc}), 42.5 (s, CH_{Ipc}), 48.9 (s, CH_{Ipc}), 50.1 (s, CH_{Ipc}), 76.9 (s, C=CH), 118.7 (s, $C_{m\text{-}Py}$), 119.9 (s, $C_{m\text{-}Py}$), 127.9 (s, C_{Ph}), 130.4 (s, C_{Ph}), 132.9 (s, C_{Ph}), 135.4 (s, C_{Ph}), 137.8 (s, $C_{p\text{-}Py}$), 152.5 (bs, $C_{o\text{-}Py}$), 158.2 (s, *C*=CH), 163.8 (bs, $C_{o\text{-}Py}$). **^{11}B{^{1}H}** NMR (128.38 MHz, CD_2Cl_2, 25 °C): δ 11.6 (bs). **^{29}Si{^{1}H} NMR** (79.49 MHz, Tol-d_8, 25 °C): δ – 14.6 (s). **HRMS (EI$^+$)**, calcd. m/z for {[(M + H) – $BIpc_2$]$^+$}: 366.1673; found 366.1677. **Elemental analyis**: calcd. for $C_{46}H_{56}BNO_2Si$: C 79.63, H 8.14, N 2.02; found: C 79.34, H 7.95, N 1.83.

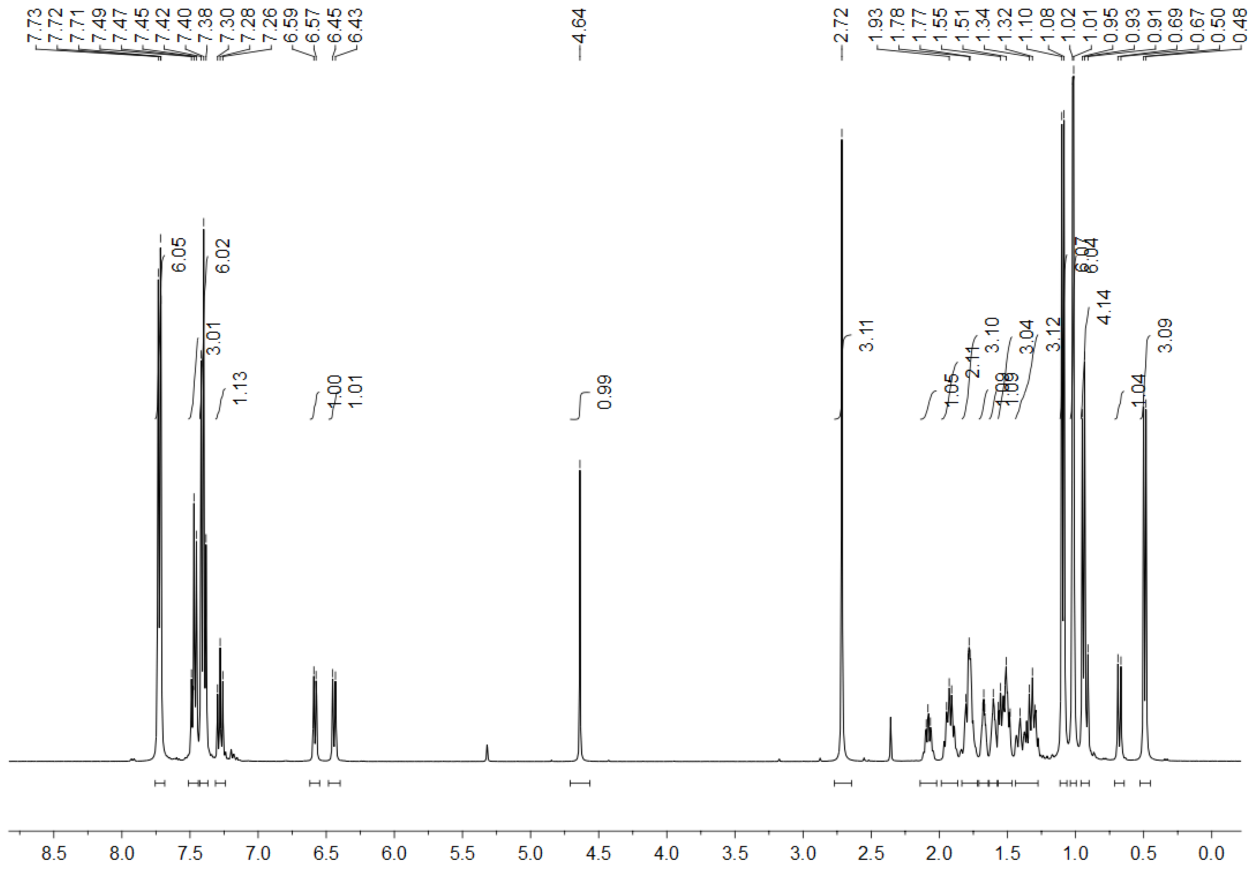

Figure S36. ^{1}H NMR spectrum (CD_2Cl_2, 298 K) of compound **6**.

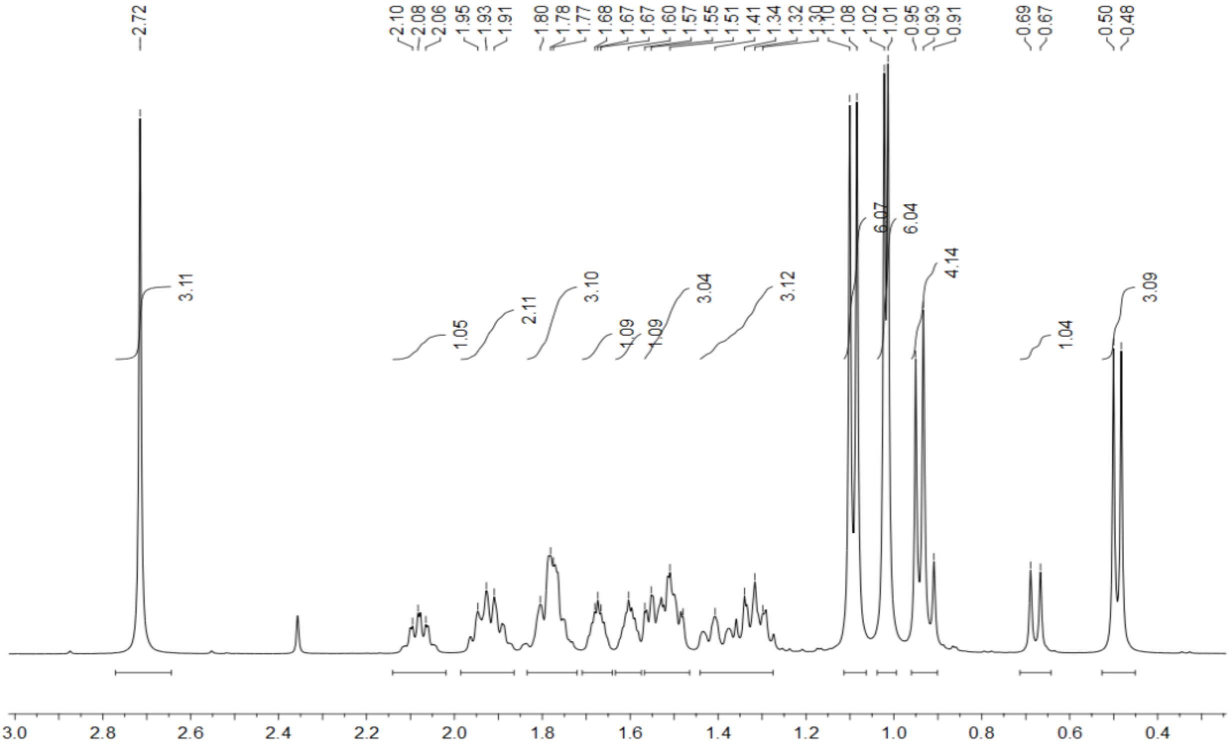

Figure S37. Zoomed aliphatic area (^{1}H NMR, CD_2Cl_2, 298 K) of compound **6**.

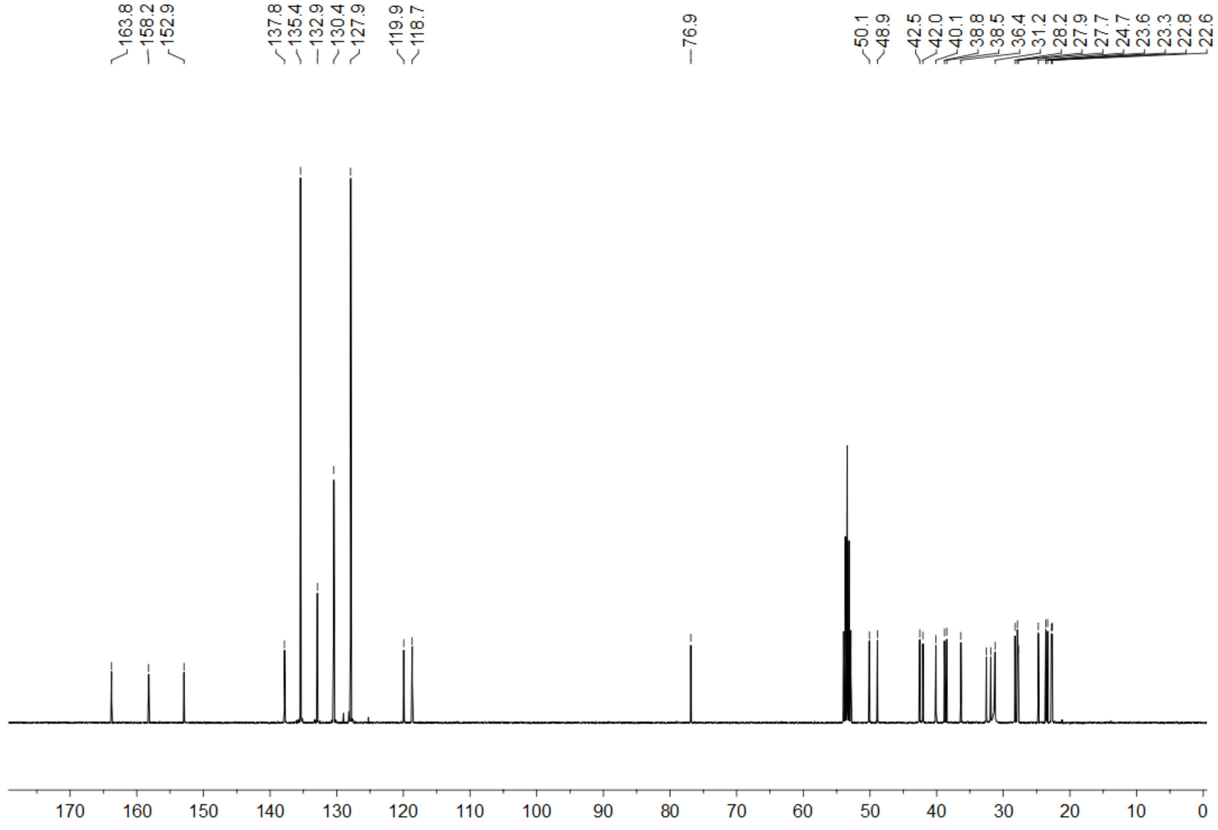

Figure S38. ^{13}C NMR spectrum (CD_2Cl_2, 298 K) of compound **6**.

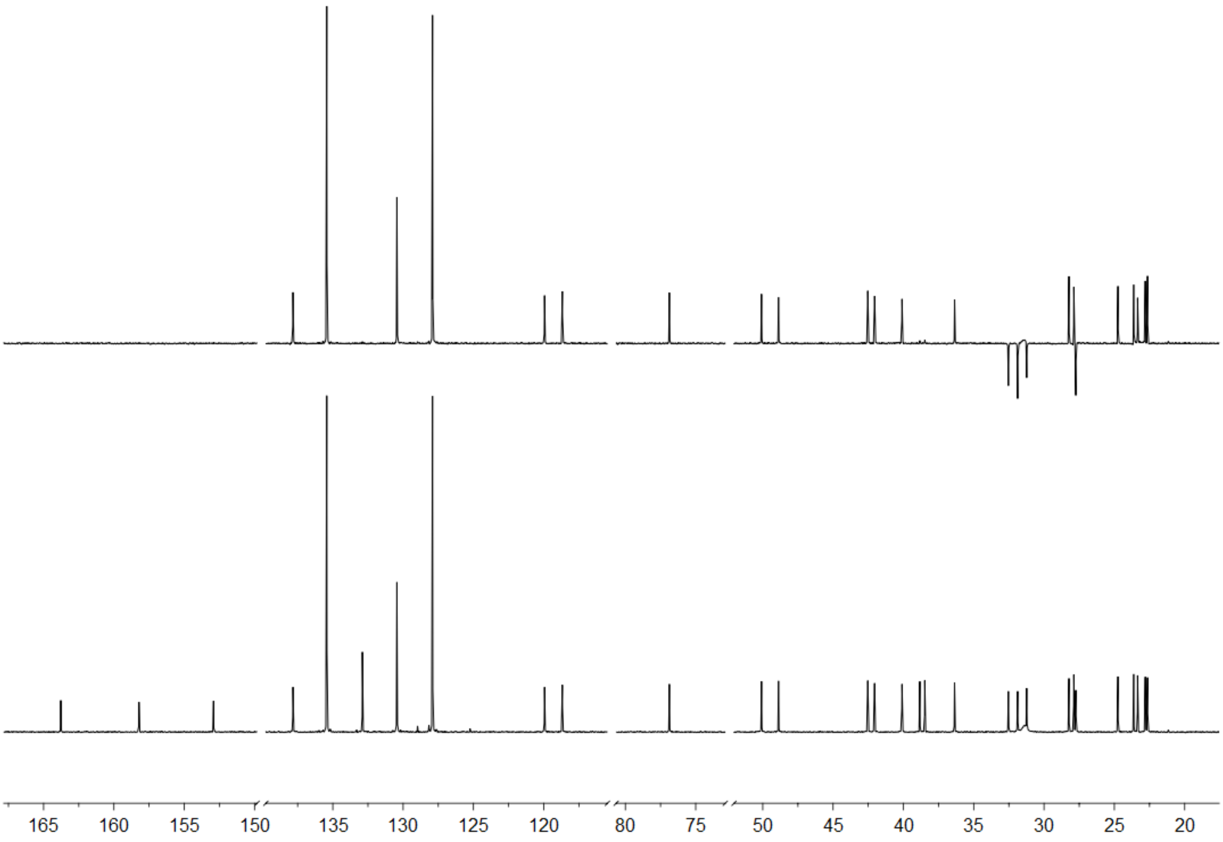

Figure S39. ^{13}C DEPT-135 (top) and ^{13}C (bottom) NMR spectra (CD_2Cl_2, 298 K), relevant for the unambiguous determination of the signals of compound **6**.

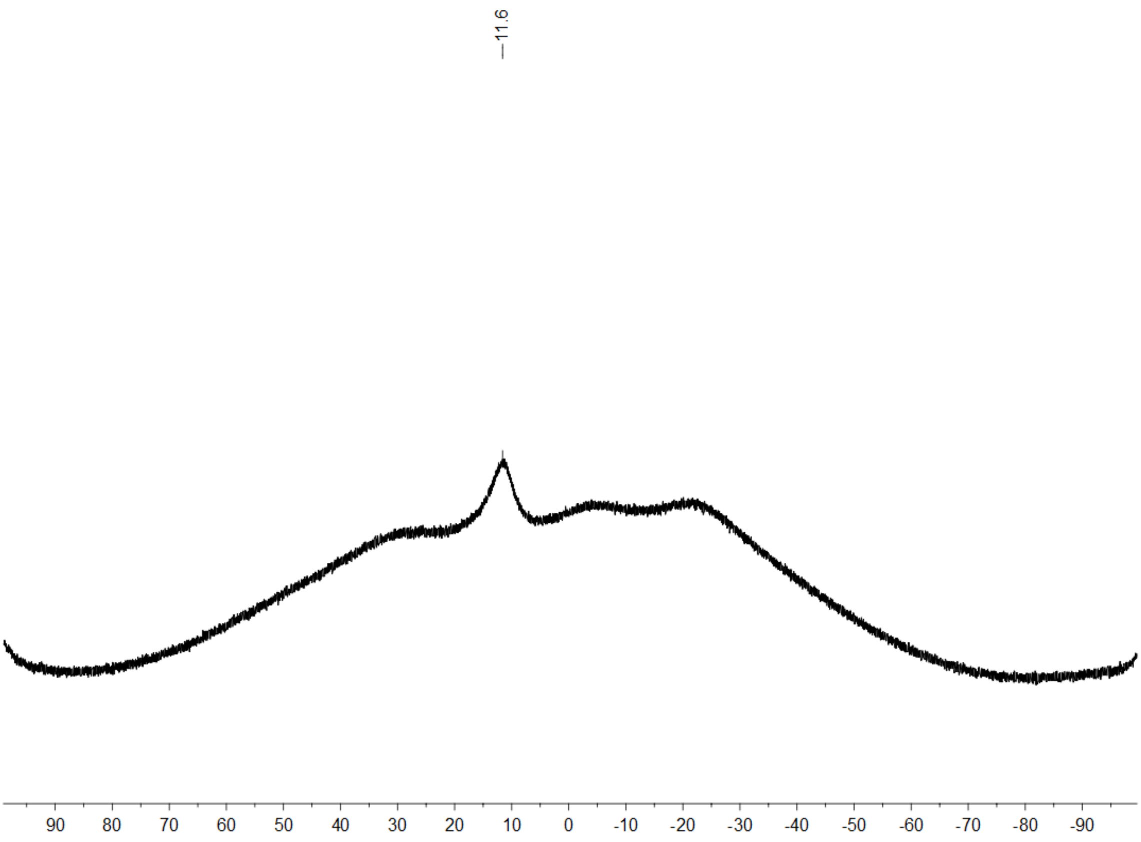

Figure S40. $^{11}B\{^{1}H\}$ NMR spectrum (CD_2Cl_2, 298 K) of compound **6**.

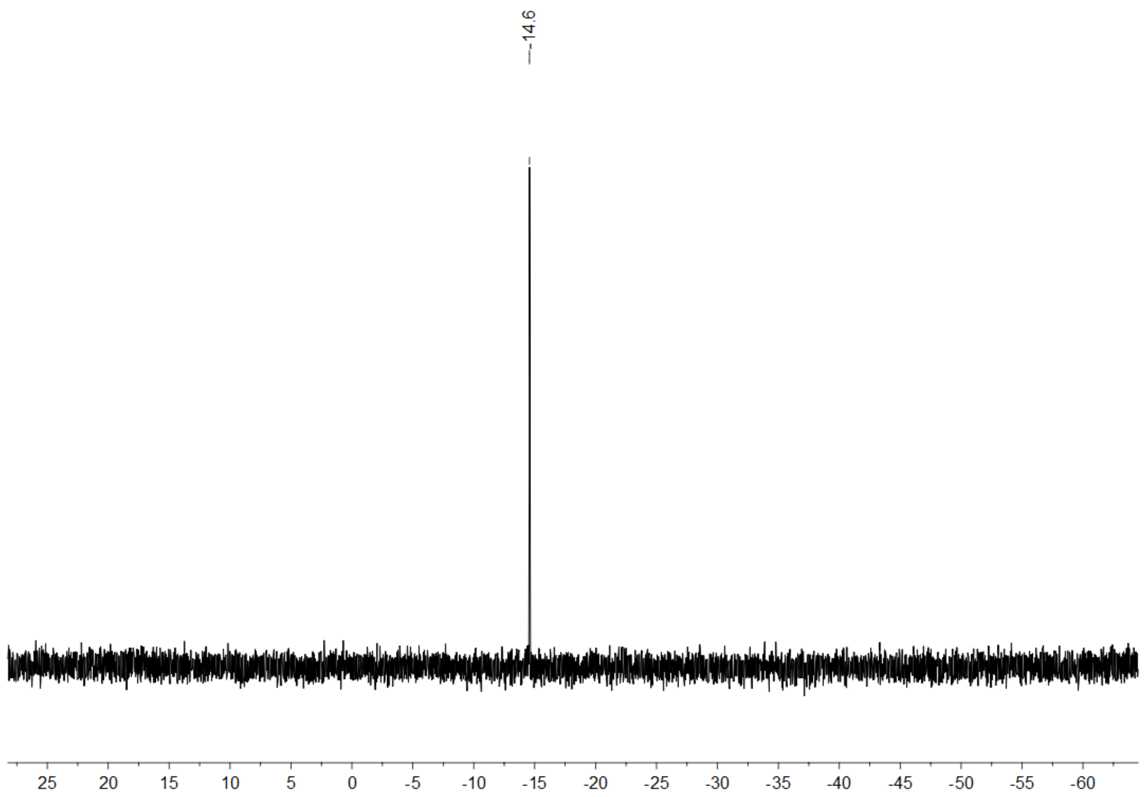

Figure S41. $^{29}Si\{^{1}H\}$ NMR spectrum (Tol-d_8, 298 K) of compound **6**.

2.7. Synthesis of Compound 9

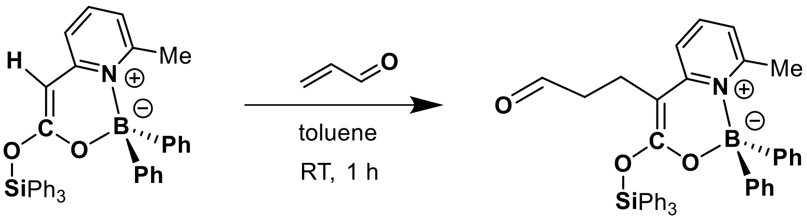

5

9

99% (73%)[a]

[a]Isolated yield in brackets

Compound **5** (86 mg, 0.15 mmol) in toluene-d_8 (0.5 mL) was treated with acrolein (10 µL, 0.15 mmol) in a Young-type NMR tube under nitrogen. Immediate color change from pale orange to glowing yellow-green was observed, and NMR confirmed full consumption of acrolein with clean formation of **9**. Due to slow decomposition at room temperature, isolation was attempted on a larger scale. Compound **5** (872 mg, 1.52 mmol) in toluene (5 mL) was reacted with acrolein (102 µL, 1.52 mmol) under nitrogen. The mixture was layered with hexane and stored at –30 °C, affording yellow crystals of **9** within one week. Single-crystal X-ray diffraction analysis confirmed the structure. After filtration, the solids were washed with cold hexane (3 × 2 mL, –20 °C) and dried under reduced pressure to yield compound **9** (670 mg, 1.11 mmol, 73 %).

Spectroscopic data of the crude mixture:

^{1}H NMR (500.18 MHz, Tol-d_8, 25 °C): δ 1.72 (s, 3H, CH_3), 2.10 (t, $^3J_{HH}$ = 7.9 Hz, 2H, CH_2), 2.71 (t, $^3J_{HH}$ = 7.9 Hz, 2H, CH_2), 5.93 (d, $^3J_{HH}$ = 7.4 Hz, 1H, $H_{m\text{-Py}}$), 6.54 (d, $^3J_{HH}$ = 8.5 Hz, 1H, $H_{m\text{-Py}}$), 6.80 (t, $^3J_{HH}$ = 8.5 Hz, 1H, $H_{p\text{-Py}}$), 7.04 (m, 4H, H_{Ph}), 7.14 (m, 11H, H_{Ph}), 7.21 (m, 4H, H_{Ph}), 7.67 (m, 6H, H_{Ph}), 9.27 (s, 1H, O=CH). **^{13}C NMR** (125.78 MHz, Tol-d_8, 25 °C): δ 18.3 (s, CH$_2$), 26.0 (s, CH$_3$), 42.8 (s, CH$_2$), 85.1 (s, C=C), 115.5 (s, $C_{m\text{-Py}}$), 119.1 (s, $C_{m\text{-Py}}$), 126.1 (s, C_{Ph}), 127.0 (s, C_{Ph}), 127.9 (s, C_{Ph}), 130.2 (s, C_{Ph}), 132.8 (s, $C_{p\text{-Py}}$), 133.3 (s, C_{Ph}), 135.9 (s, C_{Ph}), 136.2 (s, C_{Ph}), 138.5 (s, C_{Ph}), 148.4 (bs, C_{Ph}), 154.5 (s, $C_{o\text{-Py}}$), 156.3 (s, C=C), 161.4 (s, $C_{o\text{-Py}}$), 200.2 (s, O=CH). **^{11}B{^{1}H} NMR** (160.47 MHz, Tol-d_8, 25 °C): δ 6.4 (bs).

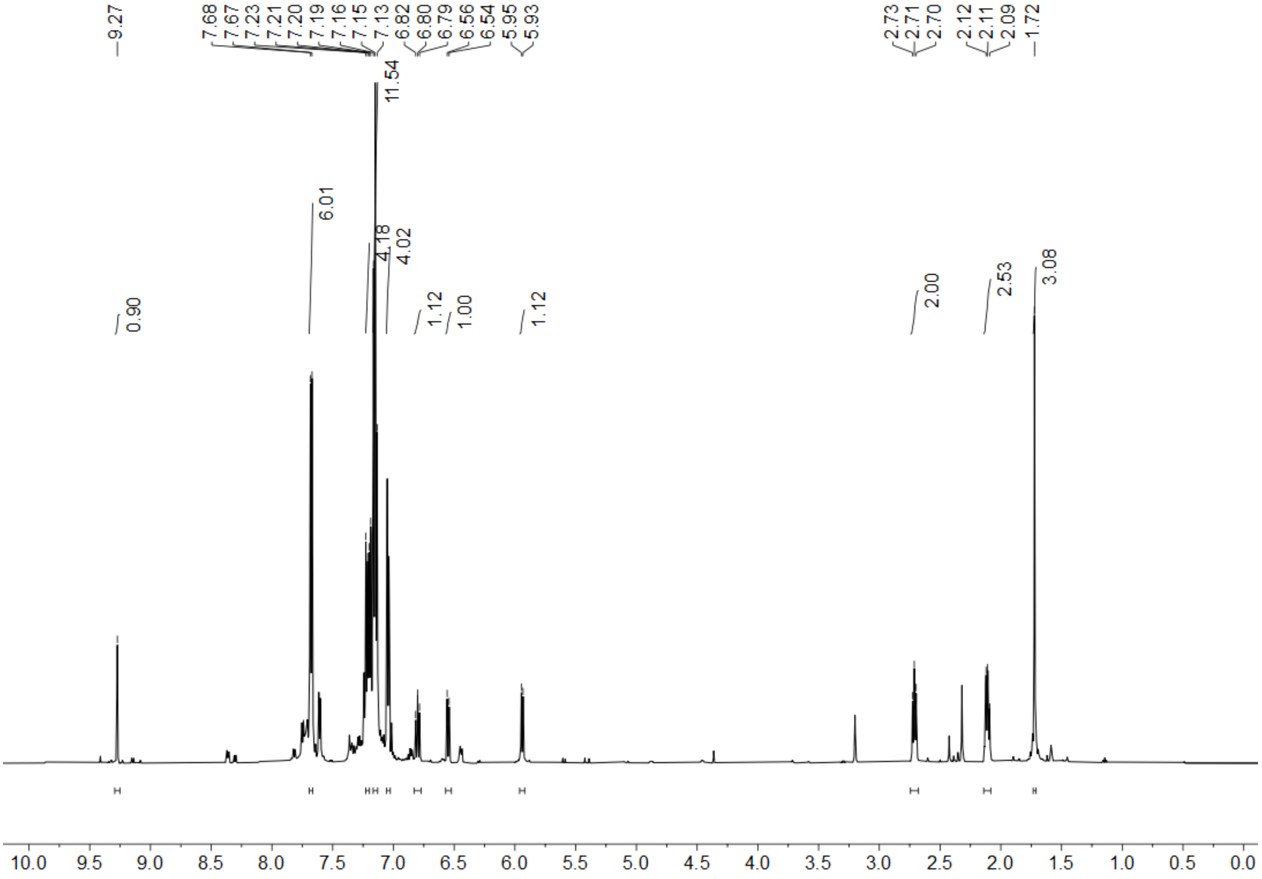

Figure S42. ^{1}H NMR spectrum (Tol-d^8, 298 K) of the crude mixture containing compound **9**.

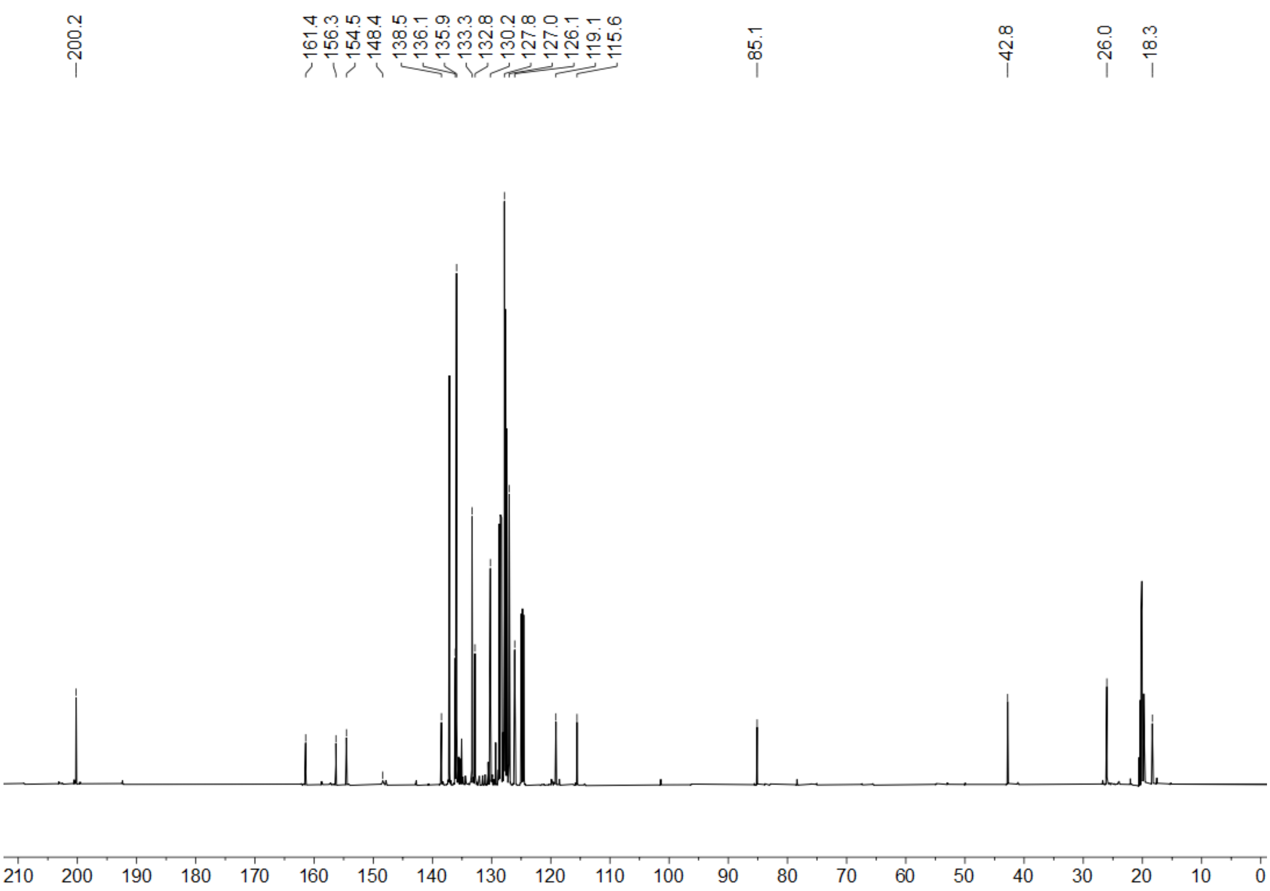

Figure S43. ^{13}C NMR spectrum (Tol-d^8, 298 K) of the crude mixture containing compound **9**.

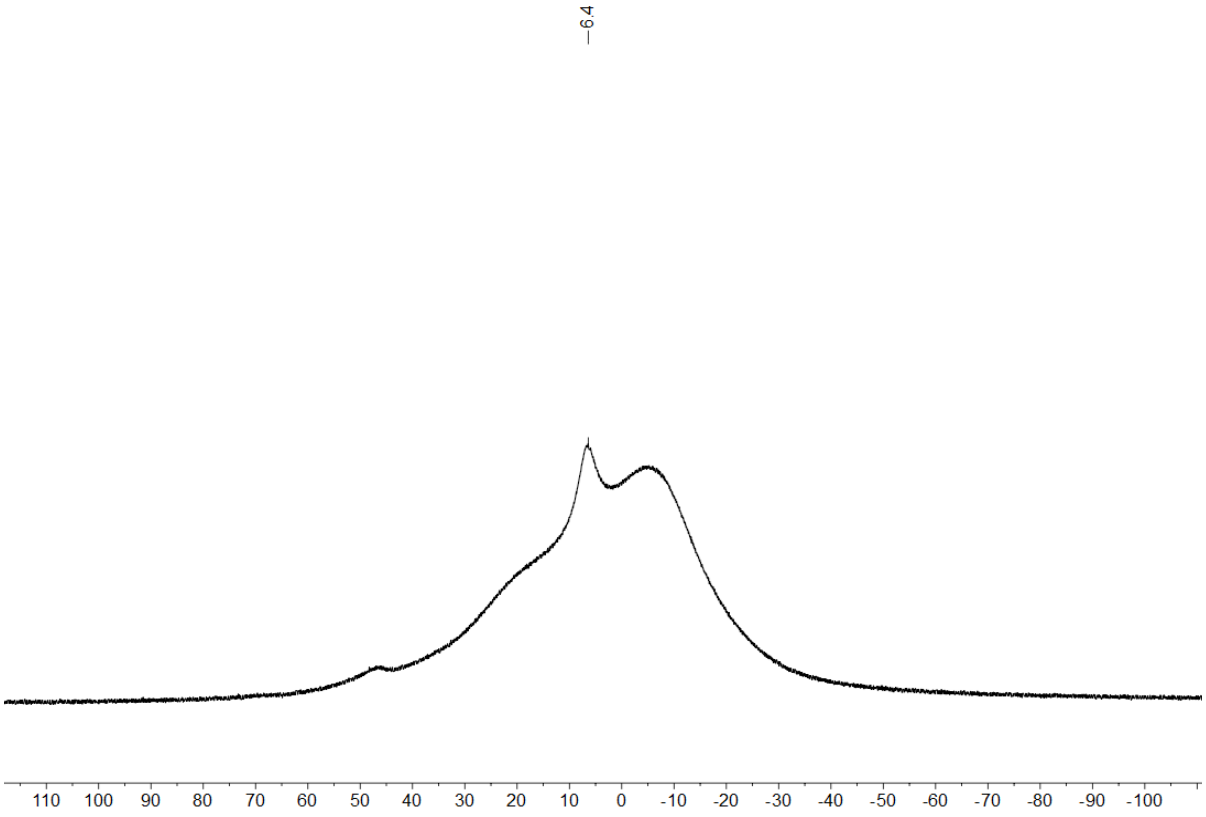

Figure S44. $^{11}B\{^1H\}$ NMR spectrum (Tol-d^8, 298 K) of the crude mixture containing compound **9**.

Spectroscopic data of the isolated compound **9**:

^{1}H NMR (500.18 MHz, C_6D_6, 25 °C): δ 1.68 (s, 3H, C*H*$_3$), 2.03 (t, $^3J_{HH}$ = 7.8 Hz, 2H, C*H*$_2$), 2.67 (t, $^3J_{HH}$ = 7.8 Hz, 2H, C*H*$_2$), 5.82 (d, $^3J_{HH}$ = 7.4 Hz, 1H, $H_{m\text{-Py}}$), 6.44 (d, $^3J_{HH}$ = 8.5 Hz, 1H, $H_{m\text{-Py}}$), 6.66 (t, $^3J_{HH}$ = 7.7 Hz, 1H, $H_{p\text{-Py}}$), 7.07 (m, 4H, H_{Ph}), 7.11 (m, 7H, H_{Ph}), 7.14 – 7.22 (m, 8H, H_{Ph}), 7.68 (m, 6H, H_{Ph}), 9.24 (s, 1H, O=C*H*). **^{13}C NMR** (125.78 MHz, C_6D_6, 25 °C): δ 18.3 (s, *C*H$_2$), 26.1 (s, *C*H$_3$), 42.7 (s, *C*H$_2$), 85.1 (s, C=*C*), 115.6 (s, $C_{m\text{-Py}}$), 119.1 (s, $C_{m\text{-Py}}$), 126.1 (s, C_{Ph}), 127.1 (s, C_{Ph}), 127.9 (s, C_{Ph}), 130.3 (s, C_{Ph}), 132.8 (s, $C_{p\text{-Py}}$), 133.3 (s, C_{Ph}), 136.0 (s, C_{Ph}), 138.4 (s, C_{Ph}), 148.4 (bs, C_{Ph}), 154.5 (s, $C_{o\text{-Py}}$), 156.3 (s, *C*=C), 161.4 (s, $C_{o\text{-Py}}$), 200.5 (s, O=*C*H). **^{11}B{^{1}H} NMR** (160.47 MHz, C_6D_6, 25 °C): δ 6.5 (bs). **^{29}Si{^{1}H} NMR** (79.49 MHz, C_6D_6, 25 °C): δ – 10.6 (s). **HRMS (FD$^+$)**, calcd. m/z for [M$^+$]: 629.25520; found 629.25624. **Elemental analysis**: calcd. for $C_{41}H_{36}BNO_3Si$: C 78.21, H 5.76, N 2.22; found: C 77.91, H 5.85, N 2.14.

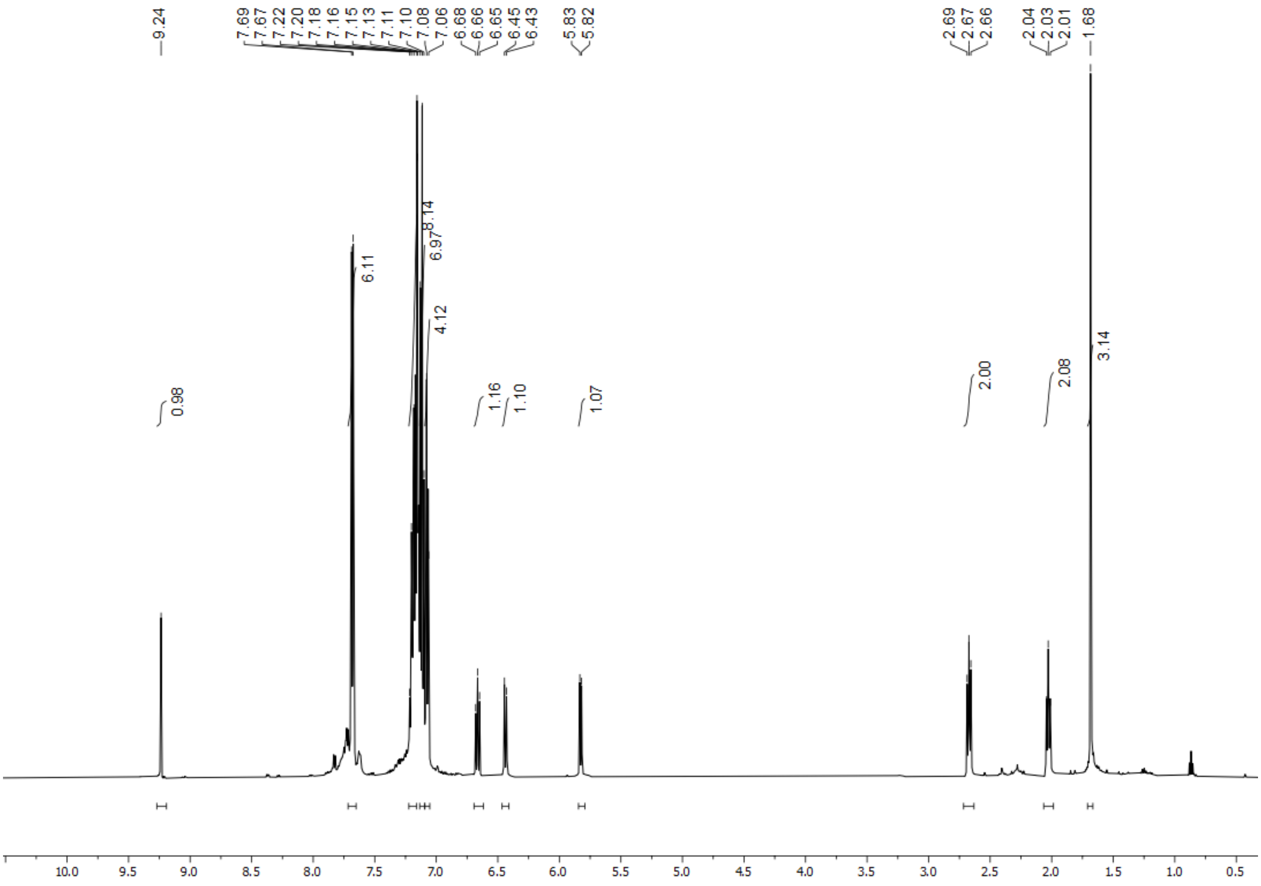

Figure S45. ^{1}H NMR spectrum (C_6D_6, 298 K) of compound **9**.

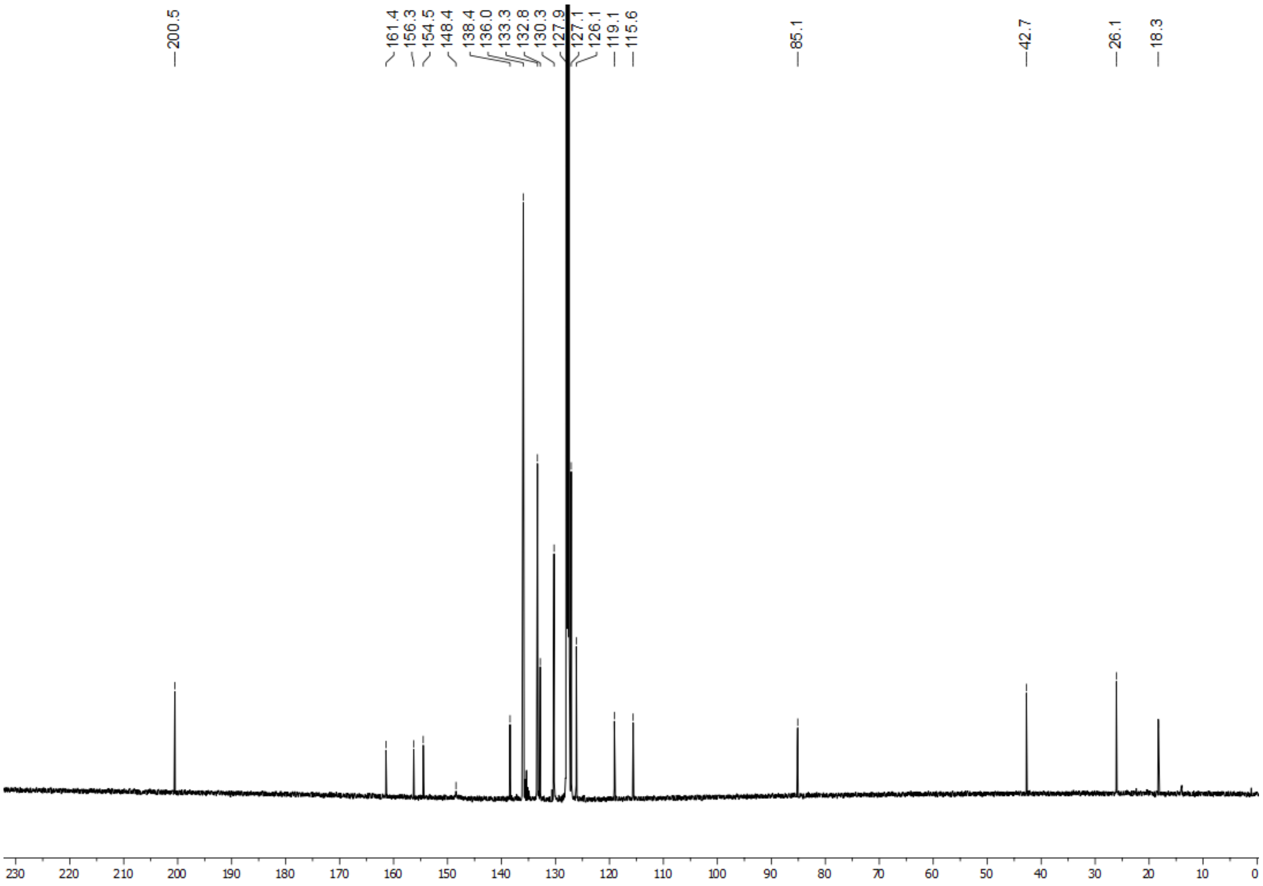

Figure S46. ^{13}C NMR spectrum (C_6D_6, 298 K) of compound **9**.

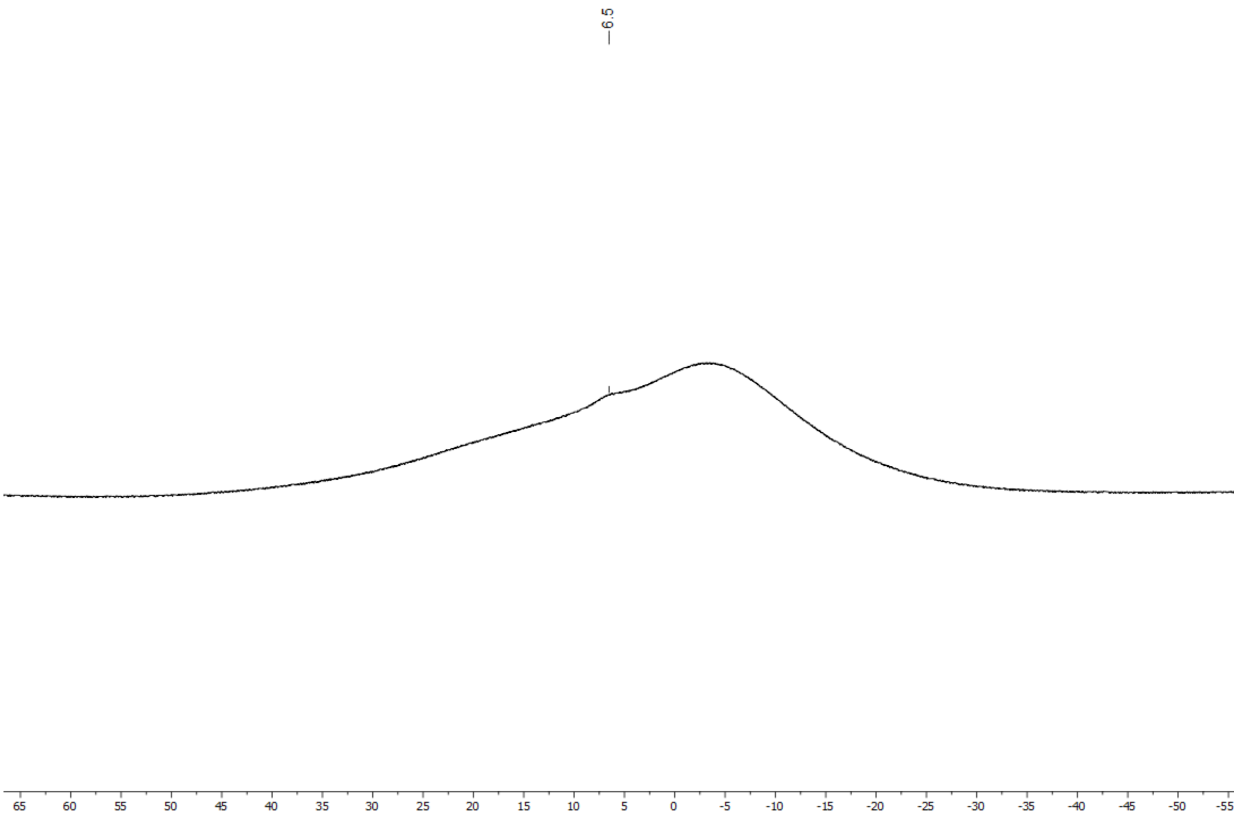

Figure S47. $^{11}B\{^1H\}$ NMR spectrum (C_6D_6, 298 K) of compound **9**.

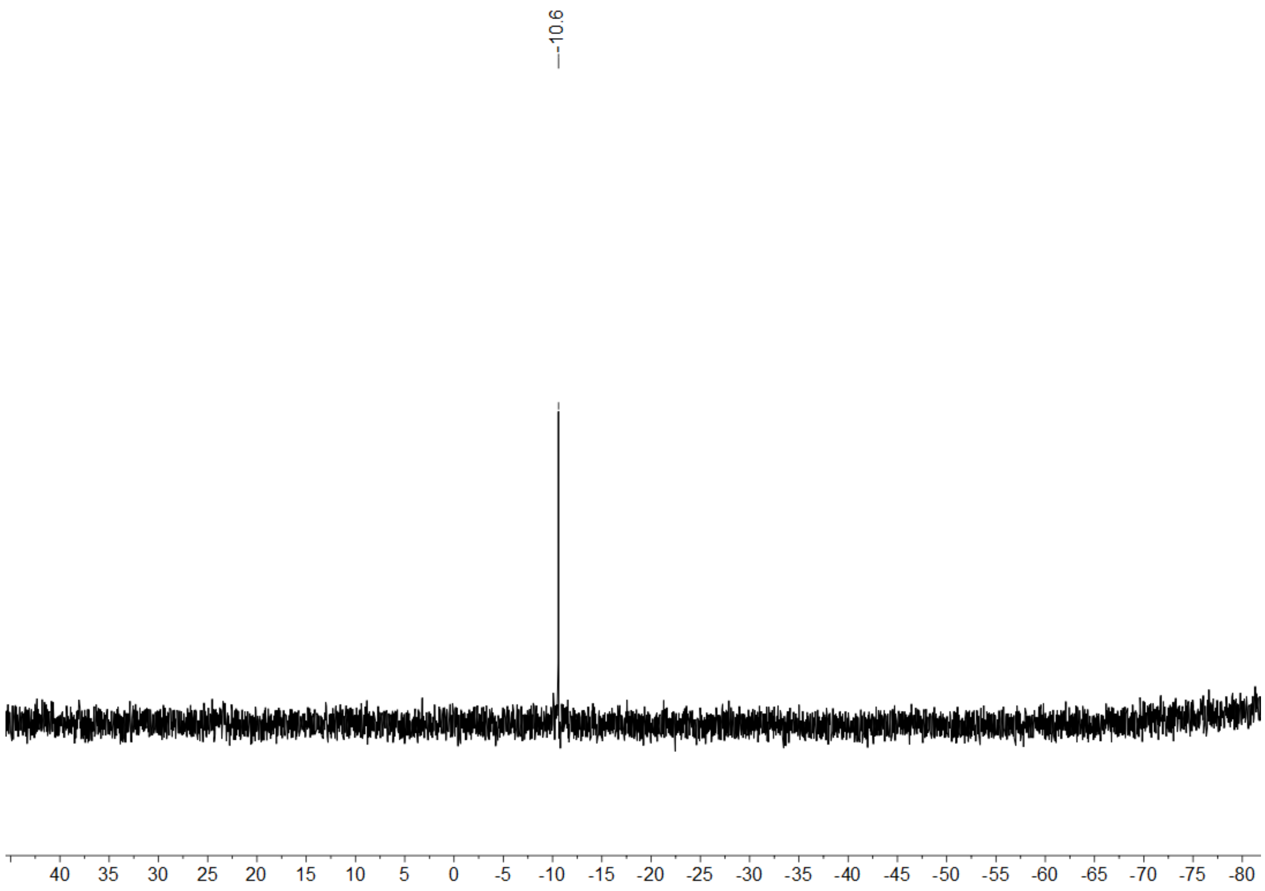

Figure S48. $^{29}Si\{^1H\}$ NMR spectrum (C_6D_6, 298 K) of compound **9**.

2.8. Synthesis of Compounds *Z*/*E*-11

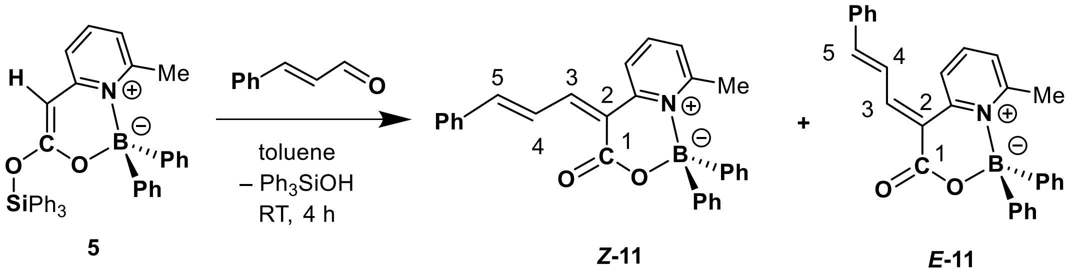

^{1}H NMR ratio 1.00 : 0.54 (***Z*-11** : ***E*-11**)

Compound **5** (86 mg, 0.15 mmol) in toluene-d_8 (0.5 mL) was treated with cinnamaldehyde (19 µL, 0.15 mmol) under nitrogen. No immediate reaction was observed by NMR, but after standing overnight, crystalline material was formed. Single-crystal X-ray diffraction analysis confirmed the *Z*-isomer. NMR analysis showed a ***Z*/*E*** mixture (1.00 : 0.54). Scale-up with compound **5** (918 mg, 1.60 mmol) and cinnamaldehyde (200 µL, 1.60 mmol) in toluene (5 mL) gave yellow crystals overnight (247 mg, 0.57 mmol, 36 %). Further precipitation from the mother liquor afforded additional ***Z*/*E*-11**. No change of the isomeric ratio was observed. The combined fractions gave compound ***Z*/*E*-11** as a yellow solid material in 58 % (398 mg, 0.93 mmol).

NMR spectroscopic data of isomer ***Z*-11** (*major*): **^{1}H NMR** (500.18 MHz, THF-d_8, 25 °C): δ 2.11 (s, 3H, CH_3), 6.95 (d, $^{3}J_{HH}$ = 15.5 Hz 1H, C=CH^5), 7.05 (d, $^{3}J_{HH}$ = 11.2 Hz 1H, C=CH^3), 7.10 – 7.21 (m, 10H, H_{Ph}), 7.28 – 7.37 (m, 3H, H_{Ph}), 7.45 – 7.55 (m, 3H, $H_{m\text{-}Py}$, H_{Ph}), 7.91 (bd, 1H $^{3}J_{HH}$ = 7.9 Hz, $H_{m\text{-}Py}$), 8.15 (t, $^{3}J_{HH}$ = 7.9 Hz, 1H, $H_{p\text{-}Py}$), 8.27 (dd, $^{3}J_{HH}$ = 15.6 Hz, $^{3}J_{HH}$ = 11.2 Hz 1H, C=CH^4). **^{13}C NMR** (125.78 MHz, THF-d_8, 25 °C): δ 25.2 (s, CH$_3$), 122.1 (s, $C_{m\text{-}Py}$), 126.0 (s, C=CH^4), 126.2 (s, $C_{m\text{-}Py}$), 126.9 (s, C_{Ph}), 127.1 (s, C_{Ph}), 127.5 (s, C_{Ph}), 127.6 (s, C_{Ph}), 128.6 (s, C_{Ph}), 129.2 (s, C_{Ph}), 129.3 (s, C_{Ph}), 132.7 (s, C_{Ph}), 132.7 (s, C_{Ph}), 132.8 (s, C_{Ph}), 136.4 (s, HC=C^2), 141.4 (s, $C_{p\text{-}Py}$), 143.8 [s, C=CH^5), 145.1 (s, C=CH^3), 148.3 (bs, C_{Ph}), 155.6 (s, $C_{o\text{-}Py}$), 156.7 (s, C=O), 163.4 (s, $C_{o\text{-}Py}$). **^{11}B{^{1}H} NMR** (160.47 MHz, THF-d_8, 25 °C): δ 6.9 (bs).

NMR spectroscopic data of isomer ***E*-11** (*minor*): **^{1}H NMR** (500.18 MHz, THF-d_8, 25 °C): δ 2.10 (s, 3H, CH_3), 7.04 (d, $^{3}J_{HH}$ = 11.2 Hz 1H, C=CH^3), 7.10 – 7.21 (m, 11H, C=CH^5, H_{Ph}), 7.28 – 7.37 (m, 3H, H_{Ph}) 7.45 – 7.55 (m, 4H, C=CH^4, $H_{m\text{-}Py}$, H_{Ph}), 7.85 (bd, 1H $^{3}J_{HH}$ = 7.9 Hz, $H_{m\text{-}Py}$), 8.22 (t, $^{3}J_{HH}$ = 7.9 Hz, 1H, $H_{p\text{-}Py}$). **^{13}C NMR** (125.78 MHz, THF-d_8, 25 °C): δ 25.0 (s, CH$_3$), 122.2 (s, C=CH^4), 125.5 (s, $C_{m\text{-}Py}$), 126.0 (s, $C_{m\text{-}Py}$), 126.9 (s, C_{Ph}), 127.1 (s, C_{Ph}), 127.5 (s, C_{Ph}), 127.6 (s, C_{Ph}), 128.6 (s, C_{Ph}), 129.2 (s, C_{Ph}), 129.3 (s, C_{Ph}), 132.7 (s, C_{Ph}), 132.7 (s, C_{Ph}), 132.8 (s, C_{Ph}), 136.2 (s, HC=C^2), 140.9 (s, $C_{p\text{-}Py}$), 141.9 (s, C=CH^5), 144.8 (s, C=CH^3), 148.3 (bs, C_{Ph}), 151.9 (s, $C_{o\text{-}Py}$), 158.0 (s, C=O), 165.3 (s, $C_{o\text{-}Py}$). **^{11}B{^{1}H} NMR** (160.47 MHz, THF-d_8, 25 °C): δ 6.9 (bs).

HRMS (FD$^+$), calcd. m/z for [M$^+$]: 429.18946; found 429.19014. **Elemental analysis**: calcd. for $C_{29}H_{24}BNO_2$: C 81.13, H 5.63, N 3.26; found: C 80.72, H 5.85, N 2.98.

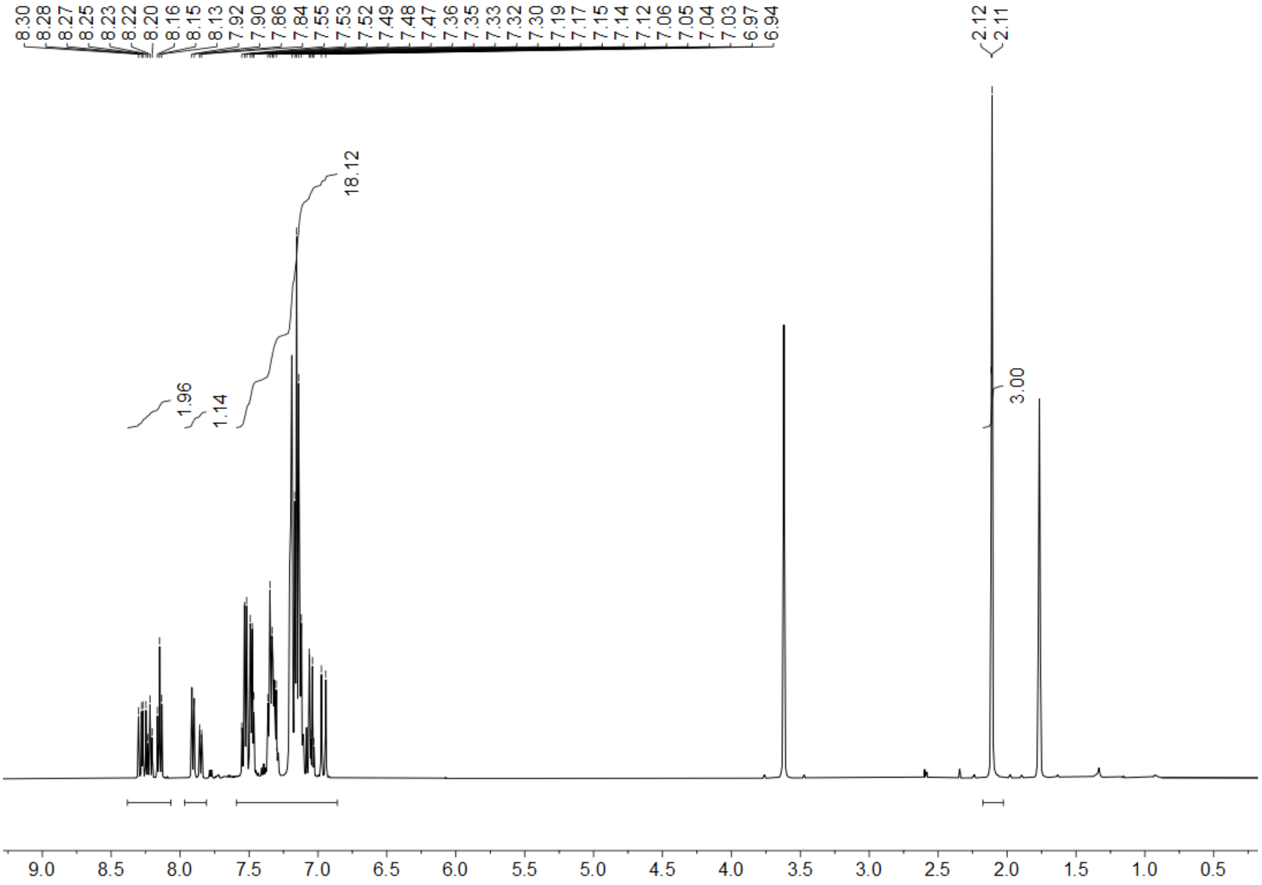

Figure S49. ^{1}H NMR spectrum (THF-d_8, 298 K) of ***Z*/*E*-11**.

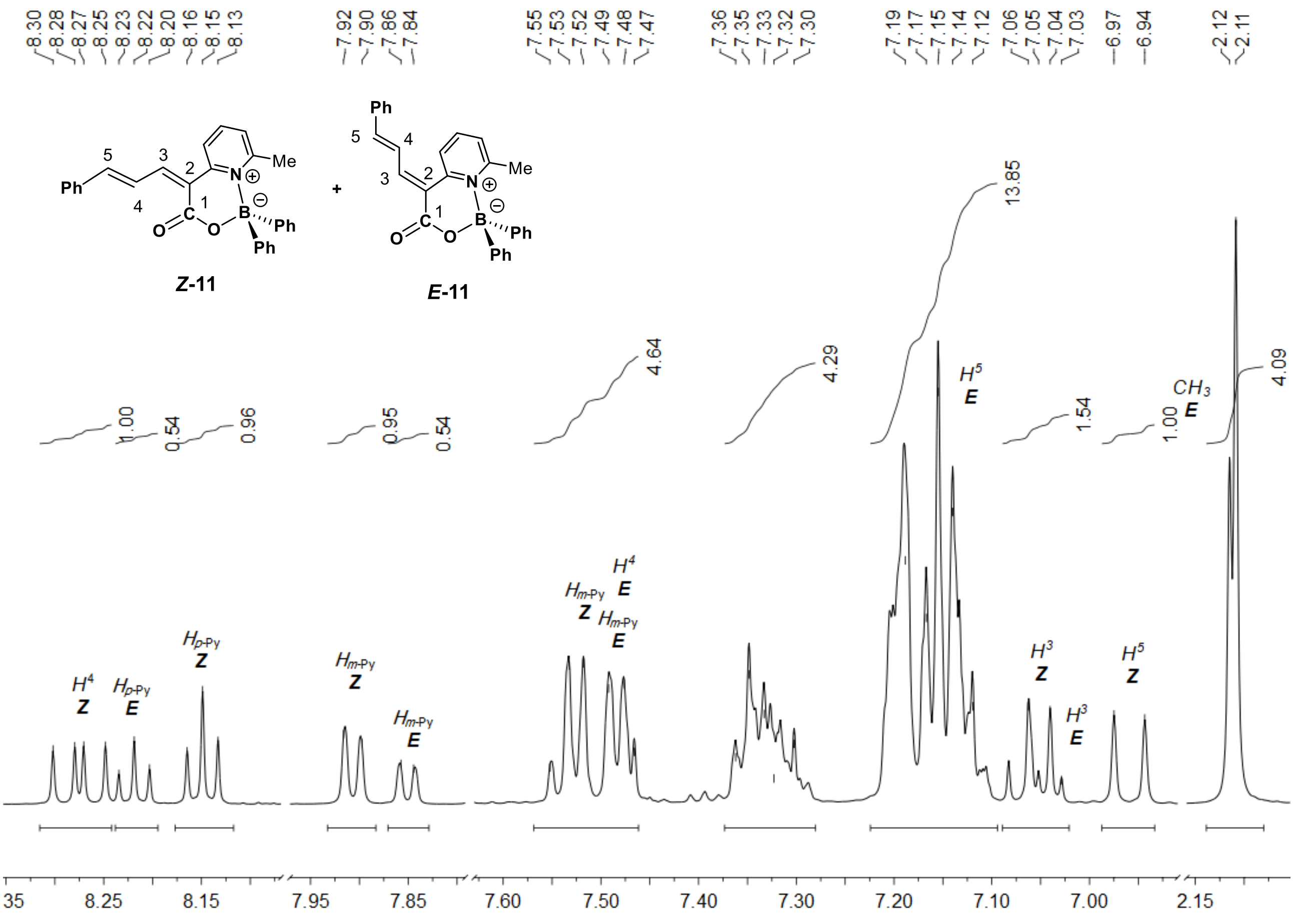

Figure S50. Enlarged ^{1}H NMR spectrum (THF-d_8, 298 K) of ***Z*/*E*-11** with assignments.

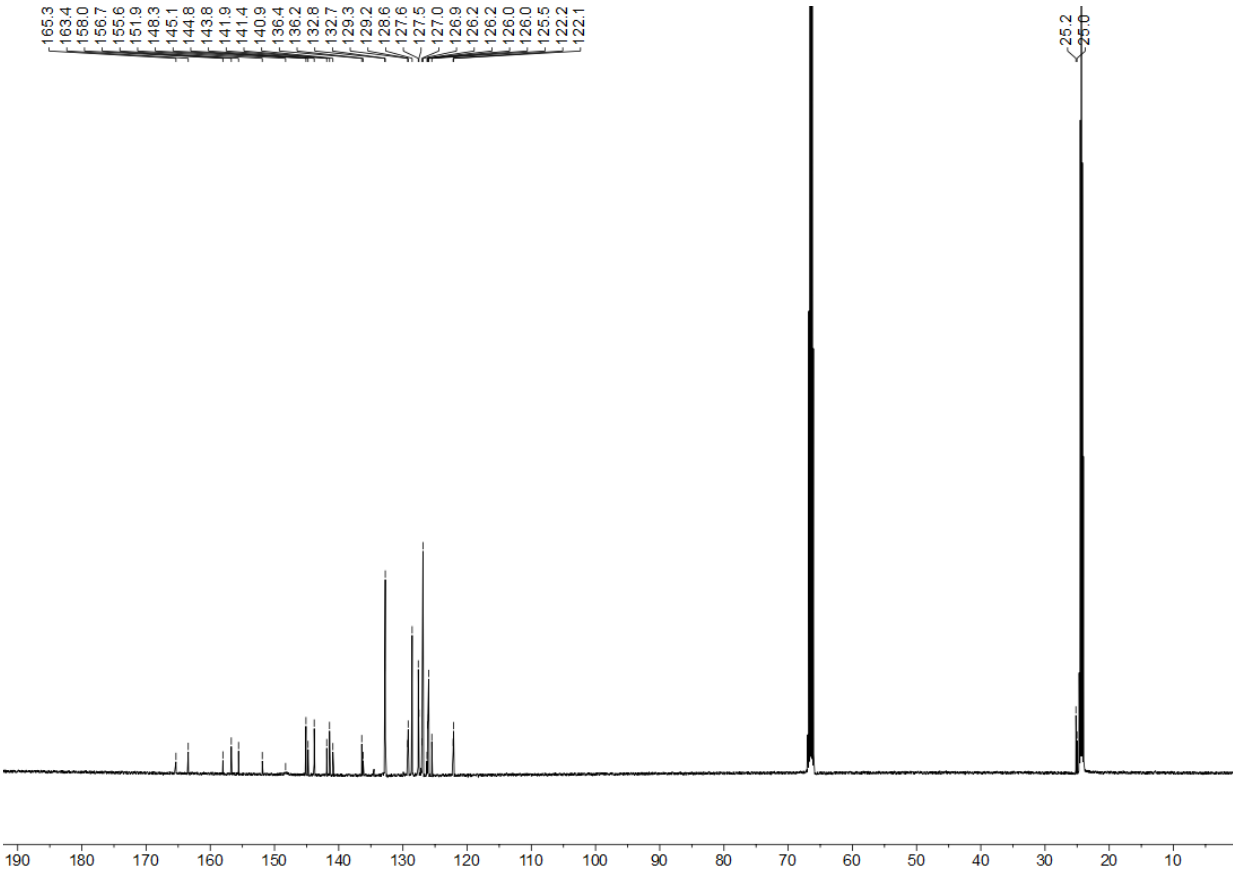

Figure S51. ^{13}C NMR spectrum (THF-d_8, 298 K) of ***Z*/*E*-11**.

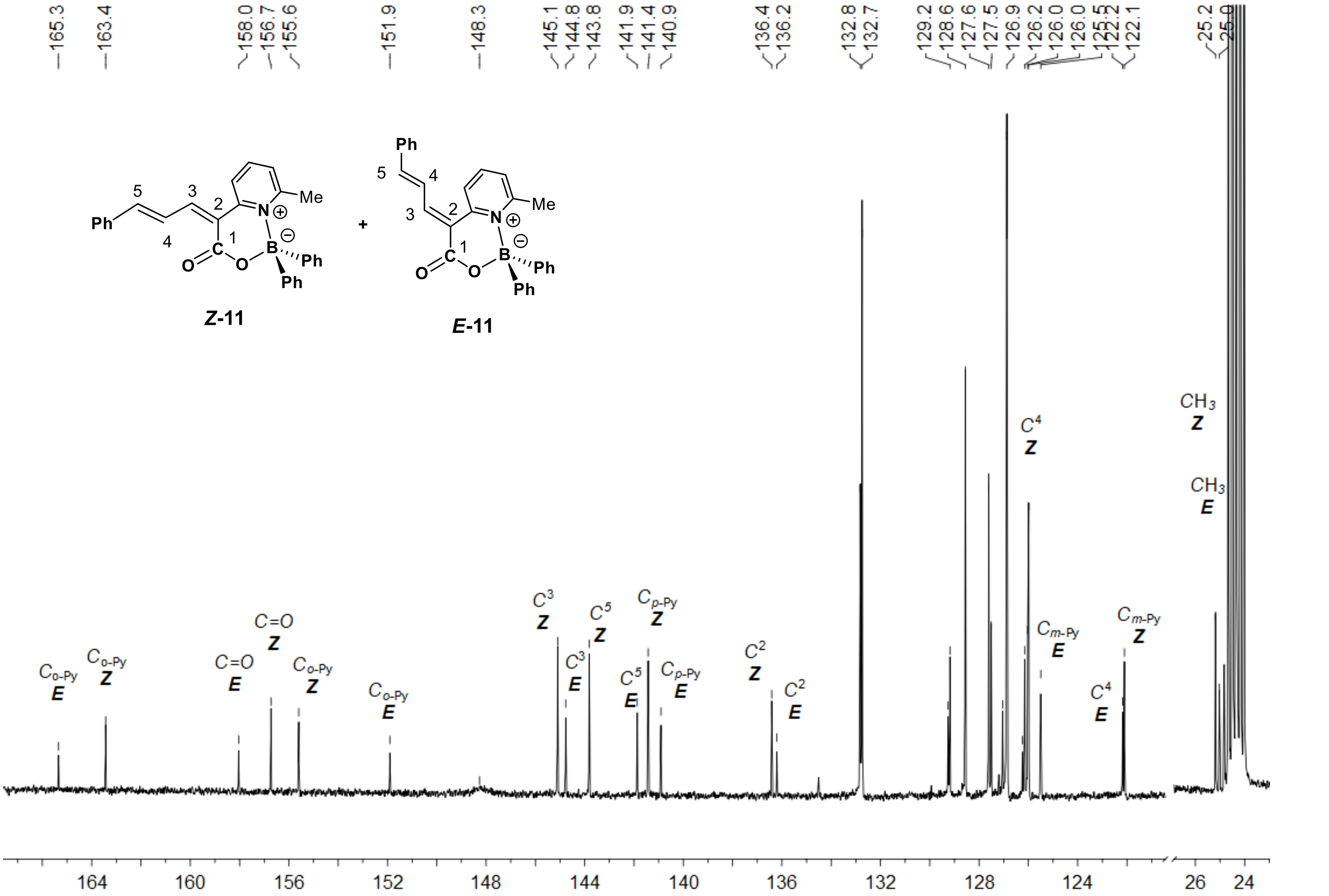

Figure S52. Enlarged ^{13}C NMR spectrum (THF-d_8, 298 K) of ***Z*/*E*-11** with assignments.

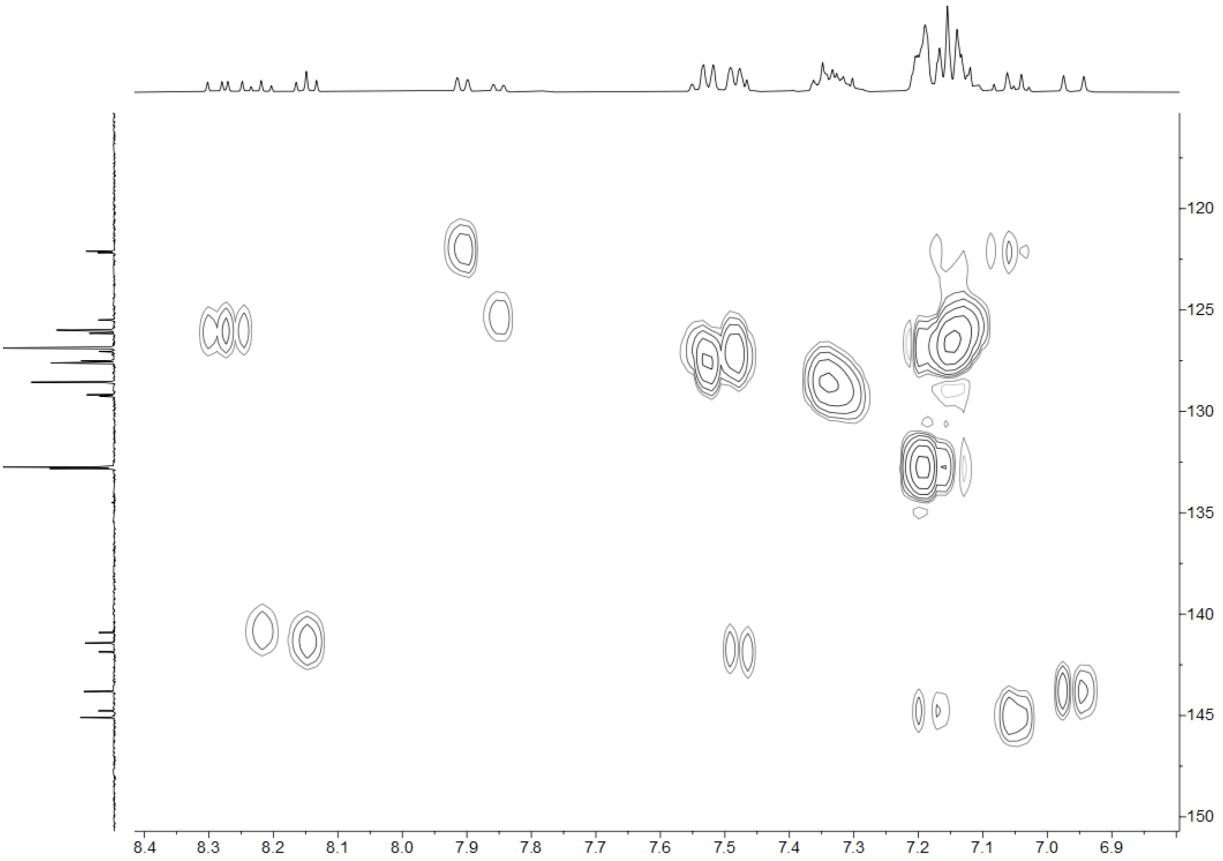

Figure S53. Enlarged HSQC (1H–^{13}C DEPT-135) NMR spectrum (THF-d_8, 298 K) of ***Z*/*E*-11**.

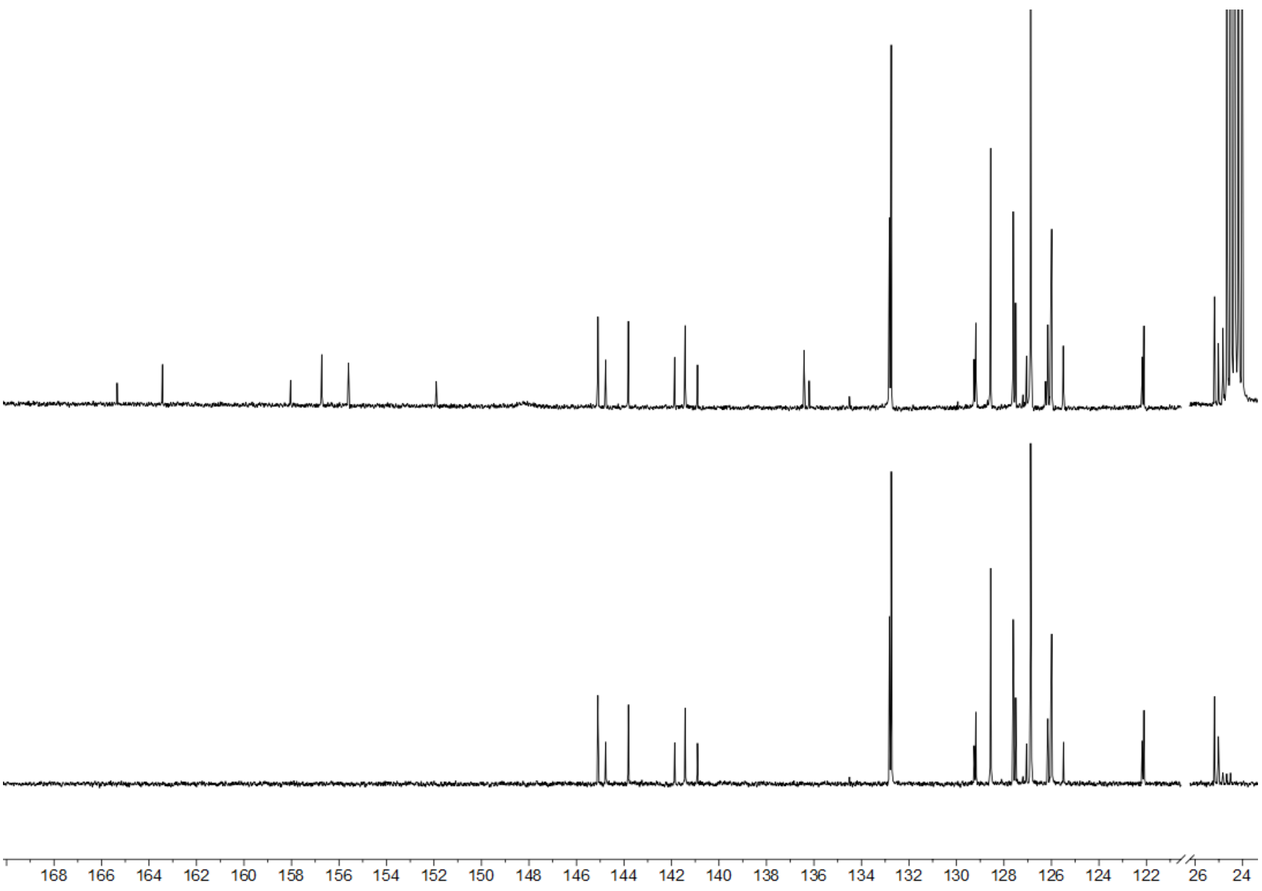

Figure S54. ^{13}C DEPT-135 (top) and ^{13}C (bottom) NMR spectra (THF-d_8, 298 K) of ***Z*/*E*-11**.

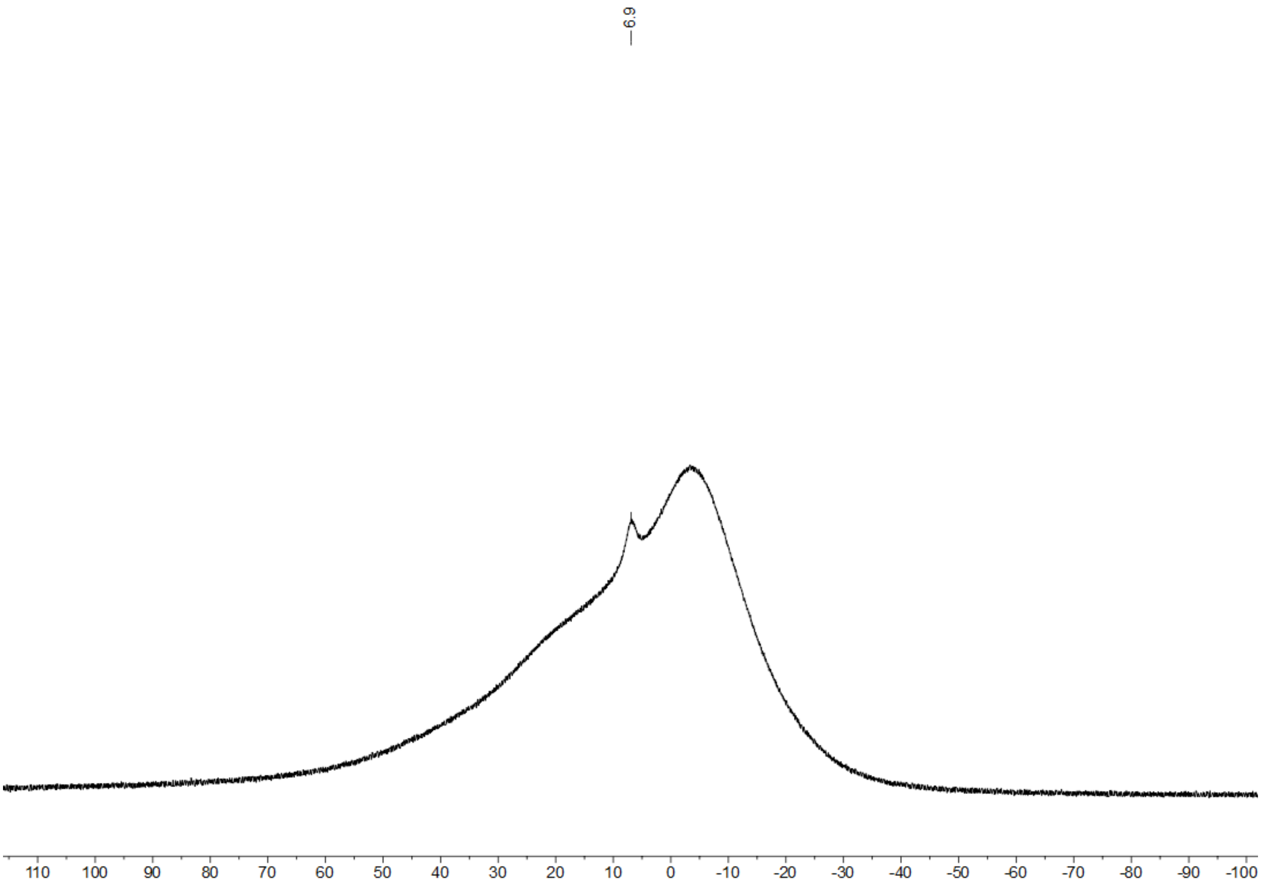

Figure S55. ^{11}B{^{1}H} NMR spectrum (THF-d_8, 298 K) of ***Z*/*E*-11**.

2.9. Synthesis of Compounds 12 and 13

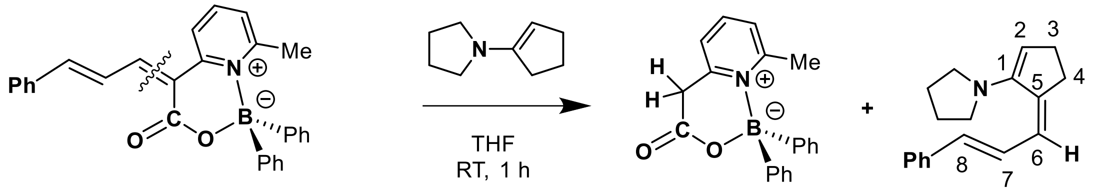

Z*/*E*-11** ^{1}H NMR ratio 1.00 : 0.54 (Z*-11** : ***E*-11**)

12 99% (62%)[a]

13 99%

[a]Isolated yield in brackets

The Z/E-11 mixture (22 mg, 0.05 mmol) in THF-d_8 (0.5 mL) was treated with 1-(1-cyclopent-1-enyl)pyrrolidine (7.5 µL, 0.05 mmol) in a Young-type NMR tube under nitrogen. NMR indicated almost complete consumption of Z/E-11 after a few hours and full conversion overnight. GC–MS analysis confirmed the formation of compound **13**, consistent with NMR data. In a preparative-scale experiment, the Z/E-11 mixture (121 mg, 0.28 mmol) in dichloromethane/toluene (0.5/0.7 mL) was reacted with 1-(1-cyclopent-1-enyl)pyrrolidine (41 µL, 0.28 mmol) at room temperature under nitrogen. After 1 h, the mixture was filtered through Celite® and washed with toluene (3 × 0.2 mL). The clear filtrate was left to slowly evaporate, affording yellow crystalline material. The solid was washed with pentane (2 × 1 mL) and dried under reduced pressure to yield compound **12** (55 mg, 0.17 mmol, 62 %). Single-crystal X-ray diffraction analysis confirmed the structure of compound **12**.

Spectroscopic data of the crude mixture of **12** and **13**:

^{1}H NMR (500.18 MHz, THF-d_8, 25 °C): δ 2.09 [s, 3H, CH_3 (**12**)], 3.63 [s, 2H, CH_2 (**12**)], 7.20 [m, 10H, H_{Ph} (**12**)], 7.50 [bd, $^{3}J_{HH}$ = 7.8 Hz 1H, $H_{m\text{-}Py}$ (**12**)], 7.58 [bd, $^{3}J_{HH}$ = 7.8 Hz 1H $H_{m\text{-}Py}$ (**12**)], 8.10 [t, $^{3}J_{HH}$ = 7.8 Hz, 1H, $H_{p\text{-}Py}$ (**12**)]. **^{13}C NMR** (125.78 MHz, THF-d_8, 25 °C): δ 25.0 [s, CH$_3$ (**12**)], 40.5 [s, CH$_2$ (**12**)], 124.2 [s, $C_{m\text{-}Py}$ (**12**)], 126.3 [s, C_{Ph} (**12**, **13**)], 127.1 [s, $C_{m\text{-}Py}$, C_{Ph} (**12**)], 132.8 [s, C_{Ph} (**12**)], 141.6 [s, $C_{p\text{-}Py}$ (**12**)], 147.3 [bs, C_{Ph} (**12**)], 154.5 [s, $C_{o\text{-}Py}$ (**12**)], 158.0 [s, C=O (**12**)], 166.2 [s, $C_{o\text{-}Py}$ (**12**)]. **^{11}B{^{1}H} NMR** (160.47 MHz, THF-d_8, 25 °C): δ 7.3 (bs).

^{1}H NMR (500.18 MHz, THF-d_8, 25 °C): δ 1.89 [m, 4H, 2 x CH_2 (**13**)], 2.47 [m, 2H, CH_2 (**13**)], 2.79 [m, 2H, CH_2 (**13**)], 3.16 [m, 4H, 2 x CH_2 (**13**)], 5.06 [t, $^{3}J_{HH}$ = 2.9 Hz 1H, C=CH^2 (**13**)], 6.35 [dd, $^{3}J_{HH}$ = 11.2 Hz, $^{4}J_{HH}$ = 2.2 Hz 1H, C=CH_2^6 (**13**)], 6.51 [d, $^{3}J_{HH}$ = 15.8 Hz 1H, C=CH_2^8 (**13**)], 7.05 [dd, $^{3}J_{HH}$ = 15.8 Hz, $^{3}J_{HH}$ = 11.2 Hz 1H, C=CH^7 (**13**)], 7.16 [bt, $^{3}J_{HH}$ = 7.7 Hz 1H, H_{Ph} (**13**)], 7.28 [bt, $^{3}J_{HH}$ = 7.7 Hz 2H, H_{Ph} (**13**)], 7.43 [bd, $^{3}J_{HH}$ = 7.7 Hz 2H, H_{Ph} (**13**)]. **^{13}C NMR** (125.78 MHz, THF-d_8, 25 °C): δ 24.7 [s, CH$_2$ (**13**)], 27.9 [s, CH$_2^4$ (**13**)], 28.1 [s, CH$_2^3$ (**13**)], 50.4 [s, CH$_2$ (**13**)], 110.6 [s, C=CH^2 (**13**)], 118.3 [s, C=CH^6 (**13**)], 125.8 [s, C_{Ph} (**13**)], 126.3 [s, C_{Ph} (**12**, **13**)], 126.5 [s, C_{Ph} (**13**)], 126.9 [s, C=CH^7 (**13**)], 128.3 [s, C_{Ph} (**13**)], 130.0 [s, C=CH^8 (**13**)], 138.3 [s, C_{Ph} (**13**)], 146.1 [s, C=C^5 (**13**)], 151.8 [s, C=C^1 (**13**)]. **^{11}B{^{1}H} NMR** (160.47 MHz, THF-d_8, 25 °C): δ 7.3 (bs). **HRMS (ESI$^+$)** for compound **13**, calcd. m/z for [(M+H)$^+$]: 252.1747; found 252.1751.

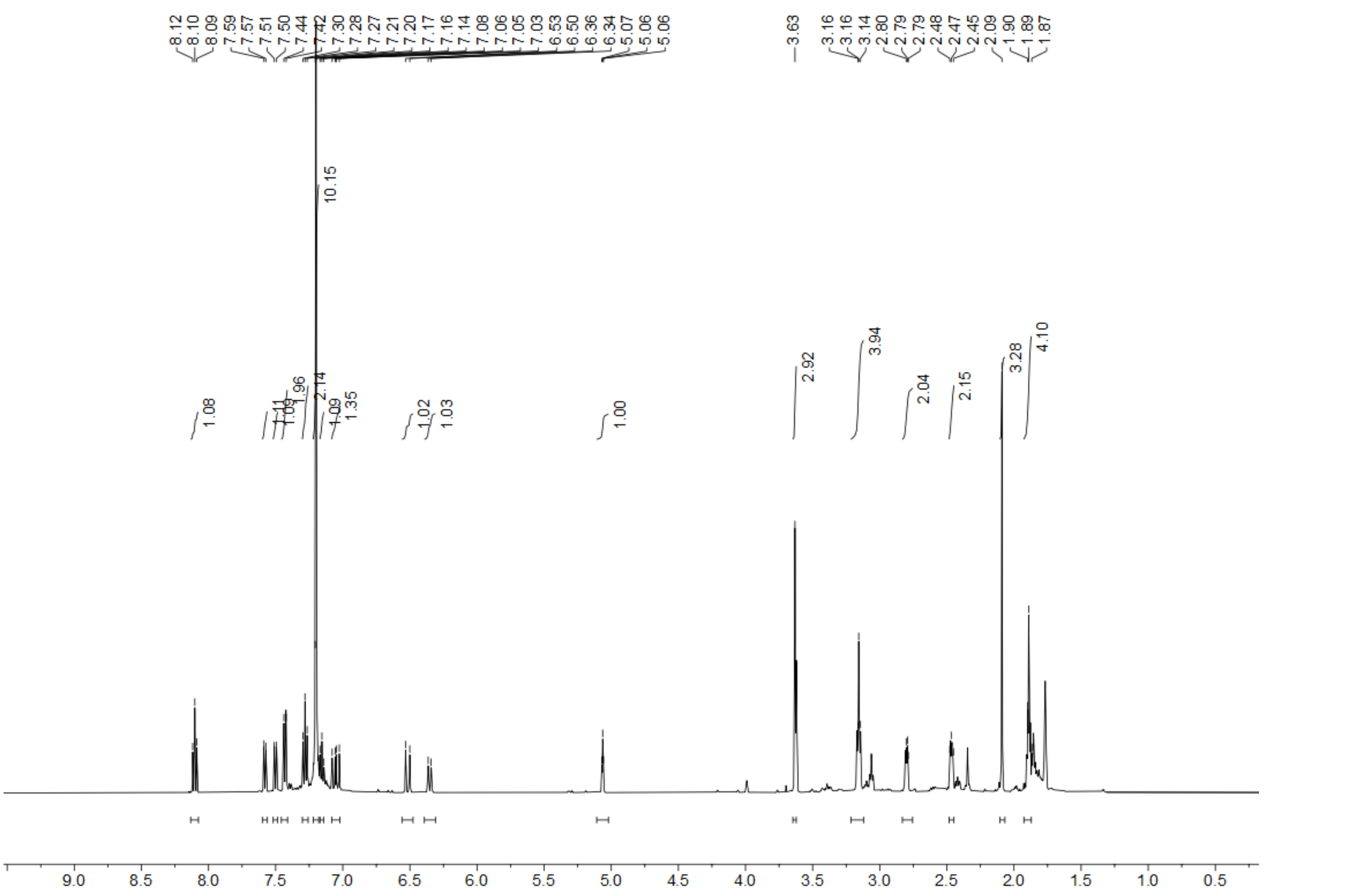

Figure S56. ^{1}H NMR spectrum (THF-d_8, 298 K) of the mixture **12**/**13**.

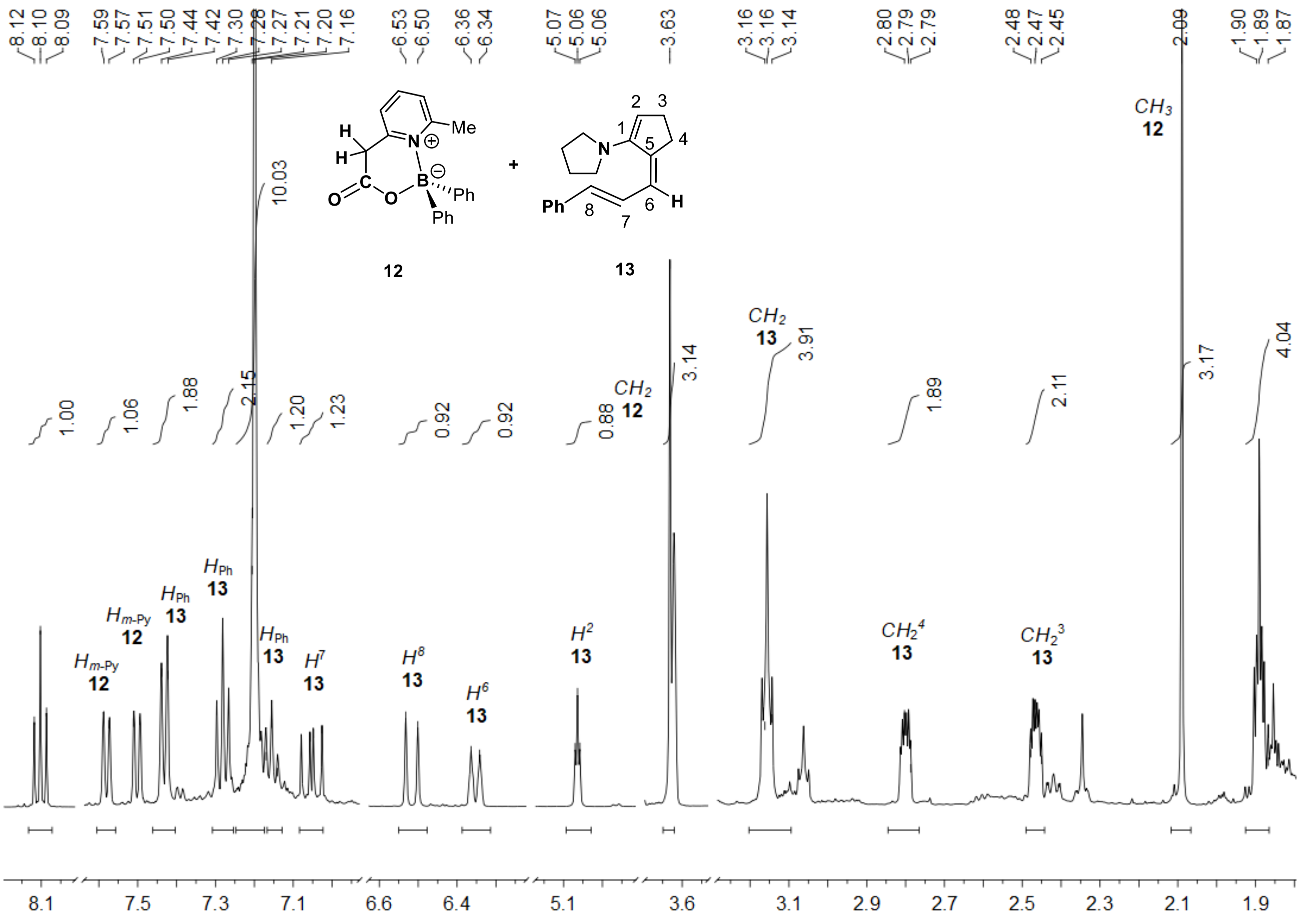

Figure S57. Enlarged ^{1}H NMR spectrum (THF-d_8, 298 K) of the mixture **12**/**13** with assignments.

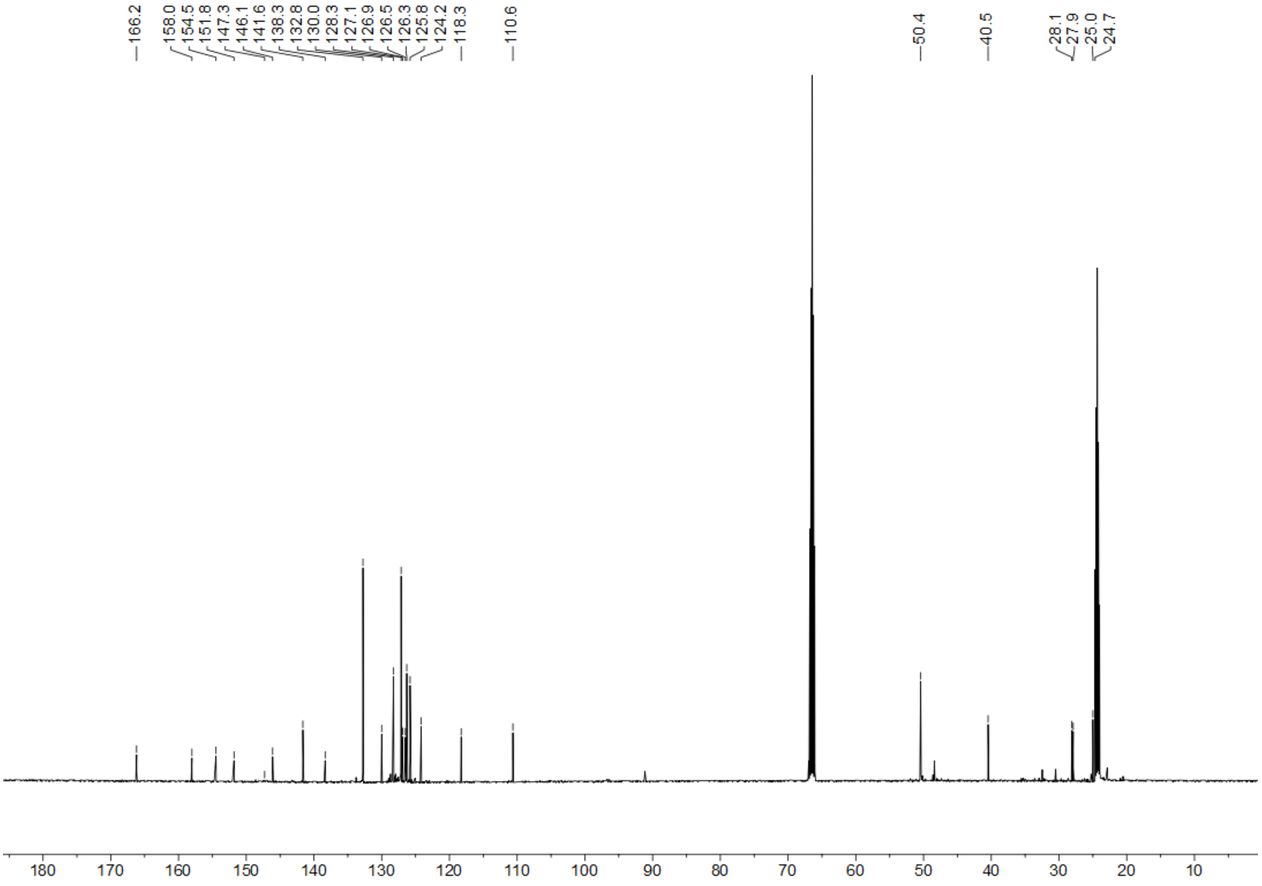

Figure S58. ^{13}C NMR spectrum (THF-d_8, 298 K) of the mixture **12**/**13**.

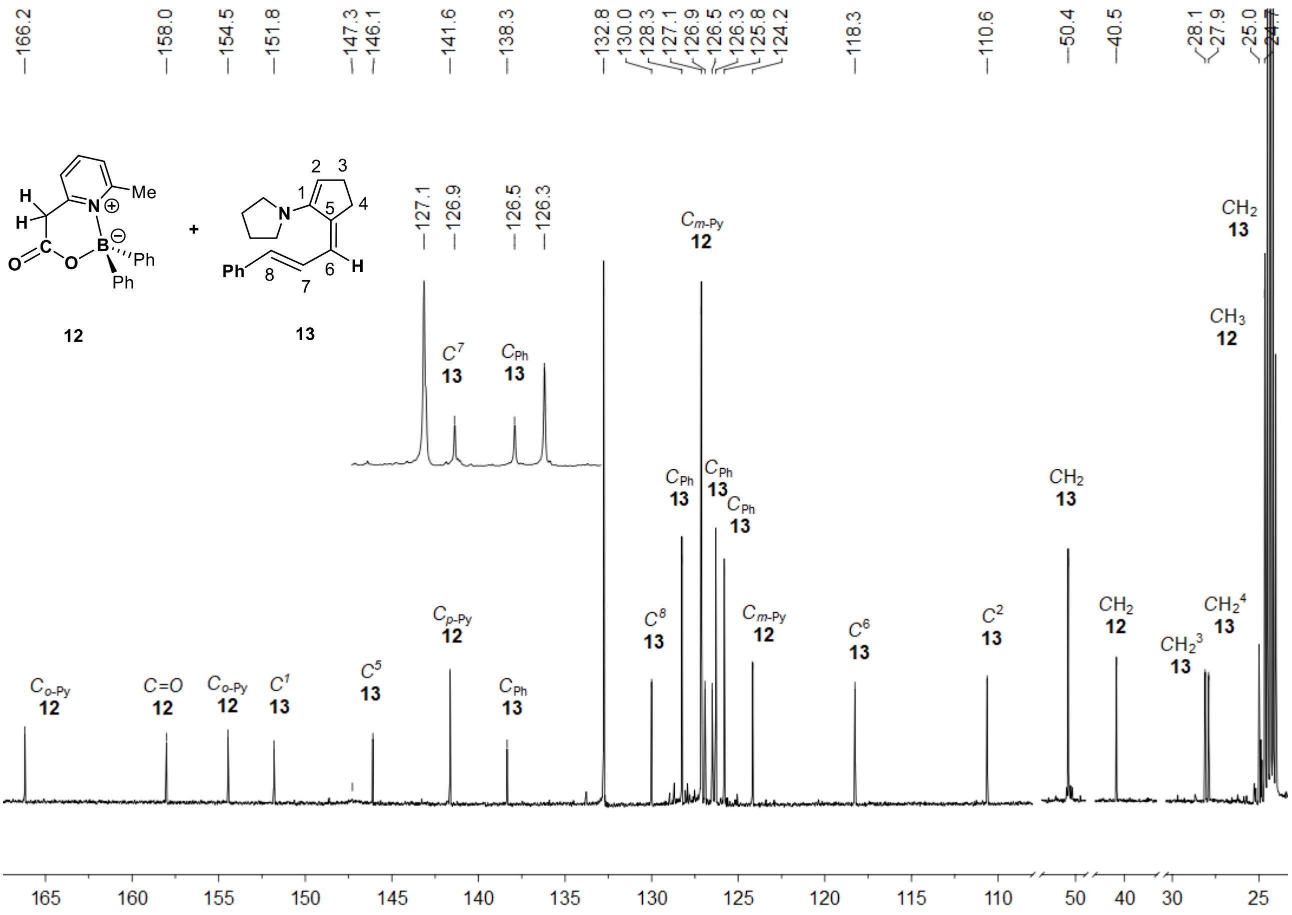

Figure S59. Enlarged ^{13}C NMR spectrum (THF-d_8, 298 K) of the mixture **12**/**13** with assignments.

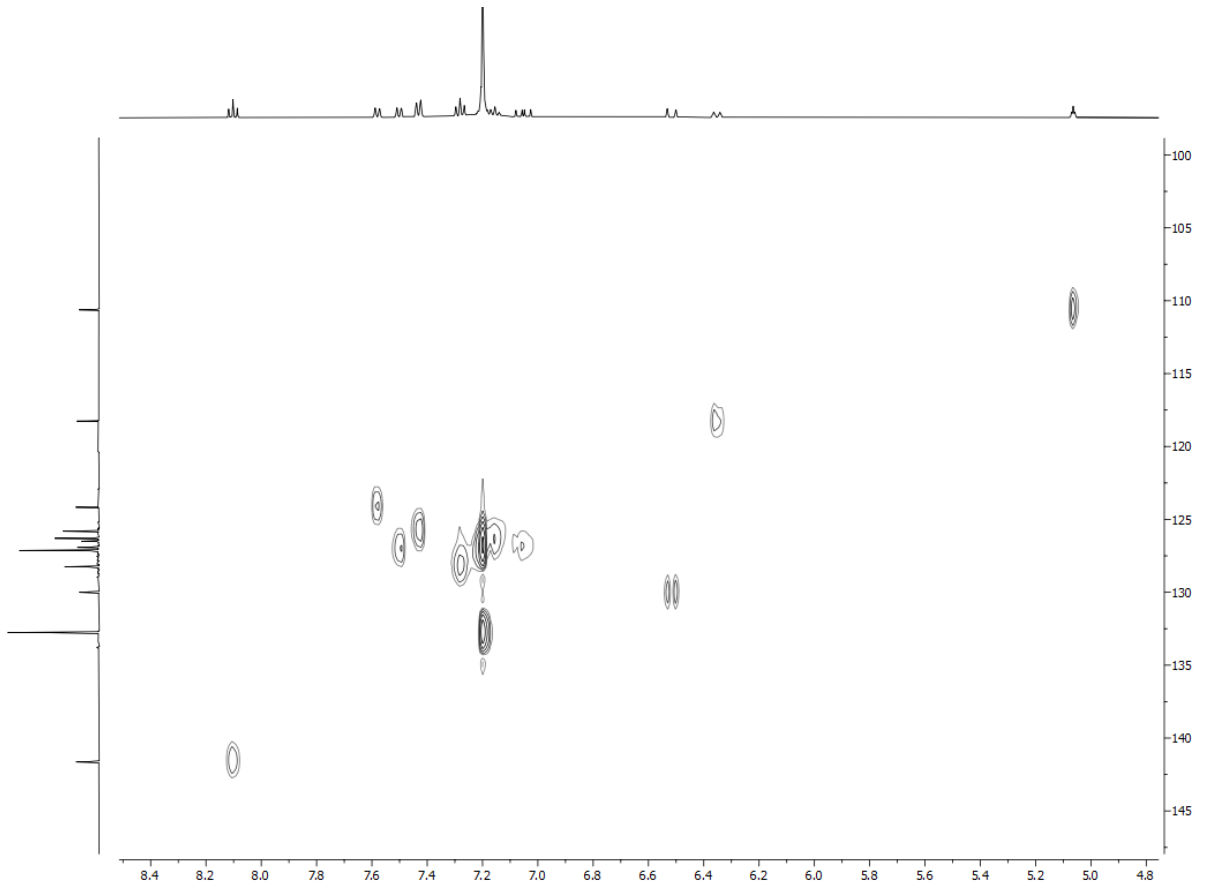

Figure S60. Enlarged HSQC (^{1}H–13C DEPT-135) NMR spectrum (THF-d_8, 298 K) of the mixture **12**/**13**.

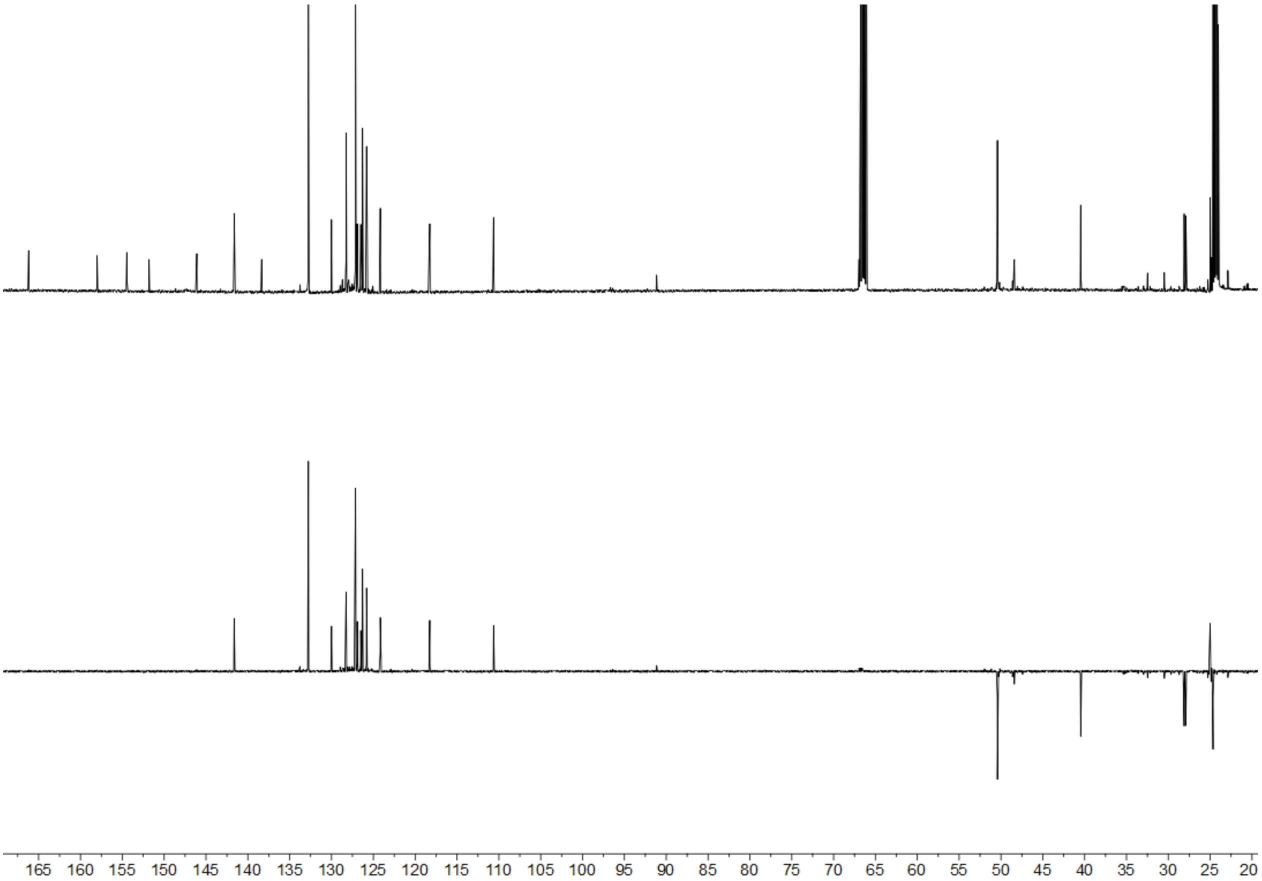

Figure S61. ^{13}C (top) and ^{13}C DEPT-135 (bottom) NMR spectra (THF-d_8, 298 K) of the mixture **12**/**13**.

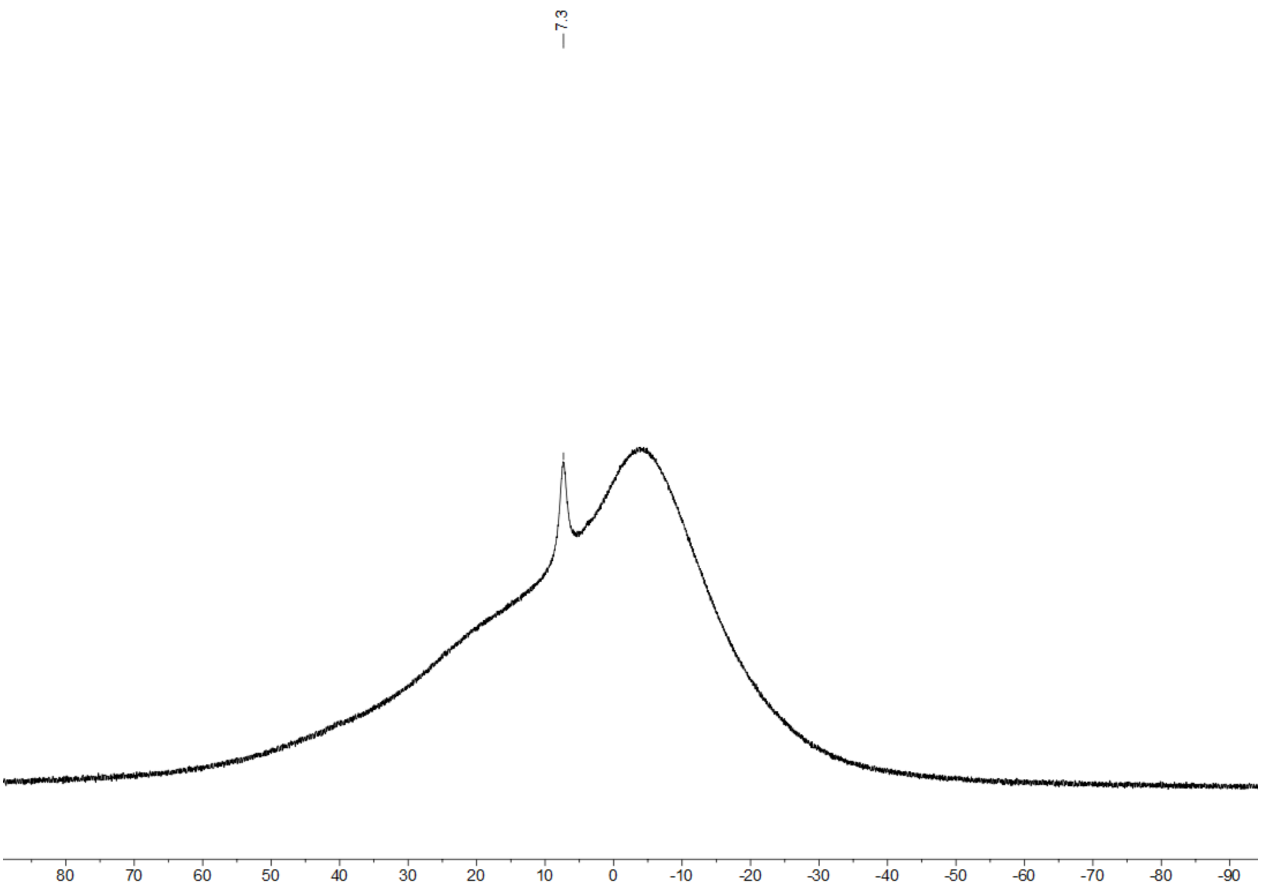

Figure S62. ^{11}B{^{1}H} NMR spectrum (THF-d_8, 298 K) of the mixture **12**/**13**.

Spectroscopic data of the isolated compound **12**:

^{1}H NMR (500.18 MHz, THF-d_8, 25 °C): δ 2.09 (s, 3H, CH_3), 3.63 (s, 2H, CH_2), 7.20 (m, 10H, H_{Ph}), 7.51 (bd, $^{3}J_{\text{HH}}$ = 7.8 Hz 1H $H_{m\text{-Py}}$), 7.59 (bd, $^{3}J_{\text{HH}}$ = 7.8 Hz 1H $H_{m\text{-Py}}$), 8.11 (t, $^{3}J_{\text{HH}}$ = 7.8 Hz, 1H, $H_{p\text{-Py}}$). **^{13}C NMR** (125.78 MHz, THF-d_8, 25 °C): δ 25.0 (s, CH$_3$), 40.5 (s, CH$_2$), 124.2 (s, $C_{m\text{-Py}}$), 126.3 (s, C_{Ph}), 127.1 (s, $C_{m\text{-Py}}$, C_{Ph}), 132.8 (s, C_{Ph}), 141.6 (s, $C_{p\text{-Py}}$), 147.3 (bs, C_{Ph}), 154.5 (s, $C_{o\text{-Py}}$), 158.0 (s, C=O), 166.2 (s, $C_{o\text{-Py}}$). **^{11}B{^{1}H} NMR** (160.47 MHz, THF-d_8, 25 °C): δ 7.2 (bs). **HRMS (FD^{+})**, calcd. m/z for [(M)$^{+}$]: 315.14251; found 315.14137. **Elemental analysis**: calcd. for $C_{20}H_{18}BNO_2$: C 76.22, H 5.76, N 4.44; found: C 76.26, H 6.08, N 4.44.

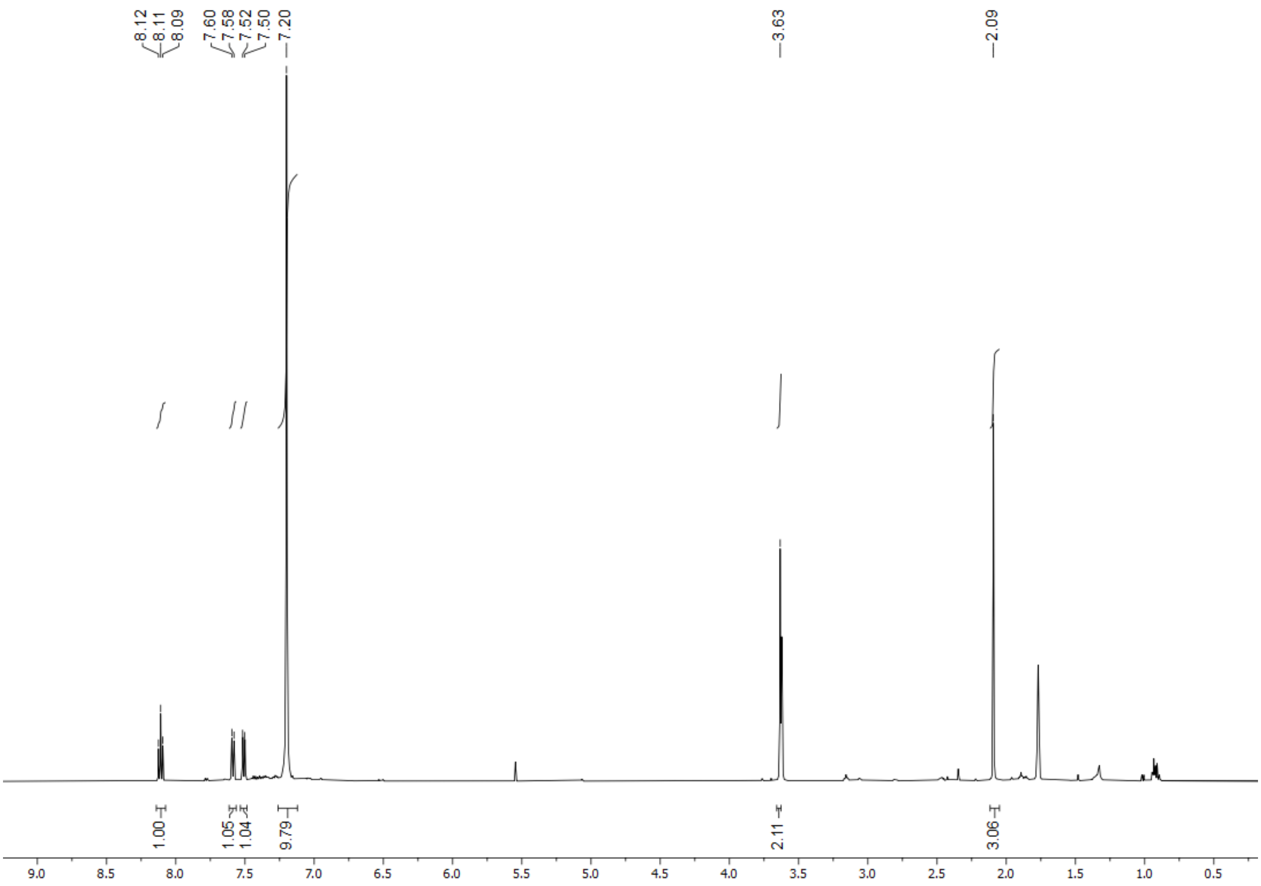

Figure S63. ^{1}H NMR spectrum (THF-d_8, 298 K) of compound **12**.

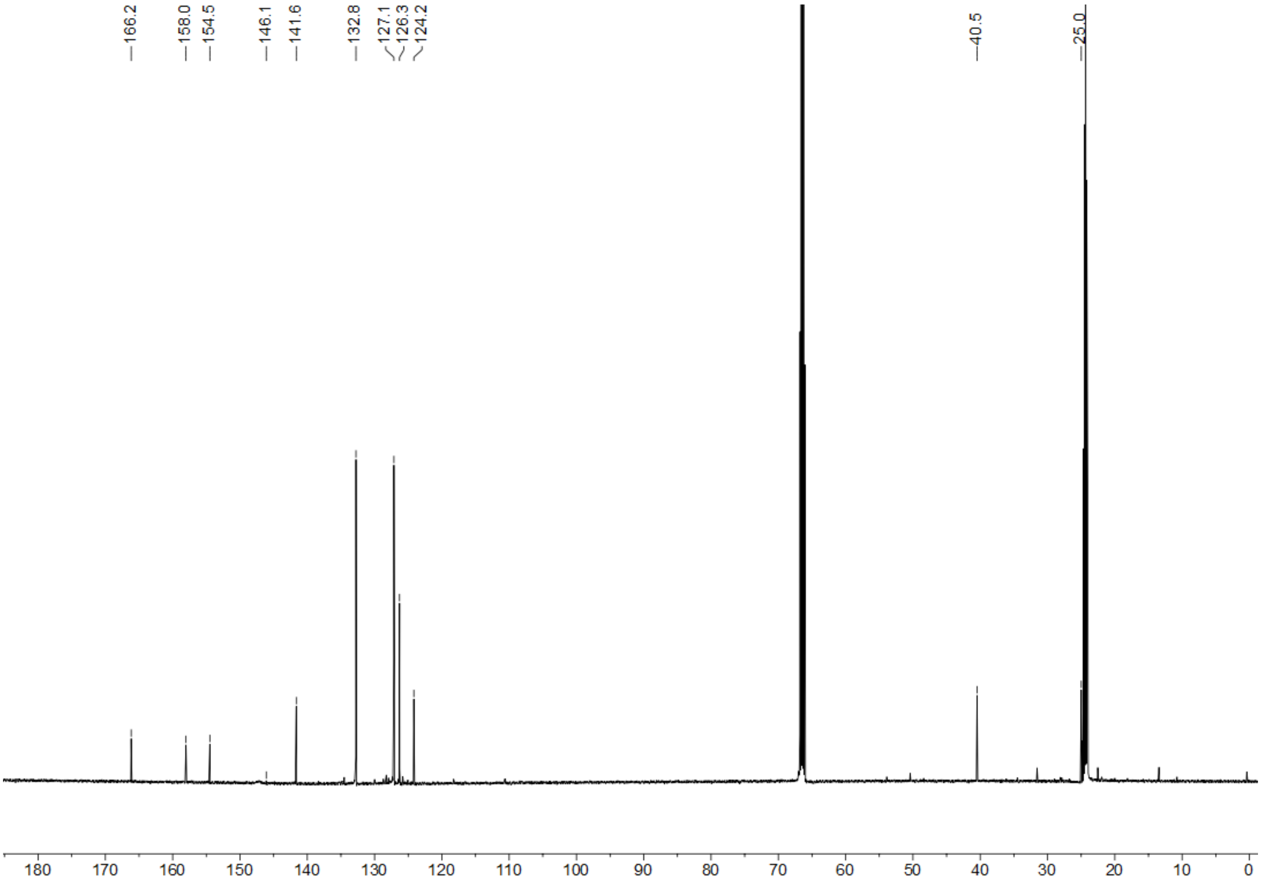

Figure S64. ^{13}C NMR spectrum (THF-d_8, 298 K) of compound **12**.

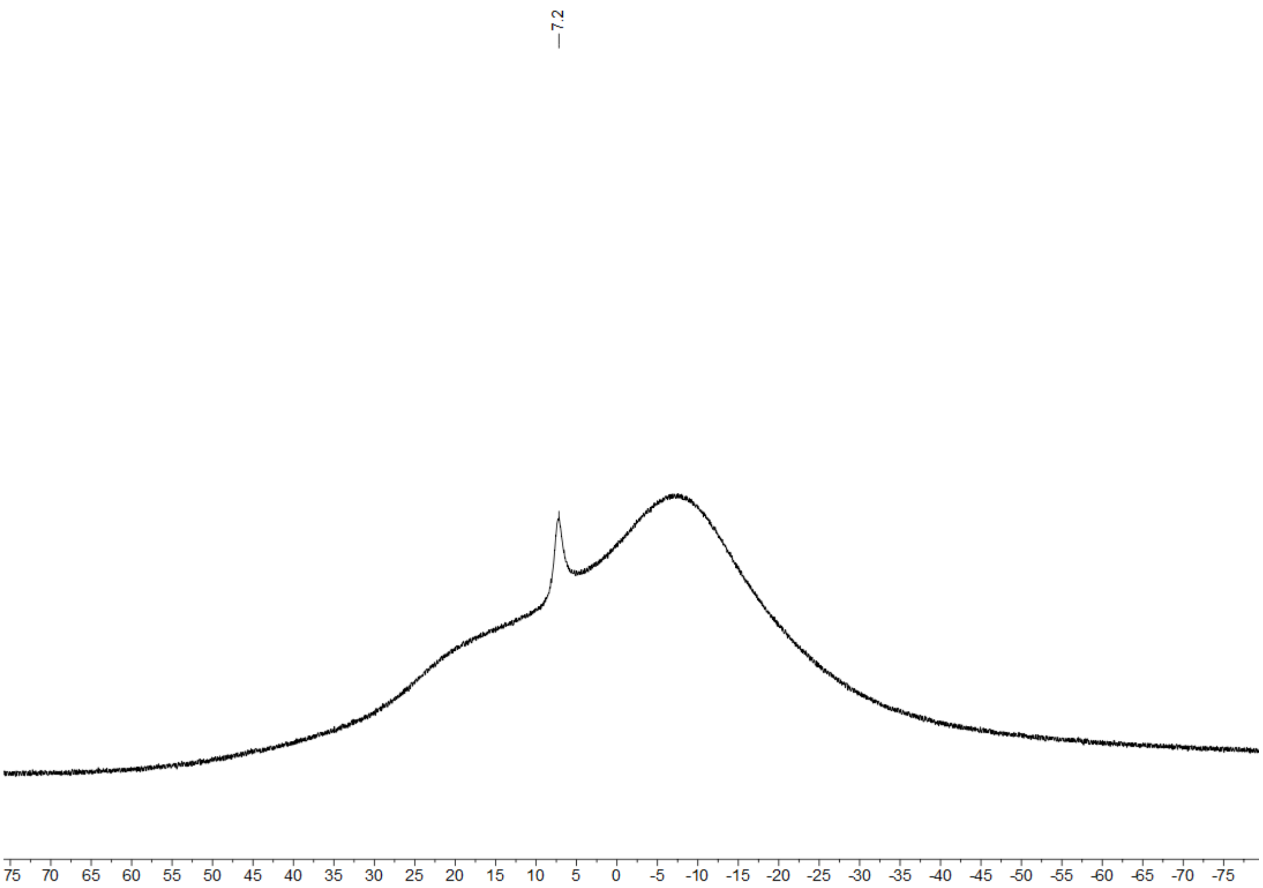

Figure S65. $^{11}B\{^{1}H\}$ NMR spectrum (THF-d_8, 298 K) of compound **12**.

3. Recovery of Compound 5

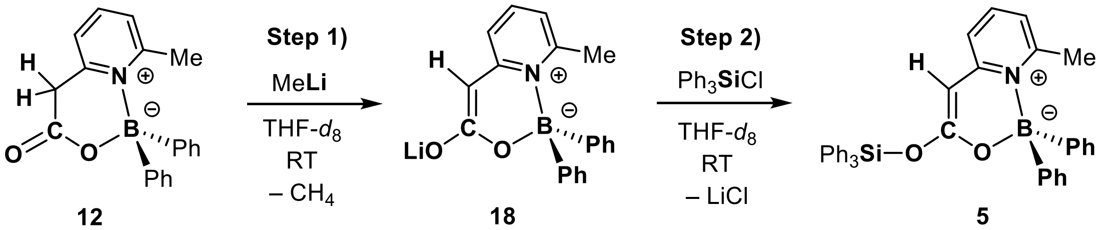

Step 1) Generation of Compound 18

An oven-dried intermediate-pressure Young-type NMR tube was charged with methyllithium (0.08 mL of a 1.6 M solution in diethyl ether, 0.13 mmol) under nitrogen, sealed, and carefully evacuated to dryness, leaving methyllithium as a white powder. The NMR tube was then introduced into a nitrogen-filled glovebox, and THF-d_8 (0.2 mL) was added via syringe, rinsing the tube walls during addition. Compound **12** (41 mg, 0.13 mmol) was suspended in THF-d_8 (0.3 mL) and added slowly to the methyllithium solution at room temperature. Immediate gas evolution was observed, and the solution turned dark red. The remaining solids were transferred with additional THF-d_8 (0.1 mL). The NMR tube was sealed, shaken, briefly opened to release overpressure caused by methane evolution, and then allowed to react for ca. 30 min. The tube was subsequently sealed, removed from the glovebox, and analyzed by NMR, confirming full consumption of precursor **12** and clean formation of compound **18**.

^{1}H NMR (400.30 MHz, THF-d_8, 25 °C): δ 1.73 (s, 3H, CH_3), 4.36 (s, 2H, CH_2), 5.96 (d, $^3J_{HH}$ = 7.0 Hz, 1H $H_{m\text{-Py}}$), 6.36 (d, $^3J_{HH}$ = 8.8 Hz, 1H $H_{m\text{-Py}}$), 6.97 (dd, $^3J_{HH}$ = 8.8 Hz, $^3J_{HH}$ = 7.0 Hz, 1H, $H_{p\text{-Py}}$), 7.12 – 7.20 (m, 6H, H_{Ph}), 7.42 (m, 4H, H_{Ph}). **^{13}C NMR** (100.65 MHz, THF-d_8, 25 °C): δ 24.9 (s, CH$_3$), 76.9 (s, CH), 110.9 (s, $C_{m\text{-Py}}$), 117.5 (s, $C_{m\text{-Py}}$), 122.9 (s, C_{Ph}), 124.7 (s, C_{Ph}), 125.1 (s, C_{Ph}), 126.1 (s, C_{Ph}), 133.2 (s, C_{Ph}), 134.2 (s, C_{Ph}), 134.6 (s, $C_{p\text{-Py}}$), 152.6 (s, $C_{o\text{-Py}}$), 159.1 (s, C=O), 170.8 (s, $C_{o\text{-Py}}$). **^{11}B{^{1}H} NMR** (128.43 MHz, THF-d_8, 25 °C): δ 5.7 (bs). **^{7}Li{^{1}H} NMR** (128.43 MHz, THF-d_8, 25 °C): δ 0.0 (bs).

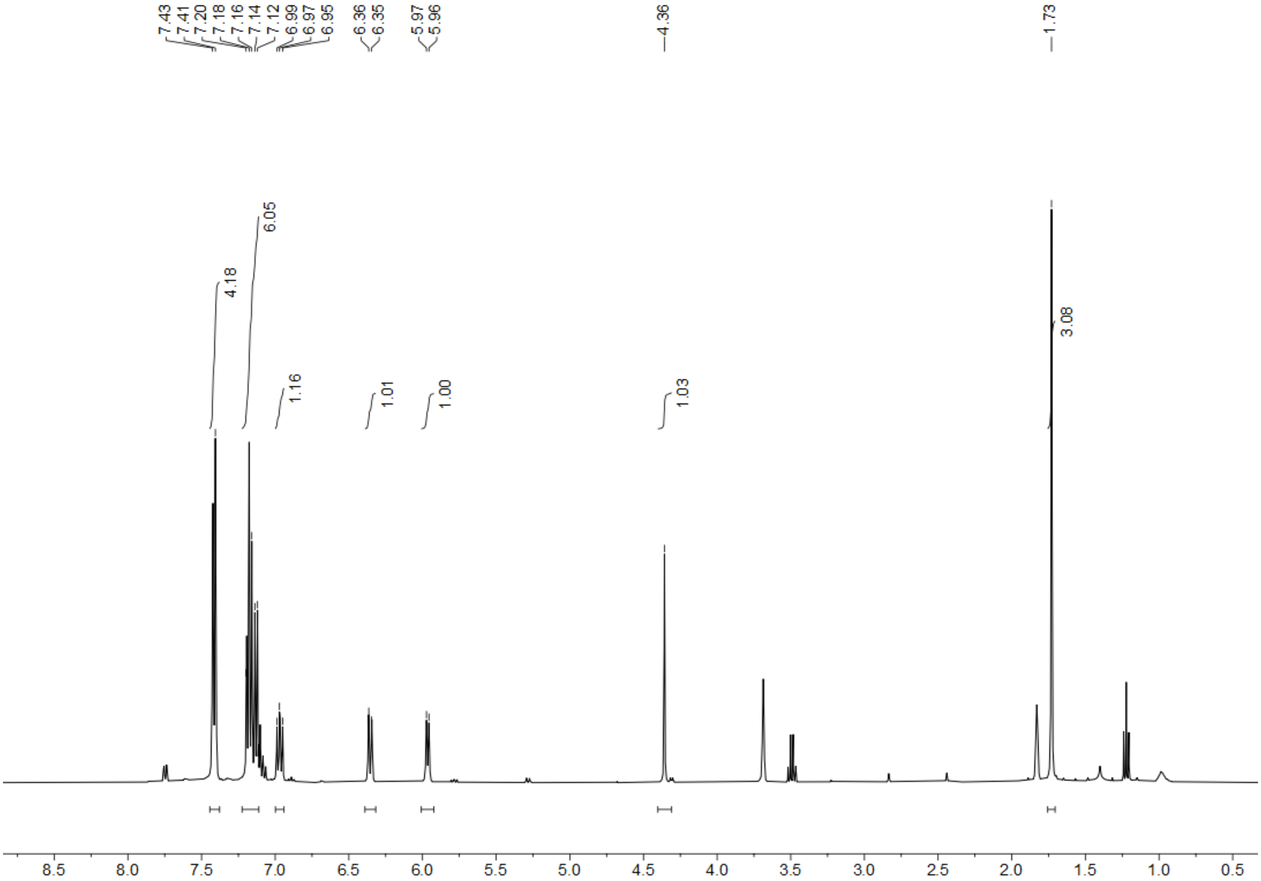

Figure S66. ^{1}H NMR spectrum (THF-d_8, 298 K) of the crude mixture containing compound **18**.

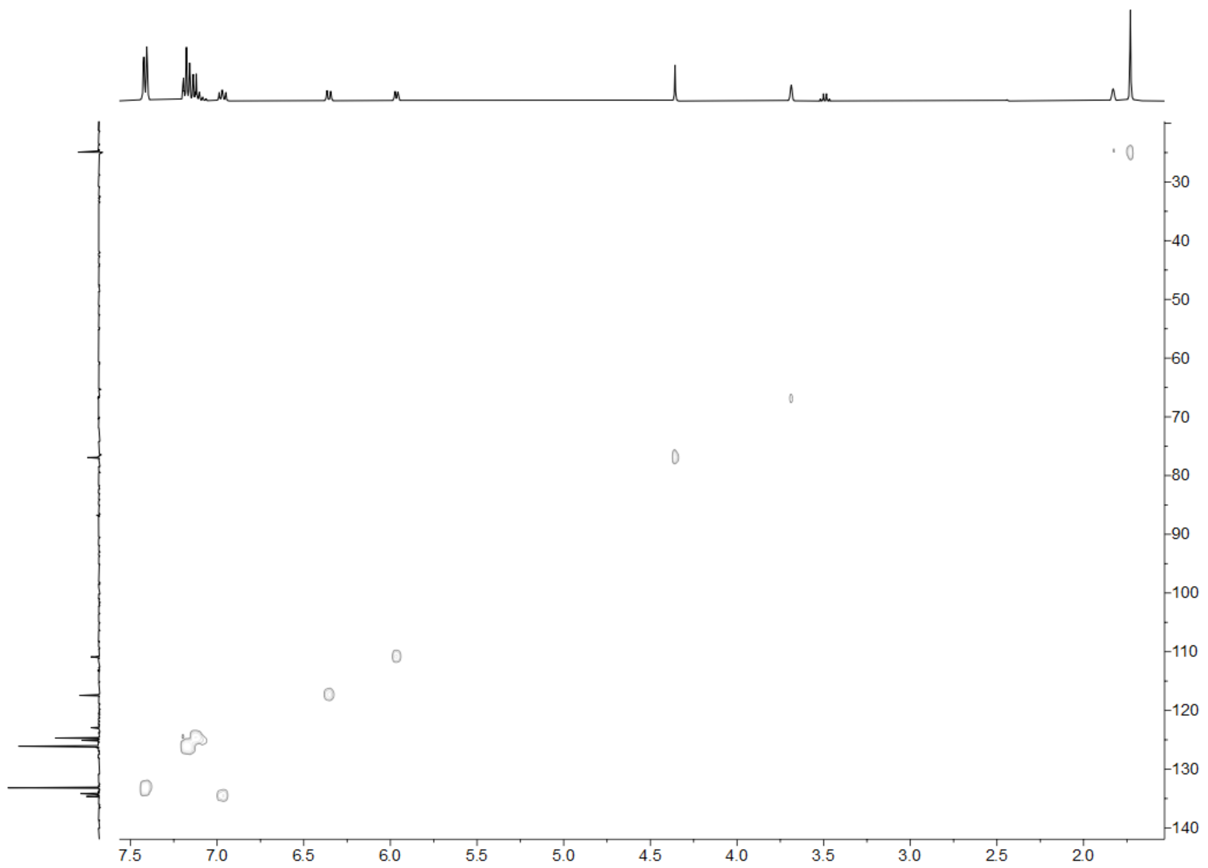

Figure S67. HSQC (1H–^{13}C DEPT-135) NMR spectrum (THF-d_8, 298 K) of the crude mixture containing compound **18**.

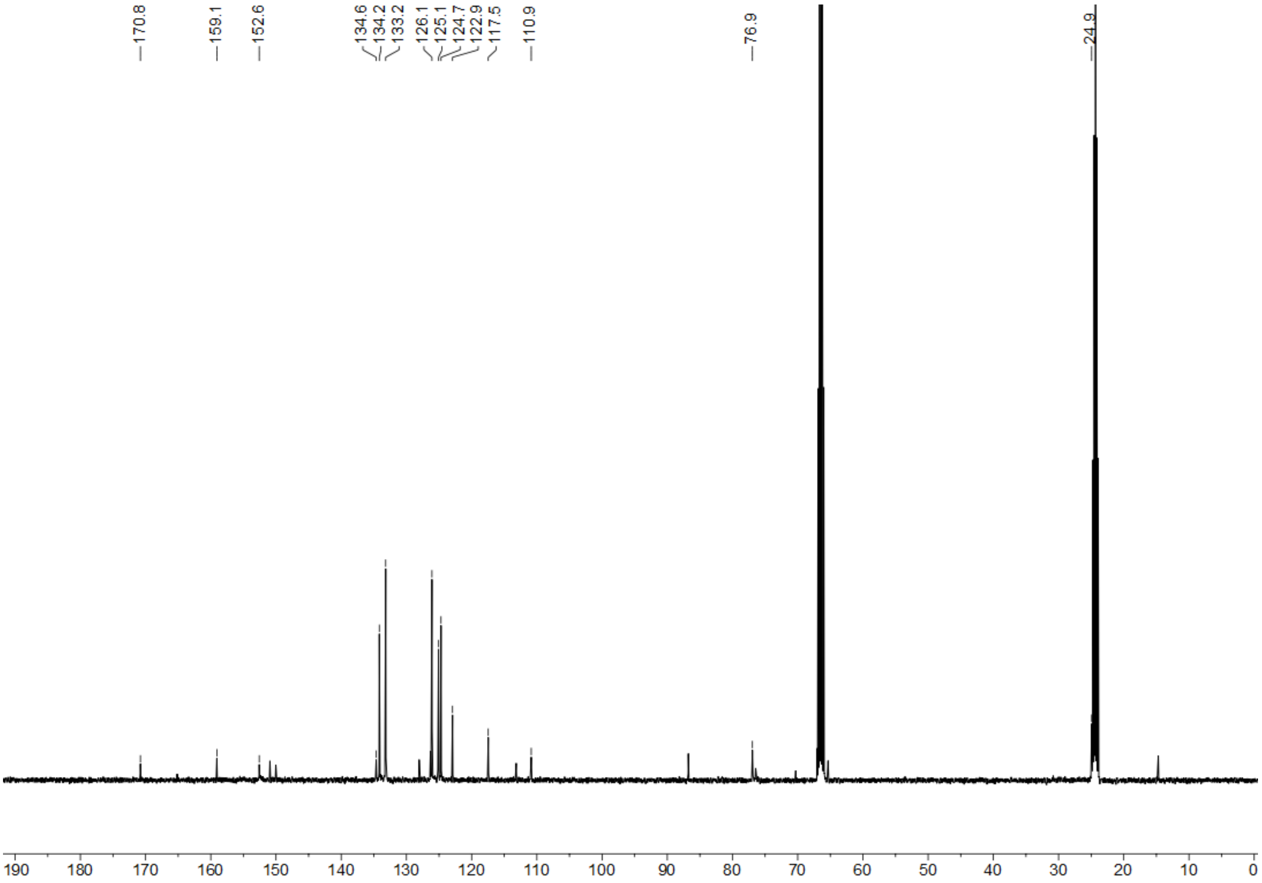

Figure S68. ^{13}C NMR spectrum (THF-d_8, 298 K) of the crude mixture containing compound **18**.

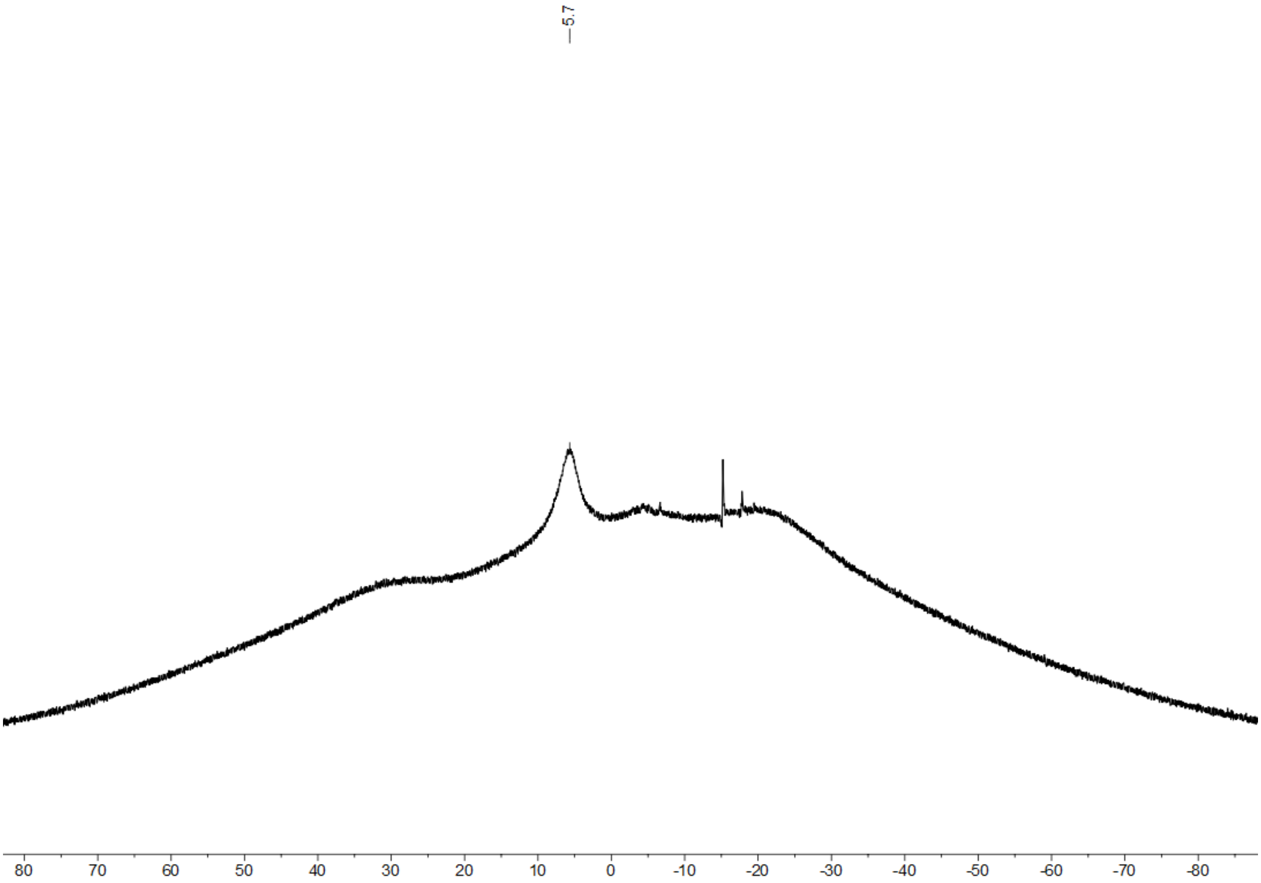

Figure S69. $^{11}B\{^1H\}$ NMR spectrum (THF-*d*$_8$, 298 K) of the crude mixture containing compound **18**.

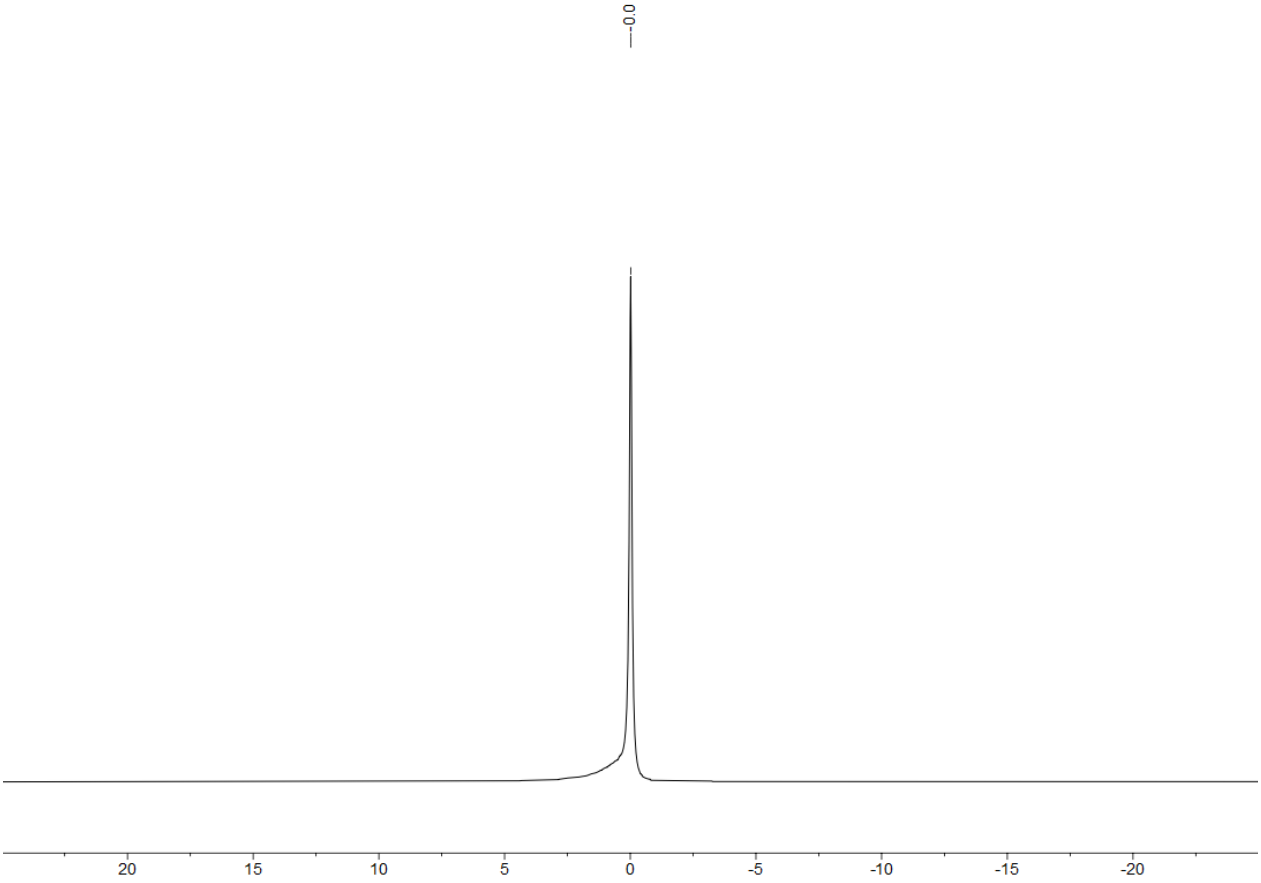

Figure S70. $^{7}Li\{^1H\}$ NMR spectrum (THF-*d*$_8$, 298 K) of the crude mixture containing compound **18**.

Step 2) Conversion of 18 to Compound 5

The NMR sample containing compound **18** was returned to the nitrogen-filled glovebox, and chlorotriphenylsilane (38 mg, 0.13 mmol) was added in one portion at room temperature. The tube was sealed, shaken, and allowed to react overnight at room temperature. The NMR tube was then removed from the glovebox, attached to a Schlenk line, and evaporated to dryness under reduced pressure, affording an oily orange residue. The tube was reintroduced into the glovebox, and the residue was extracted with toluene-d_8 (0.4 mL). The suspension was filtered through a small plug of Celite®, and the solids were rinsed with toluene-d_8 (2 × 0.2 mL). The combined clear orange filtrates were collected in an oven-dried intermediate-pressure Young-type NMR tube. After sealing and removal from the glovebox, NMR analysis confirmed full conversion of **18** to compound **5**.

Spectroscopic data of the toluene-d_8 filtrates containing compound **5**:

^{1}H NMR (500.18 MHz, Tol-d_8, 25 °C): δ 1.69 (s, 3H, CH_3), 4.94 (s, 1H, C=CH), 5.85 (dd, $^3J_{HH}$ = 7.5 Hz, $^4J_{HH}$ = 0.6 Hz, 1H, $H_{m\text{-Py}}$), 6.18 (dd, $^3J_{HH}$ = 8.5 Hz, $^4J_{HH}$ = 0.6 Hz, 1H, $H_{m\text{-Py}}$), 6.64 (dd, $^3J_{HH}$ = 8.6 Hz, $^3J_{HH}$ = 7.5 Hz, $H_{p\text{-Py}}$), 7.34 – 7.41 (m, 19H, H_{Ph}), 7.65 (m, 6H, H_{Ph}). **^{13}C NMR** (125.78 MHz, Tol-d_8, 25 °C): δ 25.4 (s, CH$_3$), 78.4 (s, C=CH), 118.1 (s, $C_{m\text{-Py}}$), 118.1 (s, $C_{m\text{-Py}}$), 126.1 (s, C_{Ph}), 127.1 (s, C_{Ph}), 127.8 (s, C_{Ph}), 130.1 (s, C_{Ph}), 132.8 (s, $C_{p\text{-Py}}$), 133.3 (s, C_{Ph}), 135.9 (s, C_{Ph}), 137.1 (s, C_{Ph}), 138.1 (s, C_{Ph}), 148.3 (bs, C_{Ph}), 153.5 (s, $C_{o\text{-Py}}$), 157.5 (s, C=CH), 164.1 (bs, $C_{o\text{-Py}}$). **^{11}B{^{1}H} NMR** (128.38 MHz, Tol-d_8, 25 °C): δ 7.2 (bs). **^{29}Si{^{1}H} NMR** (79.49 MHz, Tol-d_8, 25 °C): δ – 10.8 (s).

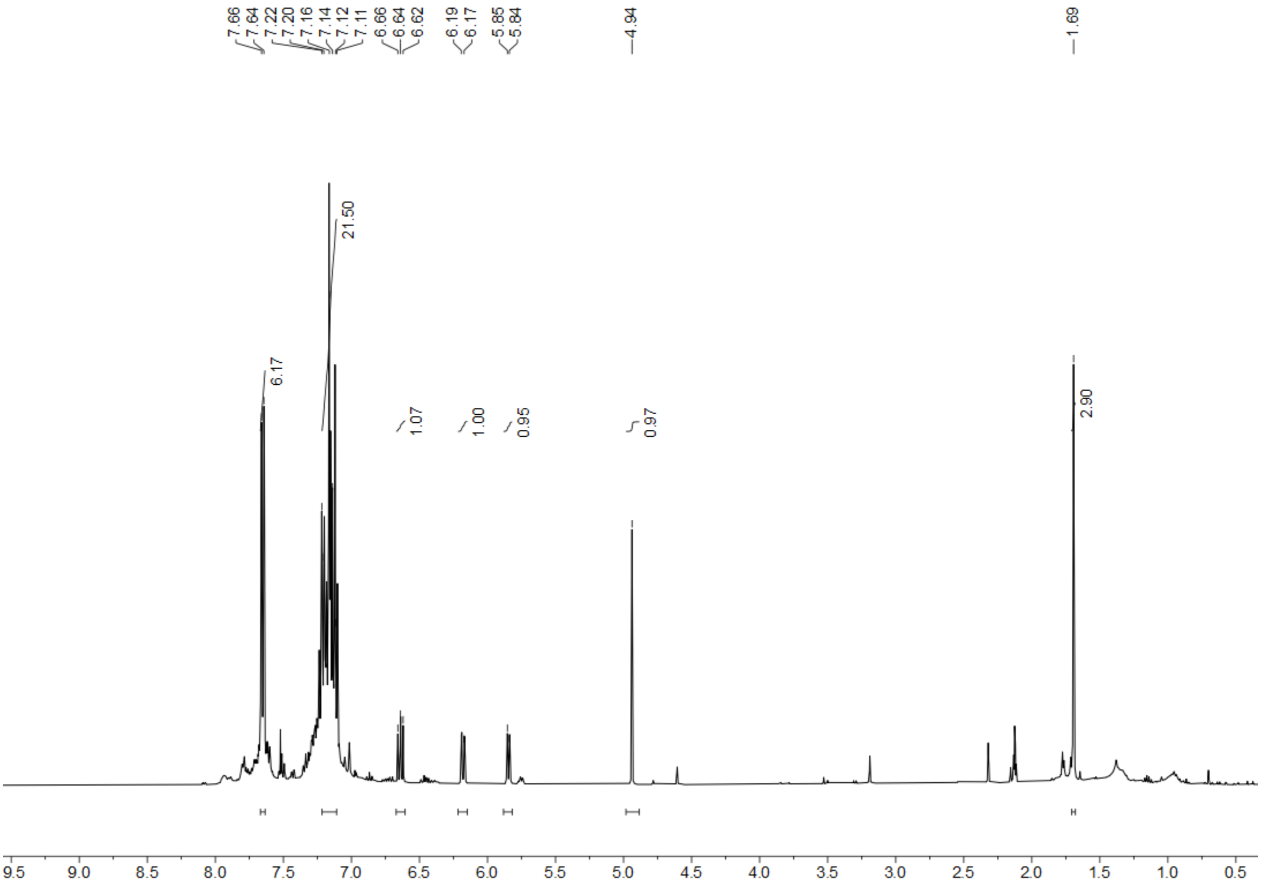

Figure S71. ^{1}H NMR spectrum (Tol-d_8, 298 K) of the toluene-d_8 filtrates containing compound **5**.

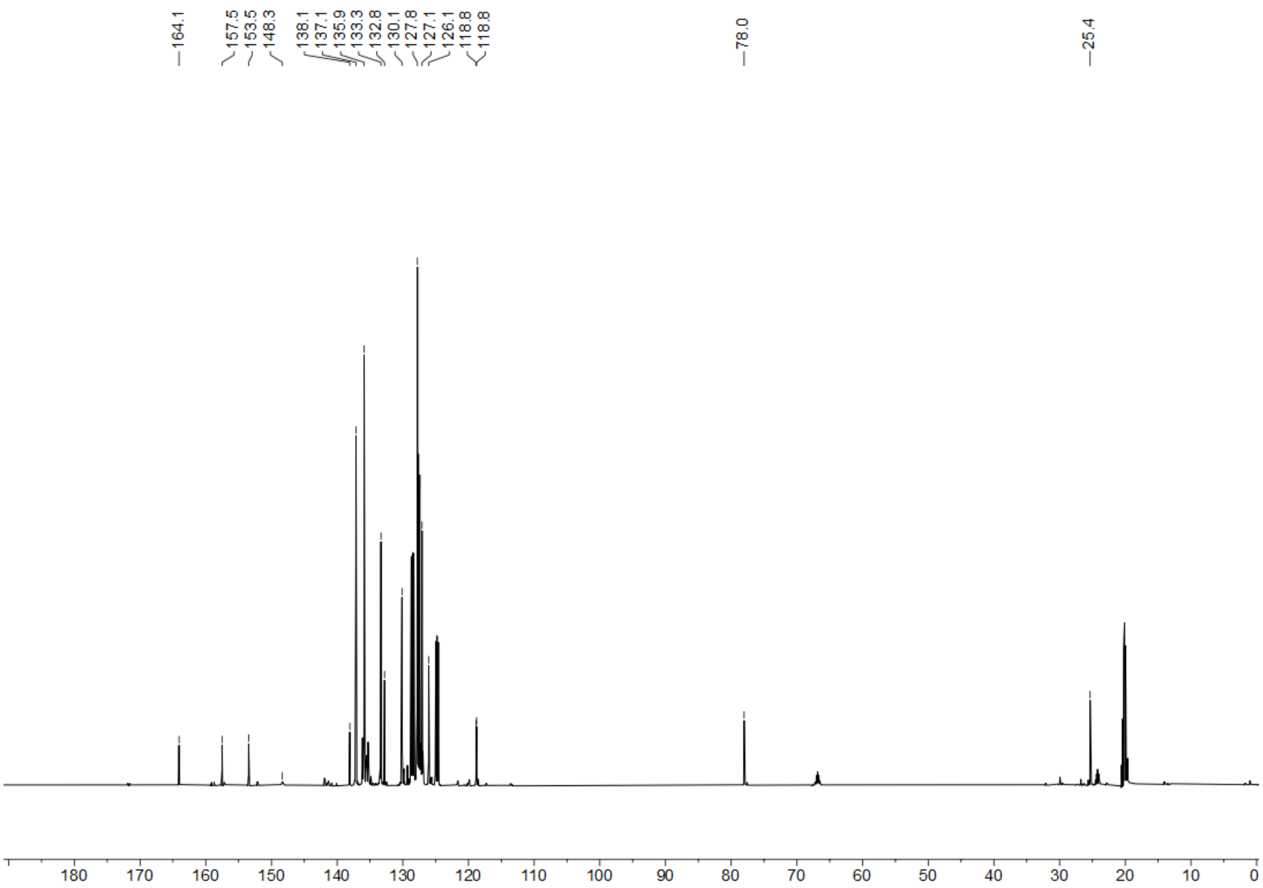

Figure S72. ^{13}C NMR spectrum (Tol-d_8, 298 K) of the toluene-d_8 filtrates containing compound **5**.

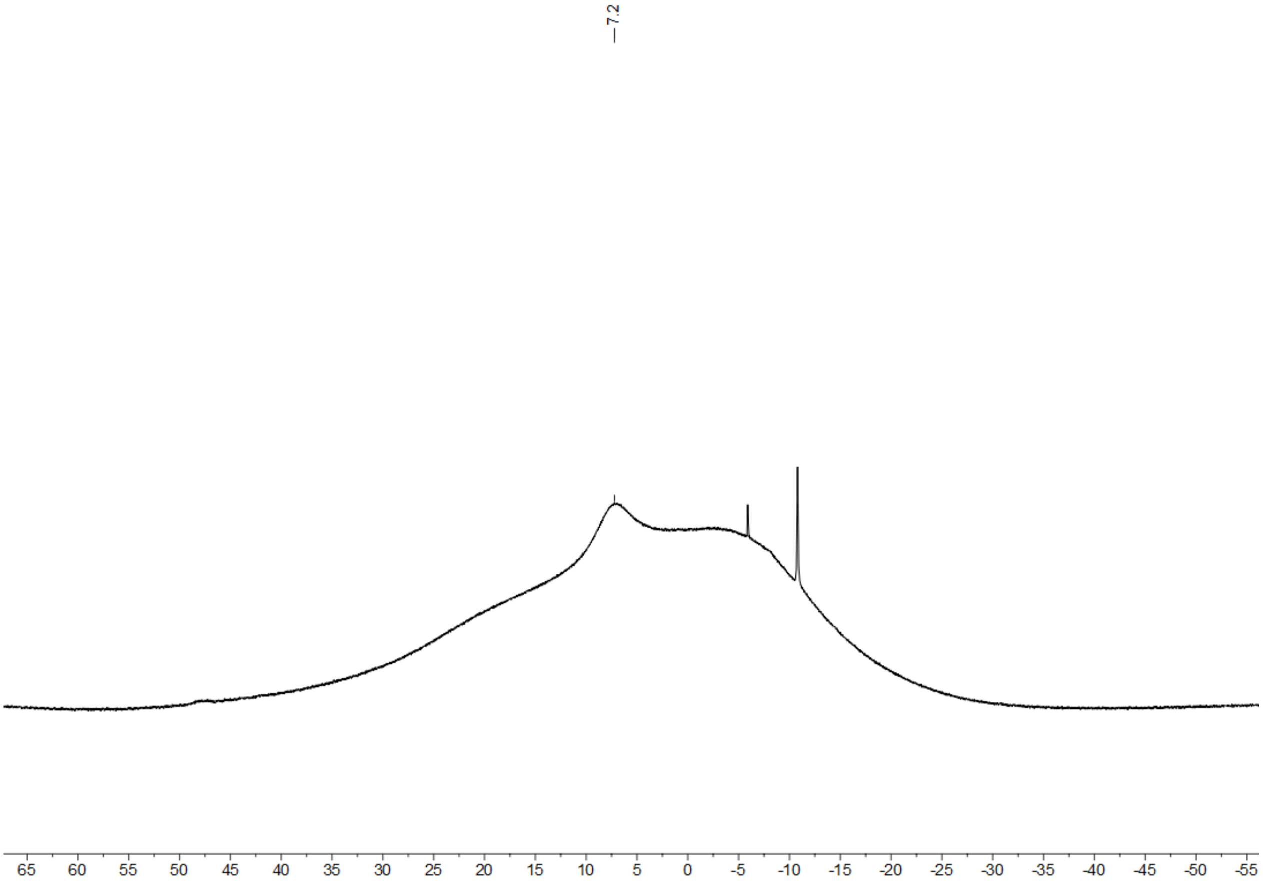

Figure S73. $^{11}B\{^1H\}$ NMR spectrum (Tol-d_8, 298 K) of the toluene-d_8 filtrates containing compound **5**.

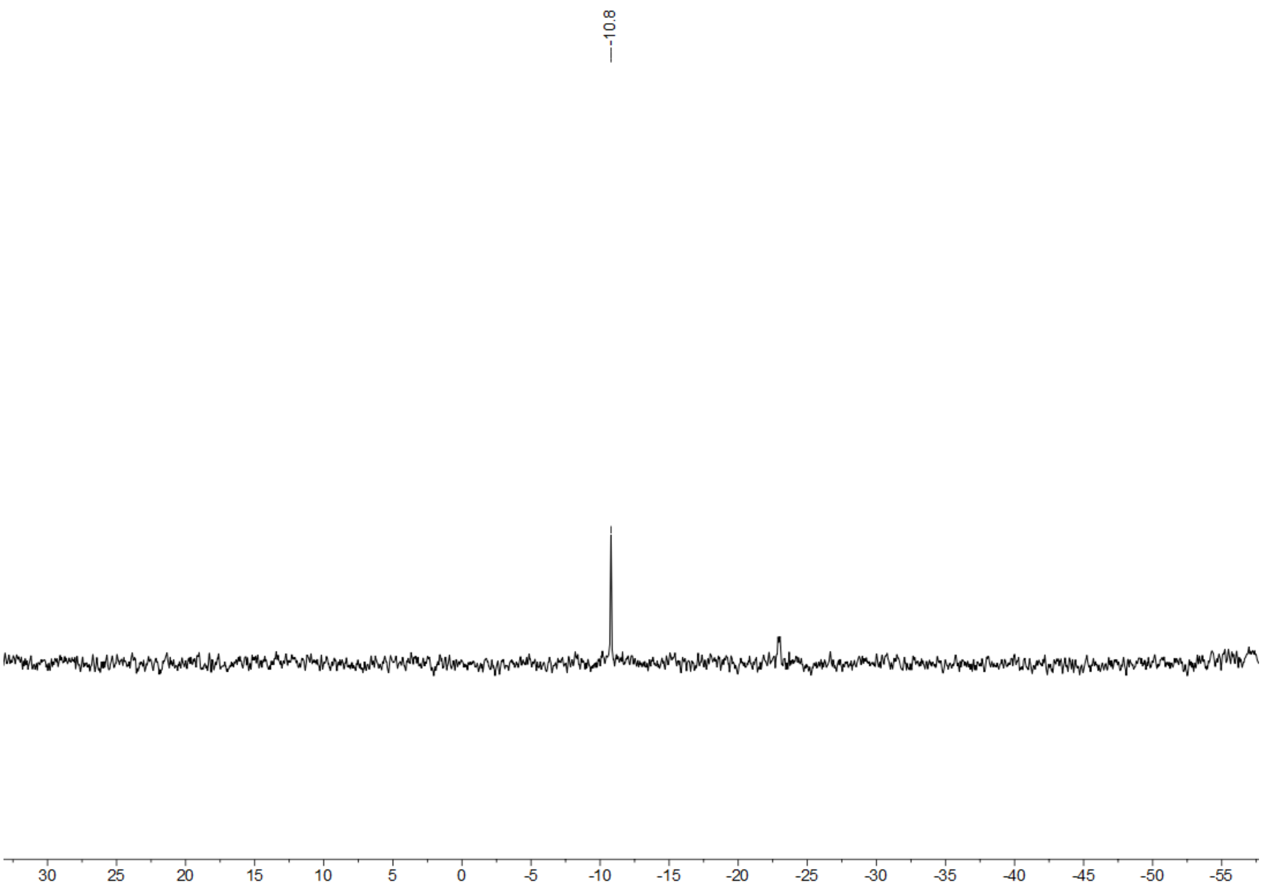

Figure S74. $^{29}Si\{^{1}H\}$ NMR spectrum (Tol-d_8, 298 K) of the toluene-d_8 filtrates containing compound **5**.

4. Single-Crystal X-Ray Diffraction Analysis

The crystals were selected and measured either on an Xcalibur Gemini Ultra diffractometer, equipped with a TitanS2 detector (**1**), an XtaLAB Synergy R, DW System, equipped with a HyPix-Arc 150 detector (**2**, **3**, **6**, **9**, ***Z*-11**, **12**), or on a SuperNova Dualflex diffractometer, equipped with a TitanS2 detector (**4**, **5**). The crystals were kept at T = 123.00(10) K during data collection. Data collection and reduction were performed with CrysAlisPro, Version 1.171.41.90a (**3**, **5**, **6**), Version 1.171.41.118a (**1**, **2**), Version 1.171.41.83a (**4**), Version 1.171.42.95a (***Z*-11**), and Version 1.171.43.36a (**9**, **12**).[135] For all compounds, a numerical absorption correction based on Gaussian integration over a multifaceted crystal model, and an empirical absorption correction using spherical harmonics as implemented in SCALE3 ABSPACK scaling algorithm was applied. Using Olex2,[136] the structures were solved with ShelXT[137] and a least-square refinement on F^2 was carried out with ShelXL.[138] All non-hydrogen atoms were refined anisotropically. Hydrogen atoms were located in idealized positions and refined isotropically according to the riding model. All figures were created with Mercury 3.10.3.[139]

Compound **1**: The asymmetric unit contains one molecule.
Compound **2**: The asymmetric unit contains two molecules.
Compound **3**: The asymmetric unit contains one molecule together with one molecule diethyl ether.
Compound **4**: The asymmetric unit contains two molecules.
Compound **5**: The asymmetric unit contains one molecule together with one molecule benzene.
Compound **6**: The asymmetric unit contains one molecule.

Compound **9**: The asymmetric unit contains two molecules. SADI and EADP restraints were used to model disorders. The squeeze tool was used for solvent masking.

Compound ***Z*-11**: The asymmetric unit contains one molecule. The squeeze tool was used for solvent masking.

Compound **12**: The asymmetric unit contains one molecule.

Table S1. Determination of the tetrahedral character (THC) at the boron atom in compounds **3** and **4**.[140]

Compound **3**		Compound **4**			
Bond Angle (°)	109.5 – θ (°)	Bond Angle (°)	Bond Angle (°)	Average Bond Angle (°)	109.5 – θ (°)
116.35(9)	– 6.85	117.4(2)	117.4(2)	117.4	– 7.9
120.57(9)	– 11.07	117.8(2)	117.7(2)	117.75	– 8.25
110.81(9)	– 1.37	113.3(2)	114.6(2)	113.95	– 4.45
111.37(9)	– 1.87	110.0(2)	109.7(2)	109.85	– 0.35
110.69(9)	– 1.19	111.79(19)	110.7(2)	111.245	– 1.745
81.77(7)	27.73	80.64(16)	80.32(16)	80.48	29.02

THC [%] = $\left[1 - \frac{\sum_{n=1-6} |109.5-\theta_n|°}{27°}\right] \times 100$

THC (**3**) = 80%

THC (**4**) = 77%

Table S2. Crystal data and structure refinement of compounds **1** – **3**.

Compound	1	2	3
Data set (internal naming)	Rx_471	EI04	Rx_750
CCDC number	2483842	2483848	2483849
Formula	$C_{25}H_{23}NSi$	$C_{37}H_{46}LiNO_3Si$	$C_{41}H_{42}BNOSi$
M / g·mol^{-1}	365.53	587.78	603.65
T / K	123.00(10)	123.00(10)	123.01(10)
Crystal system	Triclinic	Triclinic	Triclinic
Space group	$P\bar{1}$	$P\bar{1}$	$P\bar{1}$
a / Å	9.5125(8)	11.04840(10)	10.27430(10)
b / Å	9.8193(8)	13.97560(10)	10.96090(10)
c / Å	11.5679(7)	22.5219(2)	17.1548(2)
α / °	98.804(6)	77.0270(10)	96.8520(10)
β / °	95.874(6)	85.8710(10)	105.1520(10)
γ / °	107.525(7)	76.0200(10)	112.4510(10)
V / Å^3	1005.51(14)	3287.84(5)	1670.60(3)
Z	2	4	2
Z'	1	2	1
Flack parameter	-	-	-
ρ / g·cm^{-3}	1.207	1.187	1.200
μ / mm^{-1}	1.075	0.902	0.862
Crystal size / mm^3	0.62 × 0.20 × 0.17	0.57 × 0.35 × 0.09	0.36 × 0.33 × 0.21
λ / Å	1.54184	1.54184	1.54184
Radiation type	Cu K_α	Cu K_α	Cu K_α
θ range / °	3.916 – 67.113	3.336 – 73.726	2.753 – 75.002
Reflections, collected	22488	95381	65180
Reflections, independent	3568	12636	6657
Reflections with I > 2(I)	3357	11432	6498
R_{int}	0.0484	0.0276	0.0176
Parameters	245	777	409
Restraints	0	0	0
GooF	1.070	1.075	1.036
wR_2 (all data)	0.1002	0.1142	0.0889
wR_2	0.0980	0.1120	0.0885
R_1 (all data)	0.0398	0.0469	0.0345
R_1	0.0377	0.0433	0.0340
$\Delta\rho_{fin}$ (max / min) / e·Å^{-3}	0.28 / –0.32	0.40 / –0.54	0.28 / –0.28

Table S3. Crystal data and structure refinement of compounds **4** – **6**.

Compound	4	5	6
Data set (internal naming)	Rx_11_Fron	Rx_813	Rx_647
CCDC number	2483845	2483846	2483844
Formula	$C_{45}H_{56}BNSi$	$C_{44}H_{38}BNO_2Si$	$C_{46}H_{56}BNO_2Si$
M / g·mol^{-1}	649.80	651.65	693.81
T / K	123.00(10)	123.00(10)	123.00(10)
Crystal system	Triclinic	Monoclinic	Orthorhombic
Space group	$P1$	$P2_1/c$	$P2_12_12_1$
a / Å	10.0027(2)	13.73170(10)	10.99060(10)
b / Å	10.5867(3)	11.28590(10)	16.67290(10)
c / Å	18.6014(4)	22.6184(2)	20.59080(10)
α / °	79.141(2)	90	90
β / °	86.214(2)	97.2270(10)	90
γ / °	74.261(2)	90	90
V / Å^3	1861.77(8)	3477.43(5)	3773.16(5)
Z	2	4	4
Z'	2	1	1
Flack parameter	0.009(16)	-	–0.003(5)
ρ / g·cm^{-3}	1.159	1.245	1.221
μ / mm^{-1}	0.781	0.894	0.844
Crystal size / mm^3	0.17 × 0.14 × 0.11	0.67 × 0.57 × 0.31	0.24 × 0.23 × 0.14
λ / Å	1.54184	1.54184	1.54184
Radiation type	Cu K_α	Cu K_α	Cu K_α
θ range / °	4.409 – 66.989	3.940 – 67.064	3.411 – 73.226
Reflections, collected	49357	71446	38968
Reflections, independent	12852	6180	7469
Reflections with I > 2(I)	12552	5852	7293
R_{int}	0.0479	0.0617	0.0190
Parameters	879	459	467
Restraints	3	0	0
GooF	1.026	1.022	1.025
wR_2 (all data)	0.0981	0.1096	0.0703
wR_2	0.0969	0.1069	0.0700
R_1 (all data)	0.0381	0.0427	0.0275
R_1	0.0373	0.0407	0.0268
$\Delta\rho_{fin}$ (max / min) / e·Å^{-3}	0.34 / –0.22	0.36 / –0.37	0.21 / –0.21

Table S4. Crystal data and structure refinement of compounds **9**, ***Z*-11**, and **12**.

**Compound**	**9**	***Z*-11**	**12**
Data set (internal naming)	Rx_1014	Rx_1047	Rx_1070
CCDC number	2483847	2483843	2483841
Formula	$C_{41}H_{36}BNO_3Si$	$C_{29}H_{24}BNO_2$	$C_{20}H_{18}BNO_2$
M / g·mol^{-1}	629.61	429.30	315.16
T / K	123.00(10)	123.00(10)	123.00(10)
Crystal system	Triclinic	Triclinic	Orthorhombic
Space group	$P\bar{1}$	$P\bar{1}$	$P2_12_12_1$
a / Å	9.83900(10)	8.1975(2)	7.9337(2)
b / Å	19.4583(4)	10.5943(2)	14.4860(4)
c / Å	20.3389(2)	14.9362(2)	14.7676(5)
α / °	74.0160(10)	94.5670(10)	90
β / °	84.2570(10)	90.985(2)	90
γ / °	76.0170(10)	91.974(2)	90
V / Å^3	3630.07(9)	1292.02(4)	1697.20(9)
Z	4	2	4
Z'	2	1	1
Flack parameter	-	-	–0.01(11)
ρ / g·cm^{-3}	1.152	1.104	1.233
μ / mm^{-1}	0.860	0.533	0.620
Crystal size / mm^3	0.15 × 0.11 × 0.03	0.19 × 0.13 × 0.09	0.19 × 0.03 × 0.02
λ / Å	1.54184	1.54184	1.54184
Radiation type	Cu K_α	Cu K_α	Cu K_α
θ range / °	2.423 – 75.292	2.969 – 75.034	4.275 – 74.892
Reflections, collected	77413	27178	18097
Reflections, independent	14636	5189	3461
Reflections with I > 2(I)	11143	4684	2925
R_{int}	0.0362	0.0311	0.0349
Parameters	843	300	218
Restraints	3	0	0
GooF	1.077	1.063	1.048
wR_2 (all data)	0.1614	0.1213	0.0940
wR_2	0.1509	0.1178	0.0886
R_1 (all data)	0.0700	0.0453	0.0495
R_1	0.0540	0.0416	0.0378
$\Delta\rho_{fin}$ (max / min) / e·Å^{-3}	0.87 / –0.88	0.24 / –0.22	0.14 / –0.16

Compound 1

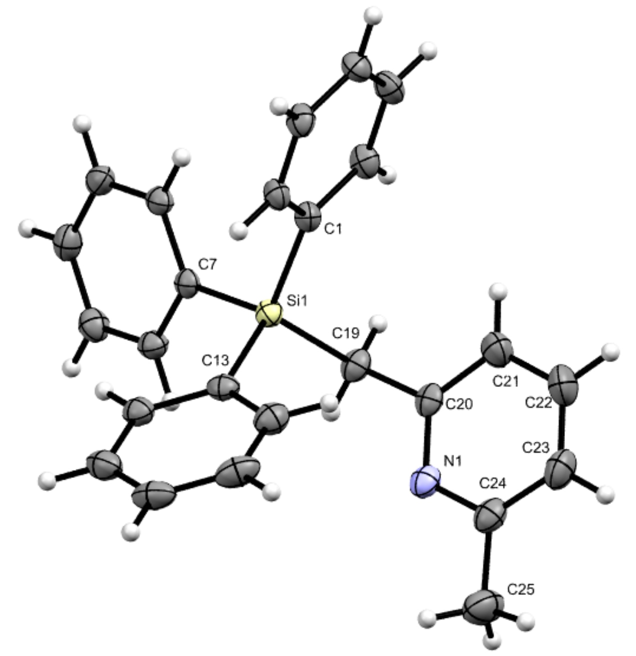

Figure S75. Molecular structure of compound **1** in the crystal (displacement ellipsoids of non-hydrogen atoms set at the 50% probability level).

Table S5. Selected bond lengths / Å and selected bond angles / ° of compound **1**.

Selected bond lengths / Å		Selected bond angles / °	
Si1–C1	1.871(1)	C1–Si1–C19	111.28(7)
Si1–C7	1.882(1)	C7–Si1–C19	105.42(7)
Si1–C13	1.881(2)	C13–Si1–C19	111.30(7)
Si1–C19	1.890(2)	Si1–C19–C20	117.2(1)
C19–C20	1.505(2)	C19–C20–N1	116.7(1)
N1–C20	1.344(2)	C19–C20–C21	121.4(1)
C20–C21	1.385(2)	C20–C21–C22	118.7(2)
C21–C22	1.382(2)	C21–C22–C23	119.9(2)
C22–C23	1.381(3)	C22–C23–C24	118.2(2)
C23–C24	1.382(2)	C23–C24–N1	122.5(2)
N1–C24	1.348(2)	C20–N1–C24	118.7(1)

Compound 2

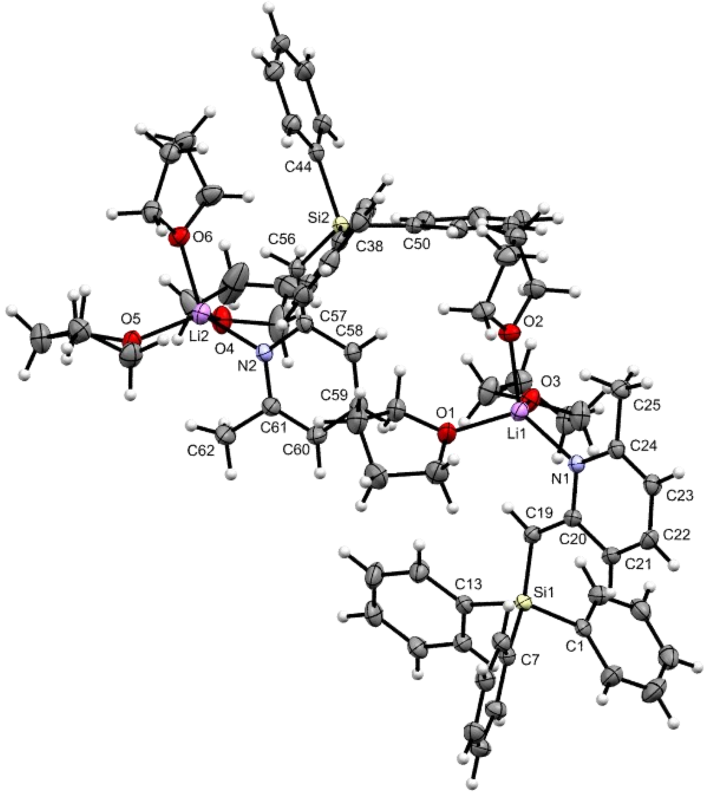

Figure S76. Molecular structure of compound **2** in the crystal (displacement ellipsoids of non-hydrogen atoms set at the 50% probability level).

Table S6. Selected bond lengths / Å and selected bond angles / ° of compound **2**.

Selected bond lengths / Å		Selected bond angles / °	
Si1–C1	1.898(2)	C1–Si1–C19	113.94(7)
Si1–C7	1.896(2)	C7–Si1–C19	114.66(7)
Si1–C13	1.894(2)	C13–Si1–C19	110.51(7)
Si1–C19	1.806(2)	Si1–C19–C20	126.1(1)
C19–C20	1.395(2)	C19–C20–C21	121.6(1)
C20–C21	1.443(2)	N1–C20–C21	117.6(1)
C21–C22	1.361(2)	C19–C20–N1	120.8(1)
C22–C23	1.403(2)	C20–C21–C22	121.2(1)
C23–C24	1.381(2)	C21–C22–C23	120.0(1)
N1–C24	1.351(2)	C22–C23–C24	117.5(1)
N1–C20	1.391(2)	C23–C24–N1	124.3(1)
Li1–N1	2.036(3)	C20–N1–C24	119.3(1)
Li1–O1	1.939(2)	O1–Li1–O2	96.2(1)
Li1–O2	1.987(3)	O2–Li1–O3	94.7(1)
Li1–O3	1.910(3)	O3–Li1–O1	113.5(1)
		C20–N1–Li1	117.9(1)
		C24–N1–Li1	122.8(1)

Compound 3

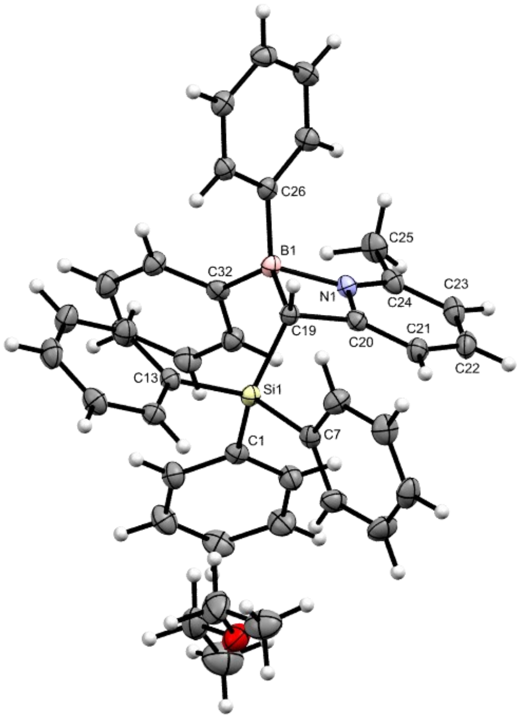

Figure S77. Molecular structure of compound **3** in the crystal (displacement ellipsoids of non-hydrogen atoms set at the 50% probability level).

Table S7. Selected bond lengths / Å and selected bond angles / ° of compound **3**.

Selected bond lengths / Å		Selected bond angles / °	
Si1–C1	1.876(1)	C1–Si1–C19	113.94(5)
Si1–C7	1.8878(9)	C7–Si1–C19	105.95(5)
Si1–C13	1.877(1)	C13–Si1–C19	109.47(5)
Si1–C19	1.878(1)	Si1–C19–C20	114.85(8)
B1–C19	1.708(2)	Si1–C19–B1	127.49(8)
C19–C20	1.499(2)	B1–C19–C20	85.04(9)
N1–B1	1.643(2)	N1–C20–C19	100.50(9)
N1–C20	1.352(1)	B1–N1–C20	92.50(9)
C20–C21	1.387(2)	N1–B1–C19	81.77(8)
C21–C22	1.392(2)	C26–B1–N1	110.69(9)
C22–C23	1.391(2)	C26–B1–C19	110.81(9)
C23–C24	1.390(2)	C32–B1–N1	111.37(9)
N1–C24	1.348(2)	C32–B1–C26	116.35(9)
		C32–B1–C19	120.57(9)
		Si1–C19–B1	127.49(8)
		C19–C20–C21	138.5(1)
		C20–C21–C22	117.1(1)
		C21–C22–C23	120.7(1)
		C22–C23–C24	120.5(1)
		C23–C24–N1	117.4(1)
		C20–N1–C24	123.4(1)
		N1–C20–C21	120.9(1)

Compound 4

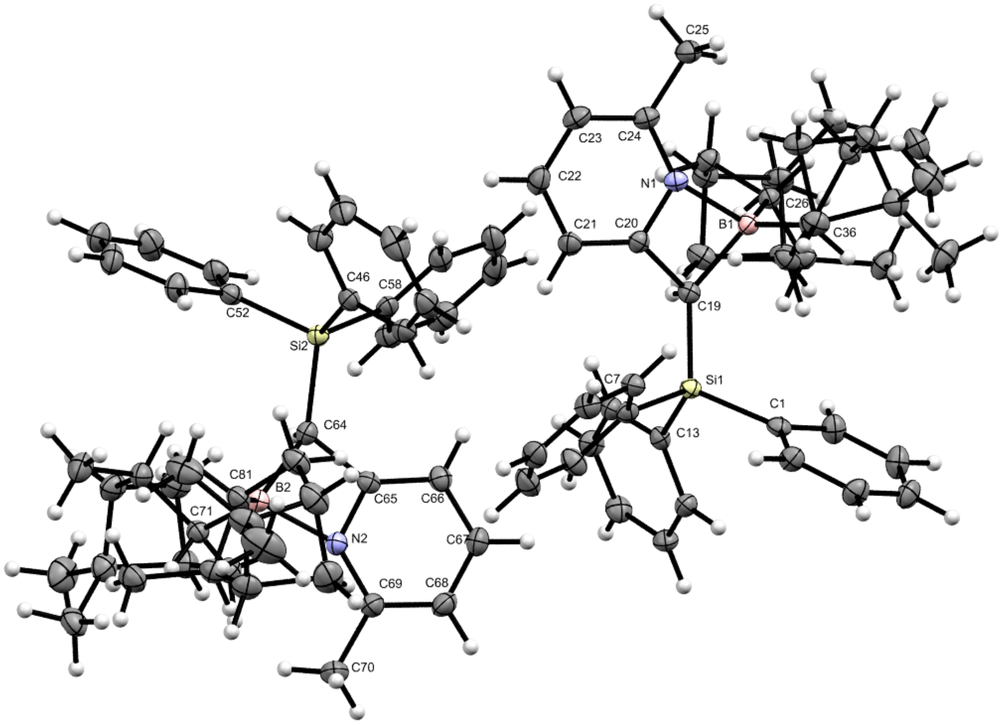

Figure S78. Molecular structure of compound **4** in the crystal (displacement ellipsoids of non-hydrogen atoms set at the 50% probability level).

Table S8. Selected bond lengths / Å and selected bond angles / ° of compound **4**.

Selected bond lengths / Å		Selected bond angles / °	
Si1–C1	1.878(3)	C1–Si1–C19	116.3(1)
Si1–C7	1.897(2)	C7–Si1–C19	114.1(1)
Si1–C13	1.893(3)	C13–Si1–C19	105.6(1)
Si1–C19	1.882(2)	Si1–C19–C20	121.4(2)
C19–C20	1.495(4)	C19–C20–C21	136.4(2)
C20–C21	1.379(3)	N1–C20–C21	121.8(2)
C21–C22	1.389(5)	C19–C20–N1	101.6(2)
C22–C23	1.391(4)	C20–C21–C22	117.7(3)
C23–C24	1.387(4)	C21–C22–C23	119.8(3)
N1–C24	1.353(4)	C22–C23–C24	120.8(3)
N1–C20	1.352(3)	C23–C24–N1	118.3(3)
B1–N1	1.703(4)	C20–N1–C24	121.6(2)
B1–C26	1.632(3)	Si1–C19–B1	136.3(2)
B1–C19	1.709(4)	N1–B1–C19	80.6(2)
		C36–B1 N1	110.0(2)
		C36–B1–C19	117.8(2)
		C36–B1 C26	117.4(2)
		C26–B1–N1	111.8(2)
		C26–B1–C19	113.3(2)
		C64–B2–N2	80.3(2)
		C71–B2–N2	110.7(2)
		C71–B2–C64	114.6(2)
		C81–B2–N2	109.7(2)
		C81–B2–C64	117.7(2)
		C81–B2–C71	117.4(2)
		B1–N1–C20	91.1(2)
		C20–C19–B1	86.2(2)
		N1–C20–C19	101.6(2)

Compound 5

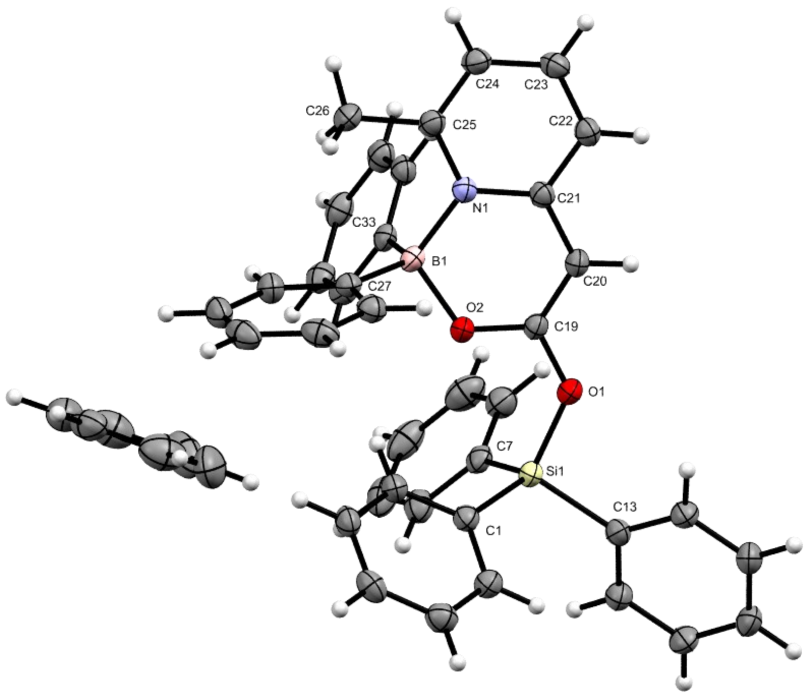

Figure S79. Molecular structure of compound **5** in the crystal (displacement ellipsoids of non-hydrogen atoms set at the 50% probability level).

Table S9. Selected bond lengths / Å and selected bond angles / ° of compound **5**.

Selected bond lengths / Å		Selected bond angles / °	
Si1–C1	1.859(1)	C1–Si1–O1	114.64(6)
Si1–C7	1.862(2)	C7–Si1–O1	109.09(6)
Si1–C13	1.863(1)	C13–Si1–O1	99.86(6)
Si1–O1	1.681(1)	Si1–O1–C19	129.24(9)
O1–C19	1.333(2)	O1–C19–O2	115.3(1)
O2–C19	1.303(2)	C19–C20–C21	121.5(1)
C19–C20	1.353(2)	C20–C21–N1	120.3(1)
N1–C21	1.375(2)	C21–N1–B1	119.4(1)
C20–C21	1.424(2)	N1–B1–O2	108.1(1)
C21–C22	1.405(2)	B1–O2–C19	122.7(1)
C22–C23	1.366(2)	O2–C19–C20	123.7(1)
C23–C24	1.391(2)	C21–C22–C23	120.6(1)
C24–C25	1.383(2)	C22–C23–C24	118.9(1)
N1–C25	1.371(2)	C23–C24–C25	120.3(1)
B1–O2	1.529(2)	C24–C25–N1	120.8(1)
B1–N1	1.623(2)	C21–N1–C25	119.4(1)
		C22–C21–N1	120.0(1)

Compound 6

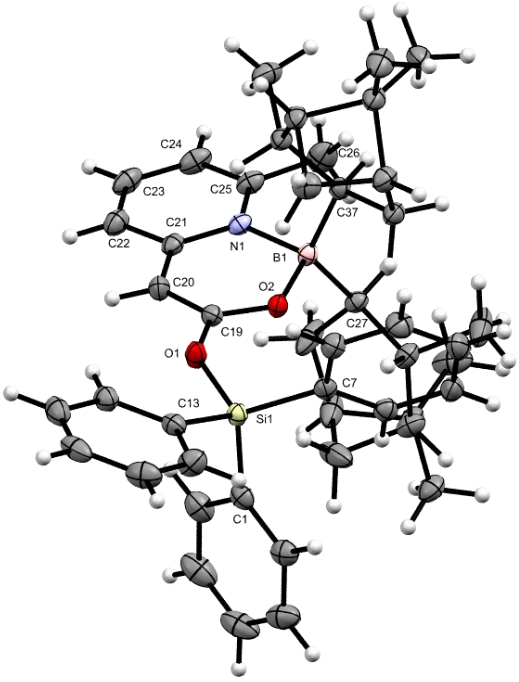

Figure S80. Molecular structure of compound **6** in the crystal (displacement ellipsoids of non-hydrogen atoms set at the 50% probability level).

Table S10. Selected bond lengths / Å and selected bond angles / ° of compound **6**.

Selected bond lengths / Å		Selected bond angles / °	
Si1–C1	1.867(2)	C1–Si1–O1	107.52(7)
Si1–C7	1.856(2)	C7–Si1–O1	115.47(7)
Si1–C13	1.869(2)	C13–Si1–O1	100.95(7)
Si1–O1	1.669(1)	Si1–O1–C19	136.0(1)
O1–C19	1.337(2)	O1–C19–O2	116.4(1)
C19–O2	1.290(2)	C19–C20–C21	121.6(2)
C19–C20	1.356(2)	C20–C21–N1	120.8(2)
N1–C21	1.370(2)	C21–N1–B1	120.1(1)
C20–C21	1.420(2)	N1–B1–O2	107.3(1)
C21–C22	1.409(3)	B1–O2–C19	125.0(1)
C22–C23	1.367(3)	O2–C19–C20	124.6(2)
C23–C24	1.387(3)	C21–C22–C23	120.5(2)
C24–C25	1.381(3)	C22–C23–C24	118.8(2)
N1–C25	1.372(2)	C23–C24–C25	120.3(2)
B1–O2	1.535(2)	C24–C25–N1	121.5(2)
B1–N1	1.679(2)	C21–N1–C25	118.5(1)
		C22–C21–N1	120.5(2)

Compound 9

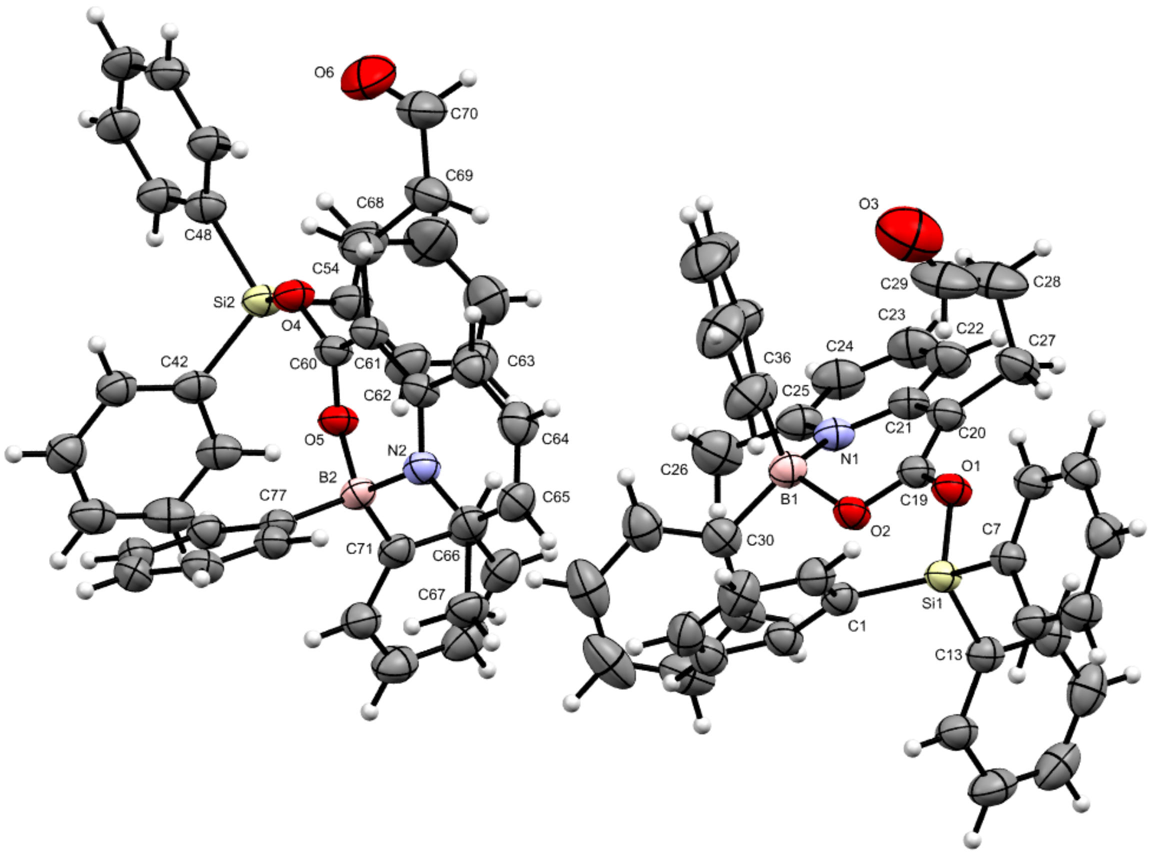

Figure S81. Molecular structure of compound **9** in the crystal (displacement ellipsoids of non-hydrogen atoms set at the 50% probability level).

Table S11. Selected bond lengths / Å and selected bond angles / ° of compound **9**.

Selected bond lengths / Å		Selected bond angles / °	
Si1–C1	1.861(2)	C1–Si1–O1	114.34(9)
Si1–C7	1.860(2)	C7–Si1–O1	101.62(9)
Si1–C13	1.863(2)	C13–Si1–O1	106.00(9)
Si1–O1	1.678(2)	Si1–O1–C19	126.0(1)
C19–O1	1.342(2)	O1–C19–O2	115.3(2)
C19–O2	1.309(3)	O1–C19–C20	121.5(2)
C19–C20	1.363(3)	O2–C19–C20	123.2(2)
C20–C21	1.436(3)	C19–C20–C21	118.4(2)
C21–C22	1.403(3)	C20–C21–N1	120.7(2)
C22–C23	1.364(4)	C21–N1–B1	114.7(2)
C23–C24	1.383(5)	N1–B1–O2	104.7(2)
C24–C25	1.386(3)	B1–O2–C19	115.8(2)
N1–C25	1.370(3)	N1–C21–C22	119.4(2)
N1–C21	1.382(3)	C21–C22–C23	120.6(2)
B1–N1	1.629(3)	C22–C23–C24	119.6(2)
B1–O2	1.515(3)	C23–C24–C25	120.0(2)
B1–C30	1.607(4)	C24–C25–N1	120.6(2)
B1–C36	1.615(3)	C25–N1–C21	119.7(2)
C20–C27	1.511(4)	C25–N1–B1	124.7(2)
C27–C28	1.526(4)	C20–C27–C28	111.6(2)
C28–C29	1.487(5)	C27–C28–C29	109.6(3)
C29–O3	1.106(4)	C28–C29–O3	134.9(4)

Compound *Z*-11

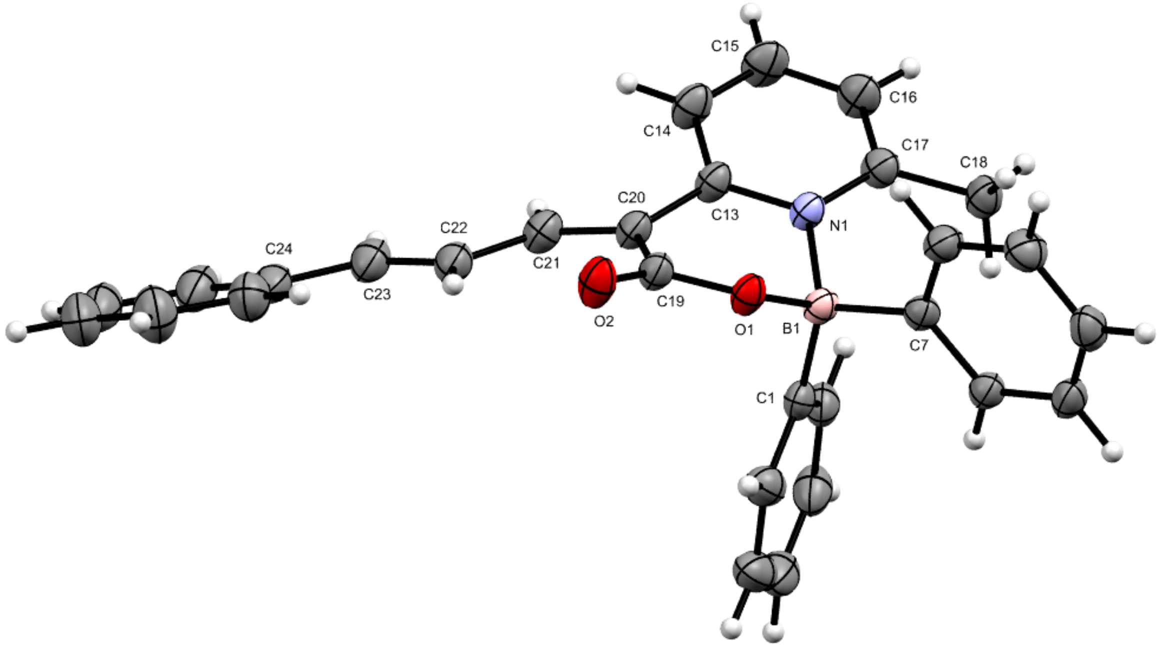

Figure S82. Molecular structure of compound ***Z*-11** in the crystal (displacement ellipsoids of non-hydrogen atoms set at the 50% probability level).

Table S12. Selected bond lengths / Å and selected bond angles / ° of compound **Z-11**.

Selected bond lengths / Å		Selected bond angles / °	
B1–C1	1.618(2)	C1–B1–C7	113.7(1)
B1–C7	1.619(2)	C1–B1–N1	106.59(9)
B1–N1	1.631(2)	C7–B1–N1	116.2(1)
B1–O1	1.496(2)	C1–B1–O1	112.6(1)
O1–C19	1.320(1)	C7–B1–O1	101.96(9)
O2–C19	1.213(1)	N1–B1–O1	105.52(9)
C19–C20	1.483(2)	B1–O1–C19	124.67(9)
N1–C13	1.369(2)	O1–C19–C20	114.5(1)
C13–C14	1.393(2)	O2–C19–C20	124.7(1)
C14–C15	1.378(2)	O1–C19–O2	120.8(1)
C15–C16	1.377(2)	C13–C20–C19	118.0(1)
C16–C17	1.389(2)	N1–C13–C20	120.0(1)
N1–C17	1.366(2)	B1–N1–C13	115.30(9)
C20–C21	1.363(2)	N1–C13–C14	120.6(1)
C13–C20	1.473(2)	C13–C14–C15	120.0(1)
		C14–C15–C16	118.9(1)
		C15–C16–C17	120.6(1)
		C13–N1–C17	119.5(1)
		C16–C17–N1	120.3(1)
		C19–C20–C21	122.1(1)

Compound 12

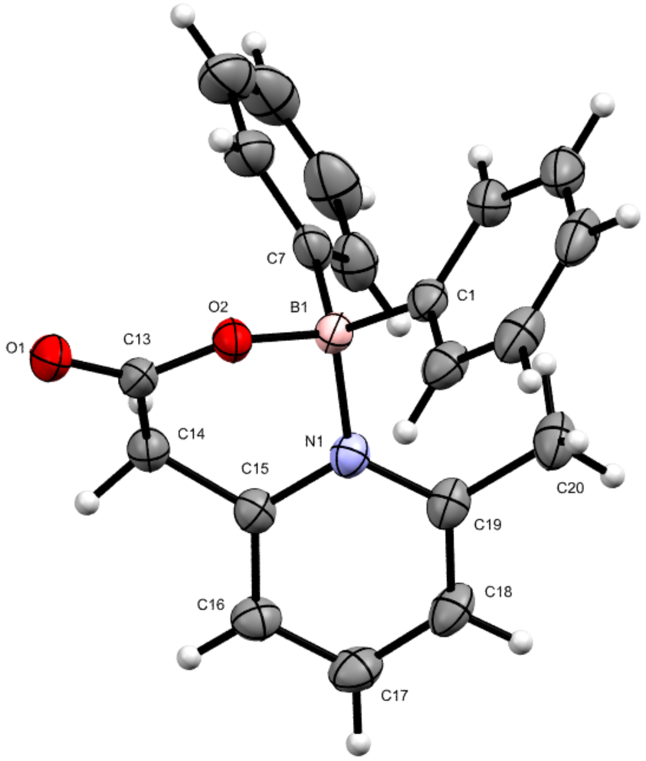

Figure S83. Molecular structure of compound **12** in the crystal (displacement ellipsoids of non-hydrogen atoms set at the 50% probability level).

Table S13. Selected bond lengths / Å and selected bond angles / ° of compound **12**.

Selected bond lengths / Å		Selected bond angles / °	
B1–C1	1.610(4)	C1–B1–C7	117.0(2)
B1–C7	1.614(4)	C1–B1–N1	113.6(2)
B1–N1	1.631(3)	C1–B1–O2	102.3(2)
B1–O2	1.510(3)	C7–B1–N1	107.8(2)
C13–O2	1.316(3)	C7–B1–O2	109.8(2)
C13–O1	1.214(3)	N1–B1–O2	105.6(2)
C13–C14	1.502(3)	B1–O2–C13	121.7(2)
C14–C15	1.497(3)	O2–C13–O1	122.2(2)
C15–C16	1.380(3)	O2–C13–C14	114.7(2)
C16–C17	1.380(4)	C13–C14–C15	114.3(2)
C17–C18	1.385(4)	O1–C13–C14	123.1(2)
C18–C19	1.379(3)	C14–C15–N1	117.9(2)
C19–C20	1.504(4)	C15–N1–B1	115.5(2)
		C14–C15–C16	120.3(2)
		N1–C15–C16	121.7(2)
		C15–C16–C17	119.4(2)
		C16–C17–C18	118.5(2)
		C17–C18–C19	121.1(2)
		C18–C19–N1	120.0(2)
		C15–N1–C19	119.0(2)
		B1–N1–C19	125.5(2)

5. Density Functional Theory (DFT) Calculations

Geometry optimization and additional harmonic vibrational frequency analyses of all structures were performed with the software package Gaussian 09 (Revision E.01)[91] on the M06-2X/6–311+G(d,p) or the M06-2X/6–31+G(d) level of theory without symmetry restrictions.[87-90] Solvation was modeled by applying the Polarizable Continuum Model (PCM) (solvent: toluene).[92] Additional single-point calculations were performed at the M06-2X/def2–QZVP, M06-2X/6–311+G(d,p), ωB97X-D/6–311+G(d,p), B3LYP/6–311+G(d,p), and B3LYP-D3/6–311+G(d,p) levels of theory on structures optimized at the M06-2X/6–311+G(d,p) level (PCM, toluene) using Gaussian 16 (Revision B.01). The GJF input files and the figures of the optimized structures were created with the program GaussView version 5.0.9.[141] For the ground state structures, the vibrational frequency analysis showed no imaginary frequency in the harmonical approximation. In case of transition-states, the vibrational frequency analysis showed exactly one imaginary frequency in the harmonical approximation. The relative energies (ΔG) of the computed structures are given based on the sum of electronic and thermal free energies (Gibbs energies) at 298.15 K in kcal mol^{-1}. The Hartree units were converted as follows: 1 Hartree = 2625.4995 kJ mol^{-1}.[142] The total electronic energies (SCF), the sums of electronic and zero-point energies (ZPE), and the sums of electronic and thermal free energies (Gibbs energies) at 298.15 K are summarized in Table S14. The Cartesian coordinates of the optimized structures can be found in Tables S17–S72.

Table S14. Total electronic energies (SCF), sums of electronic and zero-point energies (ZPE), and sums of electronic and thermal free energies (Gibbs energies) at 298.15 K for all optimized structures, obtained using Gaussian 09 (Revision E.01).

Optimized structure	Method/Basis	SCF [Hartree]	ZPE [Hartree]	Gibbs energies [Hartree]
**E_A**	M06-2X/6–311+G(d,p) (PCM, toluene)	–800.259376441	–799.972492	–800.016628
**$TS1_A$**	M06-2X/6–311+G(d,p) (PCM, toluene)	–800.229573501	–799.945198	–799.990784
**$I1_A$**	M06-2X/6–311+G(d,p) (PCM, toluene)	–800.249491361	–799.962757	–800.008838
**$TS2_A$**	M06-2X/6–311+G(d,p) (PCM, toluene)	–988.815707417	–988.515760	–988.564177
**$I2_A$**	M06-2X/6–311+G(d,p) (PCM, toluene)	–988.890987736	–988.587191	–988.634569
**$TS3_A$**	M06-2X/6–311+G(d,p) (PCM, toluene)	–988.850574605	–988.548044	–988.594729
**P_A**	M06-2X/6–311+G(d,p) (PCM, toluene)	–988.907465567	–988.603746	–988.652237
**E_B**	M06-2X/6–311+G(d,p) (PCM, toluene)	–2117.28171337	–2116.373440	–2116.449856
**$TS1_B$**	M06-2X/6–311+G(d,p) (PCM, toluene)	–2117.25689604	–2116.350048	–2116.427721
**$I1_B$**	M06-2X/6–311+G(d,p) (PCM, toluene)	–2117.25990503	–2116.353316	–2116.432615
**$TS2_B$**	M06-2X/6–311+G(d,p) (PCM, toluene)	–2305.83044075	–2304.910713	–2304.991657
**$I2_B$**	M06-2X/6–311+G(d,p) (PCM, toluene)	–2305.90059780	–2304.974597	–2305.051094
**$TS3_B$**	M06-2X/6–311+G(d,p) (PCM, toluene)	–2305.86233969	–2304.937329	–2305.014771
**P_B**	M06-2X/6–311+G(d,p) (PCM, toluene)	–2305.91391638	–2304.988537	–2305.070603
**I'_A**	M06-2X/6–311+G(d,p) (PCM, toluene)	–800.259640094	–799.972527	–800.017189

**TS'$_{A}$**	M06-2X/6–311+G(d,p) (PCM, toluene)	–988.829743823	–988.529063	–988.577048
**P'$_{A}$**	M06-2X/6–311+G(d,p) (PCM, toluene)	–988.842447036	–988.539454	–988.585925
**I'$_{B}$**	M06-2X/6–311+G(d,p) (PCM, toluene)	–2117.27073074	–2116.362310	–2116.439557
**TS'$_{B}$**	M06-2X/6–311+G(d,p) (PCM, toluene)	–2305.82840942	–2304.905556	–2304.984764
**P'$_{B}$**	M06-2X/6–311+G(d,p) (PCM, toluene)	–2305.83440066	–2304.909709	–2304.988109
**E$_{C}$**	M06-2X/6–311+G(d,p) (PCM, toluene)	–1208.89630514	–1208.506944	–1208.558279
**I1$_{C}$**	M06-2X/6–311+G(d,p) (PCM, toluene)	–1208.88304493	–1208.492651	–1208.543978
**I2$_{C}$**	M06-2X/6–311+G(d,p) (PCM, toluene)	–1397.52735917	–1397.121554	–1397.176028
**P$_{C}$**	M06-2X/6–311+G(d,p) (PCM, toluene)	–1397.55031904	–1397.145382	–1397.203138
**I'$_{C}$**	M06-2X/6–311+G(d,p) (PCM, toluene)	–1208.89348726	–1208.504245	–1208.556260
**TS'$_{C}$**	M06-2X/6–311+G(d,p) (PCM, toluene)	–1397.46320693	–1397.058208	–1397.110906
**P'$_{C}$**	M06-2X/6–311+G(d,p) (PCM, toluene)	–1397.48267679	–1397.075027	–1397.126398
**CO_2**	M06-2X/6–311+G(d,p) (PCM, toluene)	–188.576270837	–188.564325	–188.585656
***Z*-11**	M06-2X/6–311+G(d,p)	–1349.45718170	–1348.997410	–1349.057937
**Enamine**	M06-2X/6–311+G(d,p)	–406.637641708	–406.409467	–406.444299
**12**	M06-2X/6–311+G(d,p)	–1002.97119020	–1002.631785	–1002.680298
**13**	M06-2X/6–311+G(d,p)	–753.126213956	–752.777385	–752.823431
**14**	M06-2X/6–311+G(d,p)	–1756.09386571	–1755.402646	–1755.475522
**15**	M06-2X/6–311+G(d,p)	–1756.12072012	–1755.428784	–1755.501741
**Cinnamaldehyde**	M06-2X/6–31+G(d)	–422.796704717	–422.651818	–422.686555
**5**	M06-2X/6–31+G(d)	–1986.33767219	–1985.730305	–1985.802232
**TS$_{ZT}$**	M06-2X/6–31+G(d)	–2409.10994099	–2408.354488	–2408.436493
**16**	M06-2X/6–31+G(d)	–2409.16868758	–2408.410193	–2408.491954
**17$_{Z}$**	M06-2X/6–31+G(d)	–2409.15415117	–2408.396334	–2408.480033
**TS$_{Z}$**	M06-2X/6–31+G(d)	–2409.11729559	–2408.364563	–2408.447040
***Z*-11**	M06-2X/6–31+G(d)	–1349.14559309	–1348.683196	–1348.743572
**17$_{E}$**	M06-2X/6–31+G(d)	–2409.15349261	–2408.394808	–2408.474769
**TS$_{E}$**	M06-2X/6–31+G(d)	–2409.11231205	–2408.359426	–2408.440880
***E*-11**	M06-2X/6–31+G(d)	–1349.14408053	–1348.682022	–1348.742524
**Ph_3SiOH**	M06-2X/6–31+G(d)	–1059.99263171	–1059.702681	–1059.752135
**Cinnamaldehyde**	M06-2X/6–311+G(d,p)	–422.898922682	–422.754818	–422.789515
**5**	M06-2X/6–311+G(d,p)	–1986.75266318	–1986.148405	–1986.220805
**TS$_{ZT}$**[a]	M06-2X/6–311+G(d,p)	–2409.62604767	–2408.874764	–2408.957156
**16**	M06-2X/6–311+G(d,p)	–2409.68418755	–2408.930656	–2409.014181
**17$_{Z}$**	M06-2X/6–311+G(d,p)	–2409.67421404	–2408.920136	–2409.003939

**TS*Z***	M06-2X/6–311+G(d,p)	–2409.63857890	–2408.890673	–2408.968810
***Z*-11**	M06-2X/6–311+G(d,p)	–1349.45718170	–1348.997410	–1349.057937
**17*E***	M06-2X/6–311+G(d,p)	–2409.67433928	–2408.919787	–2408.999844
**TS*E***	M06-2X/6–311+G(d,p)	–2409.63477784	–2408.886059	–2408.967227
***E*-11**	M06-2X/6–311+G(d,p)	–1349.45584138	–1348.996328	–1349.056776
**Ph_3SiOH**	M06-2X/6–311+G(d,p)	–1060.20261525	–1059.913965	–1059.963298

[a] Single-point calculation on the geometry optimized at the M06-2X/6–31+G(d) level of theory.

Table S15. Total electronic energies (SCF, in Hartree) from single-point calculations on structures optimized at the M06-2X/6–311+G(d,p) level of theory (PCM, toluene), obtained using Gaussian 16 (Revision B.01).

Structure	M06-2X/ def2–QZVP	M06-2X/ 6–311+G(d,p)	ωB97X-D/ 6–311+G(d,p)	B3LYP/ 6–311+G(d,p)	B3LYP-D3/ 6–311+G(d,p)
**E_A**	–800.376707056	–800.259317491	–800.359371523	–800.543820198	–800.575728140
**$TS1_A$**	–800.348543795	–800.229641959	–800.327476750	–800.520289466	–800.550675916
**$I1_A$**	–800.368482268	–800.249524127	–800.345018919	–800.538754040	–800.567715587
**$TS2_A$**	–988.967345803	–988.815789268	–988.913384097	–989.165627453	–989.205720344
**$I2_A$**	–989.043119952	–988.891042995	–988.989076188	–989.229919185	–989.272017500
**$TS3_A$**	–989.008079504	–988.850618087	–988.944709872	–989.191822172	–989.229731319
**P_A**	–989.067504231	–988.907534997	–988.999389133	–989.253339090	–989.287671678
**E_B**	–2117.64567390	–2117.28145072	–2117.54242863	–2118.06372284	–2118.22964938
**$TS1_B$**	–2117.62325449	–2117.25672324	–2117.51471208	–2118.04964902	–2118.21016883
**$I1_B$**	–2117.62655248	–2117.25971696	–2117.51455205	–2118.05533344	–2118.21088097
**$TS2_B$**	–2306.22808058	–2305.83054230	–2306.08972650	–2306.67616275	–2306.85454367
**$I2_B$**	–2306.29807403	–2305.90052143	–2306.15628393	–2306.73287250	–2306.91206314
**$TS3_B$**	–2306.26570830	–2305.86249801	–2306.11233117	–2306.70300414	–2306.87171487
**P_B**	–2306.32035997	–2305.91360054	–2306.16189404	–2306.76572445	–2306.92598552
**I'_A**	–800.376233076	–800.259632241	–800.358837584	–800.543900595	–800.577208062
**TS'_A**	–988.980034877	–988.829668742	–988.932062847	–989.175227708	–989.216574236
**P'_A**	–988.995045065	–988.842576133	–988.943044987	–989.182530384	–989.223485860
**I'_B**	–2117.63452415	–2117.27072317	–2117.52942906	–2118.05873817	–2118.22078616
**TS'_B**	–2306.22535154	–2305.82832688	–2306.09177056	–2306.67252988	–2306.84973431
**P'_B**	–2306.23223500	–2305.83423869	–2306.09358084	–2306.67023201	–2306.84796439
**CO_2**	–188.611692795	–188.576271267	–188.582546915	–188.648110047	–188.648283445

Table S16. Energies (Δ*E*, in kcal mol⁻¹) derived from total electronic energies (SCF) obtained from single-point calculations on structures optimized at the M06-2X/6–311+G(d,p) level of theory (PCM, toluene) using Gaussian 16 (Revision B.01). Reported are the computed energies for the BLC pathway (red/blue) and the B/N-FLP pathway (gray/green) shown in the main article, evaluated with the following methods: M06-2X/def2–QZVP, M06-2X/6–311+G(d,p), ωB97X-D/6–311+G(d,p), B3LYP/6–311+G(d,p), and B3LYP-D3/6–311+G(d,p).

Structure	M06-2X/ def2–QZVP	M06-2X/ 6–311+G(d,p)	ωB97X-D/ 6–311+G(d,p)	B3LYP/ 6–311+G(d,p)	B3LYP-D3/ 6–311+G(d,p)
**E_A**	0	0	0	0	0
**$TS1_A$**	17.7	18.6	20.1	14.8	15.8
**$I1_A$**	5.3	6.2	9.1	3.1	5.0
**$TS2_A$**	13.1	12.4	17.9	16.5	11.5
**$I2_A$**	–34.4	–34.9	–29.6	–23.9	–30.1
**$TS3_A$**	–12.4	–9.3	–1.7	0.0	–3.6
**P_A**	–49.7	–45.2	–36.1	–38.5	–39.9
**E_B**	0	0	0	0	0
**$TS1_B$**	14.1	15.5	17.4	8.8	12.2
**$I1_B$**	12.0	13.6	17.4	5.3	11.7
**$TS2_B$**	18.4	17.0	22.2	22.5	14.6
**$I2_B$**	–25.6	–26.8	–19.6	–13.1	–21.5
**$TS3_B$**	–5.3	–3.1	7.9	5.5	3.8
**P_B**	–39.4	–35.1	–23.2	–33.7	–30.1
**E_A**	0	0	0	0	0
**I'_A**	0.2	–0.2	0.2	0.0	–1.0
**TS'_A**	5.3	3.8	6.2	10.5	4.8
**P'_A**	–4.1	–4.3	–0.7	6.0	0.2
**E_B**	0	0	0	0	0
**I'_B**	6.9	6.7	8.1	3.1	5.5
**TS'_B**	20.1	18.4	20.8	24.6	17.7
**P'_B**	15.8	14.8	19.6	26.1	18.9

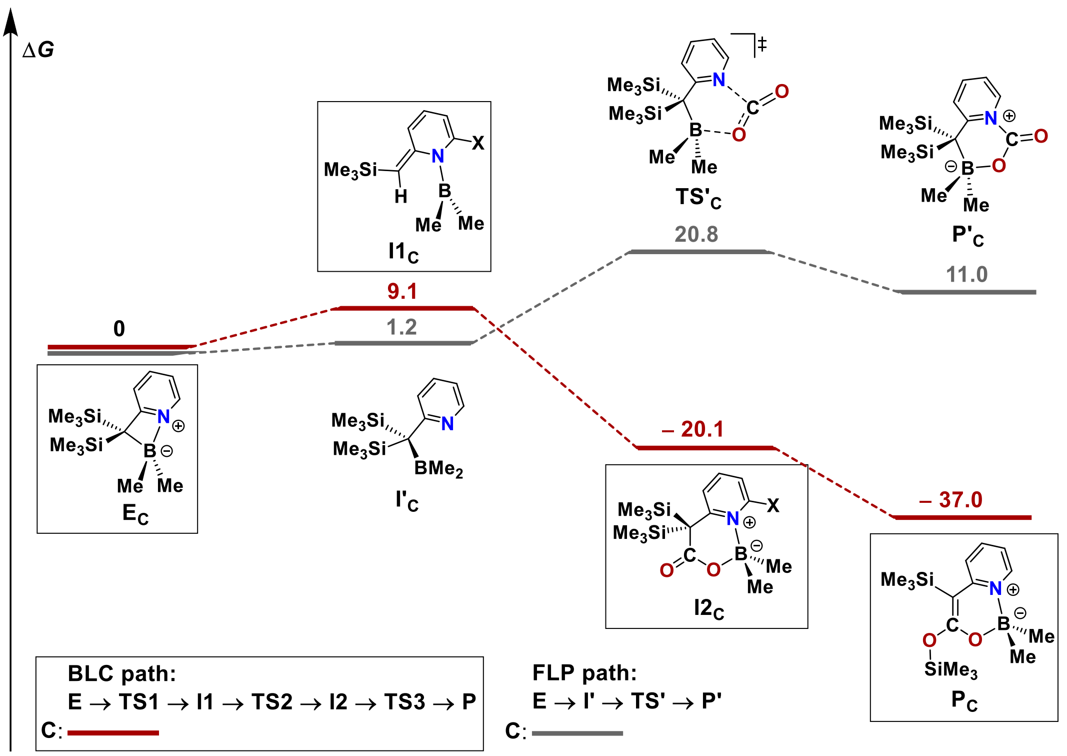

Figure S84. Computed mechanism of CO_2 activation for a bis-silyl-substituted 2-picolylborane model system at the M06-2X/6−311+G(d,p) level of theory (PCM, toluene).[87-92] Comparison of the BLC pathway (red) with the B/N-FLP pathway (gray). Gibbs energies (ΔG) in kcal mol^{-1}. As observed for the monosilyl-substituted system, the BLC pathway remains strongly exergonic, whereas the B/N-FLP-type pathway is endergonic, confirming that the BLC mechanism constitutes a general reaction principle for these systems, largely independent of the substitution pattern.

DFT Analysis of the *Z*/*E* Selectivity in the Reaction of 5 with Cinnamaldehyde

DFT calculations at the M06-2X/6–311+G(d,p) level of theory (values obtained with M06-2X/6–31+G(d) shown in brackets)[87-91] provide mechanistic insight into the formation of the *Z*/*E* isomer ratio of ***Z*/*E*-11** in the reaction of **5** with cinnamaldehyde (Figure S85, see also Scheme 4 in the main article). The process is initiated by a Zimmerman–Traxler-type chair-like transition state structure (**TS**$_{\mathbf{ZT}}$: $\Delta G^‡$ = 33.5 kcal mol^{-1}),[113] furnishing a β-siloxy ester intermediate (**16**). Although the reaction course does not suggest direct involvement of the boron atom in the initial transition state structure, the 1,2-addition step nevertheless resembles that of aldol reactions with boron enolates, for which a cyclic, highly ordered transition state is commonly proposed.[105,143-146] Direct elimination of Ph_3SiOH from intermediate **16** is unlikely; instead, tautomerization to the corresponding enol occurs. Rotation around the C(α)–C(β) bond gives access to two enol conformers (**17**$_{Z}$ and **17**$_{E}$), which differ in relative energy (ΔG = 4.1 vs. 6.7 kcal mol^{-1}). Subsequent elimination of Ph_3SiOH from these conformers proceeds via distinct transition states (**TS**$_{Z}$: $\Delta G^‡$ = 26.1 kcal mol^{-1} and **TS**$_{E}$: $\Delta G^‡$ = 27.0 kcal mol^{-1}), and the calculated energy difference between them closely reproduces the experimentally observed *Z*:*E* ratio (2:1).

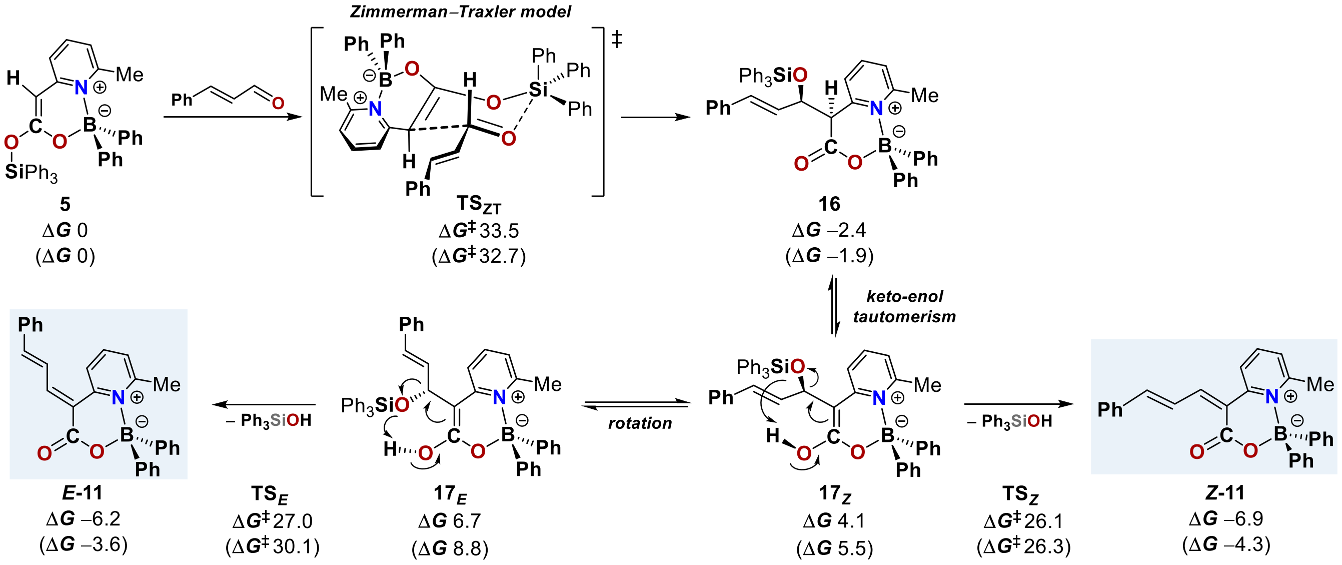

Figure S85. Mechanism for the formation of the *Z*/*E* isomer ratio of ***Z*/*E*-11** in the reaction of **5** with cinnamaldehyde, calculated at the M06-2X/6−311+G(d,p) level of theory (values obtained with M06-2X/6−31+G(d) shown in brackets). A Zimmerman–Traxler-type transition state (**TS$_{ZT}$**) furnishes a silyl ether intermediate (**16**) that tautomerizes to enol species **17$_{Z}$** and **17$_{E}$**. Subsequent Ph_3SiOH elimination proceeds via distinct transition states (**TS$_{Z}$** and **TS$_{E}$**), reproducing the experimentally observed 2:1 *Z*:*E* ratio. Gibbs energies (Δ*G*) in kcal mol^{-1}.

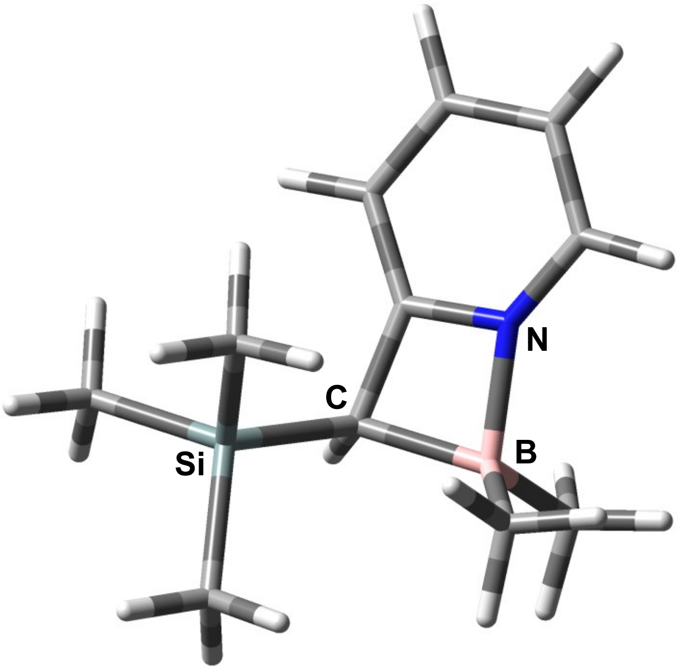

Figure S86. Optimized structure **E$_{A}$** [M06-2X/6–311+G(d,p)] (PCM, toluene).

Table S17. Cartesian coordinates of the optimized structure **E$_{A}$** [M06-2X/6–311+G(d,p)] (PCM, toluene).

Atomic symbol	x	y	z
Si	1.91195500	–0.46472800	0.04273300
N	–1.52881400	0.52047300	0.12779200
C	–2.72940800	0.32414600	0.67142200
C	–3.33446200	–0.91642800	0.55398900
H	–4.30898200	–1.08685400	0.99098200
C	–0.85039800	–0.44119200	–0.52697100
C	–2.66998400	–1.92856700	–0.14409400

H	–3.13924600	–2.90001500	–0.25036700
C	–1.41552700	–1.70421700	–0.69673800
H	–0.88433500	–2.47901300	–1.23522400
B	–0.35018800	1.64012600	–0.23060400
C	0.41797900	0.26256700	–0.85015000
H	0.63241700	0.27193000	–1.92450200
C	3.25694300	0.83650600	0.21289800
H	4.18310700	0.38889900	0.58386900
H	3.47016000	1.30119000	–0.75373300
H	2.95545100	1.62445000	0.90548400
C	2.59552500	–1.91076100	–0.95809900
H	2.90974900	–1.58547300	–1.95362600
H	3.46185300	–2.35739000	–0.46186200
H	1.84431000	–2.69590800	–1.08424400
C	1.41927200	–1.13551100	1.73361000
H	0.68156700	–1.93708000	1.63131400
H	2.29424400	–1.55361800	2.23964300
H	0.99223600	–0.36826800	2.38234000
C	0.20223300	2.39226600	1.07789300
H	0.57231400	1.73392800	1.86946500
H	1.02252700	3.06607100	0.80661500
H	–0.58137200	3.02102300	1.51787300
C	–0.91168100	2.62398600	–1.37478900
H	–1.36968400	2.08746000	–2.21347200
H	–1.65473900	3.32902900	–0.98364400
H	–0.09037800	3.22345600	–1.78339700
H	–3.18886700	1.16166500	1.18287500

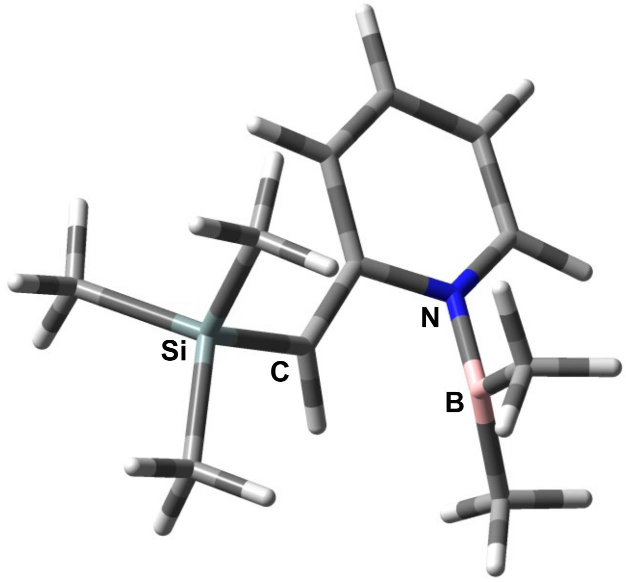

Figure S87. Optimized structure **TS1A** [M06-2X/6–311+G(d,p)] (PCM, toluene).

Table S18. Cartesian coordinates of the optimized structure **TS1A** [M06-2X/6–311+G(d,p)] (PCM, toluene).

Atomic symbol	x	y	z
Si	2.19674900	–0.18253200	–0.07677300
N	–1.80716200	0.29298300	–0.19928300
C	–3.06681500	–0.19375900	–0.16671000
C	–3.27604500	–1.53742600	–0.03000700
H	–4.28036900	–1.93314200	0.00927800
C	–0.81078700	1.98903400	1.54806200
H	0.03217000	2.68216000	1.60757500
C	–0.68807300	–0.50564300	–0.27666000
C	–1.54338300	2.82522000	–0.97366800
H	–2.54612900	3.21103900	–0.74357700
C	–2.14180700	–2.38170700	0.12229000
H	–2.29383600	–3.44081200	0.29709300
C	–0.87013500	–1.88856700	0.02634000
H	–0.00453100	–2.53602400	0.09632700
B	–1.21285200	1.67414200	0.05287300
C	0.45858500	0.23341200	–0.56342600
H	0.31275700	1.06305200	–1.25672900
H	–1.65978000	2.48938900	2.03500500
H	–0.57064100	1.09532300	2.12722800
H	–1.56237600	2.49249300	–2.01442200
H	–0.85755900	3.67096200	–0.88382300
C	3.24438600	1.35232500	–0.36282600
H	4.28941100	1.17183600	–0.09703600
H	3.21343300	1.65608800	–1.41288400
H	2.88100500	2.18844400	0.24075100
C	2.91442200	–1.60701000	–1.08447700
H	2.89246900	–1.37571000	–2.15253900
H	3.95296600	–1.80076100	–0.79986300
H	2.34968100	–2.53105500	–0.93331600
C	2.25829700	–0.65881000	1.74585700
H	1.58037900	–1.48828200	1.96707400
H	3.26623300	–0.96463000	2.04005500
H	1.96283200	0.18760800	2.37167200
H	–3.87007900	0.53347700	–0.20108200

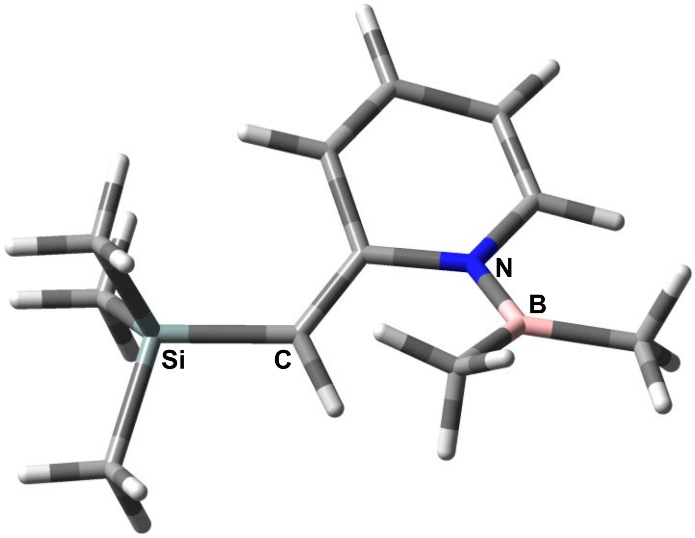

Figure S88. Optimized structure **I1$_A$** [M06-2X/6–311+G(d,p)] (PCM, toluene).

Table S19. Cartesian coordinates of the optimized structure **I1$_A$** [M06-2X/6–311+G(d,p)] (PCM, toluene).

Atomic symbol	x	y	z
Si	–2.41119400	–0.19368300	–0.11679800
N	1.80324100	–0.03101000	–0.18193100
C	2.72706500	0.99324200	–0.41501500
C	2.46469400	2.28465100	–0.14861100
H	3.22135800	3.03613400	–0.32480200
C	1.43835700	–2.48219700	0.77170900
H	1.07338100	–3.21865500	0.04552600
C	0.43085600	0.34914600	–0.01385100
C	3.77568600	–1.74220200	–0.36922600
H	4.48595300	–1.33430600	0.36046700
C	1.19019500	2.62650800	0.43920700
H	1.01511100	3.63464100	0.79582300
C	0.22762800	1.69046600	0.52845700
H	–0.74511400	1.92897000	0.94039100
B	2.29271500	–1.37923300	0.04391000
C	–0.58035900	–0.47113700	–0.37150400
H	–0.29735000	–1.40015300	–0.86116900
H	0.57310800	–2.11235400	1.32261300
H	2.09344900	–3.03818800	1.45016300
H	3.92088300	–2.82413600	–0.37453200
H	4.07187200	–1.35612300	–1.34816900
C	–2.83866400	–0.02350600	1.71072700
H	–3.92026700	0.07866000	1.83949900
H	–2.51631200	–0.91198100	2.26016100
H	–2.36557300	0.84432700	2.17619600
C	–3.02134600	1.32529100	–1.04700300

H	–2.83147000	1.21756800	–2.11795800
H	–4.09825700	1.45477000	–0.90522200
H	–2.52441100	2.23893000	–0.71263500
C	–3.28024600	–1.71517100	–0.79664600
H	–4.36470900	–1.62800600	–0.68934600
H	–3.05764100	–1.85177300	–1.85823900
H	–2.96170400	–2.61706100	–0.26678900
H	3.69495200	0.67298500	–0.77229300

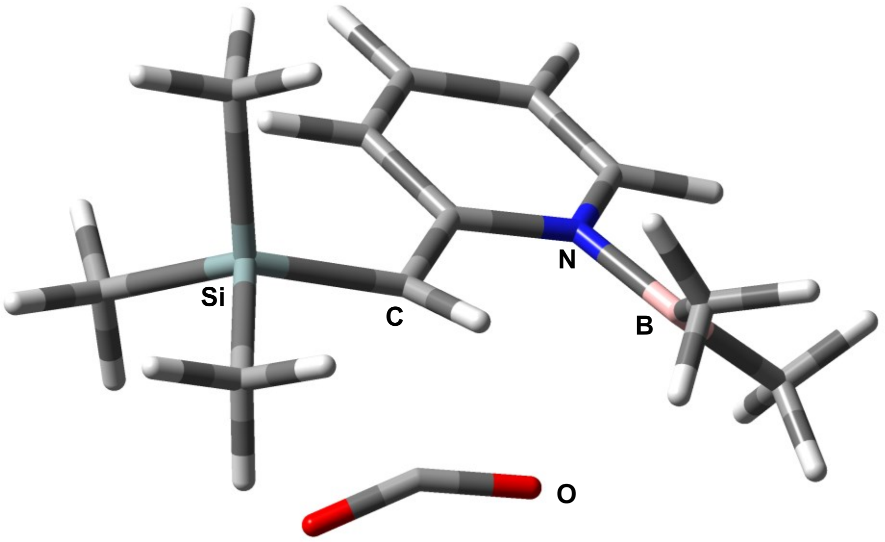

Figure S89. Optimized structure **TS2A** [M06-2X/6–311+G(d,p)] (PCM, toluene).

Table S20. Cartesian coordinates of the optimized structure **TS2A** [M06-2X/6–311+G(d,p)] (PCM, toluene).

Atomic symbol	x	y	z
N	1.80010800	0.34704800	0.30017400
C	2.77112100	1.27956200	0.01200900
C	2.49755600	2.55895200	–0.33832300
H	3.30366300	3.25242400	–0.52852100
C	1.67084400	–1.82486100	1.87909900
H	1.15985800	–2.72698400	1.52648500
C	0.44949900	0.67138800	0.17184200
C	3.64024500	–1.52362400	0.05253800
H	4.47714500	–1.25727600	0.70999900
C	1.13389900	2.94354000	–0.43707000
H	0.87310800	3.96072500	–0.70491300
C	0.15839900	2.03106800	–0.19676200
H	–0.88147500	2.31439900	–0.27770100
B	2.28404800	–1.03072300	0.66917200
C	–0.56566600	–0.28251900	0.29488800
C	–0.08634100	–1.49814400	–1.41929600
O	–1.05162000	–1.53474200	–2.09501700

O	1.02849800	–1.83749500	–1.17256400
H	0.98107300	–1.25946200	2.50685300
H	2.49937000	–2.17622900	2.50302200
H	3.84788400	–1.13077200	–0.94415600
H	3.63259500	–2.61514600	–0.00060200
H	3.78850200	0.92831000	0.10857500
H	–0.31460200	–1.18343500	0.84097900
Si	–2.40753500	0.07526500	0.33126500
C	–3.24694700	–1.59399000	0.52180700
H	–2.88427800	–2.11803300	1.41051400
H	–4.32894700	–1.47376600	0.62211200
H	–3.05370100	–2.22259700	–0.35070000
C	–2.83157700	1.15142500	1.82218400
H	–2.30294800	2.10819900	1.79442800
H	–3.90460400	1.36189500	1.85304600
H	–2.55902200	0.64780600	2.75325800
C	–3.06040000	0.91850900	–1.22074000
H	–2.84176800	1.98821300	–1.25558200
H	–2.65691600	0.45103500	–2.12032100
H	–4.14926600	0.81132300	–1.24397300

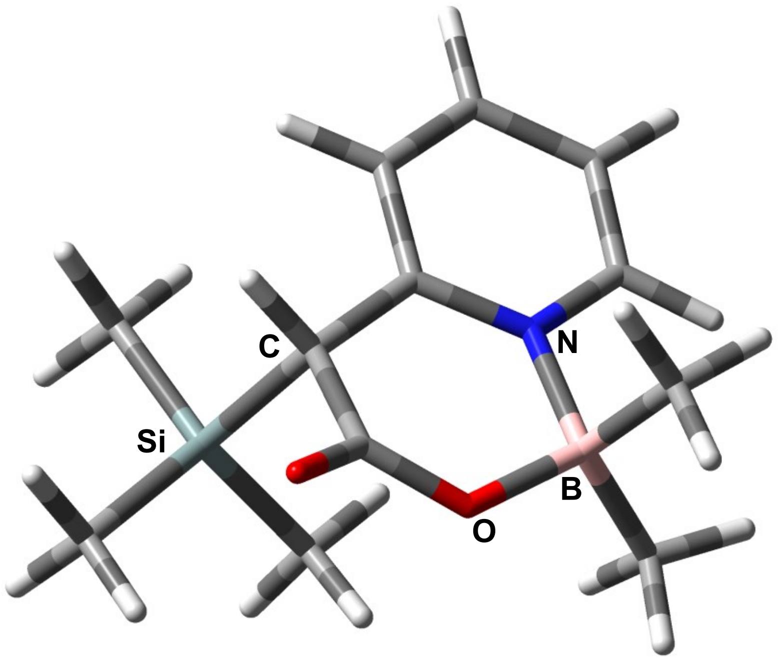

Figure S90. Optimized structure **I2A** [M06-2X/6–311+G(d,p)] (PCM, toluene).

Table S21. Cartesian coordinates of the optimized structure **I2A** [M06-2X/6–311+G(d,p)] (PCM, toluene).

Atomic symbol	x	y	z
N	–1.48425700	0.20237400	0.04099100
C	–2.54448700	0.94531400	0.41307300
C	–2.64679300	2.28657100	0.12449700

H	–3.51626900	2.84278300	0.44610300
C	–0.46447200	0.76785800	–0.62685100
C	–1.61799200	2.88437400	–0.60408000
H	–1.66994700	3.93241500	–0.87347100
C	–0.52981100	2.12280100	–0.97721100
H	0.28966500	2.55309000	–1.53883500
B	–1.61130400	–1.42431000	0.26733900
C	0.74736600	–0.03402600	–0.94060400
C	0.58663700	–1.53622500	–0.93440400
O	–0.28124600	–2.02021100	–0.07998800
O	1.31779000	–2.23438800	–1.60016600
C	–2.71864900	–1.92187700	–0.79569000
H	–2.82230700	–3.00940800	–0.73033800
H	–3.70973600	–1.49055600	–0.61701000
H	–2.43477400	–1.68485100	–1.82823300
C	–1.94107800	–1.72931300	1.81085100
H	–2.97428700	–1.49903200	2.08971600
H	–1.80494800	–2.80136200	1.98443500
H	–1.28014700	–1.20172200	2.50599000
H	–3.32038800	0.41195200	0.94592700
Si	2.13105500	0.36802400	0.37655000
H	1.16496900	0.27331900	–1.90141800
C	2.60395600	2.17433900	0.18898100
H	1.79708900	2.84672700	0.48831100
H	3.46408800	2.38485500	0.83100700
H	2.89323100	2.41180900	–0.83832200
C	3.59350300	–0.73643000	0.02177400
H	3.34067200	–1.78709900	0.17416000
H	3.93057500	–0.62372800	–1.01102300
H	4.42292600	–0.47706700	0.68532600
C	1.39822900	0.04160200	2.06596500
H	2.13744000	0.24691600	2.84500400
H	0.52499500	0.67264900	2.25593200
H	1.08788700	–1.00223300	2.15483800

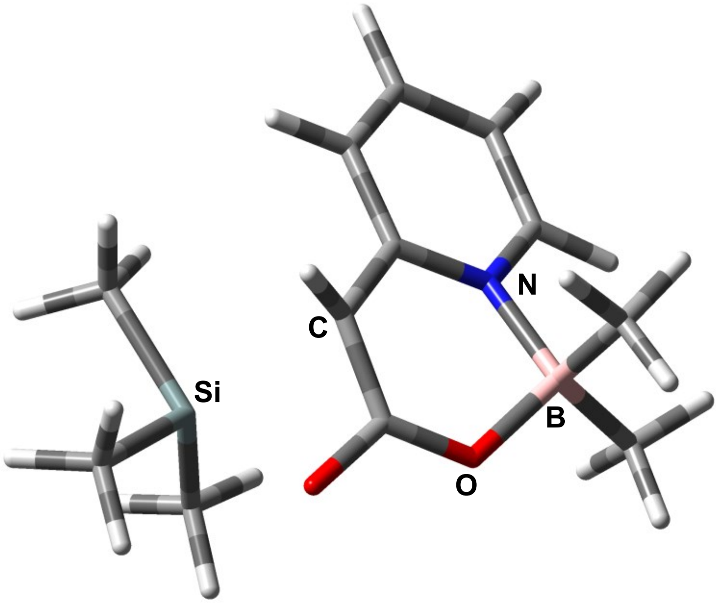

Figure S91. Optimized structure **TS3A** [M06-2X/6–311+G(d,p)] (PCM, toluene).

Table S22. Cartesian coordinates of the optimized structure **TS3A** [M06-2X/6–311+G(d,p)] (PCM, toluene).

Atomic symbol	x	y	z
N	–1.79388600	0.12256700	0.10546900
C	–2.89395200	0.75114000	0.56027300
C	–3.05953700	2.11316500	0.46181600
H	–3.96161100	2.57482200	0.83760600
C	–0.77453200	0.83361400	–0.43578100
C	–2.04806600	2.85871900	–0.15105800
H	–2.15402500	3.93051900	–0.27029200
C	–0.90817600	2.22260400	–0.59222500
H	–0.09691000	2.77108400	–1.05280700
B	–1.82588400	–1.50516500	–0.05401700
C	0.42837200	0.11758300	–0.79613000
C	0.57271800	–1.24144400	–0.37529700
O	–0.36805800	–1.95179900	0.10878300
O	1.78921900	–1.62480300	–0.32820000
C	–2.33346800	–1.80977900	–1.54850100
H	–2.33669400	–2.88840700	–1.73376700
H	–3.35655700	–1.44973700	–1.70054700
H	–1.70354500	–1.34573200	–2.31628300
C	–2.65801600	–2.19023600	1.13004500
H	–3.74185900	–2.08955300	1.01819500
H	–2.44505500	–3.26332700	1.11813400
H	–2.37984400	–1.81798200	2.12208100
H	–3.65270700	0.11100000	0.98939300
Si	2.50863100	0.14555800	0.14900200
H	0.90510600	0.37651200	–1.73718800

C	2.54504600	2.04448600	–0.01325700
H	1.71129100	2.51185900	0.51753800
H	3.47650600	2.41815700	0.42680800
H	2.52010100	2.37324400	–1.05686600
C	4.13963600	–0.33542400	–0.65077900
H	4.40284900	–1.36985100	–0.42494700
H	4.06537800	–0.24340700	–1.73919500
H	4.94270900	0.32784500	–0.31984500
C	2.33994800	–0.18821700	1.98314700
H	2.96894000	0.50636900	2.54608500
H	1.30695200	–0.03668000	2.31142000
H	2.62562100	–1.21114000	2.23206800

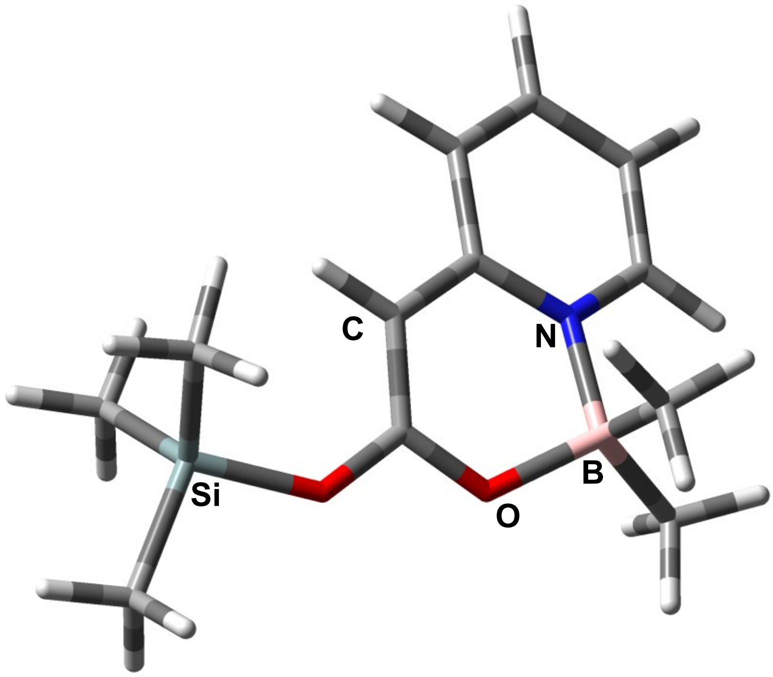

Figure S92. Optimized structure **P_A** [M06-2X/6–311+G(d,p)] (PCM, toluene).

Table S23. Cartesian coordinates of the optimized structure **P_A** [M06-2X/6–311+G(d,p)] (PCM, toluene).

Atomic symbol	x	y	z
N	–2.22071300	0.09814300	–0.09725700
C	–3.52992000	–0.11552900	–0.32597300
C	–4.09399100	–1.36765500	–0.29345800
H	–5.15164700	–1.49150900	–0.47698400
C	–1.72735300	1.97284100	1.63667700
C	–1.38244200	–0.94100900	0.15053600
C	–2.42734500	2.62855100	–0.90913800
C	–3.25854800	–2.45644100	–0.00471000
H	–3.66524100	–3.45987000	0.04156200
C	–1.91831800	–2.24690800	0.21015400

H	–1.24631000	–3.06994700	0.41683100
B	–1.66136500	1.61134100	0.06704000
C	0.01125900	–0.68782200	0.30478100
H	0.66021300	–1.50971400	0.56264500
C	0.52654600	0.53595400	–0.06257500
O	–0.21395600	1.53872700	–0.39907300
O	1.82384300	0.78943000	–0.14940600
H	–1.32449200	2.97598600	1.81103400
H	–2.76164700	1.97027000	1.99826600
H	–1.15883900	1.27493200	2.26162800
H	–2.50875000	2.25620700	–1.93638300
H	–3.43094800	2.89457300	–0.56258400
H	–1.85635300	3.56134900	–0.94780600
Si	3.19270800	–0.22294400	–0.06578500
C	3.32042200	–0.94618100	1.65160800
H	4.31105900	–1.38945900	1.78808200
H	3.19836500	–0.16360700	2.40462400
H	2.57960600	–1.72358500	1.84708200
C	4.59577700	0.94707700	–0.40857200
H	5.55325300	0.42032600	–0.39071200
H	4.47952500	1.41170900	–1.39019800
H	4.63001600	1.74102200	0.34071000
C	3.04895200	–1.53762200	–1.38291500
H	2.90907700	–1.08018500	–2.36542800
H	3.96767000	–2.12998100	–1.41613300
H	2.21562700	–2.21980900	–1.20466600
H	–4.11561500	0.77201500	–0.52563000

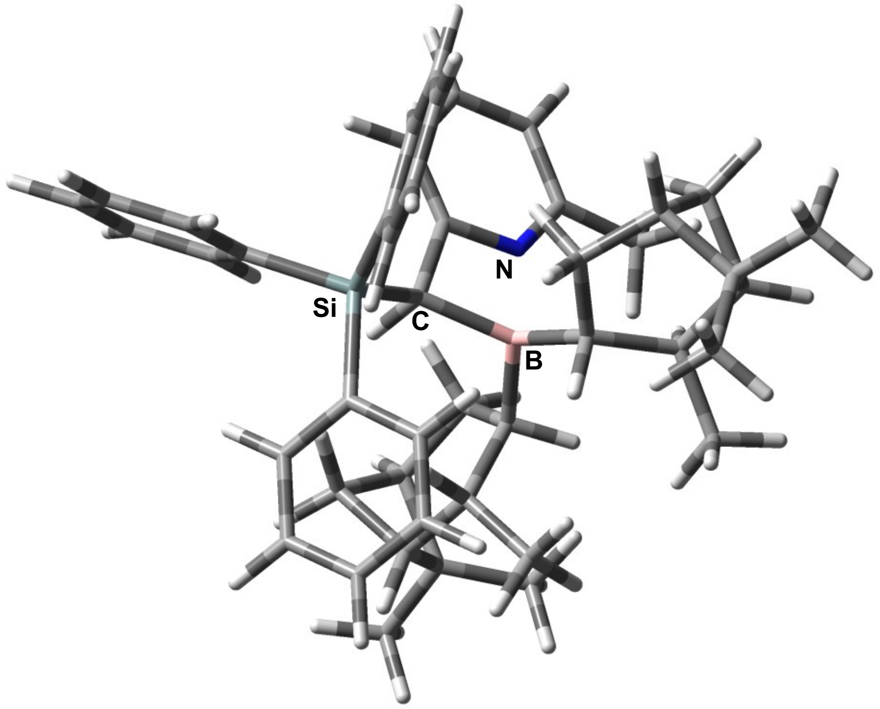

Figure S93. Optimized structure $\mathbf{E_B}$ [M06-2X/6–311+G(d,p)] (PCM, toluene).

Table S24. Cartesian coordinates of the optimized structure $\mathbf{E_B}$ [M06-2X/6–311+G(d,p)] (PCM, toluene).

Atomic symbol	x	y	z
Si	1.31979300	–1.56160500	–0.27117900
N	–0.44077800	1.00486300	2.15821200
C	0.68436700	–3.77451000	1.49974300
H	–0.16910800	–3.18895700	1.82961700
C	0.87973700	–5.03572900	2.05872700
H	0.18525800	–5.41182100	2.80153300
C	0.49103600	–1.78426000	–1.94607300
C	–5.02863400	–0.77276800	–0.04471500
C	3.05385700	–0.85571500	–0.50427700
C	–0.27944700	–2.91818800	–2.22514600
H	–0.27146100	–3.75773800	–1.53530000
C	0.45593600	–0.71996000	–2.85858100
H	1.03892700	0.17691500	–2.66415100
C	–2.70002400	–0.17246300	–1.05765800
H	–1.80711400	–0.74720900	–1.33024800
C	1.56402700	–3.25850400	0.53932100
C	–3.81853600	–1.21899600	–0.92911100
H	–4.05638300	–1.64173200	–1.91137500
C	–3.43970700	–2.20139800	0.20300300
H	–3.94666200	–3.16259200	0.14071300
H	–2.37196200	–2.37928700	0.35168600
C	–0.63932500	1.70735800	3.28195500

C	1.96447800	–5.81086500	1.66310700
H	2.11910500	–6.79224000	2.09693900
C	0.05029200	1.32125700	4.43004600
H	–0.09550600	1.88019600	5.34563200
C	0.30553000	4.52866200	0.04070900
H	–0.17464200	5.50782200	0.14514300
C	3.78289100	–0.36710700	0.58886800
H	3.32950000	–0.34470700	1.57600300
C	3.68237100	–0.87179100	–1.75394800
H	3.15154100	–1.25396800	–2.62019800
C	–0.10478500	2.01369700	–0.35083700
H	–0.31257700	1.73392100	–1.39077000
C	0.32424200	–0.10771900	2.12175400
C	–2.41533800	0.57693700	0.29486000
H	–2.87690100	1.56603300	0.20262300
C	–0.74872200	3.42946800	–0.14038400
H	–1.30680100	3.41228200	0.80132800
C	2.85260600	–5.31885700	0.70974300
H	3.70267600	–5.91670400	0.40068300
C	2.65297900	–4.05688900	0.15950200
H	3.36234500	–3.68177700	–0.57307300
C	1.29685800	4.09162200	1.14829500
H	1.82965500	4.91931200	1.61363800
H	0.90653900	3.43222800	1.92783500
C	0.89931400	0.21860900	4.39944900
H	1.43159000	–0.07192800	5.29804600
C	–0.33132100	–0.77907600	–4.00261000
H	–0.35297600	0.05895300	–4.69045000
C	–1.07669300	–2.98060300	–3.36720300
H	–1.67591500	–3.86290000	–3.56219400
C	–6.21821900	–1.72111400	–0.22633400
H	–6.71031100	–1.52516800	–1.18417900
H	–6.95591300	–1.55976600	0.56621800
H	–5.93516200	–2.77419000	–0.20905200
C	4.98449300	–0.39931900	–1.91278300
H	5.44988200	–0.41809600	–2.89172300
C	–4.12963000	–1.19517200	1.15235800
H	–4.63381600	–1.58370100	2.04330900
C	–1.60300200	2.85602600	3.28159900
H	–2.40530500	2.67909900	2.56447700

H	–1.09789100	3.78545500	3.00641900
H	–2.03050900	2.98245800	4.27648900
C	–1.10695300	–1.90903900	–4.25340700
H	–1.73241700	–1.95183000	–5.13802400
C	5.07989100	0.10892900	0.43736400
H	5.62096200	0.49013400	1.29601000
C	1.45075700	2.01931900	–0.20136000
H	1.75669400	1.40918800	0.65411500
H	1.91284700	1.54673800	–1.07439100
C	2.07926600	3.39651300	0.01402300
H	3.16923300	3.32601900	0.09182800
C	1.49896700	4.44859700	–0.96833200
C	–2.98323100	0.76150300	–2.23673700
H	–3.19909000	0.17004800	–3.13170200
H	–2.12556700	1.39597900	–2.46565900
H	–3.84310100	1.40866700	–2.04325600
C	–3.10893300	–0.11156300	1.51813500
H	–2.37036100	–0.59104200	2.17363700
H	–3.59634900	0.65485700	2.13251300
C	1.29795900	4.03147700	–2.42259200
H	0.77375800	3.08294100	–2.53233600
H	2.27636900	3.92786800	–2.90427200
H	0.74025300	4.79555900	–2.97296400
C	–1.76444000	3.83063100	–1.21538900
H	–2.09978700	4.85927500	–1.04933900
H	–2.64531700	3.18973800	–1.19388900
H	–1.34185800	3.77524000	–2.22078700
C	1.03872100	–0.52876600	3.23582500
H	1.65252500	–1.42051300	3.19216700
C	2.30509800	5.74966900	–0.96220000
H	1.76673500	6.52893400	–1.51081900
H	3.27065100	5.59925800	–1.45506500
H	2.49858300	6.12563100	0.04347300
C	–5.57666200	0.65121000	–0.09107800
H	–4.82261300	1.41776900	0.07167600
H	–6.34603500	0.76945900	0.67967400
H	–6.05164700	0.84473400	–1.05813100
C	5.68299900	0.09658100	–0.81847700
H	6.69336400	0.46980800	–0.94014400
B	–0.81692800	0.82868000	0.51207200

C	0.06903100	–0.60490800	0.74597000
H	–0.70956300	–1.37633100	0.84796900

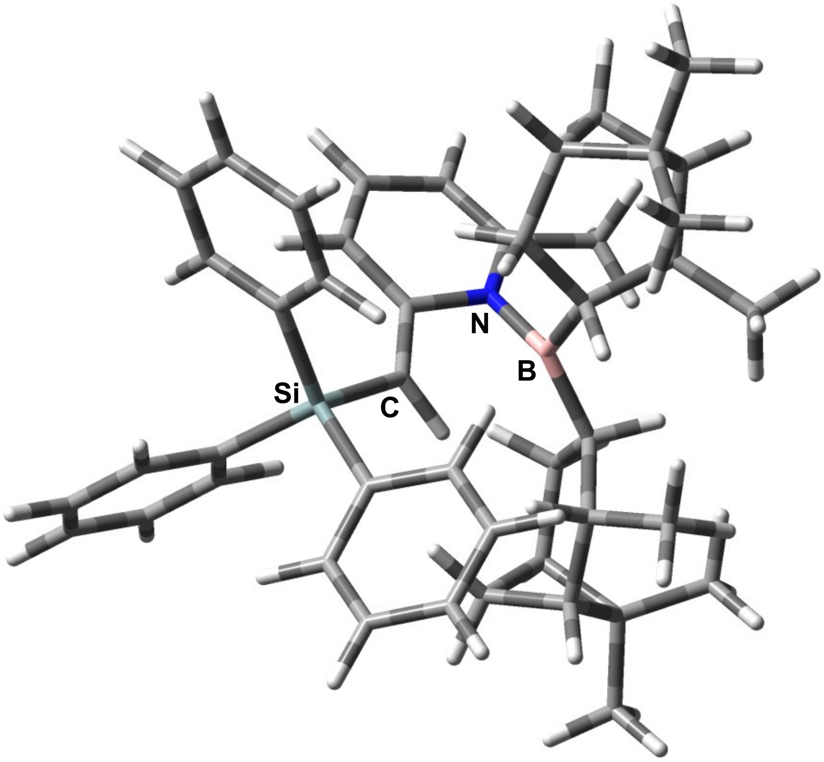

Figure S94. Optimized structure **TS1$_B$** [M06-2X/6–311+G(d,p)] (PCM, toluene).

Table S25. Cartesian coordinates of the optimized structure **TS1$_B$** [M06-2X/6–311+G(d,p)] (PCM, toluene).

Atomic symbol	x	y	z
Si	2.25566700	0.38530400	–0.23121700
N	–1.14316200	0.10983900	2.00711800
C	3.70061800	–1.30428700	1.58401700
H	2.71639500	–1.61140000	1.92638500
C	4.83763100	–1.86424500	2.16368300
H	4.73641900	–2.60577600	2.94821000
C	2.07232800	–0.30949100	–1.97911900
C	–1.67329100	–4.85457300	0.06495300
C	2.52247900	2.25799700	–0.31234200
C	3.08499400	–1.04234900	–2.60870500
H	4.02019800	–1.22556100	–2.08874600
C	0.87339300	–0.11504700	–2.68079800
H	0.05886900	0.42541400	–2.20548000
C	–1.34894000	–2.48811400	–0.98506900
H	–0.48591300	–1.88695500	–1.29574300
C	3.80239500	–0.34433600	0.57149800
C	–0.80829400	–3.91947500	–0.84332200
H	–0.52864200	–4.31230100	–1.82602200

C	0.26451700	–3.93863900	0.27069300
H	0.95239600	–4.77837800	0.19776900
H	0.84687900	–3.02570500	0.41473200
C	–1.81581600	0.30549100	3.17038700
C	6.10097700	–1.46934300	1.73665700
H	6.98757900	–1.90254900	2.18548500
C	–1.22113900	1.04425000	4.16328400
H	–1.75047800	1.21426300	5.09096200
C	–4.14390400	2.33528600	0.04210800
H	–5.22505600	2.38112800	0.21082100
C	3.27531500	2.92245000	0.66521600
H	3.75970500	2.35167000	1.45303600
C	1.95639600	3.03142600	–1.33377900
H	1.39483000	2.54761800	–2.12689700
C	–2.13637900	0.76182800	–0.36807600
H	–2.05872400	0.36051900	–1.38185600
C	0.18315600	0.45530900	1.83459000
C	–1.88590200	–1.92714300	0.38720900
H	–2.98006300	–1.94503600	0.30031900
C	–3.68979400	0.87490600	–0.07131600
H	–3.89021200	0.44788400	0.91860300
C	6.22473700	–0.51062500	0.73283000
H	7.20773200	–0.19593900	0.40108900
C	5.08555900	0.04614200	0.16108700
H	5.19654300	0.80296700	–0.61156400
C	–3.21930400	3.05903300	1.05094400
H	–3.66332800	3.94993800	1.49096300
H	–2.78155400	2.44763800	1.84575900
C	0.05578900	1.61078500	3.93585900
H	0.49611100	2.24524800	4.69684500
C	0.69206300	–0.62382200	–3.96278100
H	–0.24639900	–0.46310600	–4.48213700
C	2.90968700	–1.55802500	–3.89136500
H	3.70539300	–2.12763600	–4.35811900
C	–1.26353900	–6.31931000	–0.11926300
H	–1.65769900	–6.70224300	–1.06566600
H	–1.67794600	–6.93191800	0.68740300
H	–0.18311900	–6.46448200	–0.12735600
C	2.11181300	4.41421600	–1.36554400
H	1.66383000	4.99028900	–2.16745600

C	–0.91047500	–4.18397900	1.24233300
H	–0.73145500	–4.79121700	2.13501800
C	–3.20951400	–0.23947200	3.31123200
H	–3.94600200	0.55483400	3.16459100
H	–3.34474700	–0.64032500	4.31652600
H	–3.40969900	–1.03268700	2.59151300
C	1.71415800	–1.34843100	–4.57086400
H	1.57724100	–1.75182600	–5.56777500
C	3.43629300	4.30522700	0.64054100
H	4.02290700	4.79602700	1.40893100
C	–1.39811400	2.13893500	–0.36348700
H	–0.64177600	2.18303400	0.42165900
H	–0.85234200	2.24971200	–1.30567000
C	–2.31052200	3.34788700	–0.16439200
H	–1.73437500	4.27787300	–0.17928700
C	–3.57111400	3.27456100	–1.06750000
C	–2.35897100	–2.40853400	–2.13209200
H	–2.64000100	–1.38017500	–2.36214500
H	–3.27375300	–2.96300100	–1.90796300
H	–1.91838000	–2.83776800	–3.03649800
C	–1.51886400	–2.82955100	1.61351100
H	–0.79974800	–2.31836100	2.26367200
H	–2.41503500	–2.99575500	2.22071500
C	–3.39662200	2.79892400	–2.50649000
H	–2.82804600	1.87394100	–2.59226400
H	–2.86656000	3.56811000	–3.07803400
H	–4.36863000	2.64547100	–2.98540100
C	–4.56861800	0.08953500	–1.04901700
H	–5.62375900	0.27335800	–0.82672600
H	–4.40019200	–0.98563200	–0.97368000
H	–4.38949600	0.37962900	–2.08592300
C	0.75433400	1.34078400	2.79200500
H	1.75216300	1.72606000	2.62781700
C	–4.33165200	4.60229600	–1.10156800
H	–5.31116000	4.46421900	–1.57004300
H	–3.77826100	5.33859700	–1.69248000
H	–4.49356000	5.02518500	–0.10915100
C	–3.19914600	–4.80854000	0.05503700
H	–3.61677000	–3.81835400	0.22726500
H	–3.58472400	–5.47020400	0.83789000

H	–3.58699900	–5.17509900	–0.90047100
C	2.84938200	5.05478400	–0.37400500
H	2.97413100	6.13134700	–0.39741300
B	–1.56469600	–0.37024300	0.60736800
C	0.72560100	–0.09016000	0.67221000
H	0.33933400	–1.07187500	0.40839900

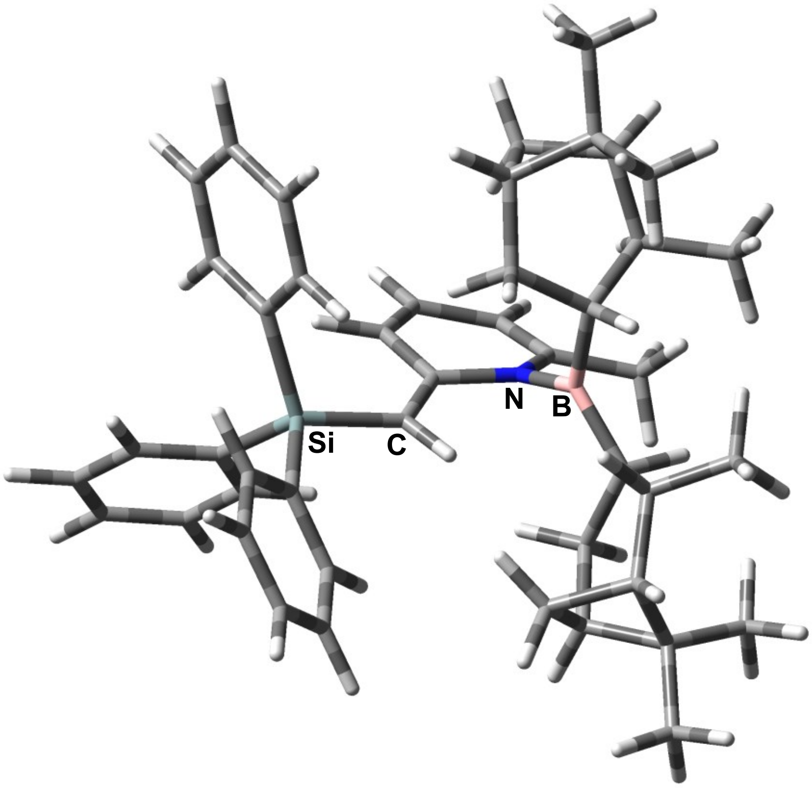

Figure S95. Optimized structure $\mathbf{I1_B}$ [M06-2X/6–311+G(d,p)] (PCM, toluene).

Table S26. Cartesian coordinates of the optimized structure $\mathbf{I1_B}$ [M06-2X/6–311+G(d,p)] (PCM, toluene).

Atomic symbol	x	y	z
Si	2.49476600	–0.21353400	–0.18764600
N	–1.10263000	0.22880000	1.83998500
C	3.46394400	–1.32402600	2.29209900
H	2.41666700	–1.47211300	2.53942200
C	4.44696300	–1.74061300	3.18733700
H	4.16361600	–2.20779600	4.12377200
C	2.64808100	–1.38152100	–1.66012700
C	–3.22771900	–4.24674400	–0.22766500
C	2.86489200	1.55427300	–0.74993600
C	2.14597700	–2.68459200	–1.53848400
H	1.65332300	–2.98627300	–0.61739600
C	3.29955100	–1.03830700	–2.85035300
H	3.71063000	–0.04078800	–2.97272700
C	–3.09445200	–1.76199900	–1.01092200

H	–2.44049900	–1.18773800	–1.68203700
C	3.80326000	–0.71675800	1.07835900
C	–2.81640500	–3.23291600	–1.34500200
H	–3.15443700	–3.46097000	–2.36091900
C	–1.34273800	–3.52613100	–0.98393400
H	–0.91716700	–4.37803800	–1.51101100
H	–0.65302700	–2.68471100	–1.06916700
C	–1.59545500	0.49684700	3.09633400
C	5.79101600	–1.55900600	2.87867100
H	6.55745900	–1.88334100	3.57340300
C	–0.80144300	1.08080900	4.03782000
H	–1.20314700	1.29636400	5.01776800
C	–2.88087200	3.72120700	0.23640000
H	–3.57470500	4.39354000	0.75266900
C	3.66612200	2.42854500	–0.00588000
H	4.16190400	2.07224800	0.89248500
C	2.24853300	2.05865500	–1.90509300
H	1.61389900	1.40975800	–2.50353300
C	–2.34276600	1.24943200	–0.26399300
H	–3.00761900	0.89234700	–1.04963900
C	0.27470200	0.27576700	1.56722400
C	–2.69840300	–1.42230600	0.47132800
H	–3.65772600	–1.24354100	0.98793400
C	–3.20203300	2.26232700	0.58619200
H	–2.91624900	2.18305900	1.64138000
C	6.14996100	–0.96342600	1.67158800
H	7.19638100	–0.82496900	1.42446400
C	5.16342400	–0.54966600	0.78292700
H	5.45877700	–0.08996800	–0.15708100
C	–1.36112200	3.91915500	0.46165300
H	–1.06851100	4.95233300	0.63532700
H	–0.89797900	3.27990800	1.22091500
C	0.53729300	1.43184300	3.69073800
H	1.14313600	1.99094800	4.39471400
C	3.43161400	–1.95759200	–3.88880300
H	3.93925400	–1.67135500	–4.80298800
C	2.27103900	–3.60620300	–2.57267800
H	1.87208300	–4.60787000	–2.45719100
C	–3.22249000	–5.67840700	–0.76941800
H	–4.10160100	–5.84523000	–1.39937700

H	–3.26205200	–6.39459900	0.05691600
H	–2.33805200	–5.90496600	–1.36595400
C	2.43061800	3.37774500	–2.30542700
H	1.94862500	3.74241800	–3.20580700
C	–1.86926100	–3.87080400	0.42882500
H	–1.35754100	–4.65161500	0.99899000
C	–3.01781900	0.10894700	3.38966300
H	–3.30331100	0.47773500	4.37351800
H	–3.12101400	–0.97937400	3.38321100
H	–3.72243100	0.51281100	2.65907900
C	2.91378100	–3.24178100	–3.75299900
H	3.01465800	–3.95770600	–4.56069400
C	3.84606600	3.75427400	–0.39581000
H	4.46922800	4.41238700	0.19927400
C	–1.13269600	1.92948900	–0.99178600
H	–0.18819800	1.66955800	–0.51824300
H	–1.08627600	1.53258000	–2.01247300
C	–1.20134200	3.45678300	–1.00540100
H	–0.38017400	3.87451500	–1.59503900
C	–2.63182900	3.99892600	–1.28024700
C	–4.53570200	–1.37245000	–1.34883300
H	–4.74725200	–1.58452900	–2.40045000
H	–4.71863400	–0.30958500	–1.18122600
H	–5.25348200	–1.92959600	–0.74346400
C	–1.98266800	–2.58183700	1.24374800
H	–0.97368100	–2.27745700	1.53802200
H	–2.52294000	–2.79486600	2.17172300
C	–3.47936700	3.34723800	–2.37009600
H	–3.52038200	2.26160100	–2.31399400
H	–3.07050700	3.60965600	–3.35135400
H	–4.50568300	3.72638100	–2.33476000
C	–4.69637700	1.95380900	0.48659700
H	–5.26925000	2.61343800	1.14402300
H	–4.91939000	0.92080700	0.77379700
H	–5.06179300	2.09499900	–0.53342500
C	1.05992200	1.05496800	2.49891900
H	2.08446400	1.29205600	2.23898700
C	–2.62161900	5.50625000	–1.55428000
H	–3.64349100	5.89779800	–1.53993400
H	–2.20112900	5.70503700	–2.54493100

H	–2.03842100	6.06938700	–0.82517700
C	–4.50128900	–4.06791700	0.59308300
H	–4.59321700	–3.08545000	1.05367300
H	–4.52208900	–4.81280700	1.39560900
H	–5.38675300	–4.23275000	–0.02844900
C	3.22989100	4.23076100	–1.54754600
H	3.37093600	5.26059000	–1.85505500
B	–2.00608300	–0.01180100	0.64157000
C	0.77947300	–0.37673500	0.47170700
H	0.08535500	–0.97525200	–0.11729100

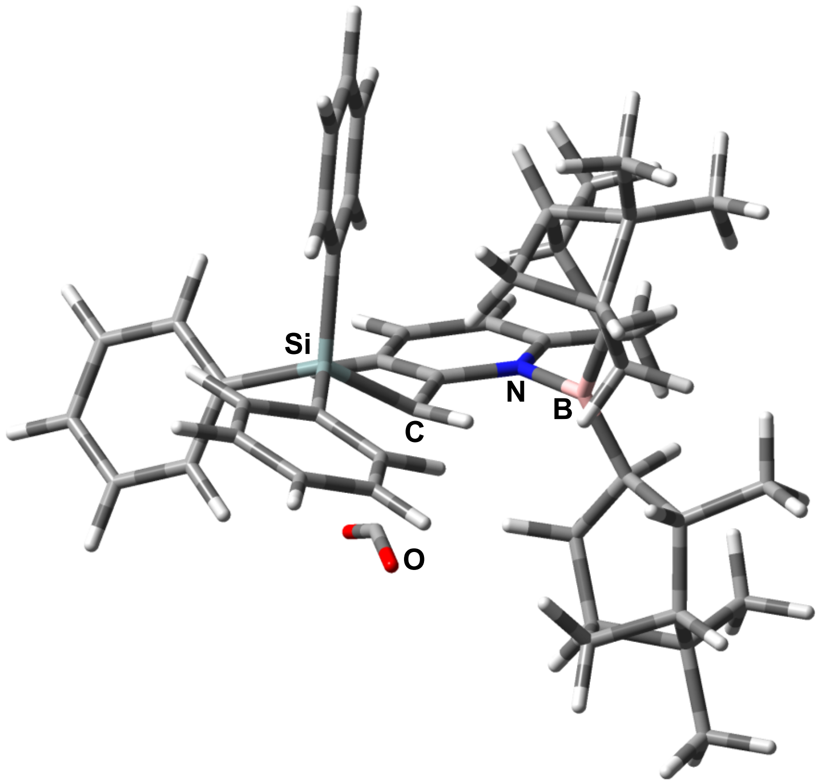

Figure S96. Optimized structure **TS2$_B$** [M06-2X/6–311+G(d,p)] (PCM, toluene).

Table S27. Cartesian coordinates of the optimized structure **TS2$_B$** [M06-2X/6–311+G(d,p)] (PCM, toluene).

Atomic symbol	x	y	z
N	1.23749400	–0.14363900	–1.83030700
C	5.46902300	–1.91101000	0.72972800
C	4.00018700	0.22188200	1.09259500
H	3.25148700	0.51606800	1.83032700
C	4.72168500	–0.97063900	1.73126600
H	5.28828800	–0.64523500	2.60996300
C	3.67124000	–2.09471900	1.90796500
H	3.92223400	–2.82010400	2.67858600
H	2.63406400	–1.77788400	2.04202200
C	1.76240500	–0.16259200	–3.09627400

C	1.13593100	–0.86150500	–4.09294800
H	1.55099700	–0.84662900	–5.09118200
C	–0.54372900	3.53261400	–0.13201100
H	–1.38262600	3.95431900	–0.69582000
C	1.52550900	2.02351500	–0.28468400
H	2.36584500	2.58850400	–0.72383300
C	0.08821700	–0.82744300	–1.49845100
C	3.28236000	–0.19494400	–0.23068500
H	3.93100300	0.23399400	–1.01394400
C	0.24295600	2.54853100	–1.00622100
H	–0.43551600	1.70605900	–1.15690600
C	–0.85772800	2.82911700	1.20933200
H	–1.71322400	3.24234900	1.73933500
H	–0.96444900	1.74484900	1.16939500
C	–0.02610100	–1.59838100	–3.79266100
H	–0.51056100	–2.18212700	–4.56663200
C	6.39887200	–2.86554800	1.48369600
H	7.29454400	–2.33172200	1.81616900
H	6.71855600	–3.67946400	0.82597100
H	5.93072800	–3.31065400	2.36213700
C	4.07060600	–2.54407700	0.48360900
H	4.03662700	–3.61025100	0.24354600
C	3.03182100	0.59656000	–3.37440100
H	3.09596300	1.52672300	–2.80606600
H	3.07368000	0.84641000	–4.43391100
H	3.90743400	–0.01443500	–3.13790100
C	1.56533200	2.34011400	1.25058500
H	1.38098700	1.43261600	1.83584800
H	2.56334900	2.68993700	1.52686900
C	0.50788100	3.35778900	1.68751300
H	0.63377500	3.61335600	2.74378600
C	0.34476300	4.53285500	0.68122500
C	4.92744100	1.42013600	0.87513600
H	5.30656800	1.79319100	1.83022000
H	4.40757800	2.24458900	0.37752800
H	5.78589500	1.14956900	0.25625400
C	3.25315400	–1.73369300	–0.51964700
H	2.23107900	–2.11654000	–0.49951800
H	3.62086300	–1.91913600	–1.53594800
C	1.59183900	5.18201600	0.08513500

H	2.07456700	5.80505900	0.84508700
H	1.31920700	5.83734500	–0.74780600
H	2.33632400	4.47408400	–0.27421600
C	0.54411800	3.12776300	–2.38935100
H	0.95250400	2.37110100	–3.06450400
H	1.25635500	3.95585100	–2.33633400
H	–0.37772300	3.50191900	–2.84363800
C	–0.53667000	–1.58446600	–2.52737600
H	–1.41024500	–2.16692700	–2.26655600
C	–0.51559500	5.65825500	1.26465800
H	–1.45460700	5.30111600	1.68848200
H	–0.75850700	6.38385200	0.48226200
H	0.03104600	6.18567600	2.05247800
C	6.24673000	–1.36986600	–0.46793100
H	5.67047900	–0.70435900	–1.10947600
H	6.58510800	–2.20828000	–1.08573600
H	7.13775600	–0.82763300	–0.13699200
B	1.97684500	0.56285000	–0.67562200
C	–0.38667700	–0.84511000	–0.16016300
C	0.06000400	–2.78607800	0.54559400
O	0.03675500	–3.51544300	–0.38952900
H	0.20860400	–0.25234000	0.54163000
Si	–2.22218700	–0.70938000	0.21142300
C	–2.99488200	0.83342500	–0.58854400
C	–2.81135800	1.12316100	–1.94994600
C	–3.76339100	1.73139200	0.16230700
C	–3.36562600	2.25804600	–2.53388900
H	–2.21446500	0.46181900	–2.57225800
C	–4.32163900	2.87034300	–0.41472600
H	–3.91777300	1.55180600	1.22160100
C	–4.12412300	3.13712200	–1.76506500
H	–3.20377000	2.45669300	–3.58760100
H	–4.90752400	3.55032700	0.19373200
H	–4.55557300	4.02367400	–2.21564100
C	–2.48051000	–0.59978000	2.07541400
C	–3.77474600	–0.80127500	2.57628800
C	–1.46827700	–0.28873600	2.98958100
C	–4.05235600	–0.67934100	3.93427800
H	–4.58086000	–1.06243300	1.89547100
C	–1.73761900	–0.16671000	4.34977700

H	–0.44734700	–0.16072000	2.64672300
C	–3.03156900	–0.35777400	4.82394800
H	–5.06091000	–0.83922300	4.29806200
H	–0.93544000	0.06833700	5.03990700
H	–3.24177800	–0.26518100	5.88337400
C	–3.22022400	–2.20138700	–0.38037500
C	–4.10569700	–2.13524400	–1.46262900
C	–3.10261600	–3.42201200	0.29935700
C	–4.83165800	–3.25338800	–1.86856700
H	–4.23896900	–1.19827200	–1.99443500
C	–3.82535700	–4.54035800	–0.09953300
H	–2.44336600	–3.50012600	1.16008200
C	–4.68946500	–4.45819600	–1.18878300
H	–5.51217600	–3.18082400	–2.70940200
H	–3.71636500	–5.47502900	0.43826500
H	–5.25456100	–5.32934700	–1.50049600
O	0.21974800	–2.62030300	1.71168100

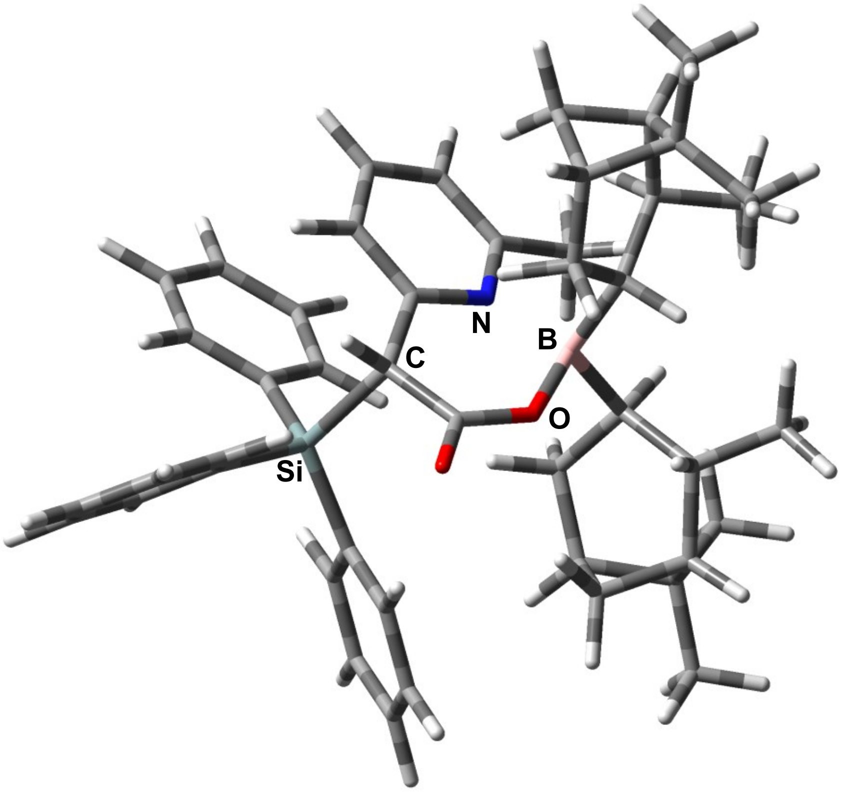

Figure S97. Optimized structure **I2$_B$** [M06-2X/6–311+G(d,p)] (PCM, toluene).

Table S28. Cartesian coordinates of the optimized structure **I2$_B$** [M06-2X/6–311+G(d,p)] (PCM, toluene).

Atomic symbol	x	y	z
N	0.86765800	–0.72137900	1.02569200
C	1.09513200	4.71816300	0.33992700
C	2.41774800	2.72847000	–0.71166300

H	2.35896700	2.17609100	–1.65717800
C	1.60759500	4.00942900	–0.95595600
H	2.11287800	4.64553000	–1.69100200
C	0.14599100	3.59411900	–1.23549000
H	–0.43034700	4.34208100	–1.77698900
H	0.00261400	2.61906800	–1.70144900
C	1.32011200	–0.90953300	2.30020600
C	0.99831300	–2.05920300	3.00575100
H	1.37721200	–2.17282900	4.01242600
C	4.68026700	–2.41260800	0.09416200
H	5.46685000	–2.69457900	0.80264900
C	3.01718300	–0.54909500	–0.54617500
H	3.55129600	0.23314400	–1.09523400
C	–0.02921400	–1.60290700	0.52220500
C	1.79702200	1.83416500	0.42560000
H	2.49179400	1.92703800	1.25935000
C	4.03070400	–1.08351000	0.51512800
H	3.49603800	–1.35197600	1.43237300
C	3.53551100	–3.42163200	–0.16308200
H	3.83335400	–4.46544000	–0.08298700
H	2.61814000	–3.26278600	0.41429200
C	0.21221400	–3.04429100	2.42493100
H	–0.01102300	–3.96224500	2.95503000
C	0.58823300	6.12841900	0.02760900
H	1.43301200	6.80622700	–0.13173600
H	0.00325200	6.51492300	0.86841400
H	–0.04108200	6.16524300	–0.86246400
C	–0.05157400	3.66699300	0.29572400
H	–1.03207200	3.96861100	0.67725000
C	2.12164100	0.14960500	3.00173400
H	2.35842000	–0.19799100	4.00620100
H	1.54175300	1.07016500	3.08059400
H	3.05153600	0.38099700	2.49070600
C	2.64157300	–1.62943800	–1.61302700
H	1.62424100	–2.00842100	–1.45296900
H	2.62947600	–1.16230000	–2.60224900
C	3.53279700	–2.87125400	–1.60806000
H	3.27403300	–3.55083300	–2.42591200
C	5.03652200	–2.52308500	–1.42321700
C	3.89679300	3.04935300	–0.47815900

H	4.26339900	3.72735800	–1.25465700
H	4.51169100	2.14939500	–0.51304800
H	4.06243900	3.52903900	0.49019600
C	0.39745800	2.33846900	0.90896800
H	–0.37521700	1.60204500	0.65873700
H	0.38694400	2.43327600	2.00225800
C	5.62779100	–1.35128900	–2.20188800
H	5.04579500	–0.43509400	–2.13311300
H	5.69644400	–1.62011100	–3.26123500
H	6.64348000	–1.13541500	–1.85525500
C	5.09431800	–0.05316600	0.89877200
H	5.73404200	–0.44185500	1.69678700
H	4.65327100	0.88441200	1.24592300
H	5.73335800	0.18811800	0.04539200
C	–0.32123800	–2.79908400	1.17957700
H	–1.00856400	–3.49424500	0.71414500
C	5.93330600	–3.73855700	–1.67910400
H	6.95482200	–3.52793600	–1.34730800
H	5.96940700	–3.96122700	–2.74997100
H	5.59428800	–4.63714600	–1.16256800
C	1.95774600	4.80505800	1.59576100
H	2.32317700	3.83814300	1.93742200
H	1.37502200	5.24965800	2.41005600
H	2.82367300	5.45189400	1.42197800
B	1.67011500	0.28282000	–0.06507200
C	–0.82778800	–1.26621000	–0.69339200
C	–0.19392500	–0.34294000	–1.69746300
O	–0.64251400	–0.26636400	–2.82472500
O	0.79237700	0.38895100	–1.27980400
H	–1.06591600	–2.19632100	–1.21500500
Si	–2.61718600	–0.61460300	–0.20914600
C	–2.83891000	–1.11379400	1.59347700
C	–2.14463400	–0.41438400	2.59391300
C	–3.53500300	–2.27009400	1.96305200
C	–2.14219500	–0.85692500	3.91120700
H	–1.58337200	0.48204000	2.33937300
C	–3.53771800	–2.71643000	3.28299300
H	–4.07060700	–2.84159100	1.21132400
C	–2.83785400	–2.01300400	4.25769500
H	–1.59366400	–0.30498200	4.66647200

H	–4.08450800	–3.61413000	3.54821300
H	–2.83630200	–2.36146300	5.28418700
C	–3.84492600	–1.51210900	–1.30909400
C	–5.19210700	–1.54206600	–0.91799600
C	–3.49959400	–2.08600200	–2.53780700
C	–6.15711500	–2.14854200	–1.71357300
H	–5.49468000	–1.08361200	0.01996400
C	–4.46541900	–2.69102900	–3.33917400
H	–2.47596000	–2.03437200	–2.89509200
C	–5.79235400	–2.72934300	–2.92567300
H	–7.19209600	–2.16489800	–1.39179900
H	–4.17961300	–3.12576100	–4.29000600
H	–6.54282900	–3.20133100	–3.54924400
C	–2.90459100	1.23434700	–0.39660100
C	–3.47035500	1.97180600	0.65391600
C	–2.74103300	1.86990800	–1.63644900
C	–3.84614800	3.30005600	0.47867300
H	–3.63789600	1.50631700	1.61886300
C	–3.11352800	3.19869200	–1.81049900
H	–2.32109900	1.32540000	–2.47574300
C	–3.66396700	3.91774800	–0.75443500
H	–4.28390200	3.84962300	1.30430900
H	–2.97196800	3.67180000	–2.77563300
H	–3.95458100	4.95290400	–0.89326400

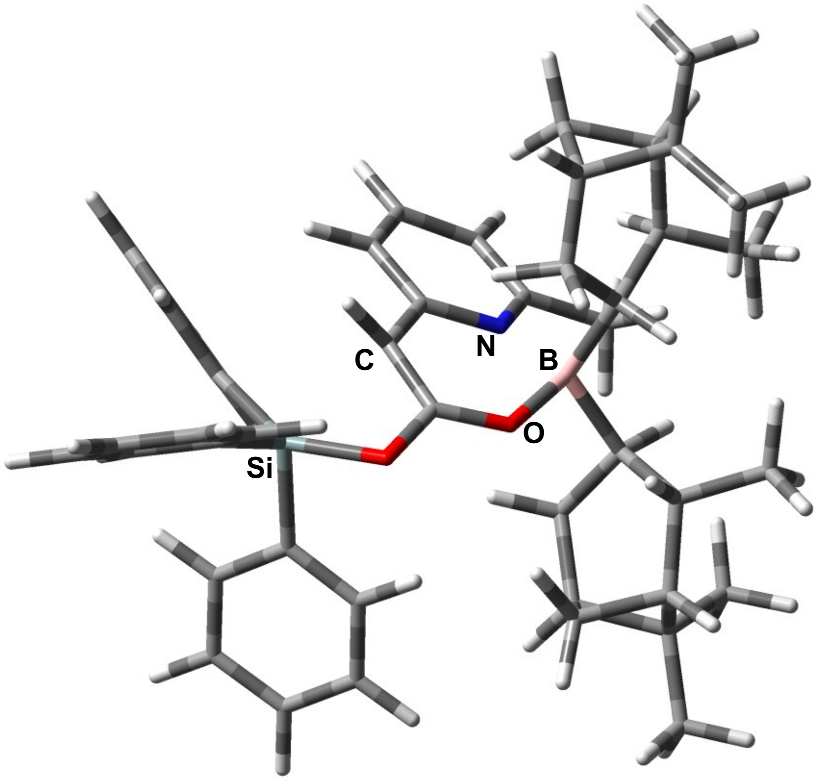

Figure S98. Optimized structure **TS3B** [M06-2X/6–311+G(d,p)] (PCM, toluene).

Table S29. Cartesian coordinates of the optimized structure **TS3B** [M06-2X/6–311+G(d,p)] (PCM, toluene).

Atomic symbol	x	y	z
N	1.13184100	–0.50364100	1.43926200
C	2.47720700	4.57863300	–0.16899600
C	3.00518800	2.20928300	–1.12808500
H	2.61191100	1.60332400	–1.95485600
C	2.51699500	3.63931500	–1.41621300
H	3.00647700	4.03641600	–2.31172600
C	0.97440100	3.61597500	–1.37947200
H	0.50057700	4.43293300	–1.92230500
H	0.52830300	2.66846300	–1.67895500
C	1.73924600	–0.49770100	2.66393100
C	1.26984600	–1.29236100	3.69940400
H	1.78609900	–1.26096000	4.64859600
C	4.12321100	–3.17538500	0.11965300
H	4.90529300	–3.57564600	0.77394400
C	2.86899600	–1.03013700	–0.57481500
H	3.48246400	–0.45245400	–1.27160200
C	–0.00512400	–1.25008600	1.28988600
C	2.43812700	1.64154500	0.22770700
H	3.29548000	1.64703900	0.89670300
C	3.88576700	–1.68814100	0.41935500
H	3.45399900	–1.70090300	1.42587800

C	2.74303200	–3.87545200	0.12173900
H	2.78628500	–4.94616900	0.31087800
H	1.97793700	–3.42680400	0.76501000
C	0.17010900	–2.12207500	3.51632400
H	–0.18275500	–2.76007600	4.31799200
C	2.28668900	6.03748900	–0.59334400
H	3.22331000	6.43758500	–0.99432200
H	2.00479800	6.64824900	0.27016400
H	1.51831200	6.16284100	–1.35727000
C	1.11949400	3.88297500	0.13940500
H	0.35159900	4.48202800	0.63913100
C	2.93339100	0.36586800	2.96398600
H	3.29922700	0.11618000	3.95834600
H	2.66194500	1.42136100	2.95760200
H	3.74271000	0.21643500	2.25736000
C	2.12540700	–2.08018000	–1.47120400
H	1.07063800	–2.16117900	–1.20309700
H	2.14762200	–1.73265300	–2.50950200
C	2.68178200	–3.50176900	–1.37726500
H	2.16122900	–4.17784100	–2.06291000
C	4.23432700	–3.52999500	–1.39815700
C	4.53361600	2.14004000	–1.21473400
H	4.87845000	2.61003300	–2.14053800
H	4.89597900	1.11203000	–1.21643200
H	5.01138400	2.65888600	–0.37945900
C	1.33425800	2.55352800	0.86248900
H	0.36709700	2.03564400	0.88533600
H	1.58076700	2.76958700	1.90778400
C	4.96807000	–2.63157100	–2.38975200
H	4.63346200	–1.59618600	–2.37907000
H	4.81745300	–3.01754200	–3.40349600
H	6.04502900	–2.64239000	–2.19515200
C	5.21649600	–0.93511500	0.50454600
H	5.84702900	–1.36437200	1.28895700
H	5.08098400	0.12656200	0.72353800
H	5.76544300	–1.00004500	–0.43760900
C	–0.47641400	–2.09176100	2.30542300
H	–1.37179000	–2.67217100	2.11851600
C	4.77498200	–4.95149200	–1.58164700
H	5.85460100	–4.96829200	–1.40316600

H	4.60066100	–5.29214700	–2.60695600
H	4.31603000	–5.67483100	–0.90658400
C	3.57486000	4.54890200	0.89081400
H	3.75757800	3.55842800	1.30362000
H	3.29972000	5.20953800	1.72008400
H	4.51746800	4.92003600	0.47642900
B	1.87337100	0.11920300	0.04596200
C	–0.78819900	–1.13277300	0.08427400
C	–0.40897700	–0.19727500	–0.90376700
O	–1.38196400	0.15410900	–1.67213600
O	0.71925200	0.37486000	–0.95723000
H	–1.26587200	–2.03745200	–0.28352700
Si	–2.80341400	–0.15929800	–0.43691900
C	–3.44599900	–1.03062500	1.15715700
C	–3.24334000	–0.44746800	2.41537300
C	–3.94644700	–2.33959600	1.12754800
C	–3.54354500	–1.12962300	3.59201600
H	–2.83407800	0.55699000	2.48305300
C	–4.23807600	–3.03441100	2.29961300
H	–4.11357400	–2.83061800	0.17279300
C	–4.04080400	–2.42829100	3.53787800
H	–3.38133000	–0.64966800	4.55099900
H	–4.62591000	–4.04566200	2.24547800
H	–4.27449400	–2.96356500	4.45109600
C	–3.92835900	–0.88204700	–1.77113400
C	–5.29812000	–1.00877500	–1.49457300
C	–3.48339100	–1.25009100	–3.04616500
C	–6.18964300	–1.47717800	–2.45290700
H	–5.67763400	–0.74109700	–0.51278100
C	–4.36905700	–1.73904400	–4.00442100
H	–2.43469800	–1.14569600	–3.29691300
C	–5.72338200	–1.85048300	–3.71086900
H	–7.24502600	–1.55598000	–2.21853500
H	–4.00062300	–2.02619000	–4.98263000
H	–6.41423000	–2.22474900	–4.45780700
C	–3.09158600	1.66665400	–0.06690200
C	–4.25968800	2.03115300	0.62106000
C	–2.24293700	2.69239100	–0.49523800
C	–4.56643100	3.36497200	0.86681300
H	–4.94133900	1.26485100	0.97720200

C	–2.53337200	4.02914200	–0.23332300
H	–1.34851300	2.45004800	–1.05073300
C	–3.69810000	4.36891000	0.44554000
H	–5.47916800	3.62076200	1.39260600
H	–1.85003600	4.80079100	–0.57116500
H	–3.93155400	5.40895600	0.64345800

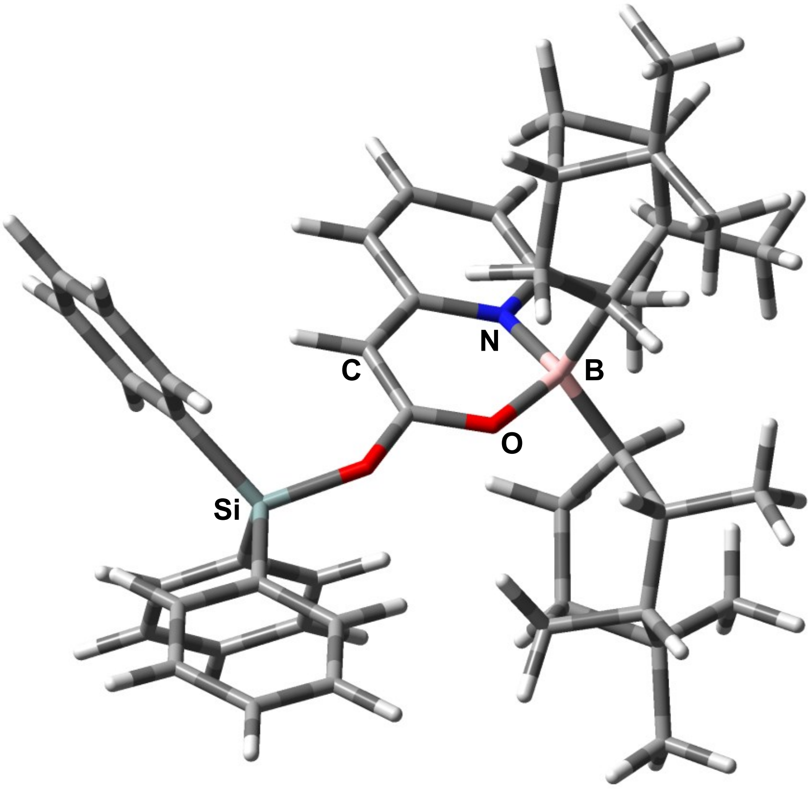

Figure S99. Optimized structure $\mathbf{P_B}$ [M06-2X/6–311+G(d,p)] (PCM, toluene).

Table S30. Cartesian coordinates of the optimized structure $\mathbf{P_B}$ [M06-2X/6–311+G(d,p)] (PCM, toluene).

Atomic symbol	x	y	z
N	1.88851000	–0.30235500	1.74764000
C	1.61943500	4.71058600	–0.38253400
C	2.23699400	2.38438000	–1.38976800
H	1.70651100	1.63067100	–1.98454400
C	1.38451600	3.65773400	–1.51279700
H	1.40638100	4.03554700	–2.54080300
C	0.00597500	3.36662300	–0.87488500
H	–0.78946900	4.02802700	–1.21477600
H	–0.32832200	2.33054300	–0.93706900
C	2.87971200	–0.13844700	2.67489500
C	2.87088000	–0.83626800	3.86925600
H	3.68393500	–0.68821600	4.56579200
C	4.66972400	–2.67414600	–0.47739600
H	5.69692500	–2.89859900	–0.16867900

C	2.91961200	–0.79078900	–0.70859000
H	3.12843200	–0.21349400	–1.61494600
C	0.80823500	–1.08041400	2.07180700
C	2.33811000	1.86819100	0.09369800
H	3.36572300	2.07945000	0.38560600
C	4.32094400	–1.21245400	–0.15668300
H	4.30040600	–1.19754300	0.93851500
C	3.52861400	–3.55268900	0.09027100
H	3.81395100	–4.58425400	0.28828900
H	3.00300200	–3.14880000	0.96252300
C	1.82620900	–1.71289500	4.16237500
H	1.82534000	–2.28161600	5.08479500
C	0.98787000	6.05585000	–0.75010200
H	1.59999800	6.56551900	–1.50079900
H	0.93447900	6.70206500	0.13189900
H	–0.02011900	5.95744900	–1.15444700
C	0.65500600	3.82355100	0.45505600
H	0.02626500	4.33480400	1.19216500
C	3.99672500	0.84663400	2.46624600
H	4.75503600	0.68197200	3.23043500
H	3.62084400	1.86548700	2.57499900
H	4.46503700	0.76218200	1.49298300
C	2.06707100	–2.01782200	–1.18673700
H	1.23773300	–2.21105200	–0.50257200
H	1.61390900	–1.77550200	–2.15352200
C	2.84057100	–3.33430800	–1.27642700
H	2.21444500	–4.13692500	–1.67892400
C	4.25035100	–3.15452500	–1.90266900
C	3.60831000	2.57838800	–2.04428800
H	3.49265100	2.98269100	–3.05447100
H	4.14906500	1.63429400	–2.12871400
H	4.23578400	3.27032100	–1.47687600
C	1.40028300	2.64290500	1.08051900
H	0.64991800	1.96392700	1.50460200
H	1.97987100	3.01226100	1.93467400
C	4.40148100	–2.26732200	–3.13511800
H	3.93358700	–2.76103700	–3.99347200
H	5.45932000	–2.12494600	–3.37853200
H	3.94551300	–1.28506000	–3.03146100
C	5.44099100	–0.27217900	–0.61080200

H	6.38201600	–0.52372100	–0.11215200
H	5.21422700	0.77531800	–0.39598600
H	5.60227000	–0.34874300	–1.68900800
C	0.79052800	–1.81773200	3.27389400
H	–0.06576500	–2.44972200	3.46941500
C	4.90221500	–4.50227100	–2.22918100
H	5.96169600	–4.35977400	–2.46464900
H	4.42321900	–4.95174900	–3.10465300
H	4.83784400	–5.21874900	–1.40929100
C	3.01265000	4.99344300	0.17312800
H	3.53640800	4.10059700	0.50988500
H	2.93263600	5.67635500	1.02594100
H	3.63371200	5.48501200	–0.58219900
B	2.01701600	0.26237100	0.17725300
C	–0.34969200	–1.13278900	1.24175500
C	–0.40157300	–0.41770900	0.07976500
O	–1.49793700	–0.30785300	–0.67326900
O	0.60164800	0.22335700	–0.39884100
H	–1.19361500	–1.71805000	1.57025500
Si	–3.13141500	–0.40095700	–0.23732900
C	–3.42602500	0.88546600	1.08697500
C	–4.64239100	0.93035500	1.78135400
C	–2.46854900	1.86876500	1.36167400
C	–4.89124000	1.92196000	2.72521000
H	–5.40767500	0.18325400	1.58759800
C	–2.71160100	2.85976300	2.30772800
H	–1.52190800	1.87202400	0.83150100
C	–3.92321200	2.88657000	2.99111000
H	–5.83759100	1.94222500	3.25305900
H	–1.95475300	3.60973800	2.50757500
H	–4.11405800	3.65835200	3.72793300
C	–3.61381700	–2.10814700	0.36166000
C	–3.80820800	–2.39919100	1.71844600
C	–3.76563900	–3.14600100	–0.56815700
C	–4.14385500	–3.68435500	2.13384900
H	–3.69332300	–1.61489800	2.46109600
C	–4.09668000	–4.43301800	–0.15685800
H	–3.62764900	–2.94615300	–1.62676000
C	–4.28792100	–4.70211500	1.19536100
H	–4.29454800	–3.89127400	3.18706200

H	–4.20834900	–5.22369100	–0.88959800
H	–4.55056500	–5.70331400	1.51702600
C	–3.99703400	–0.02461300	–1.84429400
C	–3.35982000	0.71180600	–2.85054000
C	–5.32168900	–0.43041100	–2.04833500
C	–4.02871500	1.03112700	–4.02838500
H	–2.33079100	1.02836500	–2.71471000
C	–5.99366800	–0.10939400	–3.22352000
H	–5.83420700	–1.01327300	–1.28791300
C	–5.34577700	0.62186400	–4.21487500
H	–3.52139600	1.59607900	–4.80184800
H	–7.01775800	–0.43330000	–3.36812600
H	–5.86618500	0.86919800	–5.13294700

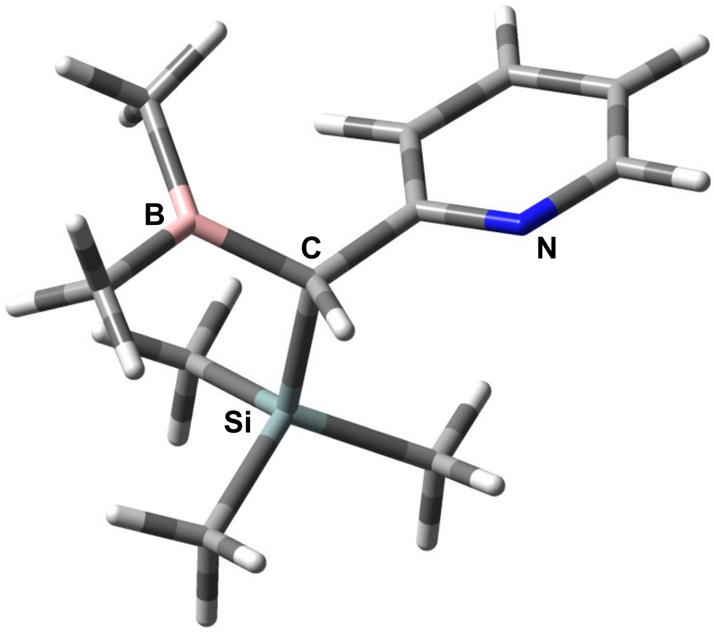

Figure S100. Optimized structure **I'$_A$** [M06-2X/6–311+G(d,p)] (PCM, toluene).

Table S31. Cartesian coordinates of the optimized structure **I'$_A$** [M06-2X/6–311+G(d,p)] (PCM, toluene).

Atomic symbol	x	y	z
Si	1.30053200	–1.08745700	0.19403700
N	–1.83921200	–0.17692700	–1.38564300
C	–3.14475900	–0.33087900	–1.16145600
C	–3.76070900	–0.05725300	0.05265300
H	–4.82552100	–0.20628500	0.17670900
C	0.75826400	2.73137800	0.72610300
H	1.35395000	3.64642800	0.69445800
C	–1.06368600	0.27583300	–0.38953000
C	2.55921800	1.93956400	–1.12543000
H	2.50257300	2.92036400	–1.61344200

C	–2.96082400	0.40757400	1.09086400
H	–3.38936900	0.63272400	2.06100800
C	–1.60302100	0.57666400	0.87065200
H	–0.95961000	0.93124000	1.66501300
B	1.20264600	1.68072400	–0.36423200
C	0.40147600	0.37921400	–0.68631100
H	0.54486600	0.10959400	–1.73973300
C	0.15371200	–2.57007600	0.10791400
H	–0.12357900	–2.78224300	–0.92802600
H	0.63968400	–3.45646900	0.52430700
H	–0.76778900	–2.38873400	0.66743000
C	1.67654800	–0.66629500	1.98916300
H	2.16102200	–1.51814200	2.47500800
H	2.35583300	0.18794600	2.06475800
H	0.77145600	–0.43494400	2.55619300
C	2.91321300	–1.47161000	–0.68843900
H	2.75733500	–1.60567000	–1.76196100
H	3.65432200	–0.68213600	–0.54709800
H	3.33369200	–2.40064200	–0.29251700
H	–0.30018700	2.99729700	0.64730300
H	0.88602800	2.29855300	1.72722800
H	2.82528700	1.19639300	–1.87909700
H	3.38655200	2.02478800	–0.40913400
H	–3.72933300	–0.69548700	–2.00135900

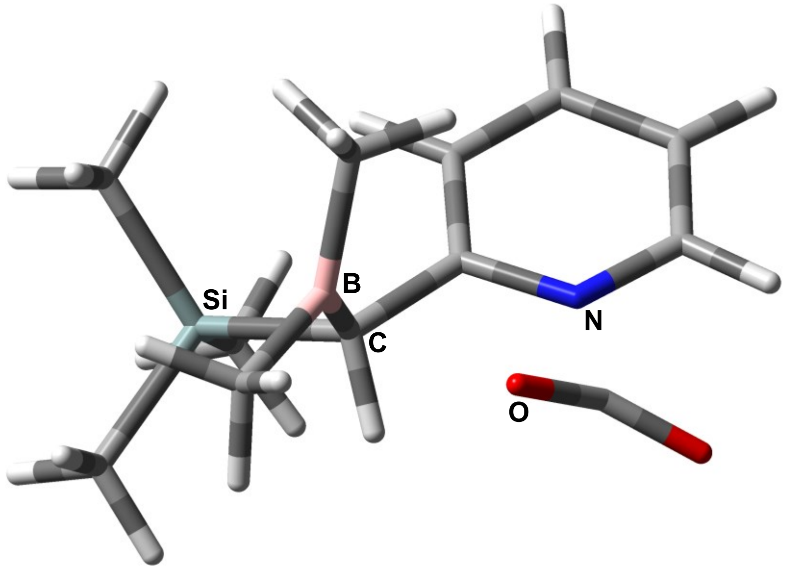

Figure S101. Optimized structure **TS'A** [M06-2X/6–311+G(d,p)] (PCM, toluene).

Table S32. Cartesian coordinates of the optimized structure **TS'A** [M06-2X/6–311+G(d,p)] (PCM, toluene).

Atomic symbol	x	y	z
Si	2.20469800	–0.54416800	–0.41999700

N	–1.89648700	–0.11454900	–0.35878500
C	–3.00754200	–0.84692100	–0.21955000
C	–2.98601700	–2.10844100	0.33963100
H	–3.90228900	–2.67291100	0.44238500
C	0.04994600	1.18071900	2.30992700
H	0.04445200	2.07134000	2.94183700
C	–0.68101600	–0.58579200	–0.00821300
C	1.38765000	2.76679500	0.54092000
H	0.87845200	3.64327300	0.95109500
C	–1.76052000	–2.60285600	0.77584800
H	–1.69717600	–3.57764500	1.24509200
C	–0.61483400	–1.84670100	0.59505300
H	0.34390200	–2.22884300	0.91890100
B	0.54740700	1.46977900	0.83994500
C	0.49803300	0.29742500	–0.24403600
H	0.36544300	0.79181700	–1.21437200
C	–2.21562900	1.61122400	–0.72098800
C	2.05838600	–2.10443600	–1.46282200
H	1.54189500	–1.88601500	–2.40178700
H	3.05951800	–2.46806000	–1.71144400
H	1.52319200	–2.91680800	–0.96851100
C	2.96585800	–0.93210000	1.25718900
H	3.93213300	–1.42600900	1.12034900
H	3.14373000	–0.00982000	1.81835500
H	2.34519700	–1.58227400	1.87862500
C	3.34032000	0.64332800	–1.32856600
H	2.89016000	0.99071200	–2.26228400
H	3.58866100	1.51908400	–0.72653600
H	4.27401400	0.13152700	–1.57861600
H	–0.94528600	0.72832500	2.34521400
H	0.73416000	0.45406400	2.77036700
H	1.59117400	2.94434400	–0.51713500
H	2.35148400	2.69999300	1.06407500
O	–1.43081400	2.26973800	–0.09306000
O	–3.15429200	1.65583500	–1.45218100
H	–3.91718600	–0.37283700	–0.56831800

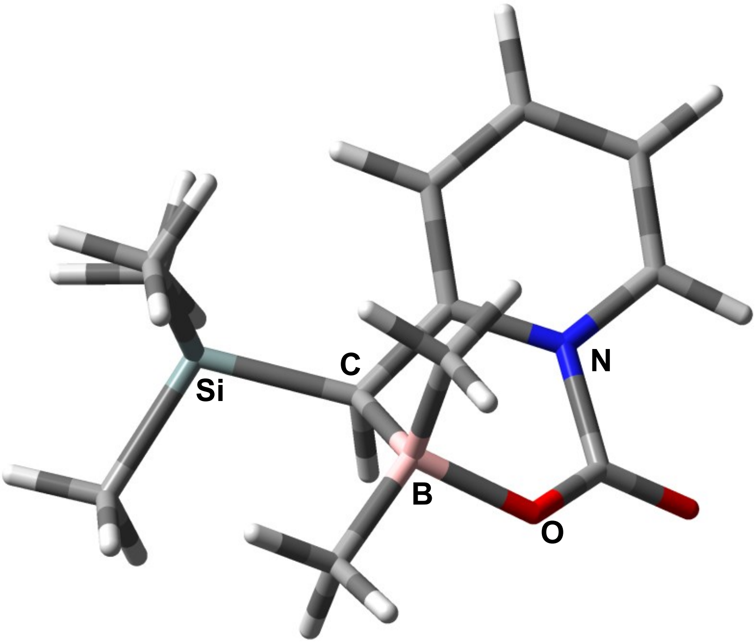

Figure S102. Optimized structure **P'$_A$** [M06-2X/6–311+G(d,p)] (PCM, toluene).

Table S33. Cartesian coordinates of the optimized structure **P'$_A$** [M06-2X/6–311+G(d,p)] (PCM, toluene).

Atomic symbol	x	y	z
Si	–2.23904900	0.52548300	–0.28347000
N	1.89608800	0.07517400	–0.36600200
C	3.05699300	0.74842000	–0.18736400
C	3.05980400	2.03285700	0.28542800
H	3.99663000	2.55196800	0.42698900
C	0.21105500	–1.23610700	2.10009500
H	–0.63709700	–0.79096700	2.63153200
C	0.66592000	0.63016300	–0.15292100
C	–1.26315200	–2.74638500	0.41835900
H	–0.85681300	–3.67399500	0.83599000
C	1.82890500	2.62253300	0.59142800
H	1.79383800	3.62801900	0.99383700
C	0.65610700	1.93183000	0.37016500
H	–0.29641300	2.39005000	0.59184100
B	–0.18164600	–1.57159800	0.57028700
C	–0.50299400	–0.22135300	–0.38782400
H	–0.42412300	–0.64275400	–1.40023200
C	2.04852700	–1.41526300	–0.65792300
O	1.13637100	–2.12816100	–0.15561500
O	3.03002000	–1.72800900	–1.27170500
C	–2.34435700	2.15395700	–1.24059400
H	–1.87613200	2.05040700	–2.22399200
H	–3.39739800	2.40139200	–1.40452400
H	–1.88632700	3.00964900	–0.74121400

C	–2.77468300	0.79950000	1.49752200
H	–3.74661600	1.30001900	1.52580900
H	–2.87252100	–0.15728900	2.01655100
H	–2.06512600	1.41020800	2.06242000
C	–3.43597800	–0.64231900	–1.13923200
H	–3.08515400	–0.89314300	–2.14428200
H	–3.58089500	–1.57397900	–0.59223400
H	–4.40865600	–0.15236400	–1.24084600
H	0.47439300	–2.15512200	2.63422100
H	1.05653900	–0.54640100	2.21888300
H	–1.54546100	–2.94724100	–0.62042400
H	–2.17855100	–2.52186000	0.97680000
H	3.95016600	0.19077200	–0.43224600

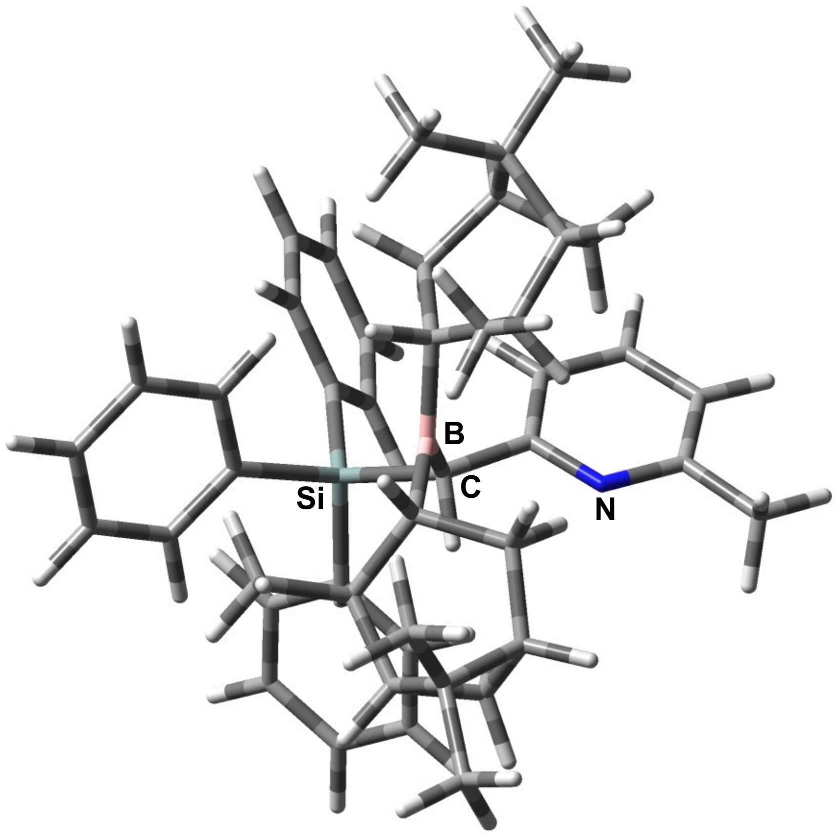

Figure S103. Optimized structure **I'**$_{\mathbf{B}}$ [M06-2X/6–311+G(d,p)] (PCM, toluene).

Table S34. Cartesian coordinates of the optimized structure **I'**$_{\mathbf{B}}$ [M06-2X/6–311+G(d,p)] (PCM, toluene).

Atomic symbol	x	y	z
Si	–1.56237800	–1.14015800	–0.30028000
N	0.76588300	0.11377500	2.78693400
C	–3.31574100	–0.19785400	1.81707300
H	–2.44935700	0.28367300	2.25846200
C	–4.53913500	–0.09185500	2.47736800
H	–4.60565700	0.47436500	3.39927100

C	–1.74897100	–0.51332500	–2.07582300
C	–0.56501000	4.99945200	0.29396100
C	–1.29608900	–3.01460100	–0.38440900
C	–2.96676500	–0.03084400	–2.57237500
H	–3.82412000	0.05235800	–1.91272200
C	–0.66400800	–0.56951300	–2.96332600
H	0.30326000	–0.91918200	–2.61782400
C	–1.12705800	2.67363700	–0.74880500
H	–1.77602200	1.82954600	–0.47745400
C	–3.19399400	–0.92198200	0.62670400
C	–1.60958300	3.84454300	0.11916400
H	–2.62204600	4.14274000	–0.17268700
C	–1.35757200	3.48370100	1.60354400
H	–1.97727700	4.04117000	2.30294500
H	–1.39112700	2.42555200	1.87014600
C	1.40578700	–0.28911400	3.88841300
C	–5.66733400	–0.71365300	1.95692900
H	–6.62022200	–0.62949600	2.46673300
C	1.77472700	–1.61896300	4.08434000
H	2.29048200	–1.91628400	4.98910200
C	4.51257200	0.08620700	0.01665800
H	5.36428900	0.66025800	0.39767900
C	–1.82053500	–3.83074000	0.62908200
H	–2.39607000	–3.38537100	1.43485900
C	–0.56877900	–3.63481900	–1.40802200
H	–0.16467300	–3.04654900	–2.22408700
C	2.11623000	0.25108000	–0.93367600
H	2.05515800	0.61908500	–1.96803800
C	0.47163400	–0.76326900	1.81493500
C	0.36723500	2.30189000	–0.47135100
H	0.92438400	2.67955000	–1.33167500
C	3.34748800	1.02964700	–0.30969900
H	3.04967700	1.43323500	0.66520900
C	–5.56660400	–1.45403600	0.78093500
H	–6.44029400	–1.95051400	0.37459400
C	–4.34269500	–1.55955000	0.13099900
H	–4.27931500	–2.15534200	–0.77609800
C	3.94793000	–1.05527000	0.89146500
H	4.69778700	–1.56395100	1.49469900
H	3.09809900	–0.79629600	1.52395900

C	1.46138300	–2.53945300	3.09467200
H	1.72851400	–3.58396800	3.20978500
C	–0.79089000	–0.18399800	–4.29455500
H	0.06517400	–0.23834900	–4.95761700
C	–3.10075500	0.35789600	–3.90269400
H	–4.05361500	0.73080300	–4.26125600
C	–1.23256900	6.24615300	0.88251900
H	–1.81825200	6.75627600	0.11146300
H	–0.47285800	6.94676000	1.24302700
H	–1.90165400	6.02170100	1.71367900
C	–0.35084600	–5.01025900	–1.40938100
H	0.21703400	–5.46612200	–2.21242600
C	0.04536100	4.09243700	1.39713000
H	0.52853700	4.58801400	2.24520100
C	1.71327900	0.77749900	4.90367700
H	0.79887500	1.30640300	5.17844800
H	2.39947900	1.51162200	4.47443900
H	2.16621600	0.35725800	5.80170300
C	–2.01558600	0.27637600	–4.76892400
H	–2.12010200	0.57893300	–5.80435500
C	–1.60933500	–5.20573600	0.63174700
H	–2.02348600	–5.81425900	1.42748100
C	2.31404500	–1.29518000	–1.00872700
H	1.50547800	–1.81192100	–0.48778400
H	2.25354300	–1.62302600	–2.05325800
C	3.64793300	–1.78104900	–0.44243000
H	3.71498200	–2.87317700	–0.46739400
C	4.82487100	–0.98364900	–1.07737400
C	–1.35569200	2.94794700	–2.23591900
H	–2.42056800	3.08236100	–2.44470400
H	–0.99603500	2.11816900	–2.84759300
H	–0.83158900	3.85255100	–2.55213100
C	0.96063600	3.01600900	0.80818800
H	1.14703500	2.29185500	1.60995700
H	1.92814600	3.45570800	0.54747600
C	4.75237600	–0.64566700	–2.56335000
H	3.81799800	–0.16973200	–2.85727500
H	4.85864200	–1.56402500	–3.15087000
H	5.57342400	0.02049700	–2.84542100
C	3.80271400	2.21541400	–1.16486300

H	4.61180300	2.75236600	–0.66069800
H	2.99289300	2.92613700	–1.34135200
H	4.17341000	1.88738500	–2.13768600
C	0.80953600	–2.11524200	1.94578300
H	0.57912200	–2.82757100	1.16818700
C	6.17597500	–1.65303900	–0.81541000
H	6.99152200	–0.97618100	–1.08807500
H	6.27277500	–2.55684400	–1.42505700
H	6.31396600	–1.93703700	0.22844300
C	0.34202500	5.46423100	–0.84243700
H	0.89489100	4.65967300	–1.32275700
H	1.07204300	6.17875000	–0.44690000
H	–0.23728100	5.98298600	–1.61226800
C	–0.86729800	–5.79830300	–0.38626700
H	–0.69968200	–6.86916300	–0.38608400
B	0.74562400	0.75970300	–0.29403100
C	–0.22424300	–0.13296500	0.61819200
H	–0.86324300	0.63274200	1.07016400

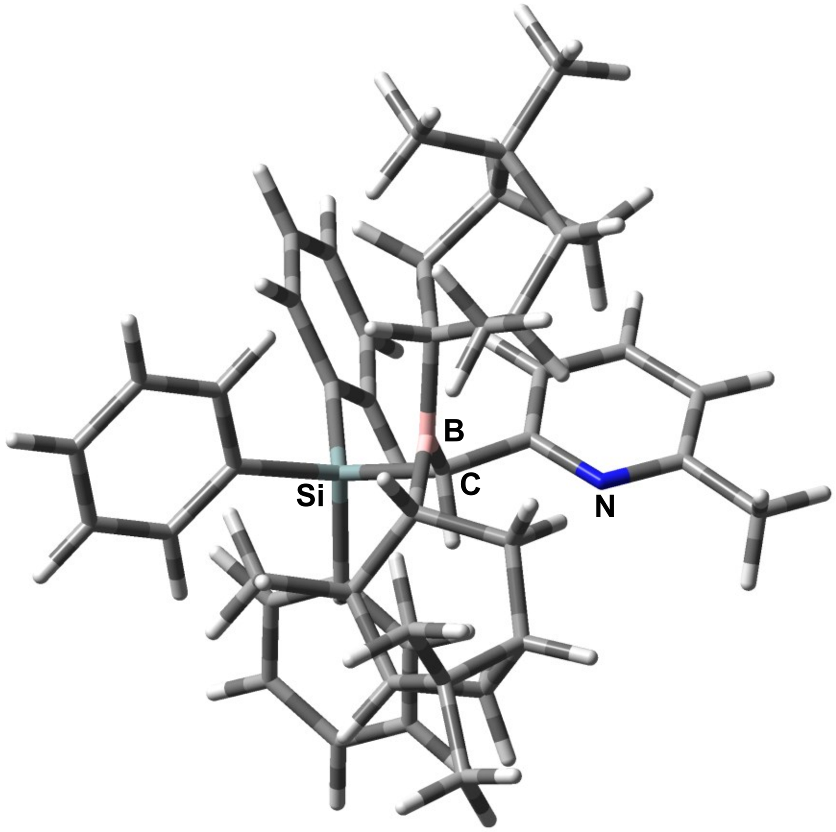

Figure S104. Optimized structure **TS'$_B$** [M06-2X/6–311+G(d,p)] (PCM, toluene).

Table S35. Cartesian coordinates of the optimized structure **TS'$_B$** [M06-2X/6–311+G(d,p)] (PCM, toluene).

Atomic symbol	x	y	z
Si	–1.50773200	1.13457700	0.56476000

N	1.44820900	1.64437700	–2.19798700
C	–3.21672500	0.85406400	–1.81692700
H	–2.34077200	0.63538100	–2.41825900
C	–4.44938600	0.89889600	–2.46503400
H	–4.50089400	0.71163400	–3.53126000
C	–1.69965100	0.09955900	2.12999800
C	–1.96553100	–4.40931000	–1.00975200
C	–1.41752800	2.95525500	1.08109000
C	–2.80614500	–0.72411400	2.37558100
H	–3.62663200	–0.75885300	1.66767300
C	–0.67089900	0.10499300	3.08162600
H	0.19517700	0.74294700	2.94224500
C	–0.56836700	–2.84397600	0.53920800
H	–0.79355700	–2.03616200	1.24229000
C	–3.11078600	1.08687500	–0.44164700
C	–1.91812100	–3.52915100	0.28141100
H	–2.28921300	–3.99185200	1.20237300
C	–2.84283100	–2.52458600	–0.44617400
H	–3.90729100	–2.72145300	–0.32314800
H	–2.65289900	–1.47018100	–0.25062000
C	2.35990600	2.51750600	–2.67978000
C	–5.60563400	1.18295000	–1.74810500
H	–6.56515100	1.21368400	–2.25123500
C	2.79318100	3.54855100	–1.86257200
H	3.51782600	4.25512300	–2.24427100
C	4.73217000	–0.48403500	0.11669500
H	5.63763300	–0.58281200	–0.49205500
C	–1.51731600	3.93765600	0.08383900
H	–1.63654800	3.64115300	–0.95562100
C	–1.30042000	3.37899000	2.40829000
H	–1.25164500	2.64989600	3.20972400
C	2.21686300	–0.99498400	0.20993900
H	2.20449000	–2.05104800	0.47258800
C	0.94064300	1.68769700	–0.93893000
C	0.02370500	–2.22846000	–0.78914200
H	0.86150200	–2.88328000	–1.05069500
C	3.49138600	–0.89202800	–0.69599300
H	3.35588200	–0.09836900	–1.43835600
C	–5.52229700	1.43977500	–0.38202500
H	–6.41609000	1.67821900	0.18303500

C	–4.28850000	1.39883500	0.25619500
H	–4.24154600	1.62022700	1.31942200
C	4.42701700	0.89172400	0.75178600
H	5.30876600	1.47047500	1.01960400
H	3.74038800	1.52379000	0.18879300
C	2.30390400	3.63954300	–0.56689200
H	2.64310300	4.43228900	0.08940300
C	–0.71892400	–0.70583000	4.21093000
H	0.09871900	–0.69063600	4.92263700
C	–2.86330800	–1.53569400	3.50520400
H	–3.72385200	–2.17530200	3.66499000
C	–3.20447400	–5.30903000	–1.00298600
H	–3.06282300	–6.13791400	–0.30222800
H	–3.36739800	–5.73534400	–1.99774100
H	–4.11285700	–4.77885500	–0.71359900
C	–1.25501200	4.73583500	2.72880800
H	–1.16602100	5.04262000	3.76471600
C	–2.23299100	–3.07004900	–1.75370700
H	–2.86158000	–3.11111300	–2.64870900
C	2.88674700	2.32809700	–4.07025600
H	2.10628500	2.52118400	–4.80645300
H	3.22884400	1.30096100	–4.21689000
H	3.72193200	3.00601500	–4.23504000
C	–1.81443600	–1.53764900	4.41929700
H	–1.85251100	–2.17893800	5.29239200
C	–1.47225900	5.29036900	0.39636300
H	–1.55269400	6.03139000	–0.39075100
C	2.36858600	–0.24652200	1.56472100
H	1.81317200	0.69652300	1.55521700
H	1.91408800	–0.85856900	2.35329300
C	3.81976700	0.09066500	1.92854000
H	3.89174300	0.51126100	2.93677500
C	4.80771000	–1.05967400	1.57097700
C	0.40248400	–3.77736600	1.27626400
H	–0.13151500	–4.32786100	2.05693900
H	1.19818000	–3.21873000	1.77369000
H	0.86766900	–4.50450400	0.60618700
C	–0.94568000	–2.28850600	–2.01065200
H	–1.23934500	–1.28617200	–2.33582000
H	–0.40465700	–2.71618200	–2.85899500

C	4.44829500	–2.50638500	1.90255300
H	3.45985600	–2.81350100	1.57367100
H	4.49651400	–2.65163100	2.98694000
H	5.17600900	–3.18834900	1.45134600
C	3.71932000	–2.19523700	–1.46269900
H	4.59253600	–2.10990300	–2.11641900
H	2.85594500	–2.43790000	–2.08425600
H	3.89586500	–3.02606400	–0.77265500
C	1.39702800	2.70410700	–0.10053600
H	1.02788100	2.76604500	0.91135200
C	6.19661300	–0.80813000	2.17289700
H	6.91964100	–1.51096000	1.74756600
H	6.17219300	–0.97052000	3.25497100
H	6.57048900	0.20014800	1.99529800
C	–0.77513100	–5.26289800	–1.43525200
H	0.14970200	–4.69955100	–1.54084600
H	–0.98914600	–5.73150800	–2.40162600
H	–0.60301300	–6.06592500	–0.71148600
C	–1.33320600	5.69247000	1.72379300
H	–1.30043600	6.74724400	1.97169900
B	0.77575200	–0.83663800	–0.51164100
C	–0.03943800	0.60854300	–0.58015800
H	–0.62817800	0.44511700	–1.48993600
C	0.99823900	0.42399100	–3.12027000
O	1.32064900	–0.63823800	–2.59448600
O	0.43145500	0.77387400	–4.12544800

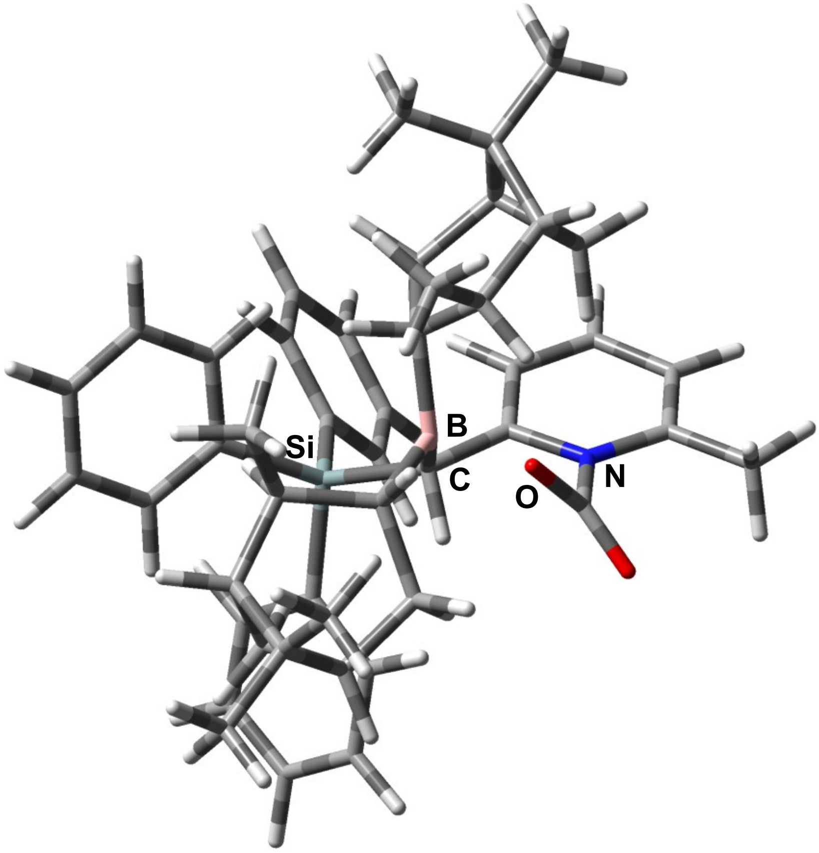

Figure S105. Optimized structure **P'B** [M06-2X/6–311+G(d,p)] (PCM, toluene).

Table S36. Cartesian coordinates of the optimized structure **P'B** [M06-2X/6–311+G(d,p)] (PCM, toluene).

Atomic symbol	x	y	z
Si	1.19536600	1.46808600	–0.50803400
N	–1.63344700	0.99683700	2.45290000
C	3.03468000	1.49397900	1.79028300
H	2.31969500	0.90554200	2.35551000
C	4.22146200	1.85541700	2.42476400
H	4.39544300	1.55409400	3.45130300
C	1.64158600	0.45656000	–2.03227800
C	2.94618100	–3.89752100	0.91742200
C	0.64435500	3.17860900	–1.11739100
C	2.95208000	0.04216200	–2.29645000
H	3.75904300	0.33832100	–1.63442700
C	0.63256500	0.03453300	–2.90848600
H	–0.39504600	0.32655800	–2.72732600
C	1.29465200	–2.65530800	–0.67428200
H	1.34217900	–1.79011100	–1.33322700
C	2.77426400	1.86256300	0.46640100
C	2.75689400	–2.97008700	–0.32770800
H	3.31063400	–3.24769000	–1.23148300
C	3.30854400	–1.80746000	0.52910400

H	4.39250400	–1.69871700	0.50338100
H	2.85362600	–0.83224900	0.35587400
C	–2.64847600	1.60374700	3.13202900
C	5.17938500	2.59552200	1.74218600
H	6.10463100	2.87402900	2.23328000
C	–3.25100800	2.70460300	2.56102800
H	–4.05674200	3.19520200	3.08941900
C	–4.30057600	–1.80013000	–0.13291200
H	–5.00527900	–2.50337100	0.32422900
C	0.74137100	4.27810400	–0.25104700
H	1.16768600	4.14553700	0.73952300
C	0.11605400	3.39794600	–2.39466400
H	0.05386800	2.58085700	–3.10512900
C	–1.80966000	–1.33409300	–0.62931800
H	–1.25960000	–1.84669900	–1.42573700
C	–1.15834100	1.39949000	1.23361800
C	0.45549600	–2.27593700	0.59859600
H	–0.23971100	–3.11469100	0.74150900
C	–2.87517200	–2.36991800	–0.09205800
H	–2.68162600	–2.51687400	0.97405000
C	4.94245300	2.97858200	0.42456800
H	5.68214400	3.55858400	–0.11521700
C	3.75220000	2.62003000	–0.19793900
H	3.57834800	2.94089400	–1.22205800
C	–4.24664300	–0.35511400	0.42585100
H	–5.20202200	0.01069200	0.79921000
H	–3.47162500	–0.16416200	1.16902600
C	–2.83964300	3.13302800	1.30358200
H	–3.32905500	3.97575600	0.82959000
C	0.91012400	–0.79596900	–3.98835500
H	0.10745400	–1.12397000	–4.63952100
C	3.23760000	–0.79023100	–3.37570700
H	4.25701800	–1.11563500	–3.54969300
C	4.37902100	–4.43037900	0.98604200
H	4.52558400	–5.21587500	0.23778500
H	4.57449800	–4.86566400	1.97137700
H	5.12713000	–3.65629700	0.80810600
C	–0.33149300	4.66020200	–2.78262600
H	–0.73886300	4.80588800	–3.77667000
C	2.77357500	–2.59458700	1.74444700

H	3.31723200	–2.52946700	2.69283300
C	–3.12549300	1.05392200	4.44297400
H	–2.35025200	1.12590500	5.20473700
H	–3.39864800	0.00071000	4.35392800
H	–4.00067100	1.62029800	4.75529500
C	2.21659800	–1.21879700	–4.21787800
H	2.43741900	–1.87932500	–5.04857100
C	0.29885100	5.53956000	–0.63223900
H	0.38333100	6.37372800	0.05499800
C	–2.46541200	–0.10235200	–1.33496300
H	–1.92793100	0.81265700	–1.08498200
H	–2.37090700	–0.19551500	–2.42158400
C	–3.95128100	0.07562900	–1.03066200
H	–4.32459400	1.04590000	–1.37603600
C	–4.72909800	–1.18956400	–1.50236700
C	0.69307100	–3.78294700	–1.52247200
H	1.36943900	–4.01780200	–2.35094400
H	–0.26508800	–3.49799400	–1.95909300
H	0.53325600	–4.69813800	–0.94867600
C	1.30042100	–2.23212100	1.91901200
H	1.28041500	–1.23404200	2.37107600
H	0.84052300	–2.88526300	2.66695600
C	–4.31185600	–1.84982500	–2.81214200
H	–3.24130900	–2.03990400	–2.87701600
H	–4.59085100	–1.20794900	–3.65507100
H	–4.82925700	–2.80578200	–2.93857500
C	–2.78141600	–3.77035800	–0.71006600
H	–3.60256600	–4.39733600	–0.34717500
H	–1.84624500	–4.25393900	–0.42033900
H	–2.82130800	–3.76562400	–1.79994600
C	–1.81204700	2.48779700	0.64708700
H	–1.51231400	2.82277600	–0.33115100
C	–6.23976800	–0.95135100	–1.55374800
H	–6.76673200	–1.90281500	–1.67588100
H	–6.49481700	–0.31603600	–2.40791600
H	–6.62564700	–0.47192600	–0.65295200
C	1.99819600	–5.05711500	1.20448300
H	0.95024300	–4.76158600	1.21929500
H	2.23724000	–5.48707700	2.18333800
H	2.11800400	–5.84921900	0.45857700

C	–0.24783600	5.73121700	–1.89989300
H	–0.59380800	6.71329100	–2.20108400
B	–0.63914400	–1.04496400	0.49170200
C	–0.04781400	0.58400500	0.66401100
H	0.61888700	0.39661300	1.51490000
C	–1.17042600	–0.36506600	2.93025900
O	–1.27469200	–1.22081500	2.00515000
O	–0.84400200	–0.47508300	4.07753600

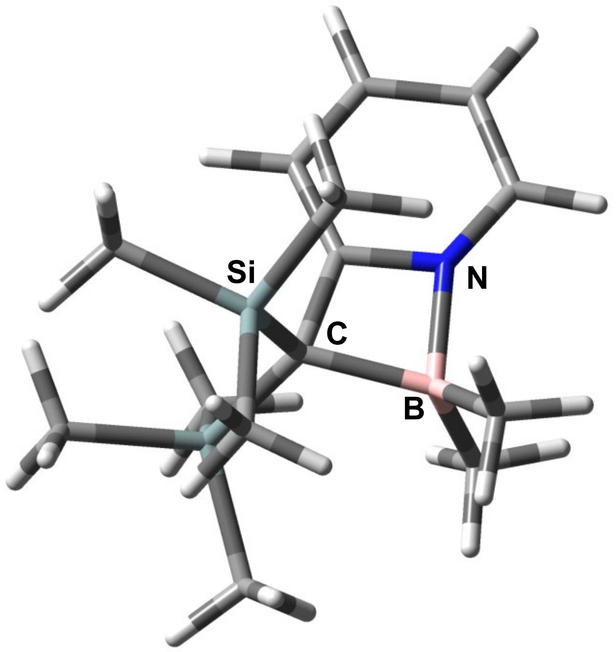

Figure S106. Optimized structure **E**$_{\mathbf{C}}$ [M06-2X/6–311+G(d,p)] (PCM, toluene).

Table S37. Cartesian coordinates of the optimized structure **E**$_{\mathbf{C}}$ [M06-2X/6–311+G(d,p)] (PCM, toluene).

Atomic symbol	x	y	z
Si	–0.73009500	1.78144600	–0.12300400
N	1.87346000	–0.25271400	0.58339500
C	3.20015600	–0.35014900	0.50076800
C	3.79981200	–0.42334300	–0.74478200
H	4.87582500	–0.49424900	–0.82513000
C	1.06419700	–0.21919700	–0.49493800
C	2.98949900	–0.41703000	–1.88520200
H	3.44866200	–0.48871900	–2.86463900
C	1.60947600	–0.32053300	–1.77684100
H	0.96803500	–0.31509200	–2.64964200
B	0.58459700	–0.31110800	1.60689600
C	–0.26951600	–0.04277600	0.12002100
C	–2.07624000	2.27213400	1.09367700
H	–2.35551600	3.31942200	0.94648300
H	–2.97355700	1.66613200	0.93358900

H	–1.76036700	2.14197500	2.12991000
C	–1.34212100	2.20194000	–1.86403800
H	–2.39629800	1.97680000	–2.02555600
H	–1.19991600	3.27573800	–2.02332700
H	–0.76273000	1.68167600	–2.63283800
C	0.82723200	2.84595900	0.02944500
H	1.36969300	2.81388400	–0.92112600
H	0.55059700	3.88885400	0.21173900
H	1.51498300	2.53313600	0.81458100
C	0.57794300	0.76403300	2.80665200
H	0.61098000	1.82127500	2.53893100
H	–0.32893900	0.61362300	3.40452400
H	1.42069000	0.57055700	3.48195000
C	0.55066500	–1.81393900	2.20583300
H	0.50360300	–2.61395100	1.46117800
H	1.45927300	–1.98030300	2.79833600
H	–0.29279700	–1.94508600	2.88930000
H	3.75719800	–0.37006600	1.43008800
Si	–1.59896000	–1.30084700	–0.35056000
C	–2.86064400	–0.67859100	–1.61036000
H	–3.47904900	0.13037300	–1.21286400
H	–2.39690500	–0.33085100	–2.53619600
H	–3.52791000	–1.51002200	–1.85947500
C	–0.81903100	–2.82548900	–1.14514500
H	–1.57379900	–3.61070300	–1.25078100
H	–0.44655900	–2.59276300	–2.14704500
H	0.01265800	–3.23184900	–0.56683900
C	–2.58973800	–1.78034300	1.18136500
H	–3.59658100	–2.09566800	0.89368700
H	–2.11952900	–2.60049200	1.72606600
H	–2.68230900	–0.93353000	1.86802000

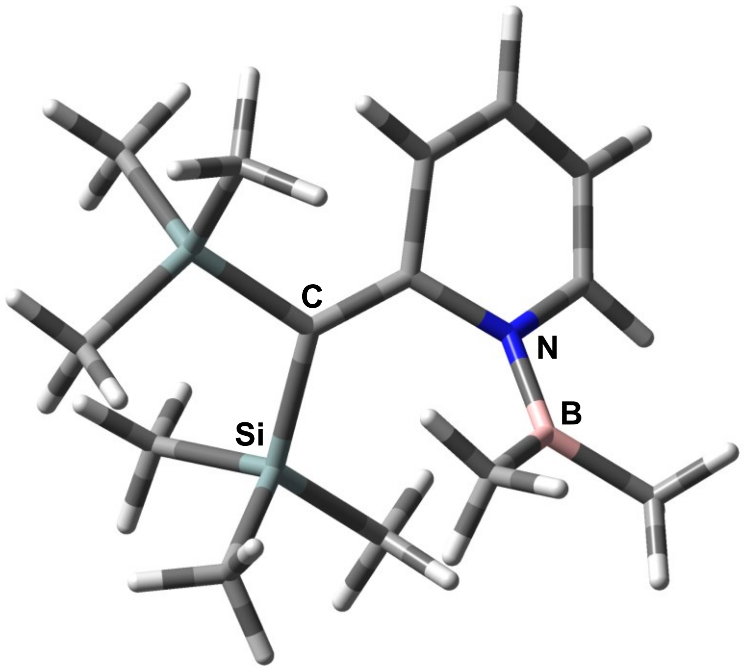

Figure S107. Optimized structure **I1$_C$** [M06-2X/6–311+G(d,p)] (PCM, toluene).

Table S38. Cartesian coordinates of the optimized structure **I1$_C$** [M06-2X/6–311+G(d,p)] (PCM, toluene).

Atomic symbol	x	y	z
N	1.93792800	0.39000500	0.03963000
C	2.90717600	1.09966700	–0.67561400
C	2.74799500	2.39419200	–1.00608300
H	3.52558500	2.91679000	–1.54525000
C	1.48615700	–1.17643000	2.13402300
H	0.56865800	–0.61981100	2.32380400
C	0.59603300	0.90116800	0.02265600
C	3.84087900	–1.23302000	0.80980800
H	4.14933000	–1.43380900	–0.21979800
C	1.55837700	3.07136700	–0.55506900
H	1.47539300	4.14621800	–0.66689500
C	0.54312500	2.36139300	–0.02116900
H	–0.37053900	2.85869800	0.27343300
B	2.37098600	–0.66083400	0.93998900
C	–0.49499200	0.08822700	–0.00549500
H	2.10853400	–1.16801500	3.03632100
H	1.21664700	–2.22793200	1.98023400
H	3.94656300	–2.15900000	1.37928300
H	4.56364800	–0.52088500	1.22618200
H	3.81733800	0.55990600	–0.89598100
Si	–2.20404700	0.82412900	0.33555700
C	–3.42689100	–0.52882500	0.81411000
H	–3.12669900	–1.01701000	1.74507600

H	–4.39951300	–0.05736700	0.98673500
H	–3.56409100	–1.30169800	0.05710600
C	–2.17053700	2.00231100	1.82196300
H	–3.07033600	1.84958200	2.42405700
H	–1.30418300	1.81222300	2.46140000
H	–2.15013400	3.05536700	1.53104500
C	–2.87245900	1.73726800	–1.17122700
H	–2.21197000	2.56112900	–1.45429400
H	–2.95558100	1.06918300	–2.03199800
H	–3.86380400	2.15107100	–0.96403000
Si	–0.48567000	–1.68957900	–0.62484000
C	–1.61512700	–1.71147700	–2.14219000
H	–1.68801600	–2.72993300	–2.53582900
H	–2.62873500	–1.36041600	–1.94012400
H	–1.19244900	–1.07664100	–2.92634800
C	–1.14398900	–2.93241500	0.64712400
H	–0.52945700	–3.83736700	0.64341400
H	–1.13688100	–2.52778200	1.66086700
H	–2.16938800	–3.22741700	0.41305000
C	1.16805600	–2.33303900	–1.27365000
H	0.94785000	–3.20470000	–1.89903100
H	1.68222100	–1.59484300	–1.89373300
H	1.85280100	–2.66744100	–0.49121200

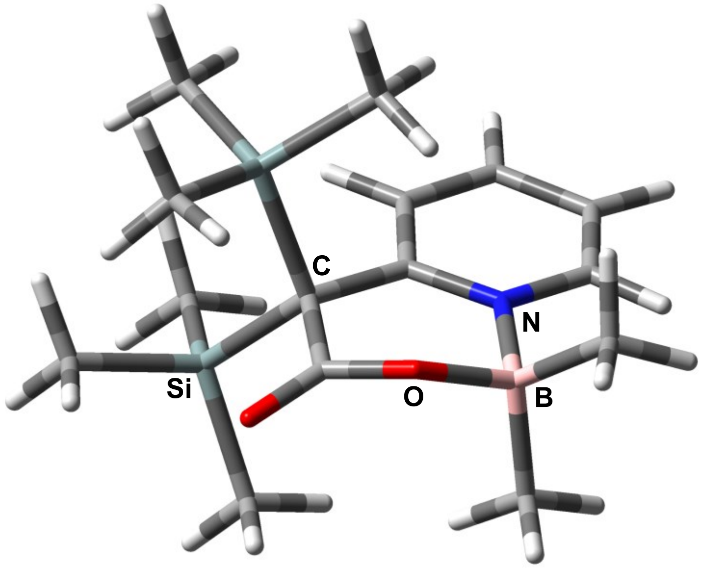

Figure S108. Optimized structure **I2c** [M06-2X/6–311+G(d,p)] (PCM, toluene).

Table S39. Cartesian coordinates of the optimized structure **I2c** [M06-2X/6–311+G(d,p)] (PCM, toluene).

Atomic symbol	x	y	z
Si	–1.18601000	0.51162600	1.70556500
N	1.97696400	0.24524400	–0.21673700

C	3.03478000	1.04035100	–0.46661100
C	2.90865300	2.36925000	–0.79530800
H	3.78941400	2.96595300	–0.98703100
C	0.71864500	0.72419400	–0.29556300
C	1.62262800	2.90129300	–0.86107700
H	1.46744200	3.94614700	–1.10216800
C	0.54179300	2.08175600	–0.61248400
H	–0.45965300	2.48025300	–0.65325000
B	2.30974600	–1.32441300	0.11719800
C	–0.45971700	–0.15257000	0.00564000
C	–0.12083300	–1.60009500	0.33553700
O	1.08398500	–1.84553600	0.79477100
O	–0.97533600	–2.45894300	0.27551500
C	3.52666400	–1.41385900	1.16468800
H	3.53691900	–2.42580900	1.58075100
H	4.51332000	–1.25226600	0.71961100
H	3.41586300	–0.71928300	2.00521600
C	2.54705500	–2.05199000	–1.30181600
H	3.39162600	–1.62284600	–1.85280300
H	2.76674900	–3.11241800	–1.14299700
H	1.66672700	–1.99880400	–1.95367300
C	–2.12738000	–0.85588000	2.56642000
H	–2.59345900	–0.44877600	3.46854500
H	–2.90432200	–1.28492000	1.93239700
H	–1.45652700	–1.66579400	2.86013800
C	0.30053500	1.00628300	2.74070000
H	1.02286100	0.18505900	2.77338500
H	0.80628200	1.89179400	2.34678900
H	–0.01213100	1.22358900	3.76559100
C	–2.31467100	1.99451200	1.45390700
H	–3.18013900	1.76367100	0.82938400
H	–2.69201500	2.28873800	2.43845100
H	–1.80364600	2.86337700	1.03457100
H	4.00105800	0.56070700	–0.38870900
Si	–1.78857400	–0.22039600	–1.41565900
C	–2.15102000	1.46329300	–2.18668200
H	–1.29681900	1.88921900	–2.71664500
H	–2.94035400	1.29388700	–2.92655800
H	–2.53130700	2.20153100	–1.47609200
C	–3.43000700	–0.88863000	–0.80099700

H	–4.12457000	–0.92005500	–1.64614800
H	–3.31205400	–1.89660000	–0.40433700
H	–3.88119100	–0.25811100	–0.03119700
C	–1.08906200	–1.30402500	–2.77552800
H	–1.80012900	–1.36298800	–3.60446900
H	–0.15607800	–0.88833200	–3.16699500
H	–0.89473600	–2.31625900	–2.41651000

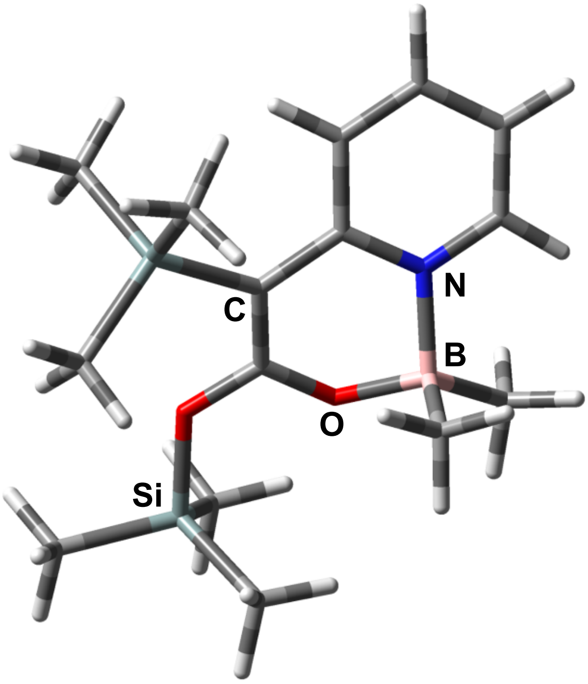

Figure S109. Optimized structure **P**$_{\mathbf{C}}$ [M06-2X/6–311+G(d,p)] (PCM, toluene).

Table S40. Cartesian coordinates of the optimized structure **P**$_{\mathbf{C}}$ [M06-2X/6–311+G(d,p)] (PCM, toluene).

Atomic symbol	x	y	z
N	1.96822400	–1.35840300	–0.04775400
C	3.13157400	–2.01373500	–0.21742300
C	4.30217300	–1.37672400	–0.54999500
H	5.21525400	–1.94268200	–0.66688200
C	0.57945700	–1.97872600	2.07175600
C	1.88651100	–0.00868000	–0.21593200
C	0.64500000	–3.67746100	–0.04858400
C	4.25677800	0.00966100	–0.73783600
H	5.15022900	0.55382700	–1.02100800
C	3.06674400	0.67899000	–0.58017000
H	3.01220700	1.74209200	–0.76620000
B	0.66553900	–2.15929700	0.47126900
C	0.61852400	0.66493000	–0.07643000
C	–0.50016000	–0.13648300	–0.19320300
O	–0.48124200	–1.43685200	–0.21843000

O	–1.70261100	0.40057500	–0.34357700
H	–0.31680800	–2.46976700	2.46539400
H	1.44196600	–2.44210200	2.56331100
H	0.54800800	–0.92925600	2.38602000
H	0.86202900	–3.76838000	–1.11877900
H	1.32927800	–4.33546100	0.49614100
H	–0.36094300	–4.07982300	0.11180100
Si	–3.18342700	–0.46868000	–0.27071200
C	–3.33399300	–1.55071300	–1.77961200
H	–4.32963600	–1.99986300	–1.83084400
H	–3.17957100	–0.97009600	–2.69235600
H	–2.59290400	–2.35166100	–1.75089000
C	–4.42882700	0.91733800	–0.27979400
H	–5.44864300	0.52451200	–0.29718800
H	–4.32158300	1.54175000	0.61058400
H	–4.29654600	1.55272000	–1.15902000
C	–3.24778600	–1.44406600	1.31778400
H	–2.93141700	–0.82860300	2.16444100
H	–4.26951600	–1.78227800	1.51200000
H	–2.59635800	–2.31811900	1.26729100
H	3.08663000	–3.08388800	–0.06545100
Si	0.45456300	2.51678100	0.19411600
C	1.78870600	3.08709000	1.40225700
H	2.80184300	3.07533000	0.99770700
H	1.57026400	4.11416400	1.70986100
H	1.77994600	2.46247800	2.30011700
C	0.61255900	3.47881700	–1.41735700
H	0.51985300	4.55361900	–1.23615300
H	1.56982600	3.30399400	–1.91518200
H	–0.18086100	3.18275900	–2.10881800
C	–1.19523400	2.93767700	0.98996600
H	–2.01594400	2.86907200	0.27554400
H	–1.41455300	2.26183300	1.82105500
H	–1.15456500	3.95844400	1.38127900

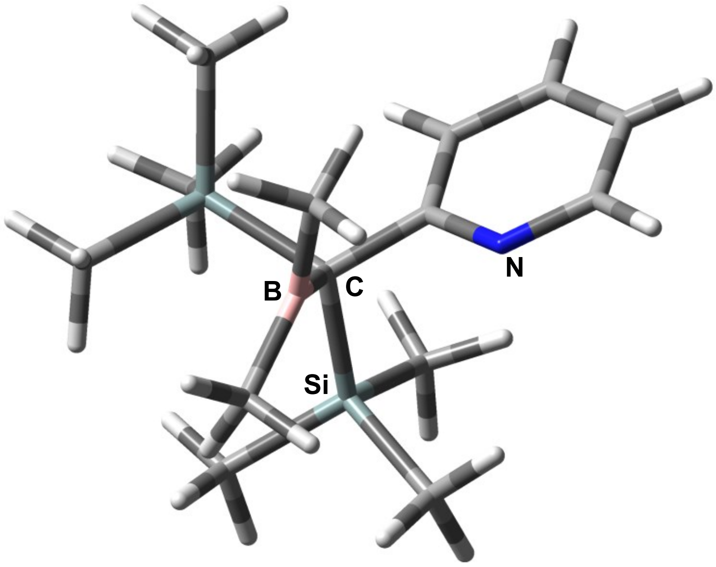

Figure S110. Optimized structure **I'c** [M06-2X/6–311+G(d,p)] (PCM, toluene).

Table S41. Cartesian coordinates of the optimized structure **I'c** [M06-2X/6–311+G(d,p)] (PCM, toluene).

Atomic symbol	x	y	z
Si	1.29583100	–1.40415400	–0.49074400
N	–2.04965300	0.41218700	0.98449500
C	–3.37463800	0.35623300	0.85620700
C	–4.02238100	–0.27966300	–0.19548100
H	–5.10317100	–0.29235100	–0.25025200
C	–0.16960000	–1.24099400	2.70252200
H	0.59663400	–1.94186700	3.06081300
C	–1.27484000	–0.18093600	0.05774900
C	1.60399700	0.75077700	2.56789300
H	1.03631800	1.53133900	3.09448000
C	–3.23288000	–0.88596900	–1.16503400
H	–3.68594700	–1.39004400	–2.01119100
C	–1.85302300	–0.83498300	–1.03959300
H	–1.22754800	–1.29175400	–1.79556600
B	0.53285500	–0.13385800	1.82042100
C	0.21213100	–0.01433000	0.26842800
C	1.53266900	–1.27428400	–2.36069200
H	1.87844900	–0.29346100	–2.69007100
H	2.29106300	–2.00799300	–2.65239300
H	0.62391200	–1.51169700	–2.91864900
C	0.56307800	–3.10287900	–0.13662900
H	1.15238600	–3.86179800	–0.66037400
H	0.59656800	–3.33219700	0.93064900
H	–0.47347000	–3.19582700	–0.46670800
C	2.99463900	–1.35341400	0.32218300

H	3.48835800	–0.38682900	0.20410100
H	2.93050400	–1.56699300	1.39313000
H	3.63389900	–2.11775400	–0.12929700
H	–0.59289400	–0.78392000	3.60384900
H	–0.95677700	–1.81189100	2.20761500
H	2.33831800	1.25927300	1.94195200
H	2.13140900	0.18878300	3.34428900
H	–3.94755500	0.85005700	1.63594600
Si	0.63041000	1.70102100	–0.48785400
C	–0.30928500	1.88791700	–2.11189100
H	–1.38484000	1.96166800	–1.92930800
H	0.01053900	2.80502800	–2.61540500
H	–0.14421400	1.05113400	–2.79519500
C	2.46469800	1.98187800	–0.84650200
H	3.07352400	2.00606700	0.05918400
H	2.90093300	1.24917700	–1.52760200
H	2.55384900	2.96415400	–1.32213800
C	0.06064100	3.09072600	0.64532400
H	0.75596500	3.25508500	1.47085800
H	–0.00709600	4.02144100	0.07389600
H	–0.92441300	2.87054200	1.06328300

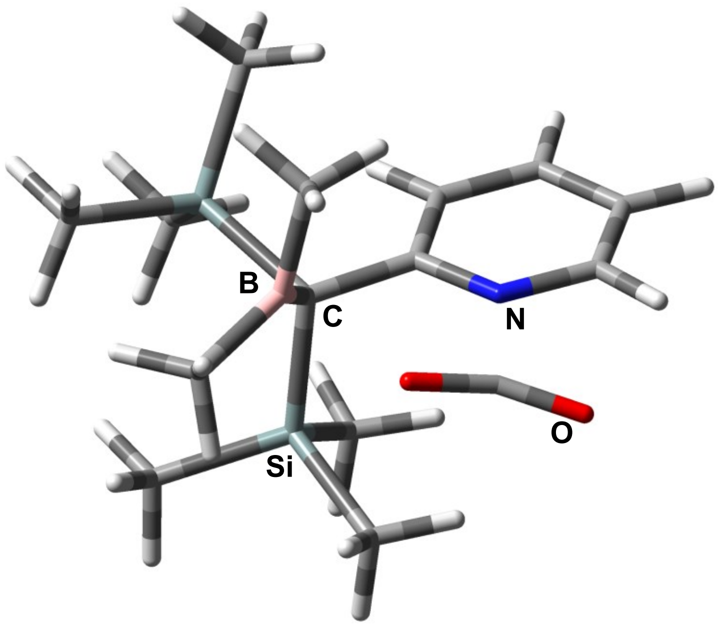

Figure S111. Optimized structure **TS'$_{C}$** [M06-2X/6–311+G(d,p)] (PCM, toluene).

Table S42. Cartesian coordinates of the optimized structure **TS'$_{C}$** [M06-2X/6–311+G(d,p)] (PCM, toluene).

Atomic symbol	x	y	z
Si	2.06371400	0.86098900	–0.48414500
N	–2.01735100	0.40707700	–0.20723600
C	–3.07645100	1.20534900	–0.03289500

C	–2.97059500	2.53335600	0.32821900
H	–3.85814700	3.13830300	0.45296300
C	–0.00453900	–0.43487800	–2.83480800
H	–0.40939000	–1.17264400	–3.53185500
C	–0.75656500	0.86407700	–0.02975800
C	0.97948400	–2.52830800	–1.41816900
H	0.65283800	–3.09314400	–2.29557900
C	–1.69198600	3.04005100	0.52785100
H	–1.54415100	4.07244700	0.82263900
C	–0.60176300	2.20896600	0.35361700
H	0.38722100	2.60578900	0.52635700
B	0.31037900	–1.08657900	–1.42028200
C	0.40676700	–0.09169200	–0.14795000
C	–2.59454100	–1.41599200	–0.72978500
C	2.85322700	1.67063300	1.03460100
H	3.13823000	0.95644200	1.80898100
H	3.77193300	2.15247600	0.68319800
H	2.24330900	2.44444800	1.50464200
C	1.77425500	2.23841300	–1.75517300
H	1.93571700	3.22408600	–1.31045400
H	2.48199700	2.12935200	–2.58152800
H	0.76765300	2.22148700	–2.17512700
C	3.40663700	–0.26215600	–1.17677400
H	3.63252500	–1.11286300	–0.53135400
H	3.16536700	–0.63681900	–2.17349600
H	4.31407000	0.34442700	–1.26122900
H	–0.68980000	0.41722500	–2.79510100
H	0.93347200	–0.06713100	–3.27341300
H	0.72099600	–3.12028700	–0.53723400
H	2.07014400	–2.47593700	–1.45880600
O	–1.62306400	–1.93663300	–1.17036900
O	–3.73538000	–1.43635500	–0.45147900
H	–4.04008300	0.73567900	–0.18721500
Si	0.49795400	–0.99398800	1.55026700
C	0.26485900	0.25562900	2.94556000
H	0.39139700	–0.25424600	3.90549200
H	0.97187100	1.08608300	2.91273700
H	–0.74665500	0.67184000	2.92426100
C	–0.89184300	–2.24233900	1.78920300
H	–0.88957300	–3.04793700	1.05455900

H	–0.77681100	–2.68420200	2.78430100
H	–1.87148400	–1.75599700	1.76514600
C	2.13811200	–1.88903200	1.80236400
H	2.09472400	–2.39943000	2.76969100
H	2.31780200	–2.64485400	1.03641400
H	2.99824300	–1.21781000	1.82550600

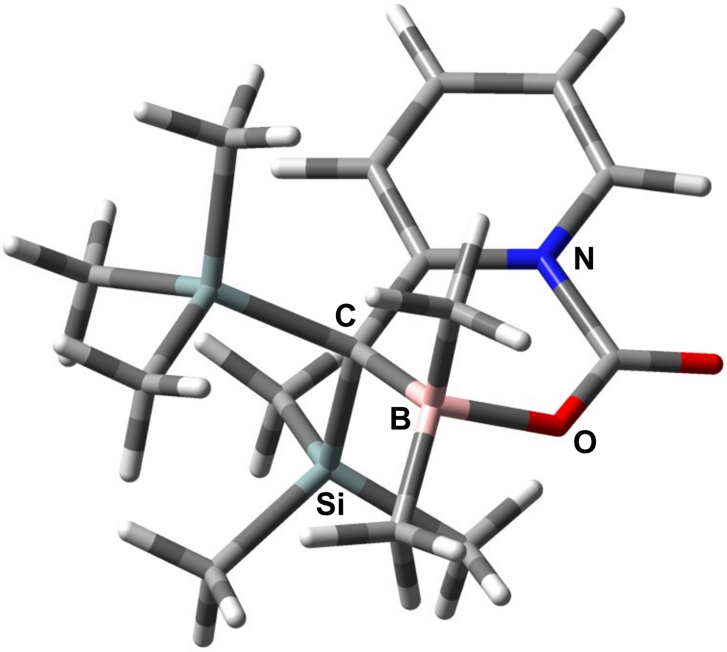

Figure S112. Optimized structure **P'$_C$** [M06-2X/6–311+G(d,p)] (PCM, toluene).

Table S43. Cartesian coordinates of the optimized structure **P'$_C$** [M06-2X/6–311+G(d,p)] (PCM, toluene).

Atomic symbol	x	y	z
Si	–1.86797800	1.05394800	0.59615700
N	2.08698900	0.19083100	0.23341700
C	3.23803000	0.90520900	0.09823000
C	3.24019200	2.18442500	–0.37451200
H	4.17105000	2.72684900	–0.45496100
C	0.08763700	–0.50891900	2.81302500
H	–0.85062100	–0.15734200	3.25367900
C	0.85019200	0.70306700	–0.07310700
C	–1.09677600	–2.49556200	1.42497200
H	–0.79916000	–3.10804400	2.28370100
C	2.01524100	2.73462400	–0.77251100
H	1.97235700	3.73219600	–1.19356400
C	0.86310400	2.00234600	–0.63031000
H	–0.07879900	2.41711100	–0.95662100
B	–0.12571600	–1.20698500	1.36310400
C	–0.37569600	–0.08414200	0.13129400
C	2.30449000	–1.26263800	0.63809800

O	1.27199800	–1.86763600	1.02114200
O	3.42703700	–1.67151900	0.51816000
C	–2.71345300	1.94078600	–0.85357300
H	–3.09795400	1.26222700	–1.61657600
H	–3.57292300	2.46904500	–0.42740900
H	–2.09951400	2.69258100	–1.35452900
C	–1.34447700	2.43551100	1.77108200
H	–2.24239000	3.00372900	2.03450900
H	–0.90806800	2.05329700	2.69423100
H	–0.63506900	3.13554600	1.32495400
C	–3.25320500	0.09489400	1.42970500
H	–3.64815900	–0.70377200	0.79918900
H	–2.94906700	–0.33946800	2.38244200
H	–4.06563000	0.80278200	1.62235400
H	0.50236900	–1.24756200	3.50779000
H	0.77547100	0.34696800	2.79926100
H	–1.01150700	–3.14041200	0.54465200
H	–2.15402900	–2.26000900	1.55574500
H	4.13312900	0.37066500	0.37861400
Si	–0.71128100	–0.95785900	–1.55863000
C	–0.50650900	0.25186200	–2.99541100
H	–0.77822100	–0.26628000	–3.92040800
H	–1.13567300	1.13997000	–2.91866300
H	0.53247700	0.57681600	–3.09880600
C	0.54843600	–2.31651500	–1.88904000
H	1.57092000	–1.92591300	–1.91493900
H	0.51621900	–3.12121600	–1.15430500
H	0.34332000	–2.73784300	–2.87844500
C	–2.44787200	–1.67657100	–1.64077700
H	–3.22653600	–0.91478300	–1.57468800
H	–2.55679100	–2.17436700	–2.60960700
H	–2.62622100	–2.41592900	–0.85982000

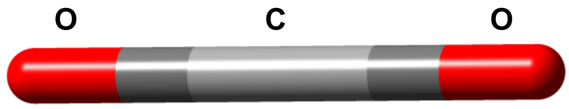

Figure S113. Optimized structure **CO_2** [M06-2X/6–311+G(d,p)] (PCM, toluene).

Table S44. Cartesian coordinates of the optimized structure **CO_2** [M06-2X/6–311+G(d,p)] (PCM, toluene).

Atomic symbol	x	y	z
C	0.00000000	0.00000000	–0.00000600
O	0.00000000	0.00000000	–1.15488900

O	0.00000000	0.00000000	1.15489400

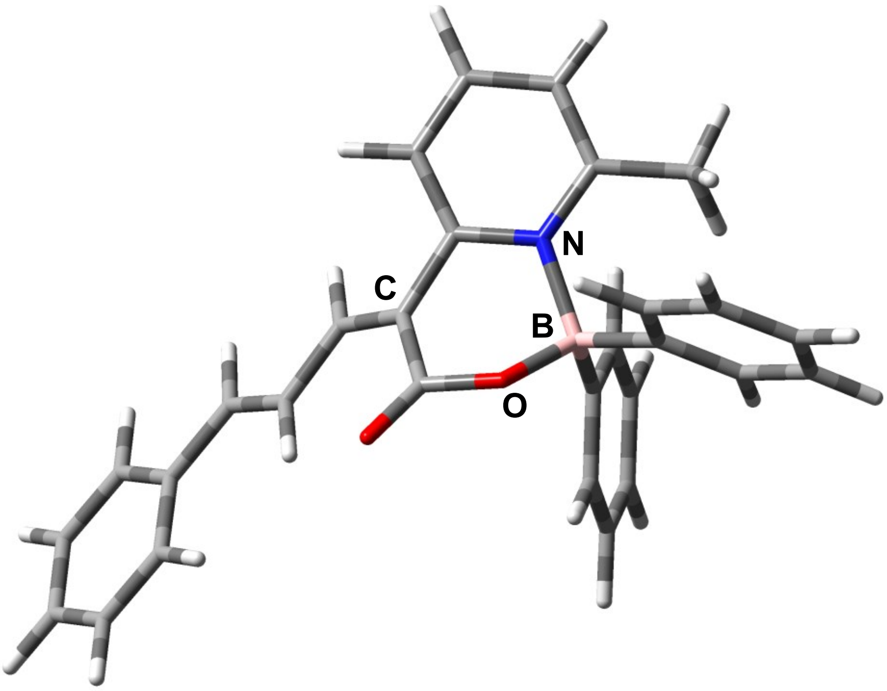

Figure S114. Optimized structure ***Z*-11** [M06-2X/6–311+G(d,p)].

Table S45. Cartesian coordinates of the optimized structure ***Z*-11** [M06-2X/6–311+G(d,p)].

Atomic symbol	x	y	z
O	–1.11474200	–0.33130700	–1.54458500
O	0.91914000	–0.27250200	–2.42877600
N	–1.87112800	1.32456600	0.16363000
C	–4.10833000	–1.73295900	–0.06183400
H	–3.66559000	–2.20651900	0.81004700
C	–3.39087500	–0.74509400	–0.73967600
C	0.12860900	0.09495000	–1.59647400
C	–1.24275000	–1.10848500	0.90791900
C	–0.67293800	1.93205400	–0.04230900
C	0.44951200	1.13434200	–0.56525800
C	–2.87726600	1.99770300	0.77232900
C	–5.38298100	–2.11569100	–0.47345900
H	–5.92024800	–2.88521900	0.06976200
C	–3.98135400	–0.17811300	–1.87565700
H	–3.42740800	0.56258900	–2.44576200
C	1.69711000	1.33747200	–0.08649700
H	1.81843200	2.06740000	0.71216900
C	–4.14599800	1.30491300	1.18028800
H	–4.79683600	1.11267400	0.32595200
H	–4.67261800	1.93882200	1.89307400
H	–3.93410000	0.34334900	1.64707900

C	–0.49804100	3.28547800	0.24172800
H	0.44457300	3.75490800	–0.00229400
C	2.89783600	0.63448200	–0.47224200
H	2.81433000	–0.09663900	–1.26443700
C	–1.19833200	–0.68676300	2.24063200
H	–1.59506400	0.28707600	2.51808400
C	–0.69492200	–2.36489800	0.61382500
H	–0.71020500	–2.71776000	–0.41338600
C	–5.96564900	–1.51376500	–1.58384900
H	–6.95861800	–1.80658200	–1.90530400
C	–2.72306600	3.34412000	1.08970200
H	–3.54550900	3.86057300	1.56606500
C	–5.25679000	–0.54530300	–2.29156400
H	–5.69633200	–0.08659600	–3.17034500
C	5.35586700	0.25290900	–0.13789700
C	–1.53788200	4.00109900	0.80291400
H	–1.42434500	5.05564400	1.02488900
C	4.06770200	0.89047000	0.14104600
H	4.08390100	1.63948800	0.93165900
C	–0.10766200	–2.72390700	2.92508500
H	0.32885800	–3.34454100	3.69905500
C	–0.13381500	–3.16379400	1.60411600
H	0.28503400	–4.13022900	1.34679700
C	6.46182400	0.59571000	0.64886600
H	6.34014000	1.32649000	1.44161600
C	–0.64181800	–1.48058300	3.24170300
H	–0.62155900	–1.12623200	4.26641200
C	5.52912700	–0.69079700	–1.16053300
H	4.69233500	–0.97009500	–1.78940900
C	7.70374800	0.01255500	0.43028300
H	8.54765400	0.28881800	1.05114900
B	–1.91970900	–0.27333100	–0.29391700
C	7.86047800	–0.92383700	–0.58510100
H	8.82709200	–1.38142400	–0.75938200
C	6.76869500	–1.27121300	–1.37959900
H	6.88709700	–1.99916300	–2.17334800

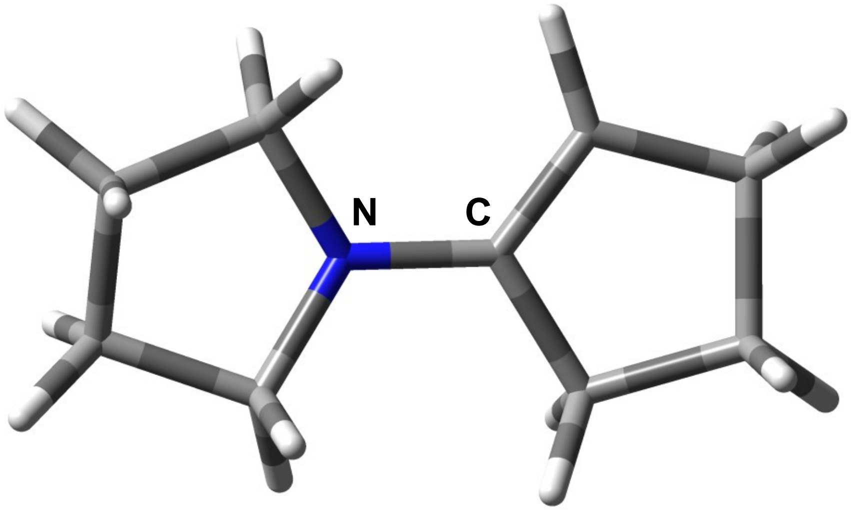

Figure S115. Optimized structure **Enamine** [M06-2X/6–311+G(d,p)].

Table S46. Cartesian coordinates of the optimized structure **Enamine** [M06-2X/6–311+G(d,p)].

Atomic symbol	x	y	z
C	1.47502400	1.16688000	–0.03946100
C	2.93960100	0.80211900	–0.07173200
C	0.69749000	0.06887000	–0.09337700
C	2.92446900	–0.69917500	0.28209400
H	3.54607200	1.38016600	0.63036600
H	3.36738400	0.96678100	–1.06938700
C	1.52963400	–1.19523800	–0.13759600
H	3.73262600	–1.25991100	–0.18973000
H	3.02705800	–0.80945100	1.36435100
H	1.52506400	–1.59779900	–1.15809200
H	1.14880400	–1.97936000	0.52168300
C	–1.46197400	1.22295500	–0.03807500
C	–1.44155400	–1.18189000	0.12427400
C	–2.87675400	0.71496600	0.28521200
H	–1.42914100	1.81977200	–0.95649400
H	–1.05721500	1.84134100	0.77336100
C	–2.86654700	–0.74045200	–0.19791400
H	–1.35135900	–1.45072400	1.18885200
H	–1.11373600	–2.03481400	–0.47325100
H	–3.03989400	0.73946300	1.36548300
H	–3.65527100	1.31662700	–0.18378700
H	–3.62167800	–1.35842100	0.28850600
H	–3.02378700	–0.78354600	–1.27855900
N	–0.66947200	0.00987100	–0.20443400
H	1.11505200	2.18656200	–0.07281700

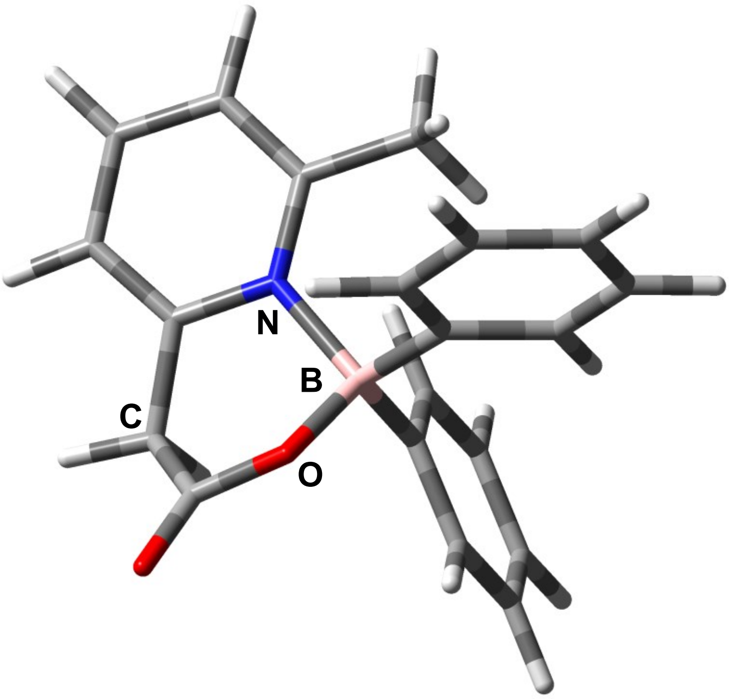

Figure S116. Optimized structure **12** [M06-2X/6–311+G(d,p)].

Table S47. Cartesian coordinates of the optimized structure **12** [M06-2X/6–311+G(d,p)].

Atomic symbol	x	y	z
O	0.22064600	–0.00176800	1.87934500
O	1.73770200	–0.21432200	3.49160500
N	0.58182200	–1.16241500	–0.31349400
C	–2.46541800	1.09029500	–0.29753800
H	–1.96310200	1.91382600	–0.79778700
C	–1.70363600	0.12820900	0.36867400
C	1.42357400	–0.27440000	2.33493800
C	0.59823500	1.47345700	–0.20776100
C	1.75563600	–1.57997100	0.21731400
C	2.40066000	–0.71042400	1.25219800
C	0.04635800	–1.81992400	–1.37096500
C	–3.85529700	1.00820900	–0.34713800
H	–4.42448300	1.76651300	–0.87322200
C	–2.38825800	–0.90305300	1.02382400
H	–1.82183800	–1.64093100	1.58523700
C	–1.13854500	–1.27344300	–2.11392300
H	–2.07524500	–1.49368000	–1.59881700
H	–1.16405600	–1.72813600	–3.10414800
H	–1.07076800	–0.19143100	–2.21949300
C	2.36638900	–2.75088400	–0.20706000
H	3.29016600	–3.06143700	0.26249300
C	1.21588100	1.51554700	–1.46182300
H	1.29163900	0.61123200	–2.06164200

C	0.54441000	2.66925100	0.52283200
H	0.07329900	2.66557700	1.50141200
C	–4.51310000	–0.04590500	0.27857500
H	–5.59414900	–0.11593400	0.23803900
C	0.63738200	–2.99521600	–1.82673900
H	0.18398800	–3.50910300	–2.66386400
C	–3.77464600	–1.00093700	0.97397700
H	–4.28196600	–1.81345800	1.48251000
C	1.78598700	–3.48346300	–1.22842800
H	2.23795600	–4.40576400	–1.57383800
C	1.68427000	3.86884900	–1.23108100
H	2.10078000	4.78912600	–1.62417300
C	1.07805600	3.85167900	0.02251800
H	1.02375000	4.76152100	0.60973000
C	1.75381200	2.69589200	–1.97337300
H	2.22766100	2.69787700	–2.94867300
B	–0.09697600	0.16847400	0.43352000
H	2.75167400	0.20509600	0.75954800
H	3.24954600	–1.21025000	1.71182500

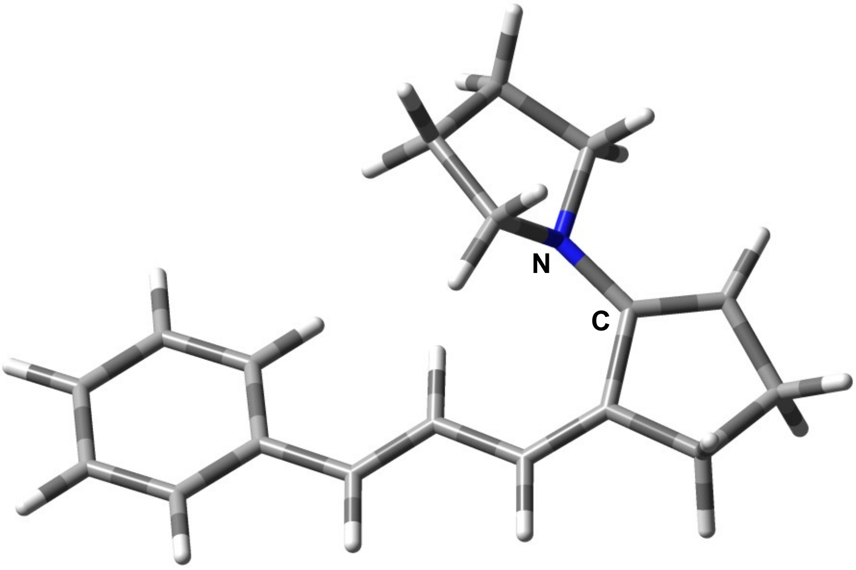

Figure S117. Optimized structure **13** [M06-2X/6–311+G(d,p)].

Table S48. Cartesian coordinates of the optimized structure **13** [M06-2X/6–311+G(d,p)].

Atomic symbol	x	y	z
C	0.82240500	–1.92469200	0.21444000
C	–0.37173700	–1.13003400	–0.01275100
H	–0.22158800	–0.11860400	–0.37505900
C	–2.85969000	–0.85464600	–0.03793300
C	–1.61447200	–1.59958000	0.18440200
H	–1.73398000	–2.61208500	0.56581900

C	–4.06186400	–1.36669200	0.46461900
H	–4.04907800	–2.30987500	1.00083000
C	–2.90138400	0.36103000	–0.73582100
H	–1.99289700	0.76777900	–1.16515500
C	–5.26078400	–0.68389100	0.29645300
H	–6.17787700	–1.09867900	0.69846400
C	–5.28372200	0.52690600	–0.38614100
H	–6.21681300	1.06033300	–0.52175600
C	–4.09725500	1.04342400	–0.90383400
H	–4.10817400	1.97917800	–1.45068400
H	0.65911100	–2.97934900	0.42875500
C	3.31084100	–2.39749300	0.23448300
C	2.62262300	–0.15089500	–0.16827200
C	4.36345700	–1.68092600	–0.63912900
H	3.09870400	–3.41993900	–0.07904700
H	3.66534900	–2.42357500	1.27047100
C	3.89100600	–0.25038700	–0.61739600
H	5.37917700	–1.79315400	–0.25430400
H	4.35717100	–2.08239600	–1.65933500
C	2.09783900	–1.50016200	0.14298600
C	1.29243100	1.29809700	1.27566400
C	2.44314900	2.25555400	–0.49518200
C	0.58076900	2.63139200	1.04304100
H	2.11009900	1.41110000	2.00533700
H	0.63335400	0.50192400	1.61990700
C	1.41747000	3.31883200	–0.06679800
H	2.62586000	2.22725300	–1.57098700
H	3.40736700	2.42562700	0.01229400
H	–0.43818500	2.44694700	0.69694800
H	0.52081100	3.22457700	1.95561300
H	0.78460400	3.60619700	–0.90681800
H	1.91602200	4.21964900	0.29193000
N	1.84871200	1.00201400	–0.05060700
H	4.49348400	0.57798000	–0.96595600

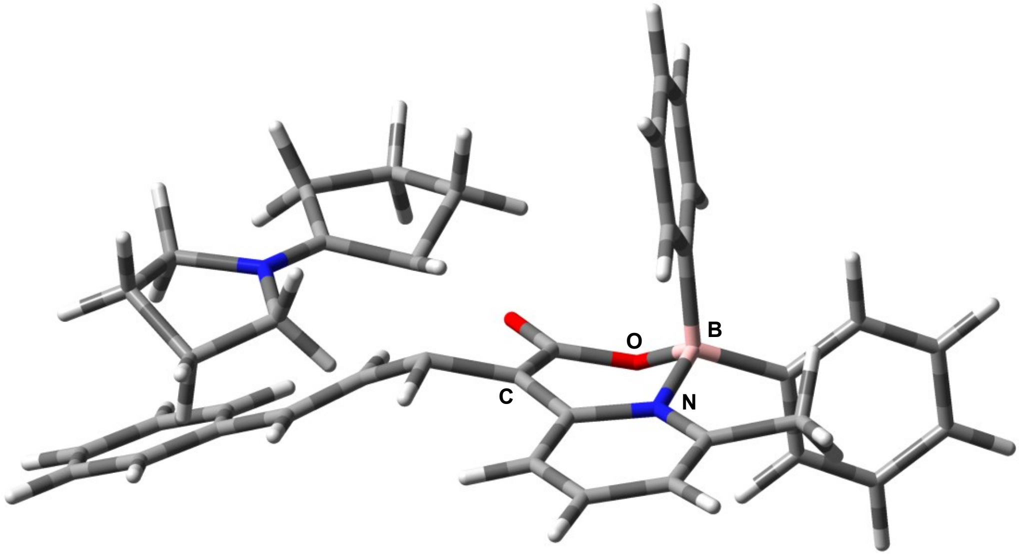

Figure S118. Optimized structure **14** [M06-2X/6–311+G(d,p)].

Table S49. Cartesian coordinates of the optimized structure **14** [M06-2X/6–311+G(d,p)].

Atomic symbol	x	y	z
O	–2.14459800	1.46691700	–0.61449500
O	–0.16510100	1.58636000	–1.60636100
N	–2.53426100	0.06418200	1.40152400
C	–5.64436700	0.65672500	–0.73489100
H	–5.57941500	–0.30991200	–1.22710200
C	–4.51242000	1.17955300	–0.10595300
C	–0.85000100	1.17808600	–0.67299300
C	–2.97101600	–0.91923100	–0.97311900
C	–1.15134700	0.05299400	1.52699900
C	–0.32551400	0.41792100	0.44390700
C	–3.33082300	–0.41566400	2.39682500
C	–6.86082200	1.33673800	–0.73227200
H	–7.72511200	0.90429700	–1.22486300
C	–4.63527100	2.43770300	0.49690100
H	–3.76006200	2.88331300	0.96084000
C	1.10799300	–0.00804500	0.41549400
C	–4.81122200	–0.56932000	2.17866300
H	–5.32177600	0.39245000	2.12873800
H	–5.22401600	–1.14876800	3.00392500
H	–5.01402600	–1.09281100	1.24324300
C	–0.61314700	–0.35427600	2.79047300
H	0.44626400	–0.25858400	2.97412100
C	2.12466200	1.00990400	–0.03505900
H	1.86019500	1.60685800	–0.89922300
C	–2.76349400	–2.20300600	–0.45678900
H	–2.69921900	–2.34662600	0.61978200

C	–3.02926400	–0.79798100	–2.36978700
H	–3.16993600	0.18981000	–2.80017600
C	–6.96810900	2.56915400	–0.09643500
H	–7.91362800	3.09970800	–0.08624700
C	–2.80882800	–0.84179200	3.60063200
H	–3.47457900	–1.21714500	4.36421800
C	–5.84532500	3.12322400	0.51519500
H	–5.91566200	4.09183400	0.99866400
C	4.42373200	2.00428600	0.16786600
C	–1.42437400	–0.77833500	3.80167900
H	–0.99809200	–1.05884100	4.75897000
C	3.30406600	1.14086300	0.58021800
H	3.50273000	0.53594600	1.46802400
C	–2.67518400	–3.16610500	–2.66772800
H	–2.56480100	–4.02724800	–3.31768300
C	–2.88389400	–1.89868300	–3.20844800
H	–2.93043000	–1.76977300	–4.28462000
C	5.64347300	1.90211700	0.84607500
H	5.72457400	1.21960900	1.68781400
C	–2.61707100	–3.31522000	–1.28616900
H	–2.46081700	–4.29805700	–0.85273800
C	4.33249200	2.90834800	–0.89911800
H	3.39456200	3.02355600	–1.42995100
C	6.74308500	2.66764000	0.47183400
H	7.67638300	2.57603200	1.01511400
B	–3.07263200	0.44350300	–0.08434500
C	6.63932800	3.55581600	–0.59180200
H	7.49061100	4.15827700	–0.88519600
C	5.42804500	3.67385100	–1.27164100
H	5.33598700	4.37295600	–2.09449600
C	1.23461300	–1.42517900	–0.40198200
C	0.58873800	–1.46681300	–1.79166500
C	2.66915200	–1.60913300	–0.70951400
H	0.82588200	–2.17256100	0.28197300
C	1.61509200	–0.78653500	–2.70830500
H	–0.38739000	–0.98809000	–1.80868100
H	0.44175200	–2.51315700	–2.07917800
C	2.97988100	–1.18695400	–2.11494200
H	1.52037400	–1.10023200	–3.74725500
H	1.46861100	0.29262100	–2.66435400

H	3.41467900	–2.04997400	–2.63556000
H	3.73323400	–0.39113600	–2.11922300
C	3.33856400	–2.48587700	1.49410900
C	5.03076100	–1.95319900	–0.18522000
C	4.70720900	–2.34808500	2.15555400
H	3.01739800	–3.53023100	1.44486700
H	2.55260700	–1.89883400	1.96491100
C	5.67591200	–2.65385700	1.00818300
H	5.31658500	–0.89739000	–0.24493800
H	5.23223600	–2.42984300	–1.14478400
H	4.84866800	–1.32208400	2.50531000
H	4.81929200	–3.01959900	3.00540800
H	6.68616300	–2.29111100	1.19152700
H	5.72426400	–3.73130800	0.83292000
N	3.58177500	–2.01033200	0.11396000
H	1.42224300	–0.29443900	1.41544700

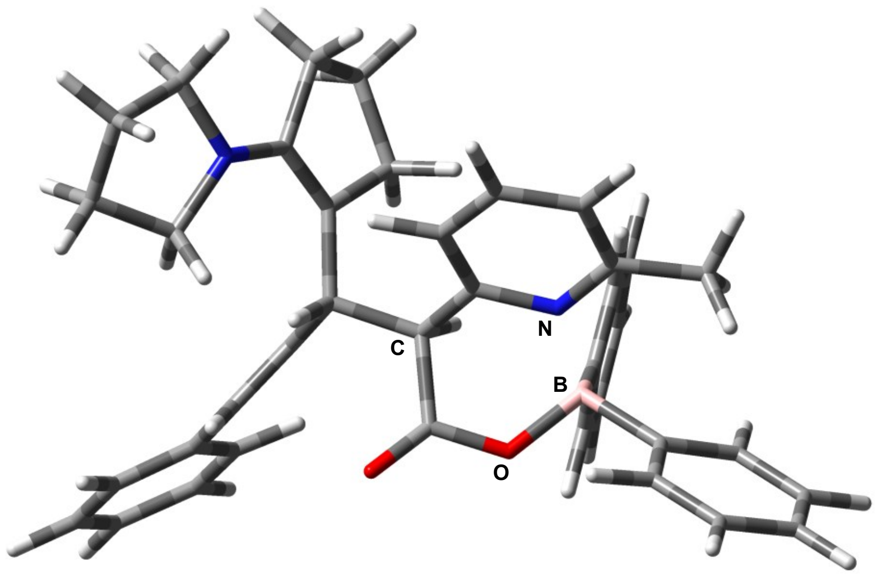

Figure S119. Optimized structure **15** [M06-2X/6–311+G(d,p)].

Table S50. Cartesian coordinates of the optimized structure **15** [M06-2X/6–311+G(d,p)].

Atomic symbol	x	y	z
O	1.80614000	0.92852300	–1.39202700
N	2.18375900	–1.18103000	–0.10810700
C	5.26466100	1.01689800	–0.26425500
H	5.12287400	1.42474300	0.73278300
C	4.17311600	0.46768500	–0.94059900
C	0.51049100	0.69687400	–1.45411000
C	2.51808500	1.16962100	1.09824400
C	0.84786300	–1.37005800	–0.26874400
C	0.01036000	–0.11440300	–0.25654700

C	3.00324900	–2.24247300	0.07879900
C	6.53466800	1.04263100	–0.83594000
H	7.36650000	1.47465100	–0.29069700
C	4.39066500	–0.02187800	–2.23441900
H	3.54905800	–0.41271800	–2.79950100
C	–1.51537900	–0.29990200	–0.20416000
C	4.43951300	–2.05719100	0.47905800
H	5.08163300	–1.89415800	–0.38868500
H	4.76967600	–2.95776500	0.99769500
H	4.55933300	–1.19982700	1.13941200
C	0.31926700	–2.64066800	–0.45317200
H	–0.73719600	–2.76862500	–0.64444600
C	–2.16962100	1.04962800	–0.01024700
H	–2.08984800	1.46248200	0.99380600
C	2.49078800	0.53685300	2.34505300
H	2.55516400	–0.54739700	2.40407200
C	2.41228500	2.56736500	1.08388300
H	2.42813900	3.08524700	0.12937500
C	6.73573000	0.51750500	–2.10822000
H	7.72328200	0.53256700	–2.55505300
C	2.49881800	–3.53337000	–0.04703900
H	3.17206200	–4.36775700	0.09904600
C	5.65558800	–0.01050500	–2.81214500
H	5.80005900	–0.40261300	–3.81283600
C	–3.49298500	3.02408300	–0.74752700
C	1.16644200	–3.73384800	–0.36425300
H	0.78031600	–4.73613600	–0.50895300
C	–2.83998400	1.71758100	–0.94721100
H	–2.92221300	1.28597300	–1.94242800
C	2.25882600	2.64597900	3.48961400
H	2.15828500	3.21266400	4.40806500
C	2.28363600	3.29904800	2.25950500
H	2.20221500	4.37959400	2.21825100
C	–4.56043800	3.39764700	–1.56966500
H	–4.88240800	2.72386600	–2.35686400
C	2.36335600	1.26045200	3.53001000
H	2.34159700	0.74173000	4.48208000
C	–3.07431900	3.91902100	0.24406500
H	–2.21460800	3.67388600	0.85731600
C	–5.21468600	4.61065700	–1.38609700

H	–6.04408500	4.87883300	–2.03016400
B	2.69349600	0.40459600	–0.30999600
C	–4.80321200	5.47998600	–0.38180700
H	–5.30710900	6.42858600	–0.23967800
C	–3.72622000	5.13027700	0.42900200
H	–3.38341700	5.81235800	1.19842900
C	–1.89635200	–1.20045200	0.95378400
C	–1.24859700	–0.99033400	2.30915300
C	–2.66064300	–2.31244200	0.96379600
C	–2.05859900	–1.90618000	3.23644400
H	–1.25557500	0.05211200	2.64323400
H	–0.19044700	–1.29545200	2.28415500
C	–2.59005100	–3.00737300	2.31033000
H	–1.47746700	–2.29564700	4.07331500
H	–2.90476000	–1.34739500	3.64285800
H	–1.88877000	–3.85057200	2.24223100
H	–3.54727300	–3.41097600	2.64505100
C	–3.85351500	–2.13459700	–1.21752400
C	–4.22304200	–4.05951200	0.18578900
C	–5.13442500	–2.86251100	–1.64136400
H	–3.13119000	–2.12194700	–2.04006500
H	–4.04019600	–1.10131300	–0.90955400
C	–4.89516400	–4.29570700	–1.16034600
H	–4.98356300	–3.80995700	0.93970500
H	–3.65511100	–4.92049200	0.54424700
H	–5.99543100	–2.43942500	–1.11732100
H	–5.31891500	–2.78625500	–2.71278500
H	–5.80982500	–4.88246600	–1.07416100
H	–4.20907700	–4.81515200	–1.83500000
N	–3.33539700	–2.92231400	–0.08281700
H	–1.83328500	–0.70769100	–1.16330500
H	0.31931800	0.44774000	0.63261900
O	–0.18549100	1.02357900	–2.37630300

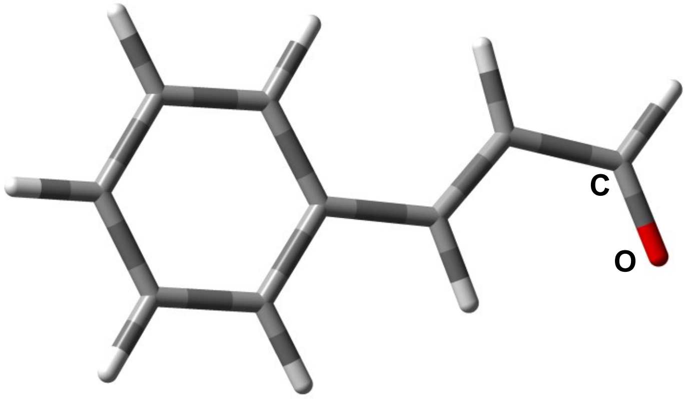

Figure S120. Optimized structure **Cinnamaldehyde** [M06-2X/6–31+G(d)].

Table S51. Cartesian coordinates of the optimized structure **Cinnamaldehyde** [M06-2X/6–31+G(d)].

Atomic symbol	x	y	z
O	3.85944400	–0.74498300	–0.00035100
C	1.05279700	–0.26174500	0.00002500
C	1.97490300	0.71595800	0.00008100
C	–0.40572200	–0.11299400	0.00010700
C	–1.04353200	1.13738400	0.00001300
C	–1.19837900	–1.26922900	0.00018300
C	–2.42965100	1.22393600	–0.00011800
H	–0.45278500	2.04862800	0.00001900
C	–2.58805900	–1.18434500	0.00002700
H	–0.71404900	–2.24286600	0.00036600
C	–3.20719700	0.06318100	–0.00013600
H	–2.90898000	2.19854300	–0.00019700
H	–3.18567800	–2.09094700	0.00004800
H	–4.29087800	0.13457900	–0.00032500
H	1.43027700	–1.28506000	0.00010700
H	1.71358200	1.77121200	0.00027900
C	3.41568300	0.38406100	0.00013100
H	4.10790400	1.24853100	0.00063300

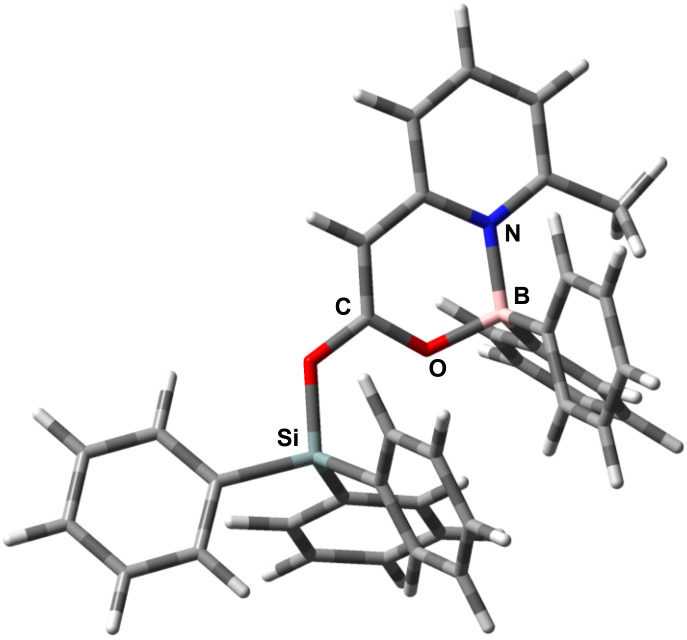

Figure S121. Optimized structure **5** [M06-2X/6–31+G(d)].

Table S52. Cartesian coordinates of the optimized structure **5** [M06-2X/6–31+G(d)].

Atomic symbol	x	y	z
Si	–2.28645300	–0.05220700	0.26449000
O	–1.29986600	–0.28981800	1.64605300
O	0.54762600	0.20115800	0.51988300
N	2.84327500	–0.05553000	1.56833700
C	0.02575400	–0.26235200	1.61196700
C	2.33482600	1.46444800	–0.58056600
C	2.18135100	–0.46429600	2.69558100
C	4.20111200	0.10475700	1.59180000
C	0.77077000	–0.65375600	2.69074600
H	0.27601200	–1.00612500	3.58471000
C	–3.98954300	–0.19207300	1.02186700
C	–1.04488000	1.88134600	–1.43999000
H	–0.33430800	1.09544400	–1.68153000
C	–1.90634200	–1.44384300	–0.91674400
C	2.21192100	–1.30914300	–0.66534400
C	2.90242800	–0.69051300	3.89026400
H	2.34840800	–1.00627900	4.76744900
C	2.08888100	2.65384200	0.12193000
H	1.70169000	2.59836400	1.13941900
C	4.92055700	–0.10587300	2.75452200
H	5.99599900	0.02566300	2.73582900
C	–2.08506000	1.62636800	–0.53248100
C	2.94404100	–2.41045100	–0.20126400
H	3.43598200	–2.36381500	0.77014800

C	4.26127200	–0.50627400	3.92253300
H	4.81990100	–0.67864500	4.83815300
C	–5.09959300	–0.48028800	0.21462700
H	–4.96003000	–0.67949300	–0.84730800
C	3.06823200	–3.58273800	–0.95035500
H	3.64313100	–4.41807300	–0.55820500
C	2.46062100	–3.67857600	–2.19946100
H	2.56038800	–4.58540000	–2.79052400
C	–4.20364800	0.04561900	2.38679700
C	–5.48815500	0.00469000	2.92810000
H	–5.63660800	0.19140700	3.98817700
C	–6.58024300	–0.27871300	2.10978000
H	–7.58156500	–0.31360000	2.53025300
C	2.80171000	1.58514300	–1.89488400
H	3.01745800	0.68773300	–2.47202200
C	–2.98026100	2.66648400	–0.24005300
H	–3.80610200	2.49272500	0.44755300
C	1.71443000	–2.60272300	–2.68118400
H	1.22067600	–2.66970500	–3.64717300
C	1.58653800	–1.44649400	–1.91672900
H	0.98072700	–0.62794700	–2.30393500
C	–1.78113600	4.16115000	–1.70912300
H	–1.65882200	5.14357000	–2.15752900
C	–0.88848500	3.13575400	–2.02203100
H	–0.05852600	3.31328600	–2.70109900
C	–6.38527900	–0.52324100	0.75060300
H	–7.23327000	–0.75197800	0.11112400
C	–2.82993500	3.92562100	–0.82189400
H	–3.53087000	4.72062000	–0.58252500
C	–2.18404400	–1.34049800	–2.28680600
H	–2.55771300	–0.40194900	–2.69414700
C	4.94719000	0.47855900	0.34080200
H	4.62359900	–0.11690900	–0.51634300
H	6.01293300	0.30515600	0.50442200
H	4.79618600	1.53207700	0.08944800
C	3.02619200	2.83314300	–2.48062200
H	3.39213200	2.89355600	–3.50265000
C	–1.39762700	–2.65678000	–0.42737500
H	–1.15707000	–2.75635700	0.62988600
C	2.31886900	3.90615100	–0.44429100

H	2.11893800	4.80805800	0.12857000
C	2.79142100	3.99938100	–1.75376500
H	2.96973600	4.97219200	–2.20448900
C	–1.98192300	–2.42508300	–3.14067400
H	–2.20470000	–2.33051400	–4.20016500
C	–1.48988200	–3.62630000	–2.63337400
H	–1.32458700	–4.46987900	–3.29866700
C	–1.18837900	–3.73960600	–1.27685900
H	–0.77857300	–4.66609200	–0.88483300
B	2.01367500	0.06262300	0.16792200
H	–3.35641000	0.26161200	3.03338300

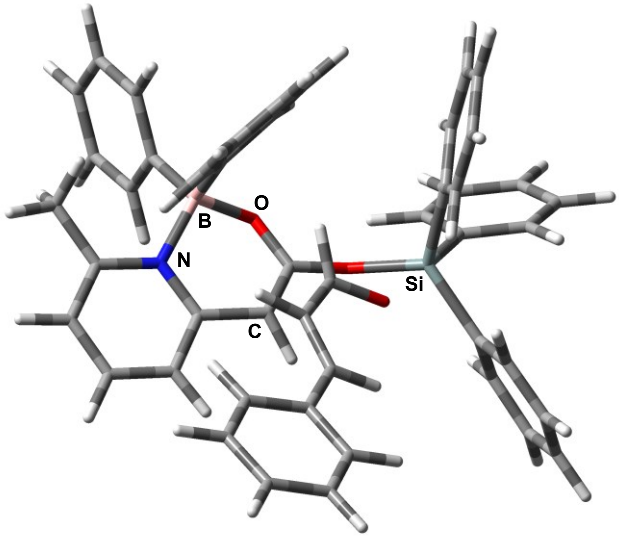

Figure S122. Optimized structure **TS$_{ZT}$** [M06-2X/6–31+G(d)].

Table S53. Cartesian coordinates of the optimized structure **TS$_{ZT}$** [M06-2X/6–31+G(d)].

Atomic symbol	x	y	z
O	1.29198300	–1.64070900	–0.79983000
N	3.10397300	0.11288700	–1.06353500
C	2.11038400	0.93131300	–1.51287800
C	3.58031800	–2.49758900	–0.52488100
C	4.40452300	0.50806900	–1.15307000
C	2.41257500	–0.76440300	1.29125300
C	2.41274800	2.15498100	–2.12745200
H	1.59377100	2.77826500	–2.47092800
C	0.71598200	0.57147900	–1.33169100
C	4.22975800	–3.20422100	0.49251600
H	4.11711200	–2.87583000	1.52469200
C	4.73008700	1.71263000	–1.76525500
H	5.77372000	1.99987200	–1.82314200
C	3.73942900	–2.95857800	–1.83972800

H	3.22732200	–2.44230600	–2.65162100
C	3.72924200	2.54306000	–2.26456400
H	3.98002600	3.48844500	–2.73690700
C	2.86973100	0.44146400	1.83952500
H	3.49751700	1.10911800	1.24786100
C	5.02929700	–4.31331700	0.21138900
H	5.52376900	–4.84516500	1.02001300
C	5.19364400	–4.73873100	–1.10558200
H	5.81815600	–5.59925700	–1.32942100
C	1.59610500	–1.56960800	2.10497100
H	1.20813100	–2.50613300	1.70670200
C	5.50680900	–0.30842300	–0.53709000
H	5.25362200	–0.61495000	0.48099000
H	6.41387700	0.29892700	–0.50561900
H	5.70969800	–1.21689400	–1.11001200
C	4.54069400	–4.05954000	–2.13538500
H	4.65276900	–4.39275000	–3.16386400
C	1.22923800	–1.17619300	3.38930500
H	0.57183800	–1.80657300	3.98049500
C	2.51152200	0.84593100	3.12785800
H	2.86969000	1.79534900	3.51820500
B	2.64264700	–1.22557800	–0.23991500
C	1.67881000	0.04155200	3.90175800
H	1.37521400	0.36067600	4.89494400
C	0.38030000	–0.78817400	–1.10643900
C	–0.26461600	1.15707500	0.36316000
O	–1.47885100	0.95782600	–0.00902600
C	–0.51769600	3.58680500	0.07122300
C	0.23758900	2.54364900	0.44469300
C	–0.11698000	5.00057400	0.09827100
C	1.21701600	5.40599100	0.26135600
C	–1.10034700	5.98726800	–0.05277800
C	1.54943900	6.75521800	0.29249800
H	2.00124400	4.65871300	0.35474900
C	–0.76903200	7.33959200	–0.02086300
H	–2.13573600	5.68447300	–0.19081000
C	0.55717000	7.72820300	0.15425000
H	2.58663200	7.05218600	0.41998600
H	–1.54654000	8.08919100	–0.13527100
H	0.81977100	8.78173800	0.17710600

H	–1.53020800	3.37095700	–0.27300600
H	1.24648700	2.65715100	0.83609100
H	0.00993300	1.14432500	–1.91999100
O	–0.83272200	–1.19982600	–1.15007700
Si	–2.33455100	–0.79866100	–0.22641400
H	0.18312600	0.45499700	1.08003000
C	–2.23434700	–1.02654800	1.66916400
C	–2.13478900	–2.31652000	2.21933900
C	–2.30878300	0.04720000	2.57322300
C	–2.11798400	–2.52603500	3.59807800
H	–2.08346600	–3.17928700	1.55994900
C	–2.30827400	–0.15382000	3.95330300
H	–2.37311300	1.06454300	2.19722000
C	–2.21487000	–1.44401300	4.47116200
H	–2.03879800	–3.53743700	3.98861900
H	–2.38115100	0.69935500	4.62286800
H	–2.21651000	–1.60480900	5.54632300
C	–3.71361200	0.22614600	–1.04420500
C	–4.23152000	1.39740100	–0.47012400
C	–4.25307800	–0.18730900	–2.26996800
C	–5.25664900	2.11562700	–1.08322000
H	–3.82908400	1.75446800	0.47504400
C	–5.25418500	0.54456600	–2.90952200
H	–3.89336600	–1.10510400	–2.73090400
C	–5.76506800	1.69451700	–2.31239100
H	–5.65726300	3.00606400	–0.60501500
H	–5.64418800	0.20665800	–3.86599200
H	–6.55806200	2.25719800	–2.79789800
C	–3.03400500	–2.54692700	–0.68020300
C	–4.35935000	–2.81610100	–0.29552800
C	–2.33100700	–3.58919600	–1.29814200
C	–4.95525200	–4.05585100	–0.51129100
H	–4.94583300	–2.03444900	0.18886100
C	–2.91899600	–4.83733800	–1.52700200
H	–1.29983600	–3.43196500	–1.60287700
C	–4.23242400	–5.07560400	–1.13331400
H	–5.98183800	–4.22776000	–0.19747100
H	–2.34504500	–5.62366400	–2.01138500
H	–4.69128000	–6.04534600	–1.30742900

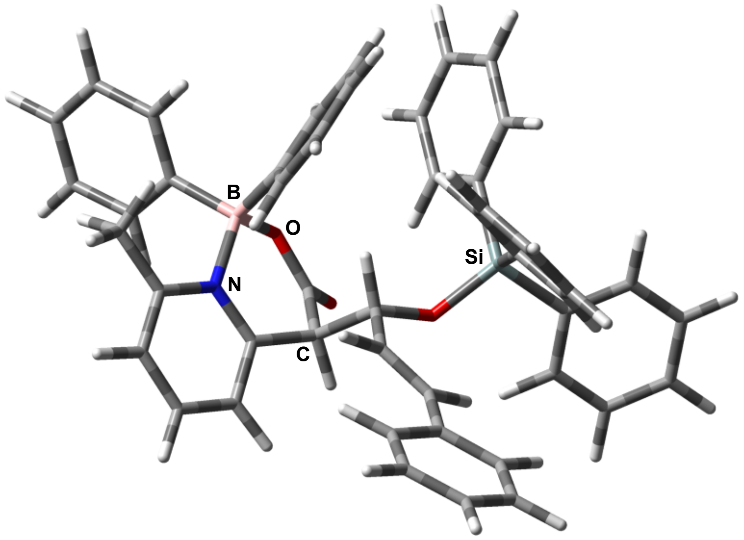

Figure S123. Optimized structure **16** [M06-2X/6–31+G(d)].

Table S54. Cartesian coordinates of the optimized structure **16** [M06-2X/6–31+G(d)].

Atomic symbol	x	y	z
O	2.46249900	–0.86450400	–1.25281700
N	2.71198400	1.62374700	–0.80550100
C	1.63314400	1.79657000	–1.60831600
C	4.64426500	–0.18311200	–0.37331400
C	3.47489300	2.69826200	–0.46171600
C	2.42114200	–0.08321300	1.21340500
C	1.32735300	3.03778700	–2.16026200
H	0.45677900	3.12147300	–2.80177800
C	0.71593700	0.63788100	–1.87651900
C	5.43799500	–0.56846100	0.71237600
H	4.98115300	–0.68643900	1.69370000
C	3.18370600	3.95633700	–0.98471200
H	3.80966000	4.79289400	–0.69407000
C	5.27602500	–0.05254800	–1.61885200
H	4.68166700	0.21496400	–2.49269000
C	2.11807200	4.13306400	–1.85537000
H	1.89854000	5.11143200	–2.27248800
C	2.01217200	0.96554500	2.04959600
H	2.03883500	1.99469100	1.68941800
C	6.80927900	–0.78799900	0.57259800
H	7.40141800	–1.08507800	1.43435200
C	7.42007400	–0.62306900	–0.66932100
H	8.48831800	–0.78759700	–0.78161100
C	2.32008800	–1.38645100	1.73064900
H	2.60242800	–2.22834400	1.10092800
C	4.62600800	2.57897500	0.49867500

H	4.41579700	1.88430900	1.31235700
H	4.82676900	3.56706700	0.92018500
H	5.52716400	2.22475900	–0.01174700
C	6.64570100	–0.25777200	–1.77170100
H	7.10845200	–0.14149500	–2.74836200
C	1.86645100	–1.62665800	3.02654000
H	1.79901600	–2.64788300	3.39161000
C	1.55735300	0.73863200	3.35061800
H	1.25302400	1.57813200	3.97107200
B	3.05077200	0.08423300	–0.26722500
C	1.49342200	–0.56090000	3.84776200
H	1.14151600	–0.74508300	4.85961600
C	1.48579600	–0.64975400	–2.10645600
C	–0.32086000	0.40961000	–0.73073000
O	–1.21263300	–0.56551100	–1.21047200
C	–2.28376800	1.96729500	–0.66823400
C	–1.01429300	1.69489000	–0.35617900
C	–3.04428800	3.16801200	–0.28238600
C	–2.44165900	4.31712700	0.24906300
C	–4.43606900	3.15968900	–0.43967500
C	–3.21033300	5.41262700	0.62700900
H	–1.36127000	4.35752800	0.36356600
C	–5.20867800	4.25517100	–0.06170200
H	–4.91392400	2.27398900	–0.85351100
C	–4.59807000	5.38601500	0.47615600
H	–2.72649500	6.29416600	1.03845100
H	–6.28726600	4.22503200	–0.18783800
H	–5.19576700	6.24362900	0.77070900
H	–2.84326100	1.21114600	–1.22017500
H	–0.41796600	2.38553100	0.24075800
H	0.12897400	0.84907700	–2.77386300
O	1.19711400	–1.41822800	–2.99433800
Si	–1.99183800	–1.65411800	–0.19257100
H	0.22365200	0.04188100	0.15164200
C	–2.63156400	–0.72625000	1.30408700
C	–1.73636000	–0.35574300	2.31963400
C	–3.93346400	–0.20798100	1.34667600
C	–2.12332900	0.51642700	3.33513900
H	–0.71656500	–0.74137400	2.31700200
C	–4.32811100	0.66081200	2.36342100

H	–4.64609700	–0.47449900	0.56660900
C	–3.42050200	1.02908700	3.35543200
H	–1.40912200	0.79694300	4.10551100
H	–5.33842700	1.06106900	2.37528800
H	–3.72419600	1.71447000	4.14216400
C	–3.39693600	–2.28868700	–1.24403100
C	–4.31838800	–3.20967500	–0.72299800
C	–3.57468900	–1.83866300	–2.55925300
C	–5.38874100	–3.66484000	–1.48943200
H	–4.20006000	–3.57805500	0.29580100
C	–4.64368600	–2.29318700	–3.33154400
H	–2.86235300	–1.13340300	–2.98066900
C	–5.55261700	–3.20409500	–2.79629900
H	–6.09344100	–4.37809000	–1.07095800
H	–4.76560200	–1.93775100	–4.35105900
H	–6.38650900	–3.55753800	–3.39667100
C	–0.78791000	–2.97332600	0.36095300
C	–0.89770700	–3.57120600	1.62588700
C	0.23022700	–3.41380800	–0.49923300
C	–0.01833900	–4.57652300	2.02341000
H	–1.67162600	–3.24076000	2.31710300
C	1.11921100	–4.41089500	–0.10006400
H	0.34186100	–2.97359600	–1.48785200
C	0.99647300	–4.99271200	1.16152000
H	–0.11938800	–5.02839400	3.00692600
H	1.91050100	–4.72616600	–0.77459900
H	1.68940000	–5.77015400	1.47295700

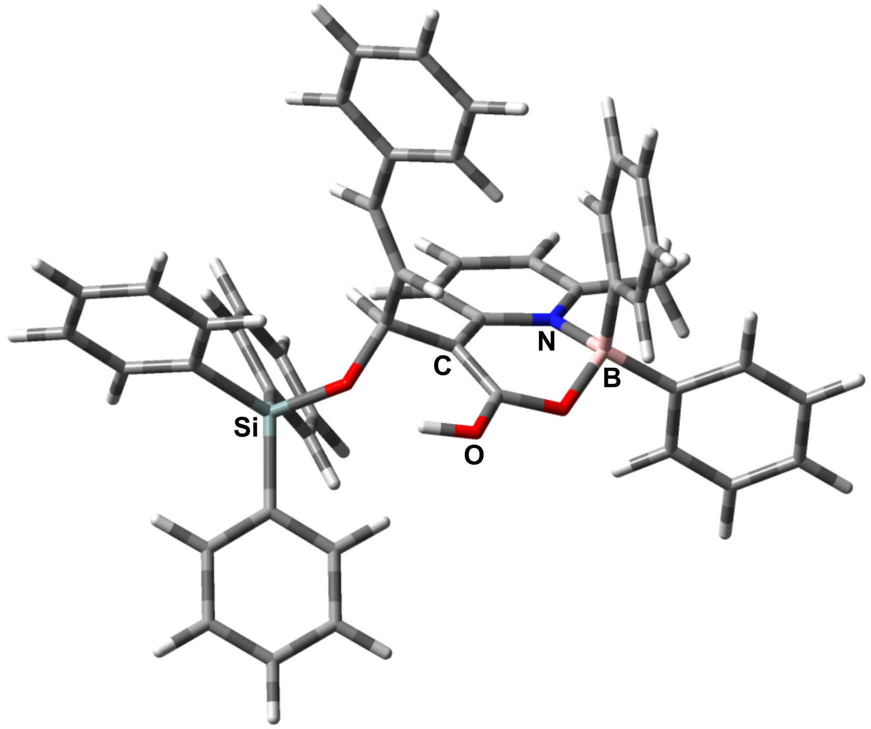

Figure S124. Optimized structure **17*z*** [M06-2X/6–31+G(d)].

Table S55. Cartesian coordinates of the optimized structure **17*z*** [M06-2X/6–31+G(d)].

Atomic symbol	x	y	z
O	–2.28804200	–1.09747800	–1.32094700
O	–0.40663000	–0.58235000	–2.33003100
N	–2.65230900	–0.85456500	1.13730600
C	–1.07969900	–0.65223200	–1.18549000
C	–1.29472700	–0.66552600	1.22412000
C	–4.60828300	–1.49714300	–0.64864700
C	–3.38875800	–1.09284800	2.26099100
C	–3.49952000	1.00713900	–0.49689400
C	–0.65298900	–0.81938200	2.47115500
H	0.42564700	–0.74060300	2.53160800
C	–0.54079200	–0.33031600	0.04318800
C	–5.86824900	–0.95598900	–0.92615800
H	–5.99518200	0.12558200	–0.92432400
C	–2.77194300	–1.24019600	3.49307900
H	–3.38498700	–1.42839900	4.36686900
C	–4.48548500	–2.89415700	–0.67667400
H	–3.51134700	–3.34472400	–0.49034100
C	–1.38518100	–1.12367900	3.59373500
H	–0.88446300	–1.26291900	4.54807400
C	–3.52989700	1.91330100	0.57162200
H	–3.37759200	1.55611900	1.59029500
C	–6.97120400	–1.77049000	–1.18715400

H	–7.93971400	–1.32238200	–1.39461600
C	–6.83180500	–3.15719600	–1.17963800
H	–7.68894900	–3.79517600	–1.37826900
C	–3.67022900	1.53643900	–1.78713900
H	–3.64193600	0.86107100	–2.64116700
C	–4.89275500	–1.11861900	2.20969800
H	–5.28436100	–0.26904200	1.64450400
H	–5.27308600	–1.06243500	3.23209800
H	–5.27575000	–2.02804200	1.74053500
C	–5.57950900	–3.71994900	–0.92839900
H	–5.45760300	–4.80024400	–0.93392000
C	–3.87964000	2.89858200	–1.99956400
H	–4.01196500	3.27646600	–3.01039900
C	–3.74100600	3.27954700	0.37388200
H	–3.75273500	3.95634400	1.22434900
B	–3.31008400	–0.59341400	–0.33842900
C	–3.92377700	3.77575200	–0.91485100
H	–4.08957700	4.83836700	–1.07359900
C	0.77132200	0.41661800	0.09912700
C	0.55979000	1.84606500	–0.31537700
H	0.06621800	1.99630900	–1.27665000
C	0.89855200	2.88578300	0.45327900
H	1.42484500	2.69658200	1.39128200
C	0.63322100	4.29853200	0.13028700
C	1.45118600	5.30073100	0.66710800
C	–0.43313900	4.67155100	–0.69972900
C	1.23020700	6.64143000	0.36142000
H	2.27404100	5.02136200	1.32196100
C	–0.65551400	6.01095900	–1.00273400
H	–1.11239800	3.91253900	–1.08003100
C	0.17689000	7.00069300	–0.47828000
H	1.87892700	7.40527700	0.78083900
H	–1.49231500	6.28332800	–1.64026200
H	–0.00252300	8.04601000	–0.71279100
H	1.17943800	0.41404400	1.11480800
O	1.72434900	–0.19157200	–0.79949900
Si	3.08909500	–1.01809100	–0.27413100
C	3.38924000	–2.30709800	–1.58920500
C	4.68256700	–2.66691800	–1.99291900
C	2.29929700	–2.96400900	–2.18272900

C	4.88367400	–3.65847700	–2.95225400
H	5.54556300	–2.16051900	–1.56414100
C	2.49523100	–3.95309900	–3.14474000
H	1.28117500	–2.69735500	–1.90123700
C	3.78959900	–4.30260600	–3.52794800
H	5.89279200	–3.92247900	–3.25587200
H	1.63924200	–4.44428600	–3.59856700
H	3.94451000	–5.07102700	–4.28006100
C	2.71011600	–1.79922100	1.39378500
C	3.07123700	–1.17324600	2.59742400
C	1.97484800	–2.99301800	1.46514400
C	2.70796400	–1.71725600	3.83027200
H	3.64551700	–0.24840400	2.57369800
C	1.60374400	–3.53827100	2.69281100
H	1.68853400	–3.50725300	0.54965800
C	1.97135100	–2.90053700	3.87743300
H	3.00111700	–1.21921700	4.75053600
H	1.02804500	–4.45887900	2.72538700
H	1.68689200	–3.32743500	4.83563200
C	4.48826100	0.20596200	–0.08914900
C	5.68766200	–0.13638600	0.55588600
C	4.35774800	1.50270000	–0.60712500
C	6.72771300	0.78318700	0.67249500
H	5.81052600	–1.13174500	0.98264700
C	5.39407100	2.42888600	–0.48725400
H	3.43357300	1.79277400	–1.10249400
C	6.57980200	2.06893000	0.15021500
H	7.64941000	0.50092500	1.17370800
H	5.27365500	3.42994500	–0.89195500
H	7.38771900	2.78930200	0.24366000
H	0.54378300	–0.43583900	–2.12743700

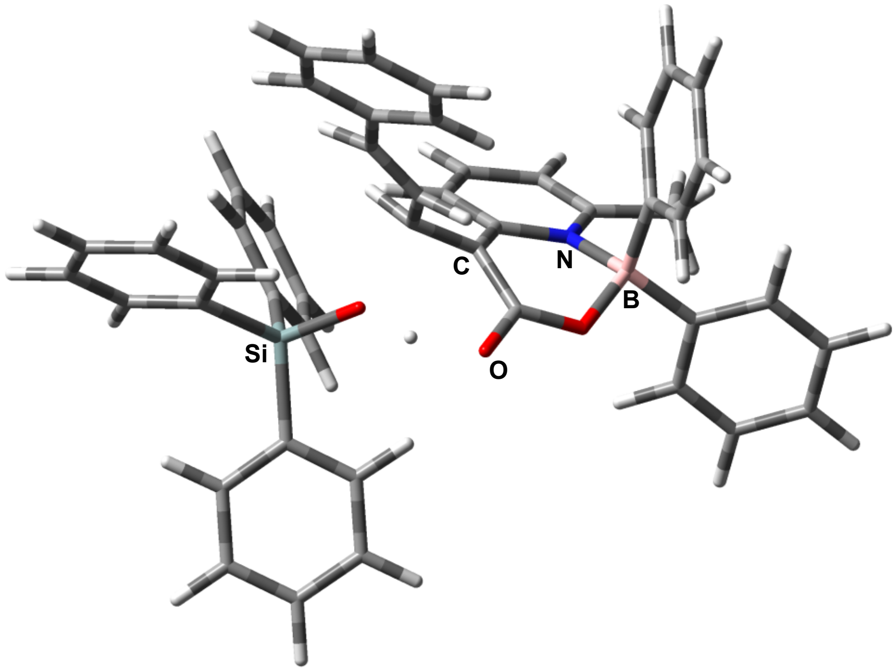

Figure S125. Optimized structure **TS***Z* [M06-2X/6–31+G(d)].

Table S56. Cartesian coordinates of the optimized structure **TS***Z* [M06-2X/6–31+G(d)].

Atomic symbol	x	y	z
O	–2.45837300	–0.18169800	–1.36349900
O	–0.36625100	0.05647600	–2.06295300
N	–2.92975500	–0.49481300	1.08267100
C	–1.21044400	0.06759200	–1.12173100
C	–1.57456800	–0.40271400	1.27813900
C	–4.82203700	–0.56647700	–0.88130000
C	–3.73859100	–0.94217500	2.08887800
C	–3.58304200	1.74982900	–0.10068400
C	–1.00758200	–0.87153800	2.48235200
H	0.06842600	–0.85653000	2.61012400
C	–0.77547200	0.19415200	0.24250800
C	–6.03548200	0.10471300	–1.06523300
H	–6.10371400	1.16027200	–0.80540800
C	–3.19834800	–1.40071700	3.27839700
H	–3.86665000	–1.75109100	4.05626900
C	–4.77106000	–1.91977700	–1.24757500
H	–3.83148800	–2.46135000	–1.14401100
C	–1.81405700	–1.38524400	3.46714100
H	–1.37523700	–1.76682500	4.38507400
C	–3.66247200	2.37861800	1.14821800
H	–3.63134800	1.78105700	2.05958700
C	–7.16465500	–0.55092200	–1.55867200
H	–8.09638400	–0.00605100	–1.68833100

C	–7.09840400	–1.90416600	–1.88609700
H	–7.97649300	–2.41937000	–2.26654400
C	–3.60768600	2.57699600	–1.23594700
H	–3.54660500	2.11857900	–2.22208900
C	–5.23706900	–0.87609900	1.96717700
H	–5.56251700	0.10517600	1.61294300
H	–5.67055700	–1.05160500	2.95430000
H	–5.63072900	–1.62070000	1.27155700
C	–5.89178400	–2.58905100	–1.73487500
H	–5.82649800	–3.64093500	–2.00198400
C	–3.70588500	3.96311700	–1.13086100
H	–3.72169800	4.57586100	–2.02882200
C	–3.76538500	3.76607500	1.26768800
H	–3.82399000	4.22327800	2.25228600
B	–3.49065700	0.15007300	–0.31893800
C	–3.78646000	4.56411800	0.12614500
H	–3.86447700	5.64450900	0.21418400
C	0.54086500	0.70525600	0.44549700
C	0.89627500	2.02434200	–0.09921400
H	0.32987000	2.36763400	–0.96177500
C	1.86856100	2.76123300	0.45950800
H	2.42443600	2.33295200	1.29651100
C	2.31041900	4.09141400	0.02207600
C	3.55988300	4.56364600	0.44659400
C	1.53136600	4.90503300	–0.81470300
C	4.03274400	5.80517600	0.02931400
H	4.16720800	3.94217800	1.10152800
C	2.00204500	6.14579400	–1.22794100
H	0.54505200	4.57314000	–1.12684300
C	3.25541800	6.59909900	–0.81185300
H	5.00543400	6.15394500	0.36397300
H	1.38580500	6.76670800	–1.87156400
H	3.61882800	7.56957900	–1.13665600
H	1.01566000	0.49502000	1.40395000
O	1.59435100	–0.28359000	–0.68842600
Si	2.66307700	–1.51241600	–0.29071900
C	2.55429500	–2.80649800	–1.63018700
C	3.66185200	–3.59121800	–1.98306300
C	1.33583500	–3.03615900	–2.28841700
C	3.55512700	–4.58340100	–2.95587600

H	4.62404000	–3.42030200	–1.50305300
C	1.22627900	–4.02527400	–3.26469500
H	0.46168200	–2.42969100	–2.05494100
C	2.33564800	–4.80174400	–3.59627900
H	4.42366300	–5.18004500	–3.22014900
H	0.27724900	–4.18268100	–3.76910600
H	2.25147000	–5.57109500	–4.35874300
C	2.16330100	–2.22747700	1.37385300
C	2.64323500	–1.67743600	2.57396300
C	1.25960300	–3.29795200	1.45303500
C	2.23016700	–2.17387100	3.81072800
H	3.35716200	–0.85503000	2.54359700
C	0.84331700	–3.79943600	2.68549800
H	0.87710700	–3.74865000	0.53891100
C	1.32916700	–3.23767900	3.86565700
H	2.61642300	–1.73824300	4.72823300
H	0.14036100	–4.62694100	2.72512800
H	1.01106000	–3.63211900	4.82728000
C	4.33833600	–0.69903900	–0.12165400
C	5.41572600	–1.34195800	0.50823400
C	4.53707600	0.59754800	–0.61950300
C	6.65499100	–0.71612700	0.62834800
H	5.28494800	–2.34050300	0.92459000
C	5.77385600	1.23118700	–0.49575800
H	3.71202800	1.11774100	–1.10170100
C	6.83390300	0.57347000	0.12645400
H	7.47777600	–1.22917400	1.11835600
H	5.90681000	2.23636400	–0.88698100
H	7.79853100	1.06411500	0.22333100
H	0.83519300	–0.20707100	–1.47763900

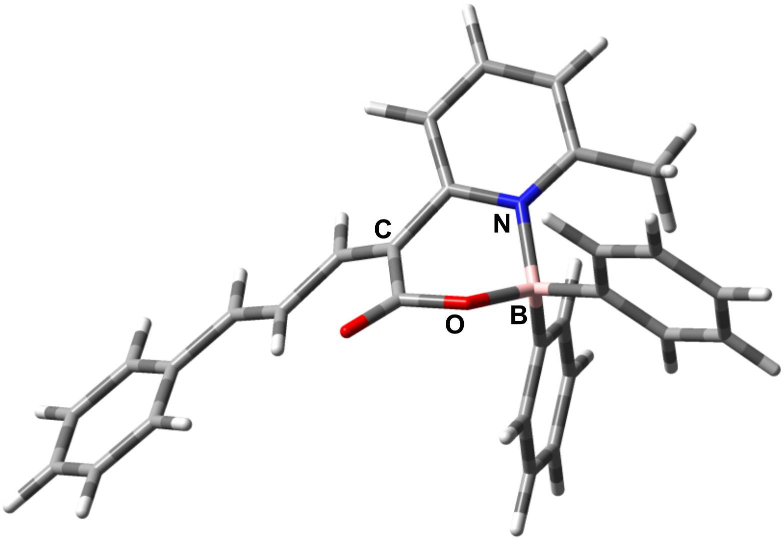

Figure S126. Optimized structure ***Z*-11** [M06-2X/6–31+G(d)].

Table S57. Cartesian coordinates of the optimized structure ***Z*-11** [M06-2X/6–31+G(d)].

Atomic symbol	x	y	z
O	–1.11610900	–0.33669100	–1.54068700
O	0.93243900	–0.30809900	–2.40800200
N	–1.86974400	1.32677200	0.16352300
C	–4.12478800	–1.72501200	–0.07644400
H	–3.68444700	–2.20502700	0.79643200
C	–3.40077900	–0.73430900	–0.74857300
C	0.13422500	0.07825900	–1.58182100
C	–1.26063500	–1.11233000	0.91190200
C	–0.66548700	1.92828500	–0.03922000
C	0.45546200	1.12437000	–0.55858700
C	–2.87403700	2.00557100	0.77223100
C	–5.40210100	–2.10368300	–0.49239500
H	–5.94266600	–2.87652700	0.04805700
C	–3.99131500	–0.15756800	–1.88266000
H	–3.43569800	0.59042400	–2.44697500
C	1.70865200	1.33201900	–0.08556700
H	1.83337800	2.06937400	0.70915300
C	–4.15011200	1.31953000	1.17466600
H	–4.80150300	1.13136500	0.31691600
H	–4.67789400	1.95813000	1.88672000
H	–3.94989300	0.35467600	1.64546900
C	–0.48469100	3.28358900	0.24527100
H	0.46229600	3.75064200	0.00163300
C	2.90989200	0.62726500	–0.47147300
H	2.82234500	–0.11425900	–1.25690200
C	–1.21483700	–0.69046400	2.24707500

H	–1.60546400	0.28958600	2.52296400
C	–0.72028800	–2.37526600	0.62042000
H	–0.73587000	–2.72971200	–0.40904100
C	–5.98272200	–1.49356800	–1.60290900
H	–6.97824600	–1.78302900	–1.92858700
C	–2.71361700	3.35314400	1.09273000
H	–3.53604600	3.87405400	1.56997700
C	–5.26927700	–0.51985600	–2.30431100
H	–5.70734000	–0.05224500	–3.18247800
C	5.37494500	0.25472700	–0.14214500
C	–1.52286000	4.00638100	0.80735700
H	–1.40367800	5.06255900	1.03044000
C	4.08573100	0.89327400	0.13466000
H	4.10418400	1.65387600	0.91782800
C	–0.13971600	–2.73910300	2.93736700
H	0.29128500	–3.36420000	3.71466900
C	–0.16643500	–3.18014300	1.61386800
H	0.24638300	–4.15220800	1.35650600
C	6.48720400	0.61426200	0.63283400
H	6.36857100	1.35956300	1.41606800
C	–0.66588100	–1.48868900	3.25236800
H	–0.64539900	–1.13209400	4.27913700
C	5.54461300	–0.70774300	–1.15139200
H	4.70225200	–1.00078100	–1.77070900
C	7.73178800	0.02985800	0.41575600
H	8.58055000	0.31969800	1.02815300
B	–1.92691100	–0.27234300	–0.29535300
C	7.88507300	–0.92535400	–0.58673800
H	8.85407600	–1.38427500	–0.75980600
C	6.78686500	–1.28983700	–1.36939500
H	6.90170500	–2.03260700	–2.15326100

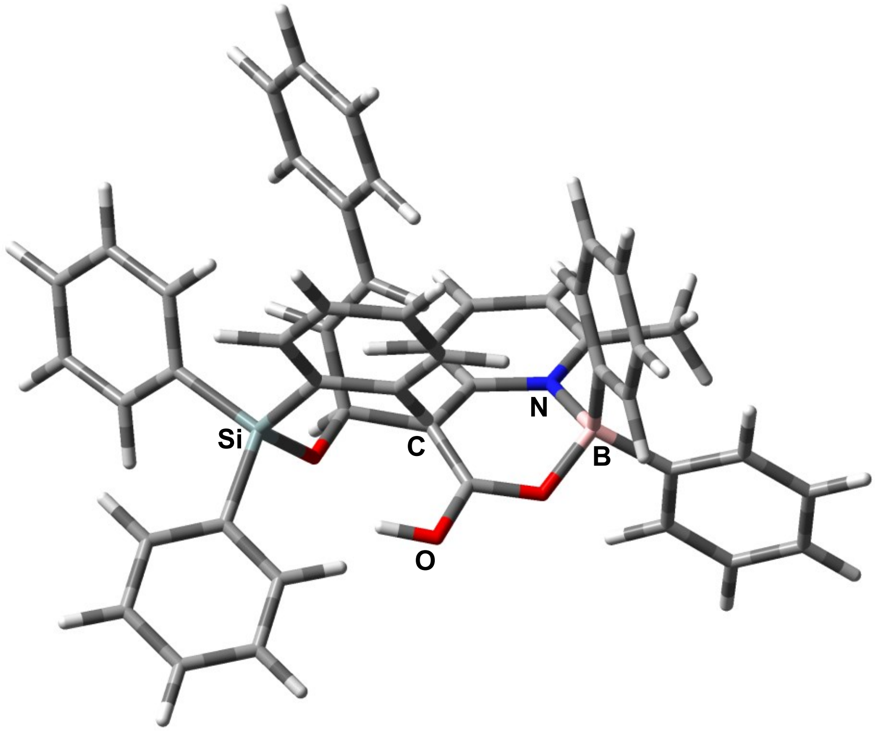

Figure S127. Optimized structure **17*E*** [M06-2X/6–31+G(d)].

Table S58. Cartesian coordinates of the optimized structure **17*E*** [M06-2X/6–31+G(d)].

Atomic symbol	x	y	z
O	–2.67977000	1.72265200	–0.24003900
O	–0.73258100	2.70207200	–0.21022000
N	–3.17463400	–0.41748000	–1.41450000
C	–1.43459900	1.64071500	–0.58450100
C	–1.90422000	–0.31219200	–1.91801100
C	–5.02383800	1.05176800	–0.11109900
C	–4.04909500	–1.33522000	–1.91992400
C	–3.06034100	–0.36705500	1.20469300
C	–1.54926100	–1.08673700	–3.04675500
H	–0.56020200	–0.98217400	–3.47336900
C	–0.94129900	0.57929400	–1.32200600
C	–5.97232400	0.80044500	0.88639400
H	–5.70518400	0.16909400	1.73222600
C	–3.71059000	–2.10440600	–3.02045400
H	–4.42592700	–2.82601200	–3.39730500
C	–5.41728800	1.87143200	–1.17951100
H	–4.69562900	2.10357600	–1.96192000
C	–2.45204300	–1.95911900	–3.60520400
H	–2.17945800	–2.54420300	–4.47881900
C	–2.62361400	–1.69884600	1.17323400
H	–2.53641200	–2.21970400	0.21894800
C	–7.26505800	1.32199900	0.81380400

H	–7.98160900	1.10822100	1.60293900
C	–7.63951100	2.11355600	–0.27083700
H	–8.64671600	2.51668100	–0.33397000
C	–3.13083000	0.24885600	2.46663200
H	–3.45425400	1.28723700	2.52723500
C	–5.37083700	–1.59163900	–1.24808100
H	–5.25810200	–1.65983500	–0.16322900
H	–5.76614700	–2.54030300	–1.61829200
H	–6.09756000	–0.80242000	–1.45602000
C	–6.70703200	2.39114500	–1.27078400
H	–6.98470200	3.01478800	–2.11673900
C	–2.81860300	–0.43818300	3.63822000
H	–2.90195900	0.06275800	4.60001500
C	–2.28829800	–2.39426200	2.33822100
H	–1.94770500	–3.42500300	2.27469300
B	–3.51038200	0.48359400	–0.09897400
C	–2.39635800	–1.76778500	3.57709700
H	–2.14880500	–2.30756600	4.48758000
C	0.51873300	0.42408000	–1.72588000
C	1.06481100	–0.96806600	–1.46818400
H	1.85333500	–1.30532900	–2.13964300
C	0.73461500	–1.68565000	–0.38996400
H	–0.02549400	–1.29294500	0.28689300
C	1.40233600	–2.92270700	0.04410000
C	1.44568700	–3.20379600	1.41649200
C	2.05618700	–3.78587300	–0.84549900
C	2.14934400	–4.30765800	1.89188800
H	0.94563400	–2.53041700	2.11103000
C	2.74919600	–4.89494600	–0.37075400
H	2.01639200	–3.58840900	–1.91396000
C	2.80362500	–5.15613700	0.99979600
H	2.18781500	–4.50265900	2.95998500
H	3.24642100	–5.56066700	–1.07110400
H	3.34799600	–6.02143100	1.36740700
H	0.18639300	2.57279100	–0.53846100
O	1.32802400	1.40293400	–1.04153900
H	0.64581000	0.65752200	–2.79353200
Si	2.74651200	1.16403700	–0.14859600
C	2.35272900	0.44347000	1.53308200
C	3.37271000	–0.07077700	2.34771400

C	1.05138000	0.49030000	2.05536400
C	3.10008300	–0.55179000	3.62745400
H	4.39930900	–0.09557900	1.98454400
C	0.77403500	0.02333700	3.33893200
H	0.23289400	0.89002500	1.45723100
C	1.79794700	–0.50675300	4.12344300
H	3.90353900	–0.95683700	4.23666800
H	–0.24311600	0.06694200	3.71511200
H	1.58192200	–0.87830700	5.12176900
C	3.37323600	2.91402900	0.08124100
C	4.67774600	3.30933700	–0.24382000
C	2.51811300	3.86721400	0.65872700
C	5.10971500	4.61668900	–0.01704000
H	5.37145600	2.59066800	–0.67522900
C	2.94065400	5.17500400	0.87960500
H	1.50892400	3.58721200	0.96066600
C	4.24016600	5.55204700	0.53888300
H	6.12569500	4.90299900	–0.27476900
H	2.26012100	5.89658800	1.32247900
H	4.57482200	6.57090900	0.71330800
C	3.95960000	0.12308300	–1.12722900
C	4.34816600	–1.16581100	–0.73188500
C	4.42566300	0.60521800	–2.36151200
C	5.18125800	–1.94262800	–1.53604700
H	3.97313200	–1.58909200	0.19836300
C	5.25612400	–0.16857000	–3.17101100
H	4.12863000	1.59711400	–2.70083500
C	5.63725600	–1.44460100	–2.75580600
H	5.46115600	–2.94169900	–1.21293800
H	5.60469200	0.22185000	–4.12311300
H	6.28424300	–2.05061700	–3.38409000

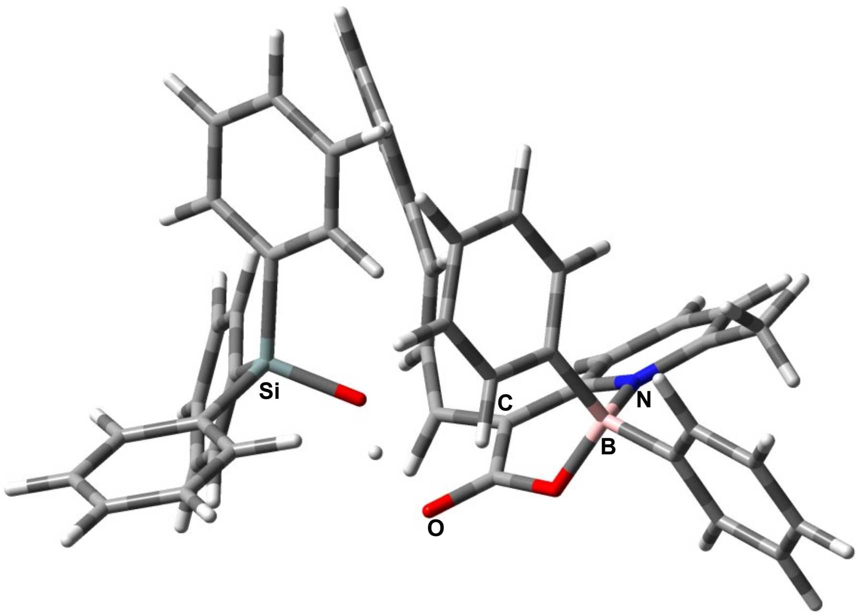

Figure S128. Optimized structure **TS*E*** [M06-2X/6–31+G(d)].

Table S59. Cartesian coordinates of the optimized structure **TS*E*** [M06-2X/6–31+G(d)].

Atomic symbol	x	y	z
O	–3.02173200	1.03915100	–1.42157500
O	–1.03519300	1.95497300	–1.83335400
N	–3.21233300	–1.34852900	–0.69518500
C	–1.73306700	0.92895100	–1.61228300
C	–1.98406700	–1.52331400	–1.28445200
C	–5.19472800	0.49741400	–0.43850800
C	–3.98738000	–2.42589500	–0.37376100
C	–2.92344900	0.55086200	1.08972900
C	–1.61283900	–2.81999000	–1.71123600
H	–0.69729000	–2.94288600	–2.27305100
C	–1.14511300	–0.37734700	–1.52583400
C	–5.96215100	0.93701000	0.64535500
H	–5.48596200	1.07046400	1.61588200
C	–3.59748300	–3.70769600	–0.72149500
H	–4.23020700	–4.54312200	–0.44535800
C	–5.84244100	0.35862000	–1.67487100
H	–5.26344300	0.04863100	–2.54393300
C	–2.41055400	–3.90148000	–1.43109900
H	–2.12022300	–4.89552000	–1.75944400
C	–2.64456400	–0.38943300	2.09101900
H	–2.82968400	–1.44790500	1.90812600
C	–7.32821100	1.19262300	0.51653000
H	–7.90149800	1.52853100	1.37703400
C	–7.95769700	1.01678300	–0.71465600

H	–9.02205700	1.21014200	–0.81897200
C	–2.62538300	1.89425300	1.37516200
H	–2.81490300	2.64617400	0.61049200
C	–5.25742900	–2.26198500	0.41921000
H	–5.13149200	–1.56426200	1.24940100
H	–5.53787500	–3.23845900	0.82141700
H	–6.07918700	–1.88730300	–0.19670000
C	–7.20697500	0.60380600	–1.81667100
H	–7.68528200	0.47896400	–2.78496900
C	–2.09586000	2.28259400	2.60519400
H	–1.87429800	3.32978800	2.79622800
C	–2.13020600	–0.01156100	3.33348600
H	–1.94454800	–0.76694600	4.09417700
B	–3.61349700	0.19828900	–0.33532200
C	–1.85723200	1.32945400	3.59624200
H	–1.45524500	1.63198600	4.56006700
C	0.27618100	–0.40296200	–1.78085600
C	1.15103100	–1.53858700	–1.42501100
H	1.86632600	–1.85425700	–2.18261000
C	1.20735100	–2.02638100	–0.17723000
H	0.53600900	–1.60575600	0.57380900
C	2.18448000	–3.01634300	0.29398200
C	2.51888200	–3.04110300	1.65436800
C	2.84388600	–3.89232800	–0.58097900
C	3.50745500	–3.90161200	2.12598100
H	2.01936900	–2.35732900	2.33731400
C	3.82298500	–4.75887000	–0.10796600
H	2.58409700	–3.89928900	–1.63658800
C	4.16262400	–4.76184300	1.24703900
H	3.76631100	–3.89497800	3.18103900
H	4.32099200	–5.43712400	–0.79517400
H	4.93003300	–5.43715900	1.61416900
H	0.17098500	1.62134800	–1.20417100
O	0.86488000	0.93374000	–0.75736000
H	0.59841700	0.11721000	–2.68452100
Si	2.43550900	1.27782100	–0.22426000
C	2.54811000	0.59900800	1.51331800
C	3.78583100	0.34536400	2.12255400
C	1.38108500	0.42399300	2.27453800
C	3.86220300	–0.09196500	3.44400600

H	4.70847200	0.49028200	1.56208200
C	1.45480800	–0.00252000	3.60030500
H	0.40337600	0.61891600	1.83079000
C	2.69384900	–0.26687800	4.18437900
H	4.83055800	–0.29075100	3.89498200
H	0.54076700	–0.12905500	4.17287300
H	2.74823000	–0.60261500	5.21656300
C	2.56065800	3.14237900	–0.25621100
C	3.78318900	3.79044600	–0.48359200
C	1.42594800	3.92735000	0.00168700
C	3.87271700	5.18110300	–0.45625900
H	4.67725700	3.20486500	–0.69293600
C	1.51056900	5.31800900	0.02444900
H	0.46119200	3.45465400	0.18309100
C	2.73461700	5.94559900	–0.20351300
H	4.82753300	5.66729000	–0.63603800
H	0.62069500	5.91030200	0.21751900
H	2.80086600	7.02990700	–0.18671800
C	3.68463900	0.50311400	–1.38684600
C	4.36715400	–0.68714200	–1.09101000
C	3.88255100	1.08327300	–2.65046500
C	5.21715300	–1.27826800	–2.02449000
H	4.21947700	–1.17775900	–0.13009500
C	4.72762900	0.49376000	–3.58973300
H	3.36839300	2.00902800	–2.90642800
C	5.39745000	–0.68821600	–3.27564300
H	5.73281900	–2.20170300	–1.77451900
H	4.86464300	0.95654400	–4.56299500
H	6.06020200	–1.14727700	–4.00415900

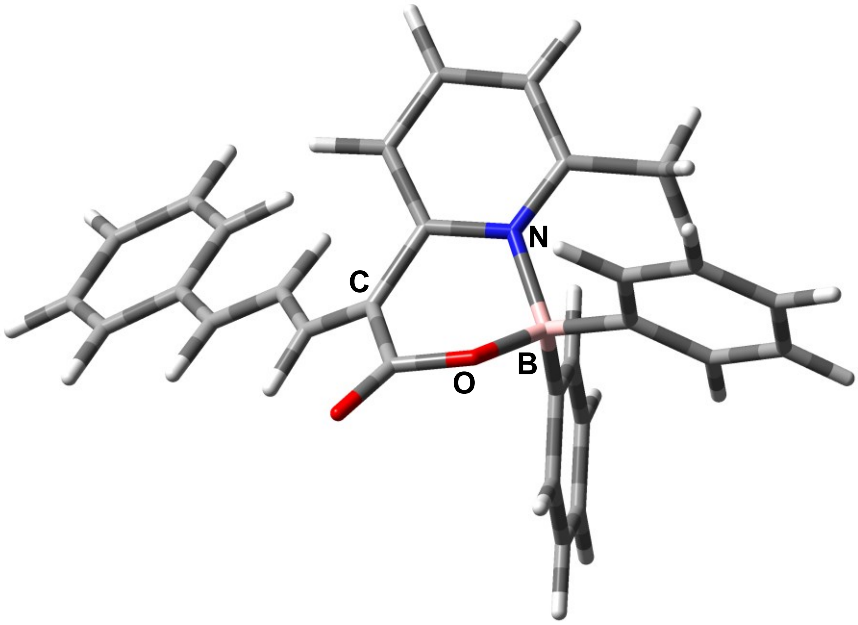

Figure S129. Optimized structure ***E*-11** [M06-2X/6–31+G(d)].

Table S60. Cartesian coordinates of the optimized structure ***E*-11** [M06-2X/6–31+G(d)].

Atomic symbol	x	y	z
O	–1.90895400	0.28649900	1.83811700
O	–0.43548500	0.84546200	3.42059500
N	–1.27745600	–1.15734800	–0.10499500
C	–4.47370800	0.78953000	–0.69333400
H	–3.97668600	1.54760600	–1.29715300
C	–3.71581400	0.02057700	0.19705400
C	–0.69268000	0.34882700	2.34819800
C	–1.46372200	1.44076700	–0.42601200
C	–0.09045600	–1.32917600	0.53923500
C	0.35278600	–0.29402000	1.48509300
C	–1.65641100	–2.02893300	–1.07076200
C	–5.84847500	0.59680800	–0.83571200
H	–6.41349400	1.20615900	–1.53652800
C	–4.39635600	–0.92620600	0.97628900
H	–3.83830300	–1.51087300	1.70648000
C	–2.85322400	–1.76146800	–1.94031100
H	–3.79201300	–1.97685300	–1.42291300
H	–2.78622100	–2.39533800	–2.82764100
H	–2.88714800	–0.71606000	–2.25374800
C	0.67536500	–2.47888900	0.33877600
H	1.57232500	–2.62032600	0.93114400
C	–0.75733800	1.33094600	–1.63086800
H	–0.59044100	0.34989000	–2.07699600
C	–1.62964100	2.73057500	0.10431800

H	–2.16882400	2.84821100	1.04299500
C	–6.49792000	–0.37565800	–0.07724600
H	–7.56755600	–0.53274700	–0.18813500
C	–0.89619800	–3.17284400	–1.31670800
H	–1.22994600	–3.86106300	–2.08535300
C	–5.76765100	–1.13482300	0.83877400
H	–6.26972500	–1.88174500	1.44853300
C	0.25891400	–3.41988000	–0.58913300
H	0.83352100	–4.32654900	–0.75467200
C	–0.41832600	3.71911700	–1.73770700
H	–0.01480400	4.59404900	–2.24005400
C	–1.11572300	3.85575500	–0.53690700
H	–1.25638100	4.84056800	–0.09913600
C	–0.23963800	2.45071400	–2.28474400
H	0.30547300	2.33171100	–3.21782100
B	–2.12598300	0.20192700	0.36837800
C	2.77286100	–0.18510400	0.79313500
H	2.61361300	–0.77658600	–0.10548900
C	4.00220200	0.27966200	1.09712900
H	4.10816000	0.87327500	2.00610400
C	1.62906100	0.13139200	1.62065300
H	1.78866400	0.85418200	2.42085400
C	5.23856300	0.07212000	0.33789500
C	6.43802500	0.58589400	0.85171900
C	5.27677600	–0.61439600	–0.88660600
C	7.64054900	0.41697300	0.17112900
H	6.42189400	1.12448800	1.79633200
C	6.47603700	–0.78338800	–1.56682400
H	4.36226000	–1.01236500	–1.31667600
C	7.66349100	–0.26959400	–1.04058200
H	8.55832900	0.82261400	0.58632600
H	6.48740900	–1.31530200	–2.51369000
H	8.59941800	–0.40251500	–1.57503600

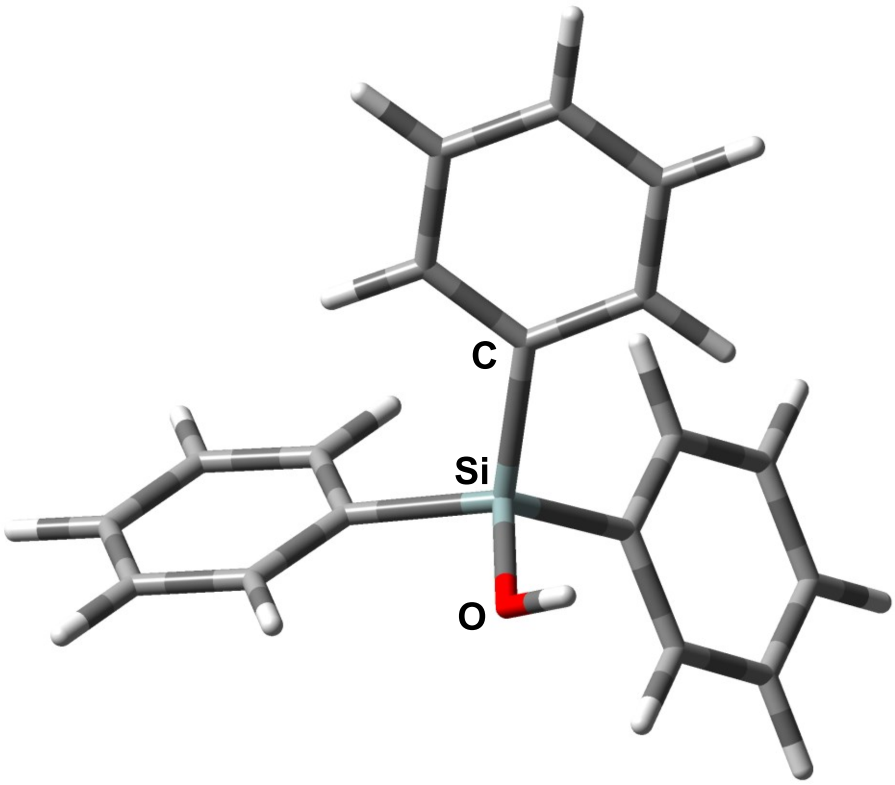

Figure S130. Optimized structure **Ph_3SiOH** [M06-2X/6–31+G(d)].

Table S61. Cartesian coordinates of the optimized structure **Ph_3SiOH** [M06-2X/6–31+G(d)].

Atomic symbol	x	y	z
O	–0.10841700	–0.19457500	2.44543700
Si	–0.00923500	–0.05944200	0.78340100
C	–0.06213100	1.73701400	0.23814200
C	–1.26704700	2.33705300	–0.15753400
C	1.09682500	2.53057800	0.25313600
C	–1.31638300	3.68257900	–0.51988800
H	–2.17862800	1.74284300	–0.18873700
C	1.05351500	3.87696300	–0.10435300
H	2.05178900	2.08833800	0.53537100
C	–0.15547700	4.45398700	–0.49198000
H	–2.25845600	4.12787800	–0.82775000
H	1.96135100	4.47367300	–0.08817700
H	–0.19105900	5.50202800	–0.77661600
C	–1.50510400	–0.97506000	0.14483200
C	–1.60886500	–1.31024700	–1.21311900
C	–2.57226100	–1.29804500	0.99418500
C	–2.74547900	–1.94183300	–1.71245200
H	–0.78942700	–1.07889200	–1.89287100
C	–3.71165300	–1.93343000	0.49985200
H	–2.50534400	–1.05275800	2.05151700
C	–3.80005700	–2.25317100	–0.85371800

H	–2.80840700	–2.19471700	–2.76723100
H	–4.53006800	–2.17963500	1.17092100
H	–4.68780700	–2.74655600	–1.24000600
C	1.59348200	–0.82323200	0.18360400
C	2.15701500	–0.47994100	–1.05462000
C	2.24183000	–1.79619300	0.95863300
C	3.32489200	–1.09096000	–1.50835500
H	1.68517900	0.28448700	–1.67112300
C	3.41360600	–2.40645800	0.51309200
H	1.82316100	–2.07727200	1.92312800
C	3.95508600	–2.05509600	–0.72257000
H	3.74643300	–0.81091700	–2.46993200
H	3.90367400	–3.15610600	1.12840700
H	4.86821200	–2.52934500	–1.07186600
H	0.35525900	0.46870000	2.97253900

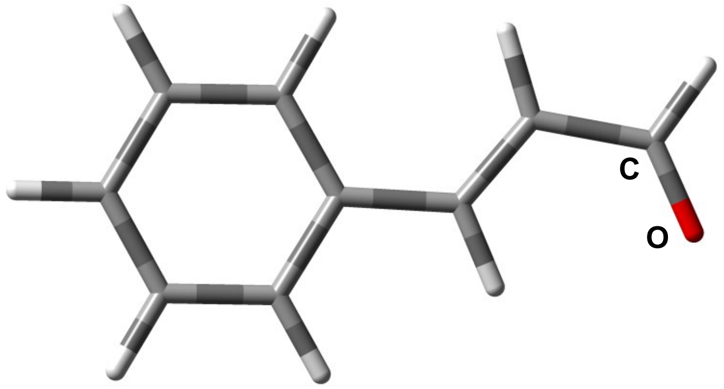

Figure S131. Optimized structure **Cinnamaldehyde** [M06-2X/6–311+G(d,p)].

Table S62. Cartesian coordinates of the optimized structure **Cinnamaldehyde** [M06-2X/6–311+G(d,p)].

Atomic symbol	x	y	z
O	3.85417100	–0.74111000	0.00046500
C	1.05134600	–0.26167100	0.00004600
C	1.97047800	0.71309400	–0.00053000
C	–0.40599600	–0.11287900	0.00010100
C	–1.04224600	1.13545800	0.00034400
C	–1.19708000	–1.26700300	–0.00006600
C	–2.42543800	1.22172200	0.00020100
H	–0.45304200	2.04469100	0.00059000
C	–2.58409200	–1.18179100	–0.00021000
H	–0.71354100	–2.23795700	–0.00009500
C	–3.20150700	0.06320600	–0.00008800
H	–2.90424900	2.19360000	0.00040800
H	–3.18096900	–2.08593600	–0.00041900

H	–4.28260300	0.13464400	–0.00021100
H	1.42850600	–1.28271700	0.00050000
H	1.70930700	1.76580500	–0.00094100
C	3.41188900	0.38128000	–0.00027900
H	4.09911100	1.24824300	–0.00066300

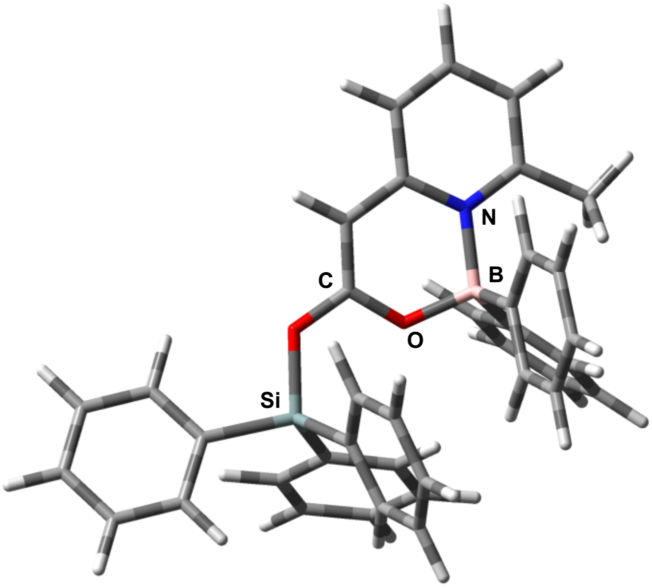

Figure S132. Optimized structure **5** [M06-2X/6–311+G(d,p)].

Table S63. Cartesian coordinates of the optimized structure **5** [M06-2X/6–311+G(d,p)].

Atomic symbol	x	y	z
Si	–2.29398400	–0.06200900	0.25625100
O	–1.30516300	–0.27481400	1.63812200
O	0.53830900	0.19110400	0.50866400
N	2.82944800	–0.00503200	1.57569400
C	0.01756900	–0.23811600	1.61139100
C	2.31750400	1.44353900	–0.61877900
C	2.16548000	–0.38561600	2.70952600
C	4.18387000	0.17195300	1.60272400
C	0.75866500	–0.58867000	2.70404600
H	0.26245200	–0.91296900	3.60438700
C	–3.99585900	–0.18794300	1.01374400
C	–1.05244200	1.83754700	–1.47743100
H	–0.35681900	1.04180900	–1.71815600
C	–1.90580700	–1.46666900	–0.90551400
C	2.22943700	–1.32898400	–0.62457200
C	2.88121200	–0.56794000	3.91261000
H	2.32704800	–0.86314800	4.79361800
C	2.04790300	2.64804600	0.04380000
H	1.65136800	2.61884000	1.05579700

C	4.89651200	0.00451600	2.77328900
H	5.96772400	0.14778600	2.75767500
C	–2.08521700	1.60459500	–0.55932000
C	2.97706100	–2.40150700	–0.12645700
H	3.46512400	–2.31888700	0.84134700
C	4.23453600	–0.36793700	3.94623100
H	4.78793700	–0.50609200	4.86784000
C	–5.10398400	–0.48067200	0.20974700
H	–4.96405100	–0.69681800	–0.84607900
C	3.12112700	–3.59069800	–0.83917200
H	3.70732600	–4.40262500	–0.42300200
C	2.51856500	–3.73334500	–2.08328700
H	2.63574300	–4.65309400	–2.64529300
C	–4.20809800	0.07153000	2.37246700
C	–5.49130100	0.04688500	2.91084100
H	–5.64038200	0.24983400	3.96514900
C	–6.58172300	–0.24090800	2.09674500
H	–7.58142700	–0.26213600	2.51505900
C	2.79450400	1.52866800	–1.92955700
H	3.02500500	0.61894000	–2.47576700
C	–2.96155200	2.65615500	–0.26275100
H	–3.78035200	2.49738900	0.43275000
C	1.75728500	–2.68687900	–2.59735300
H	1.26906900	–2.79074900	–3.55989600
C	1.60902900	–1.51211600	–1.87022400
H	0.99337800	–0.71712600	–2.28213200
C	–1.75880500	4.12120400	–1.75106500
H	–1.62842800	5.09699700	–2.20576500
C	–0.88597400	3.08361300	–2.06790400
H	–0.06408700	3.24532400	–2.75626500
C	–6.38758500	–0.50713100	0.74440700
H	–7.23488200	–0.73926900	0.10962700
C	–2.79870100	3.90693300	–0.85240700
H	–3.48261900	4.71222600	–0.61043300
C	–2.16438000	–1.37388100	–2.27762200
H	–2.53228400	–0.44095000	–2.69608400
C	4.93221100	0.51637300	0.34649400
H	4.61353800	–0.10295200	–0.49209300
H	5.99557400	0.35512000	0.51916200
H	4.77221400	1.55966400	0.06906400

C	3.00977200	2.75908800	–2.54894500
H	3.38398700	2.79393900	–3.56629700
C	–1.40425500	–2.67288800	–0.39866000
H	–1.17992200	–2.76336100	0.66024000
C	2.26994000	3.88251400	–0.55709200
H	2.05515300	4.79709100	–0.01560000
C	2.75527300	3.94143800	–1.86089000
H	2.92851800	4.90000600	–2.33714100
C	–1.94898400	–2.46360400	–3.11752600
H	–2.15668500	–2.37913100	–4.17818500
C	–1.46362900	–3.65742100	–2.59427000
H	–1.28897800	–4.50436500	–3.24844500
C	–1.18187800	–3.75947900	–1.23531700
H	–0.77769700	–4.68048800	–0.83167300
B	2.00985300	0.06167200	0.16788600
H	–3.36102200	0.29071400	3.01381700

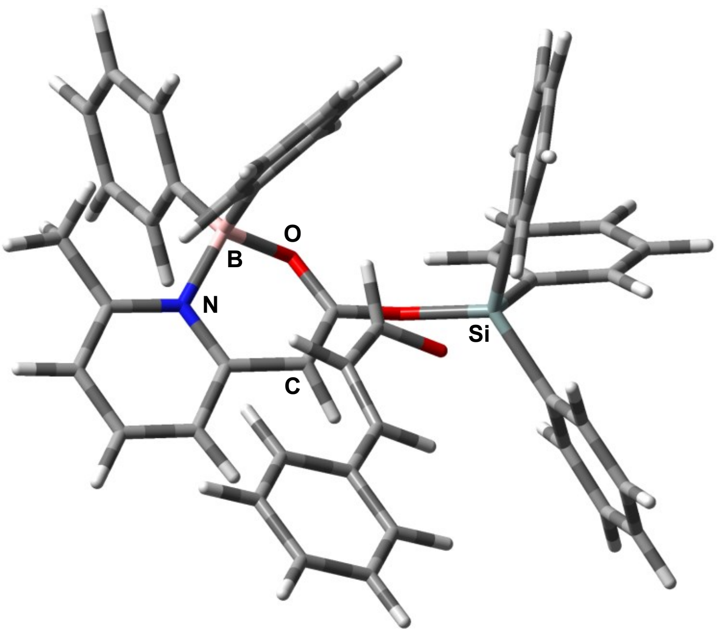

Figure S133. Optimized structure **TS$_{ZT}$** [M06-2X/6–311+G(d,p)].

Table S64. Cartesian coordinates of the optimized structure **TS$_{ZT}$** [M06-2X/6–311+G(d,p)].

Atomic symbol	x	y	z
O	1.41197500	–1.53370300	–0.81769100
N	3.07721400	0.35686100	–1.05958700
C	2.02092500	1.09982700	–1.49139100
C	3.76270100	–2.21193400	–0.55751900
C	4.34170900	0.84738700	–1.16314500
C	2.47277500	–0.60672900	1.28383600
C	2.22271700	2.34489700	–2.09872600

H	1.35659200	2.90713500	–2.42306300
C	0.66048300	0.63385000	–1.29834400
C	4.48732500	–2.85971000	0.44487000
H	4.36056900	–2.54818800	1.47788300
C	4.56798900	2.07240700	–1.77370000
H	5.58414200	2.43508000	–1.84626300
C	3.93701000	–2.65290600	–1.87475100
H	3.36652100	–2.18543900	–2.67349000
C	3.50291900	2.82745300	–2.25200000
H	3.67622300	3.78867600	–2.72162100
C	2.85856300	0.61306900	1.84874000
H	3.43946200	1.32635400	1.26766000
C	5.37741100	–3.88850000	0.14530300
H	5.92867000	–4.37713500	0.94099200
C	5.55745600	–4.29128200	–1.17368600
H	6.25181800	–5.08911400	–1.41081200
C	1.71379200	–1.47430500	2.08503800
H	1.38280700	–2.42380300	1.67336300
C	5.50715500	0.11198900	–0.56509400
H	5.27775400	–0.23568700	0.44242600
H	6.35899000	0.78957900	–0.52045100
H	5.78010500	–0.76263700	–1.15767000
C	4.82873700	–3.67313000	–2.18729800
H	4.95257600	–3.99249000	–3.21598800
C	1.33319700	–1.12680800	3.37610700
H	0.72004200	–1.80411300	3.95854400
C	2.48693400	0.96981700	3.14478300
H	2.79155600	1.92818800	3.55121200
B	2.72929800	–1.02371200	–0.25315000
C	1.71297500	0.10350700	3.90760900
H	1.40185500	0.38428300	4.90719800
C	0.43452700	–0.75032800	–1.09033700
C	–0.34730900	1.12949100	0.39643700
O	–1.54443800	0.83949900	0.03745000
C	–0.79489800	3.52800700	0.12822800
C	0.04767600	2.55034400	0.47828200
C	–0.51492200	4.96950400	0.14334100
C	0.74720200	5.49547400	0.45089400
C	–1.54660800	5.85901200	–0.17360500
C	0.96260300	6.86540200	0.44844700

H	1.56764900	4.82864900	0.69102300
C	–1.33189600	7.23268600	–0.17636000
H	–2.52680200	5.46310600	–0.41781800
C	–0.07651800	7.74055400	0.13585500
H	1.94391100	7.25595800	0.69150800
H	–2.14547600	7.90497000	–0.42138700
H	0.09506300	8.81030300	0.13509600
H	–1.78871100	3.22885700	–0.20018000
H	1.05372000	2.73727800	0.83963700
H	–0.09191000	1.15346000	–1.87404300
O	–0.74025500	–1.25126200	–1.11714900
Si	–2.28012600	–0.96895800	–0.21349100
H	0.15858600	0.46546500	1.10736000
C	–2.15199500	–1.23349800	1.67379700
C	–1.96949600	–2.52717400	2.18663400
C	–2.27064100	–0.18882500	2.60374500
C	–1.91283400	–2.76729500	3.55662100
H	–1.88123000	–3.36675400	1.50597400
C	–2.23483400	–0.42406700	3.97527500
H	–2.39709600	0.82927100	2.25470400
C	–2.05710300	–1.71660800	4.45649500
H	–1.76854000	–3.77874400	3.91996600
H	–2.34700500	0.40320500	4.66717700
H	–2.03124200	–1.90340200	5.52446900
C	–3.72227900	–0.02070200	–1.00994700
C	–4.32287500	1.08935900	–0.40169900
C	–4.21477900	–0.42438500	–2.25541800
C	–5.38496800	1.75673700	–1.00265700
H	–3.95556000	1.43788400	0.55794500
C	–5.25295900	0.26135500	–2.88140300
H	–3.78925100	–1.29685700	–2.74147900
C	–5.84706900	1.34860400	–2.25134200
H	–5.85077800	2.59762200	–0.50046900
H	–5.60615700	–0.06414100	–3.85334800
H	–6.66832200	1.87282200	–2.72704100
C	–2.85091700	–2.74685700	–0.71485400
C	–4.16761900	–3.10400700	–0.38402700
C	–2.05256600	–3.73791800	–1.29513400
C	–4.66482700	–4.38074400	–0.61672400
H	–4.82450400	–2.36505100	0.06950500

C	–2.54217300	–5.02163900	–1.53904700
H	–1.02495900	–3.51136300	–1.55502400
C	–3.84917700	–5.34782700	–1.20011200
H	–5.68666600	–4.62289100	–0.34600900
H	–1.89750800	–5.76683800	–1.99241800
H	–4.23149200	–6.34515200	–1.38630800

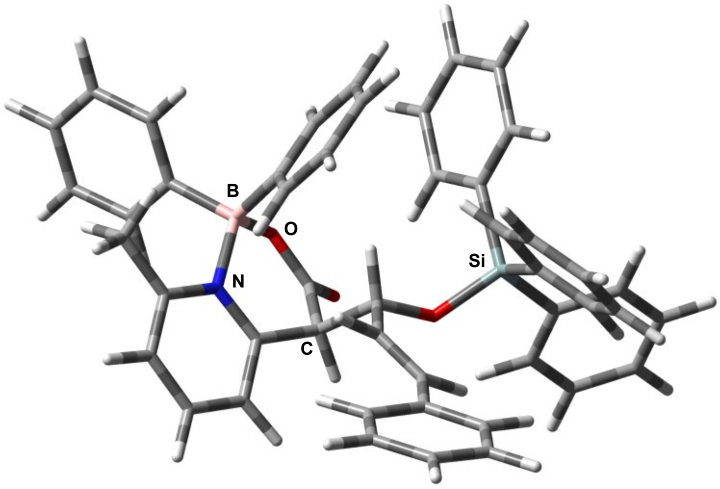

Figure S134. Optimized structure **16** [M06-2X/6–311+G(d,p)].

Table S65. Cartesian coordinates of the optimized structure **16** [M06-2X/6–311+G(d,p)].

Atomic symbol	x	y	z
O	2.49051600	–1.27680900	–1.01000000
N	2.92554300	1.21405000	–0.92421100
C	1.86392800	1.34354200	–1.75265700
C	4.68368400	–0.64992900	–0.12326500
C	3.76293600	2.26673600	–0.72817400
C	2.39427300	–0.11133500	1.29059100
C	1.66033900	2.50308500	–2.49198900
H	0.80076600	2.55722300	–3.14668900
C	0.84978400	0.23987800	–1.84401600
C	5.39207900	–0.92025700	1.04936100
H	4.88779500	–0.83664100	2.00797700
C	3.57593800	3.44524500	–1.44190900
H	4.25990300	4.26530400	–1.26835300
C	5.36846900	–0.78141100	–1.33723400
H	4.83538000	–0.61360200	–2.26980000
C	2.53422000	3.56406500	–2.34702000
H	2.39313800	4.47881200	–2.91052800
C	1.98280000	1.08460800	1.88883300
H	2.17257500	2.03654800	1.39797100
C	6.73854600	–1.27755900	1.01803000

H	7.26615300	–1.48231400	1.94296600
C	7.40710700	–1.37065300	–0.19795400
H	8.45597900	–1.64338100	–0.22525300
C	2.11277400	–1.30027800	1.97971100
H	2.41238600	–2.24730800	1.54161800
C	4.87209400	2.21025100	0.28304200
H	4.57118800	1.67306100	1.18041200
H	5.14537000	3.23080500	0.55152400
H	5.74995400	1.70427700	–0.12382600
C	6.71509900	–1.12543900	–1.38190000
H	7.22333100	–1.21323000	–2.33575700
C	1.43940300	–1.29609600	3.19617000
H	1.22867600	–2.23310800	3.69762200
C	1.30189500	1.10207000	3.10551200
H	0.98445600	2.04634500	3.53469900
B	3.12337200	–0.23621000	–0.14497100
C	1.02461600	–0.09197600	3.76111400
H	0.49016200	–0.08639600	4.70445100
C	1.49466600	–1.13209200	–1.85258900
C	–0.23247700	0.28491400	–0.70870600
O	–1.29536700	–0.51239000	–1.16365300
C	–1.83005700	2.22241800	–0.73046400
C	–0.64494000	1.70237900	–0.42045700
C	–2.25082700	3.59471000	–0.40172800
C	–1.33462800	4.64178700	–0.25109100
C	–3.60961800	3.86227100	–0.21194600
C	–1.76450800	5.91354200	0.10411500
H	–0.28011100	4.45859100	–0.43083500
C	–4.04067100	5.13425700	0.14637400
H	–4.32564200	3.05527500	–0.32699900
C	–3.11942300	6.16373600	0.30871000
H	–1.04327500	6.71514000	0.21442100
H	–5.09717400	5.32226200	0.29858400
H	–3.45381500	7.15675800	0.58430700
H	–2.57319800	1.58378700	–1.20091100
H	0.09212700	2.29613100	0.11510200
H	0.28783900	0.35752000	–2.77079500
O	1.09712200	–2.01426400	–2.56886400
Si	–2.29713700	–1.42365500	–0.17428400
H	0.20239000	–0.14293700	0.20446500

C	–3.26694400	–0.29359500	0.96623100
C	–2.60699400	0.34909200	2.02460000
C	–4.60650500	0.03278100	0.72674300
C	–3.25860500	1.29743900	2.80406300
H	–1.56347800	0.11928600	2.23448500
C	–5.26971300	0.96758500	1.51881500
H	–5.13878800	–0.44446500	–0.09082600
C	–4.59362000	1.60612400	2.55265900
H	–2.72675900	1.79854700	3.60506500
H	–6.30995100	1.20287200	1.32253400
H	–5.10271200	2.34612700	3.15953700
C	–3.40739200	–2.31319200	–1.37753000
C	–4.50421500	–3.05142400	–0.91451900
C	–3.13570600	–2.30720400	–2.74980600
C	–5.31780600	–3.75113300	–1.79790300
H	–4.72611200	–3.08383800	0.14945300
C	–3.94801500	–3.00885300	–3.63657500
H	–2.27510200	–1.75902000	–3.11866600
C	–5.04011700	–3.72710600	–3.16224200
H	–6.16364500	–4.31768400	–1.42580900
H	–3.72465900	–2.99923800	–4.69720200
H	–5.67160800	–4.27454700	–3.85259400
C	–1.27887200	–2.62200700	0.83882200
C	–1.60626700	–2.94590300	2.16163200
C	–0.22706500	–3.31322600	0.22216700
C	–0.90619500	–3.93371600	2.84799500
H	–2.41755800	–2.42577300	2.66233700
C	0.48431800	–4.28969500	0.91186200
H	0.03798600	–3.09796800	–0.80880100
C	0.14323000	–4.60281300	2.22446000
H	–1.17582600	–4.17838000	3.86956100
H	1.30072800	–4.80633100	0.41987100
H	0.69109700	–5.37065800	2.75951000

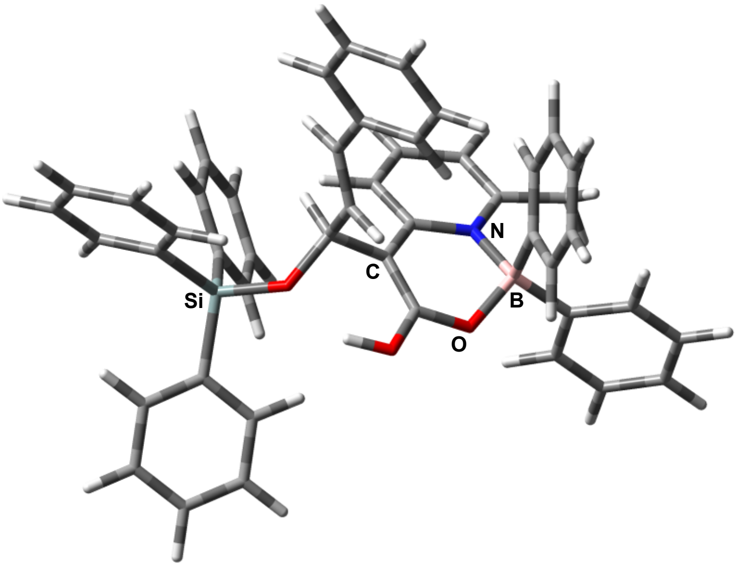

Figure S135. Optimized structure **17*Z*** [M06-2X/6–311+G(d,p)].

Table S66. Cartesian coordinates of the optimized structure **17*Z*** [M06-2X/6–311+G(d,p)].

Atomic symbol	x	y	z
O	–2.26675800	–1.09627000	–1.32677600
O	–0.38664000	–0.55727500	–2.31945200
N	–2.63514500	–0.89246700	1.13397900
C	–1.06577400	–0.64438000	–1.18323100
C	–1.28121000	–0.69236200	1.22286700
C	–4.58057200	–1.53731000	–0.65693100
C	–3.36735500	–1.15903200	2.25235900
C	–3.51170400	0.97753500	–0.47463500
C	–0.63710500	–0.86408200	2.46395500
H	0.43789100	–0.77664000	2.52546500
C	–0.53328600	–0.33061800	0.04814300
C	–5.84423900	–1.01079500	–0.93313500
H	–5.98571700	0.06614300	–0.92271400
C	–2.74795400	–1.32380200	3.47789900
H	–3.35631700	–1.53376600	4.34664500
C	–4.43731900	–2.92967900	–0.69877500
H	–3.45933800	–3.36739600	–0.51760300
C	–1.36444600	–1.19633100	3.57812100
H	–0.86187800	–1.34917200	4.52666000
C	–3.56738600	1.86542700	0.60501900
H	–3.41150300	1.49920400	1.61682900
C	–6.93269800	–1.83695600	–1.20436700
H	–7.90443000	–1.40214500	–1.41108000
C	–6.77455000	–3.21874700	–1.20877200
H	–7.62012400	–3.86512500	–1.41487100

C	–3.68894400	1.51878000	–1.75662600
H	–3.64209200	0.85723600	–2.61725300
C	–4.86983700	–1.19549800	2.20193700
H	–5.26370300	–0.34775700	1.64069400
H	–5.24833000	–1.14797300	3.22245800
H	–5.24312600	–2.10361800	1.72762800
C	–5.51772200	–3.76566000	–0.96040700
H	–5.38199900	–4.84150200	–0.97660400
C	–3.93093100	2.87566200	–1.94978300
H	–4.06854300	3.26391900	–2.95317000
C	–3.81156200	3.22617900	0.42480900
H	–3.84480300	3.88943300	1.28199400
B	–3.29652800	–0.61992300	–0.33586500
C	–4.00216600	3.73424300	–0.85504600
H	–4.19516100	4.79152300	–0.99928800
C	0.77234200	0.42556400	0.11201800
C	0.54405400	1.85138400	–0.30155300
H	0.06307000	1.99497600	–1.26733700
C	0.84709000	2.89413500	0.47104300
H	1.36289700	2.72021800	1.41457200
C	0.54558400	4.29651200	0.13859300
C	1.34709700	5.32251000	0.64760300
C	–0.54159000	4.63115700	–0.67638300
C	1.09025200	6.65023500	0.32592500
H	2.18375000	5.07202700	1.29209100
C	–0.79909100	5.95787200	–0.99498300
H	–1.20753900	3.85220600	–1.03198800
C	0.01773500	6.97151800	–0.50036500
H	1.72501000	7.43395200	0.72246800
H	–1.64966600	6.20176700	–1.62143300
H	–0.18790100	8.00649900	–0.74719800
H	1.17463200	0.42491400	1.12688700
O	1.73287000	–0.17348700	–0.78553500
Si	3.11435000	–0.97667800	–0.27724300
C	3.42598100	–2.25274000	–1.59980400
C	4.72105800	–2.59718300	–2.00285100
C	2.34355000	–2.91532800	–2.19510600
C	4.93062900	–3.58086000	–2.96442900
H	5.57634900	–2.08477500	–1.57241500
C	2.54957300	–3.89681700	–3.15877400

H	1.32557700	–2.65811100	–1.91410900
C	3.84485500	–4.23148000	–3.54173900
H	5.93947900	–3.83400700	–3.26899800
H	1.70109900	–4.39303400	–3.61513100
H	4.00680200	–4.99382300	–4.29507600
C	2.75435900	–1.77135200	1.38750600
C	3.09830300	–1.13969800	2.59050000
C	2.04421600	–2.97783000	1.45332300
C	2.73998500	–1.69097000	3.81856000
H	3.65479400	–0.20702200	2.56896300
C	1.67997700	–3.52977000	2.67704400
H	1.77535800	–3.49624500	0.53791200
C	2.02792900	–2.88615900	3.86122900
H	3.01829400	–1.19000100	4.73870500
H	1.12599500	–4.46074400	2.70745600
H	1.74939300	–3.31870600	4.81563700
C	4.49689900	0.26336700	–0.08856500
C	5.69663100	–0.07023200	0.55543400
C	4.35107600	1.55893000	–0.59823600
C	6.72398400	0.85860800	0.67717000
H	5.82826600	–1.06393400	0.97684800
C	5.37558200	2.49334200	–0.47201800
H	3.42549700	1.84021000	–1.09064100
C	6.56227100	2.14278400	0.16263500
H	7.64635500	0.58563400	1.17649400
H	5.24564900	3.49340200	–0.86946900
H	7.36060300	2.86944500	0.26045400
H	0.55593300	–0.41100400	–2.10655400

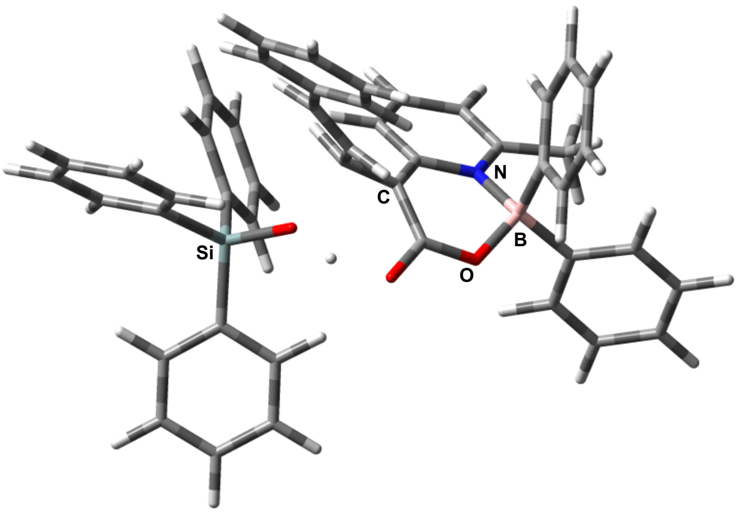

Figure S136. Structure **TS*Z*** [M06-2X/6–311+G(d,p)], obtained as a single-point calculation on the geometry optimized at the M06-2X/6–31+G(d) level of theory.

Table S67. Cartesian coordinates of structure **TS*Z*** [M06-2X/6–311+G(d,p)].

Atomic symbol	x	y	z
O	–2.45837300	–0.18169800	–1.36349900
O	–0.36625100	0.05647600	–2.06295300
N	–2.92975500	–0.49481300	1.08267100
C	–1.21044400	0.06759200	–1.12173100
C	–1.57456800	–0.40271400	1.27813900
C	–4.82203700	–0.56647700	–0.88130000
C	–3.73859100	–0.94217500	2.08887800
C	–3.58304200	1.74982900	–0.10068400
C	–1.00758200	–0.87153800	2.48235200
H	0.06842600	–0.85653000	2.61012400
C	–0.77547200	0.19415200	0.24250800
C	–6.03548200	0.10471300	–1.06523300
H	–6.10371400	1.16027200	–0.80540800
C	–3.19834800	–1.40071700	3.27839700
H	–3.86665000	–1.75109100	4.05626900
C	–4.77106000	–1.91977700	–1.24757500
H	–3.83148800	–2.46135000	–1.14401100
C	–1.81405700	–1.38524400	3.46714100
H	–1.37523700	–1.76682500	4.38507400
C	–3.66247200	2.37861800	1.14821800
H	–3.63134800	1.78105700	2.05958700
C	–7.16465500	–0.55092200	–1.55867200
H	–8.09638400	–0.00605100	–1.68833100
C	–7.09840400	–1.90416600	–1.88609700
H	–7.97649300	–2.41937000	–2.26654400
C	–3.60768600	2.57699600	–1.23594700
H	–3.54660500	2.11857900	–2.22208900
C	–5.23706900	–0.87609900	1.96717700
H	–5.56251700	0.10517600	1.61294300
H	–5.67055700	–1.05160500	2.95430000
H	–5.63072900	–1.62070000	1.27155700
C	–5.89178400	–2.58905100	–1.73487500
H	–5.82649800	–3.64093500	–2.00198400
C	–3.70588500	3.96311700	–1.13086100
H	–3.72169800	4.57586100	–2.02882200
C	–3.76538500	3.76607500	1.26768800
H	–3.82399000	4.22327800	2.25228600
B	–3.49065700	0.15007300	–0.31893800

C	–3.78646000	4.56411800	0.12614500
H	–3.86447700	5.64450900	0.21418400
C	0.54086500	0.70525600	0.44549700
C	0.89627500	2.02434200	–0.09921400
H	0.32987000	2.36763400	–0.96177500
C	1.86856100	2.76123300	0.45950800
H	2.42443600	2.33295200	1.29651100
C	2.31041900	4.09141400	0.02207600
C	3.55988300	4.56364600	0.44659400
C	1.53136600	4.90503300	–0.81470300
C	4.03274400	5.80517600	0.02931400
H	4.16720800	3.94217800	1.10152800
C	2.00204500	6.14579400	–1.22794100
H	0.54505200	4.57314000	–1.12684300
C	3.25541800	6.59909900	–0.81185300
H	5.00543400	6.15394500	0.36397300
H	1.38580500	6.76670800	–1.87156400
H	3.61882800	7.56957900	–1.13665600
H	1.01566000	0.49502000	1.40395000
O	1.59435100	–0.28359000	–0.68842600
Si	2.66307700	–1.51241600	–0.29071900
C	2.55429500	–2.80649800	–1.63018700
C	3.66185200	–3.59121800	–1.98306300
C	1.33583500	–3.03615900	–2.28841700
C	3.55512700	–4.58340100	–2.95587600
H	4.62404000	–3.42030200	–1.50305300
C	1.22627900	–4.02527400	–3.26469500
H	0.46168200	–2.42969100	–2.05494100
C	2.33564800	–4.80174400	–3.59627900
H	4.42366300	–5.18004500	–3.22014900
H	0.27724900	–4.18268100	–3.76910600
H	2.25147000	–5.57109500	–4.35874300
C	2.16330100	–2.22747700	1.37385300
C	2.64323500	–1.67743600	2.57396300
C	1.25960300	–3.29795200	1.45303500
C	2.23016700	–2.17387100	3.81072800
H	3.35716200	–0.85503000	2.54359700
C	0.84331700	–3.79943600	2.68549800
H	0.87710700	–3.74865000	0.53891100
C	1.32916700	–3.23767900	3.86565700

H	2.61642300	–1.73824300	4.72823300
H	0.14036100	–4.62694100	2.72512800
H	1.01106000	–3.63211900	4.82728000
C	4.33833600	–0.69903900	–0.12165400
C	5.41572600	–1.34195800	0.50823400
C	4.53707600	0.59754800	–0.61950300
C	6.65499100	–0.71612700	0.62834800
H	5.28494800	–2.34050300	0.92459000
C	5.77385600	1.23118700	–0.49575800
H	3.71202800	1.11774100	–1.10170100
C	6.83390300	0.57347000	0.12645400
H	7.47777600	–1.22917400	1.11835600
H	5.90681000	2.23636400	–0.88698100
H	7.79853100	1.06411500	0.22333100
H	0.83519300	–0.20707100	–1.47763900

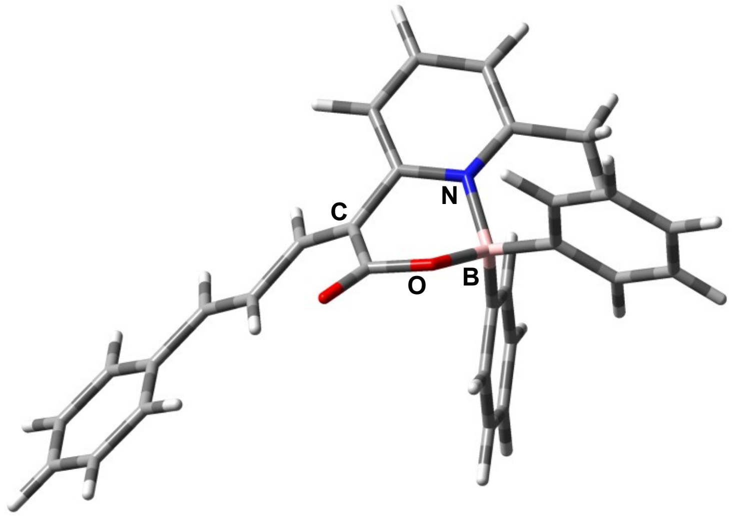

Figure S137. Optimized structure ***Z*-11** [M06-2X/6–311+G(d,p)].

Table S68. Cartesian coordinates of the optimized structure ***Z*-11** [M06-2X/6–311+G(d,p)].

Atomic symbol	x	y	z
O	–1.11474200	–0.33130700	–1.54458500
O	0.91914000	–0.27250200	–2.42877600
N	–1.87112800	1.32456600	0.16363000
C	–4.10833000	–1.73295900	–0.06183400
H	–3.66559000	–2.20651900	0.81004700
C	–3.39087500	–0.74509400	–0.73967600
C	0.12860900	0.09495000	–1.59647400
C	–1.24275000	–1.10848500	0.90791900
C	–0.67293800	1.93205400	–0.04230900
C	0.44951200	1.13434200	–0.56525800
C	–2.87726600	1.99770300	0.77232900

C	–5.38298100	–2.11569100	–0.47345900
H	–5.92024800	–2.88521900	0.06976200
C	–3.98135400	–0.17811300	–1.87565700
H	–3.42740800	0.56258900	–2.44576200
C	1.69711000	1.33747200	–0.08649700
H	1.81843200	2.06740000	0.71216900
C	–4.14599800	1.30491300	1.18028800
H	–4.79683600	1.11267400	0.32595200
H	–4.67261800	1.93882200	1.89307400
H	–3.93410000	0.34334900	1.64707900
C	–0.49804100	3.28547800	0.24172800
H	0.44457300	3.75490800	–0.00229400
C	2.89783600	0.63448200	–0.47224200
H	2.81433000	–0.09663900	–1.26443700
C	–1.19833200	–0.68676300	2.24063200
H	–1.59506400	0.28707600	2.51808400
C	–0.69492200	–2.36489800	0.61382500
H	–0.71020500	–2.71776000	–0.41338600
C	–5.96564900	–1.51376500	–1.58384900
H	–6.95861800	–1.80658200	–1.90530400
C	–2.72306600	3.34412000	1.08970200
H	–3.54550900	3.86057300	1.56606500
C	–5.25679000	–0.54530300	–2.29156400
H	–5.69633200	–0.08659600	–3.17034500
C	5.35586700	0.25290900	–0.13789700
C	–1.53788200	4.00109900	0.80291400
H	–1.42434500	5.05564400	1.02488900
C	4.06770200	0.89047000	0.14104600
H	4.08390100	1.63948800	0.93165900
C	–0.10766200	–2.72390700	2.92508500
H	0.32885800	–3.34454100	3.69905500
C	–0.13381500	–3.16379400	1.60411600
H	0.28503400	–4.13022900	1.34679700
C	6.46182400	0.59571000	0.64886600
H	6.34014000	1.32649000	1.44161600
C	–0.64181800	–1.48058300	3.24170300
H	–0.62155900	–1.12623200	4.26641200
C	5.52912700	–0.69079700	–1.16053300
H	4.69233500	–0.97009500	–1.78940900
C	7.70374800	0.01255500	0.43028300

H	8.54765400	0.28881800	1.05114900
B	–1.91970900	–0.27333100	–0.29391700
C	7.86047800	–0.92383700	–0.58510100
H	8.82709200	–1.38142400	–0.75938200
C	6.76869500	–1.27121300	–1.37959900
H	6.88709700	–1.99916300	–2.17334800

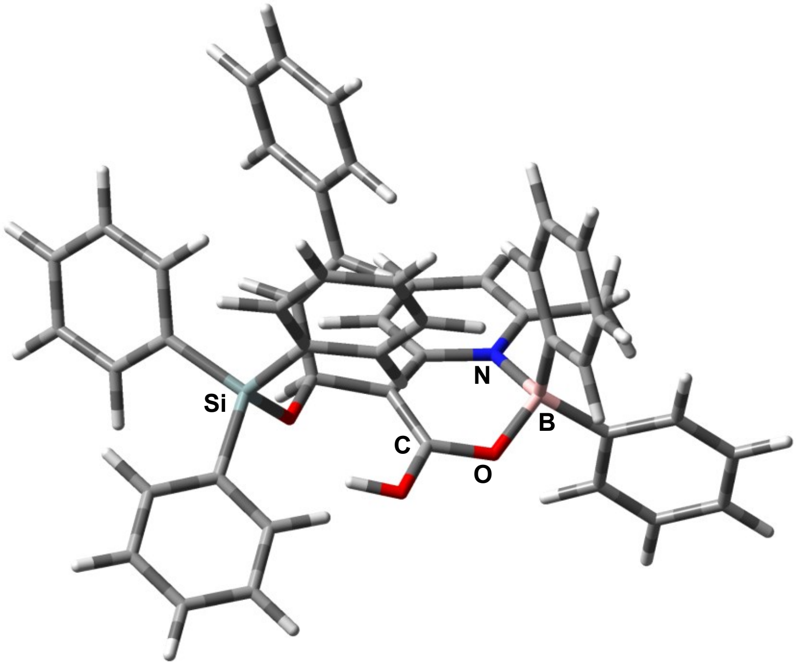

Figure S138. Optimized structure **17*E*** [M06-2X/6–311+G(d,p)].

Table S69. Cartesian coordinates of the optimized structure **17*E*** [M06-2X/6–311+G(d,p)].

Atomic symbol	x	y	z
O	–2.68353000	1.71270700	–0.25659100
O	–0.73857900	2.69190300	–0.24352800
N	–3.17503300	–0.43917700	–1.40842800
C	–1.44195000	1.62970300	–0.60113700
C	–1.90717700	–0.33451000	–1.91375800
C	–5.02592400	1.03433600	–0.11510000
C	–4.04800000	–1.35931700	–1.90810000
C	–3.05858000	–0.36374400	1.20878000
C	–1.55251200	–1.11342200	–3.03696700
H	–0.56618200	–1.01029700	–3.46368900
C	–0.94718400	0.56170800	–1.32469700
C	–5.97184400	0.77995100	0.88068700
H	–5.70354900	0.15167600	1.72496000
C	–3.70975600	–2.13129000	–3.00327400
H	–4.42268900	–2.85392900	–3.37531900
C	–5.41903000	1.85278000	–1.18144500
H	–4.69854500	2.08956000	–1.95958900

C	–2.45398700	–1.98672200	–3.58857300
H	–2.18284100	–2.57390900	–4.45842400
C	–2.60626200	–1.68786400	1.18830900
H	–2.51023000	–2.21424200	0.24111400
C	–7.26346400	1.29697900	0.80722400
H	–7.97847400	1.08231300	1.59381200
C	–7.63860500	2.08614300	–0.27526700
H	–8.64455100	2.48515700	–0.33871500
C	–3.14284900	0.26052100	2.46345000
H	–3.48051700	1.29201500	2.51432000
C	–5.36532000	–1.61579500	–1.23115100
H	–5.24557900	–1.66757500	–0.14867300
H	–5.75466500	–2.56868200	–1.58865200
H	–6.09341900	–0.83285200	–1.44629900
C	–6.70816900	2.36703400	–1.27229400
H	–6.98716300	2.98991200	–2.11509800
C	–2.82877100	–0.41344200	3.63878300
H	–2.92342600	0.09122300	4.59461100
C	–2.26796700	–2.36852300	2.35844600
H	–1.91608800	–3.39320900	2.30514200
B	–3.51226200	0.47108500	–0.10184800
C	–2.39049100	–1.73564100	3.58947600
H	–2.14309000	–2.26467300	4.50313000
C	0.51219700	0.41103900	–1.72970300
C	1.06559400	–0.97735800	–1.47032300
H	1.86027600	–1.30759900	–2.13377200
C	0.73817300	–1.69266100	–0.39458100
H	–0.02653500	–1.30767500	0.27757900
C	1.42356800	–2.91652000	0.04540800
C	1.47740600	–3.18402200	1.41757400
C	2.08975400	–3.77205100	–0.83860500
C	2.20570700	–4.26617400	1.89820600
H	0.96643400	–2.51675000	2.10582200
C	2.80623100	–4.85994800	–0.35788900
H	2.04082500	–3.58566700	–1.90584900
C	2.87255200	–5.10651500	1.01209900
H	2.25306400	–4.45121700	2.96502300
H	3.31269400	–5.52056500	–1.05230200
H	3.43546200	–5.95464800	1.38389700
H	0.17531300	2.55114500	–0.56697700

O	1.31769200	1.39060900	–1.04062500
H	0.63570200	0.64422800	–2.79488200
Si	2.73690700	1.16758900	–0.14927800
C	2.34612200	0.44472200	1.53129400
C	3.36666800	–0.06876100	2.34188700
C	1.04758500	0.49401400	2.05425400
C	3.09546700	–0.54840300	3.61945700
H	4.38967600	–0.09478900	1.97707900
C	0.77374100	0.03006100	3.33689000
H	0.23087500	0.89440800	1.45890900
C	1.79695800	–0.50061700	4.11719000
H	3.89708100	–0.95299700	4.22662200
H	–0.23929600	0.07732200	3.71571300
H	1.58359100	–0.86919200	5.11432500
C	3.35279900	2.92025200	0.07536300
C	4.65598800	3.31758600	–0.24227700
C	2.48964200	3.87019400	0.64050200
C	5.07922400	4.62562500	–0.01937400
H	5.35343000	2.60085100	–0.66442300
C	2.90522200	5.17800700	0.85694200
H	1.48124700	3.58833800	0.93413500
C	4.20300300	5.55781100	0.52403700
H	6.09294600	4.91548700	–0.27121600
H	2.22069600	5.89825300	1.28968400
H	4.53112000	6.57661200	0.69487700
C	3.95435400	0.13105600	–1.12522800
C	4.33992800	–1.15676700	–0.73152600
C	4.42220600	0.61603400	–2.35512400
C	5.17156700	–1.93081900	–1.53525600
H	3.96361700	–1.58044700	0.19497300
C	5.25125200	–0.15606900	–3.16280700
H	4.12861400	1.60730300	–2.69065200
C	5.62885200	–1.43111300	–2.75060900
H	5.44947000	–2.92851600	–1.21488500
H	5.60161900	0.23437100	–4.11142700
H	6.27477900	–2.03488300	–3.37797100

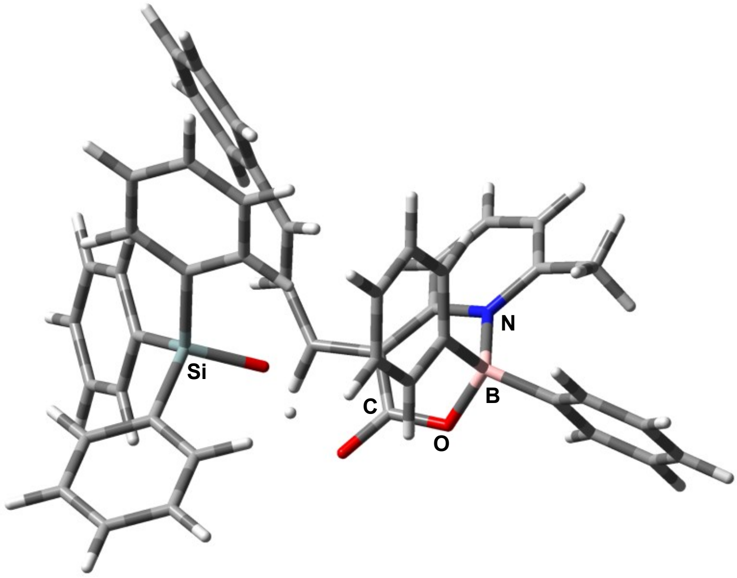

Figure S139. Optimized structure **TS*E*** [M06-2X/6–311+G(d,p)].

Table S70. Cartesian coordinates of the optimized structure **TS*E*** [M06-2X/6–311+G(d,p)].

Atomic symbol	x	y	z
O	–3.01260500	0.99596600	–1.45291600
O	–1.03171700	1.89604500	–1.88239200
N	–3.20197100	–1.36770800	–0.65591900
C	–1.72942200	0.87824400	–1.64112800
C	–1.98084400	–1.56107400	–1.24958100
C	–5.18844500	0.47603300	–0.46662600
C	–3.97935100	–2.43259400	–0.30553700
C	–2.92800200	0.58783200	1.07054900
C	–1.61779200	–2.86659900	–1.64643600
H	–0.70874500	–3.00712900	–2.20881500
C	–1.14114400	–0.42403000	–1.52880200
C	–5.95867000	0.96091400	0.59208000
H	–5.48765500	1.13625500	1.55555000
C	–3.59638400	–3.72107800	–0.62425500
H	–4.22957100	–4.54515500	–0.32628400
C	–5.82693600	0.28553800	–1.69818800
H	–5.24374400	–0.05667000	–2.54891300
C	–2.41754400	–3.93490600	–1.33617000
H	–2.13462600	–4.93520500	–1.64306000
C	–2.69281700	–0.31177800	2.11639000
H	–2.90572800	–1.36914500	1.98116500
C	–7.32158700	1.20941200	0.44507700
H	–7.89856300	1.58185600	1.28435600
C	–7.94298100	0.98073900	–0.77822400
H	–9.00409400	1.16887300	–0.89618100

C	–2.59517400	1.93158900	1.29706000
H	–2.75314600	2.65174200	0.49911400
C	–5.24548800	–2.24537600	0.48640700
H	–5.12255500	–1.50854300	1.27861000
H	–5.51220900	–3.20326800	0.93274500
H	–6.07013000	–1.90908200	–0.14376100
C	–7.18815200	0.52326800	–1.85609700
H	–7.66128500	0.35973900	–2.81827800
C	–2.07127300	2.35944700	2.51359400
H	–1.82062800	3.40466500	2.65880500
C	–2.18891600	0.10939400	3.34605400
H	–2.04032800	–0.61213200	4.14302800
B	–3.60884500	0.18607300	–0.34295200
C	–1.87750700	1.44948900	3.54957600
H	–1.47993100	1.78316200	4.50175100
C	0.27131300	–0.45515300	–1.80987200
C	1.16823900	–1.56505900	–1.44384700
H	1.92139400	–1.83713600	–2.17738900
C	1.20628200	–2.06863700	–0.20478700
H	0.49781000	–1.68933200	0.53051300
C	2.21151800	–3.01977200	0.28170300
C	2.50352800	–3.04622400	1.64913100
C	2.94360800	–3.84342100	–0.58306000
C	3.52286200	–3.85453700	2.13921600
H	1.94658500	–2.40309400	2.32232400
C	3.95346000	–4.65770000	–0.09132100
H	2.71842700	–3.84877100	–1.64374900
C	4.25106100	–4.66050900	1.27093900
H	3.74850900	–3.84944400	3.19923400
H	4.51047100	–5.29484900	–0.76838400
H	5.04324100	–5.29447200	1.65128300
H	0.13589500	1.59399700	–1.29617200
O	0.87649000	0.94171700	–0.83087200
H	0.57448500	0.05683900	–2.72117600
Si	2.41185900	1.27363900	–0.23639100
C	2.48494600	0.58170900	1.49853700
C	3.70946600	0.33583100	2.13257400
C	1.30403400	0.39174800	2.22996900
C	3.75946500	–0.11059400	3.44916100
H	4.64040500	0.49373400	1.59518700

C	1.35276500	–0.04270500	3.55202000
H	0.33725300	0.58339300	1.76642700
C	2.57838300	–0.30073700	4.16010600
H	4.71667200	–0.30306600	3.92014900
H	0.43035400	–0.17875000	4.10287100
H	2.61255600	–0.64225000	5.18861400
C	2.55259100	3.13735500	–0.24631400
C	3.78139100	3.78094400	–0.43291900
C	1.41478200	3.92162800	–0.01529000
C	3.87350000	5.16851400	–0.39049900
H	4.67602300	3.19473300	–0.62354800
C	1.50347900	5.30892500	0.02302500
H	0.44663300	3.44909700	0.13106500
C	2.73338000	5.93283000	–0.16364200
H	4.83173500	5.65285500	–0.53936700
H	0.61287700	5.90211100	0.19471700
H	2.80228600	7.01418800	–0.13548600
C	3.69982900	0.50784500	–1.36319900
C	4.37885200	–0.67865000	–1.05648200
C	3.92110000	1.09123400	–2.61890500
C	5.24734900	–1.26364100	–1.97320700
H	4.21652000	–1.16957700	–0.10129600
C	4.78550000	0.50786800	–3.53999000
H	3.41076700	2.01429400	–2.88127400
C	5.45043600	–0.67110900	–3.21639100
H	5.75989600	–2.18376900	–1.71568000
H	4.94132200	0.97257500	–4.50678800
H	6.12731000	–1.12513400	–3.93121300

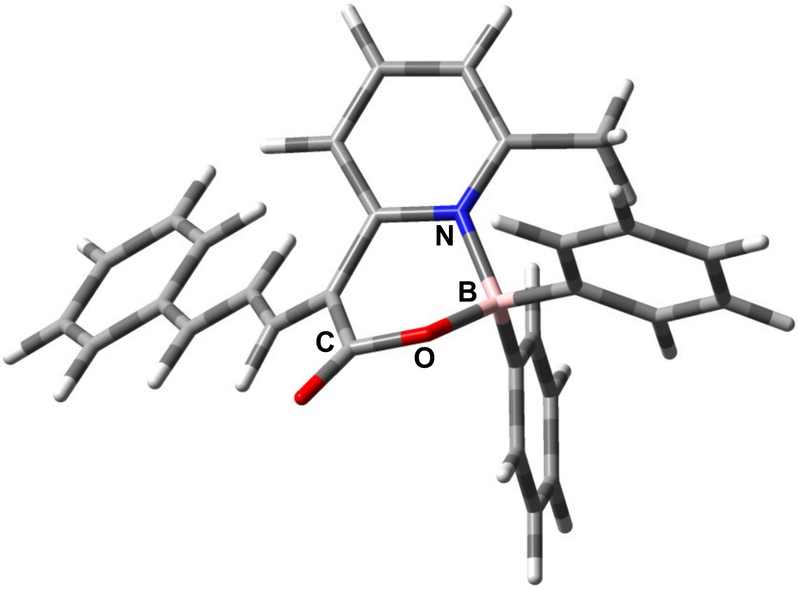

Figure S140. Optimized structure ***E*-11** [M06-2X/6–311+G(d,p)].

Table S71. Cartesian coordinates of the optimized structure ***E*-11** [M06-2X/6–311+G(d,p)].

Atomic symbol	x	y	z
O	–1.91538600	0.28476800	1.83850500
O	–0.45344000	0.83831900	3.42486000
N	–1.27578800	–1.16166300	–0.09720000
C	–4.44844200	0.79816500	–0.72458200
H	–3.94083400	1.54454700	–1.32933200
C	–3.70776900	0.03376700	0.17994200
C	–0.70515400	0.34611400	2.35687500
C	–1.44275600	1.43537500	–0.42061900
C	–0.09480600	–1.33329600	0.55355200
C	0.34472300	–0.29531400	1.49645900
C	–1.65370700	–2.03883900	–1.05600300
C	–5.82058000	0.61405300	–0.87749400
H	–6.37341400	1.21763900	–1.58869900
C	–4.40109900	–0.89537700	0.96480600
H	–3.85629900	–1.47004100	1.70858300
C	–2.84391500	–1.77385900	–1.93255200
H	–3.78296200	–1.97888000	–1.41628500
H	–2.77602000	–2.41424600	–2.81171900
H	–2.86921900	–0.73219600	–2.25081400
C	0.66805600	–2.48354800	0.36458400
H	1.56064100	–2.62103800	0.95936000
C	–0.74243700	1.31970100	–1.62569900
H	–0.59105900	0.34108900	–2.07595900
C	–1.59026300	2.72233400	0.11557100
H	–2.12378200	2.84264300	1.05401200

C	–6.48320000	–0.34274900	–0.11569200
H	–7.55019000	–0.49299300	–0.23514500
C	–0.89719500	–3.18507500	–1.28818200
H	–1.22916100	–3.87714100	–2.05042800
C	–5.76982900	–1.09412000	0.81570900
H	–6.28380000	–1.82615600	1.42872400
C	0.25222200	–3.42831200	–0.55559600
H	0.82322500	–4.33601400	–0.71145800
C	–0.37099000	3.69904800	–1.72160000
H	0.04301600	4.56822900	–2.21946000
C	–1.06268000	3.84025300	–0.52128500
H	–1.18803300	4.82318000	–0.08073900
C	–0.21180200	2.43350100	–2.27391500
H	0.32802100	2.31255200	–3.20661500
B	–2.12024000	0.20241900	0.36586900
C	2.75664700	–0.18336300	0.79904100
H	2.59624200	–0.78130400	–0.09154400
C	3.98220800	0.28627000	1.09327800
H	4.09093300	0.88475400	1.99568700
C	1.61580700	0.13453000	1.62762500
H	1.77443800	0.86025500	2.42236400
C	5.21402000	0.07547700	0.32947900
C	6.41669900	0.57374700	0.84405600
C	5.24327400	–0.59880800	–0.89916300
C	7.61402300	0.39939500	0.16113300
H	6.40680100	1.10351800	1.79063300
C	6.43745400	–0.77280000	–1.58126700
H	4.32650400	–0.98209200	–1.33124800
C	7.62824400	–0.27576700	–1.05370600
H	8.53430500	0.79249600	0.57640300
H	6.44287100	–1.29460500	–2.53081900
H	8.55958300	–0.41238000	–1.58989600

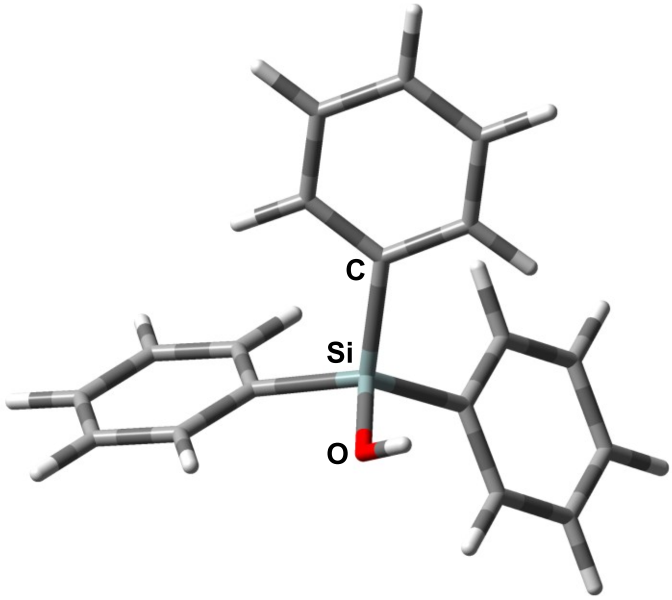

Figure S141. Optimized structure **Ph₃SiOH** [M06-2X/6–311+G(d,p)].

Table S72. Cartesian coordinates of the optimized structure **Ph_3SiOH** [M06-2X/6–311+G(d,p)].

Atomic symbol	x	y	z
O	–0.10874800	–0.19978100	2.44544900
Si	–0.00869600	–0.06306600	0.78803500
C	–0.05826700	1.73155600	0.24152700
C	–1.25830900	2.32856000	–0.16415200
C	1.09916700	2.52287500	0.26642100
C	–1.30381300	3.67117900	–0.52725300
H	–2.16665200	1.73434100	–0.20297500
C	1.05785300	3.86622900	–0.09145100
H	2.04887200	2.08111700	0.55670100
C	–0.14570800	4.44108400	–0.48921600
H	–2.24061900	4.11545200	–0.84333200
H	1.96277800	4.46259200	–0.06782900
H	–0.17909100	5.48655400	–0.77402800
C	–1.50555400	–0.97199300	0.14603700
C	–1.60878600	–1.29775500	–1.21178800
C	–2.57112300	–1.29511400	0.99303000
C	–2.74629100	–1.91977100	–1.71324400
H	–0.78959400	–1.06690200	–1.88774300
C	–3.71101900	–1.92069000	0.49492400
H	–2.50092000	–1.05703000	2.04919700
C	–3.80023400	–2.23043700	–0.85792100
H	–2.81099000	–2.16568500	–2.76685800
H	–4.52888100	–2.16731300	1.16217600
H	–4.68817600	–2.71569000	–1.24652900
C	1.59281200	–0.82496100	0.18701600

C	2.15777100	–0.46689100	–1.04369000
C	2.23238100	–1.81124200	0.94806700
C	3.32012900	–1.07770400	–1.50356000
H	1.69189900	0.30790400	–1.64677000
C	3.39874300	–2.41996400	0.49527100
H	1.81165600	–2.10271200	1.90564800
C	3.94215200	–2.05464600	–0.73233300
H	3.74388000	–0.78788600	–2.45820100
H	3.88323400	–3.17932800	1.09824400
H	4.85033300	–2.52854800	–1.08670800
H	0.35256200	0.44240300	2.98682100

6. References

[131] Thomas, J. C.; Peters, J. C. Bis(phosphino)borates: A New Family of Monoanionic Chelating Phosphine Ligands. *Inorg. Chem.* **2003**, *42*, 5055-5073.

[132] Espinosa-Jalapa, N. A.; Berg, N.; Seidl, M.; Shenderovich, I. G.; Gschwind, R. M.; Bauer, J. O. Complexation behaviour of LiCl and $LiPF_6$ – model studies in the solid-state and in solution using a bidentate picolyl-based ligand. *Chem. Commun.* **2020**, *56*, 13335-13338.

[133] Sinha, J.; Soars, S.; Bowman, C. N. Enamine Organocatalysts for the Thiol-Michael Addition Reaction and Cross-Linking Polymerizations. *Macromolecules* **2021**, *54*, 1693-1701.

[134] The crude isolated brown liquid was further purified by distillation under vacuum (40 °C, 1 x 10^{-2} mbar) yielding the enamine as a clear oil that was stored over molecular sieves (3Å) at 0 °C within a glovebox. ^{1}H NMR (500.18 MHz, C_6D_6, 25 °C): δ 1.49 (m, 4H, 2 x CH_2), 1.92 (quint, $^3J_{HH}$ = 7.4 Hz, 2H, CH_2), 2.37 (m, 2H, CH_2), 2.58 (m, 2H, CH_2), 2.90 (m, 4H, 2 x CH_2), 4.25 (bs, 1H, C*H*).

[135] Rigaku Oxford Diffraction, *CrysAlisPro Software System*, 2023.

[136] Dolomanov, O. V.; Bourhis, L. J.; Gildea, R. J.; Howard, J. A. K.; Puschmann, H. *OLEX2*: a complete structure solution, refinement and analysis program. *J. Appl. Crystallogr.* **2009**, *42*, 339-341.

[137] Sheldrick, G. M. *SHELXT* – Integrated space-group and crystal-structure determination. *Acta Crystallogr., Sect. A: Found. Adv.* **2015**, *71*, 3-8.

[138] Sheldrick, G. M. Crystal structure refinement with *SHELXL*. *Acta Crystallogr., Sect. C: Struct. Chem.* **2015**, *71*, 3-8.

[139] Macrae, C. F.; Edgington, P. R.; McCabe, P.; Pidcock, E.; Shields, G. P.; Taylor, R.; Towler, M.; van de Streek, J. *Mercury*: visualization and analysis of crystal structures. *J. Appl. Crystallogr.* **2006**, *39*, 453-457.

[140] Höpfl, H. The tetrahedral character of the boron atom newly defined—a useful tool to evaluate the N→B bond. *J. Organomet. Chem.* **1999**, *581*, 129-149.

[141] Dennington, R. D., II; Keith, T. A.; Millam, J. M. *GaussView 5.0*; Gaussian, Inc.: Wallingford, CT, USA, 2008.

[142] Foresman, J. B.; Frisch, A. *Exploring Chemistry with Electronic Structure Methods*, 2nd ed.; Gaussian, Inc.: Pittsburgh, PA, USA, 1996.

[143] Bernardi, A.; Gennari, C.; Goodman, J. M.; Paterson, I. The Rational Design and Systematic Analysis of Asymmetric Aldol Reactions Using Enol Borinates: Applications of Transition State Computer Modelling. *Tetrahedron: Asymmetry* **1995**, *6*, 2613-2636.

[144] Paton, R. S.; Goodman, J. M. Understanding the Origins of Remote Asymmetric Induction in the Boron Aldol Reactions of β-Alkoxy Methyl Ketones. *Org. Lett.* **2006**, *8*, 4299-4302.

[145] Goodman, J. M.; Paton, R. S. Enantioselectivity in the boron aldol reactions of methyl ketones. *Chem. Commun.* **2007**, 2124-2126.

[146] Paton, R. S.; Goodman, J. M. 1,5-Anti Stereocontrol in the Boron-Mediated Aldol Reactions of *β*-Alkoxy Methyl Ketones: The Role of the Formyl Hydrogen Bond. *J. Org. Chem.* **2008**, *73*, 1253-1263.
